# Predicting Cloned Disease Resistance Gene Homologs (CDRHs) in Radish, Underutilised Oilseeds, and Wild Brassicaceae Species

**DOI:** 10.3390/plants11223010

**Published:** 2022-11-08

**Authors:** Aldrin Y. Cantila, William J. W. Thomas, Philipp E. Bayer, David Edwards, Jacqueline Batley

**Affiliations:** School of Biological Sciences, The University of Western Australia, Perth 6009, Australia

**Keywords:** Brassicaceae cultivated and weedy species, resistance gene analogs and homologs

## Abstract

Brassicaceae crops, including *Brassica*, *Camelina* and *Raphanus* species, are among the most economically important crops globally; however, their production is affected by several diseases. To predict cloned disease resistance (*R*) gene homologs (CDRHs), we used the protein sequences of 49 cloned *R* genes against fungal and bacterial diseases in Brassicaceae species. In this study, using 20 Brassicaceae genomes (17 wild and 3 domesticated species), 3172 resistance gene analogs (RGAs) (2062 nucleotide binding-site leucine-rich repeats (NLRs), 497 receptor-like protein kinases (RLKs) and 613 receptor-like proteins (RLPs)) were identified. CDRH clusters were also observed in *Arabis alpina*, *Camelina sativa* and *Cardamine hirsuta* with assigned chromosomes, consisting of 62 homogeneous (38 NLR, 17 RLK and 7 RLP clusters) and 10 heterogeneous RGA clusters. This study highlights the prevalence of CDRHs in the wild relatives of the Brassicaceae family, which may lay the foundation for rapid identification of functional genes and genomics-assisted breeding to develop improved disease-resistant Brassicaceae crop cultivars.

## 1. Introduction

The Brassicaceae family, also known as Cruciferae due to its cross-shape four-petal flower [1], is one of the most diverse and agronomically important plant families, consisting of 44 tribes, 372 genera and 4060 species [2,3]. The *Brassica* species (*B. rapa*, *B. nigra*, *B. oleracea*, *B. juncea*, *B. napus* and *B. carinata*), *Camelina sativa*, *Raphanus sativus* and *Sinapis alba* are crop members, which are produced for vegetables, edible oil, herbs, spices, condiments and fodder. The Brassicaceae also contains many model species that are used in various areas of research, including *Arabidopsis thaliana* for genetic studies [4], *Arabidopsis halleri* for heavy metal (e.g., cadmium and zinc) accumulation and tolerance [5], *Arabis alpina* in ecological studies [6], *Barbarea vulgaris* for insect resistance [7], *Boechera* species in apomixis research [8], *Brassica* species in crop evolution [9], *C. sativa* in metabolic oils [10], *Cardamine hirsuta* in leaf structure and morphology [11], *Eutrema salsugineum* in salinity stress [12] and *Lepidium meyenii* in floral structure [13]. In addition, species, such as *Amoracia rusticana*, *Cheiranthes cheiri*, *Isatis tinctoria*, *Matthiola incana* and *Raphanus raphanistrum*, have industrial uses (biofuels, dyes, etc.) [14,15,16,17,18], while species in the genera *Aethionema*, *Cheiranthus*, *Erysimum*, *Hesperis*, *Iberis*, *Lobularia*, *Lunaria*, *Malcolmia* and *Matthiola* are cultivated as ornamentals [19,20].

The production of Brassicaceae species, especially the crop members, is limited by various pathogens, such as *Leptosphaeria* species *(L. maculans*, *L. biglobosa*), *Sclerotinia sclerotiorum*, *Albugo candida*, *Hyaloperonospora* species (*H. parasitica*, *H. arabidopsidis*), *Pseudomonas syringae*, *Plasmodiophora brassicae*, *Xanthomonas* spp., *Fusarium oxysporum matthioli*, *Botrytis cinerea*, *Erysiphe cichoracearum* and *Alternaria* species (*A. brassicicola*, *A. brassicae*), which cause blackleg, Sclerotinia stem rot, white rust, downy mildew, bacterial leaf spot, clubroot, black rot, Fusarium wilt, grey mould, powdery mildew and Alternaria black spot diseases, respectively [21,22,23,24,25]. Crops have qualitative and quantitative disease resistance to overcome pathogens. The quantitative resistance, governed by many minor genes, is a partial resistance manifesting at later stages of the crop, while qualitative resistance, governed by major genes or resistance genes (*R* genes), is largely manifested from the early stages up to the maturity stage of the crop. Among the types of resistance in Brassicaceae crops, qualitative resistance is commonly used to screen lines in early stages of the genotypes for disease resistance breeding and development. For instance, a set of pathogen isolates containing avirulence (*Avr*) genes is used to screen white rust resistance in *B. juncea* genotypes [26] and blackleg resistance in *B. napus* genotypes [27,28] by assessing a hypersensitive response observed in the cotyledons. Clubroot resistance is also screened either in the cotyledon and roots of the seedlings in Brassicaceae species [29,30,31,32].

The crop wild relatives (CWRs) of the cultivated Brassicaceae species can be used to improve disease resistance by integrating favourable alleles harboured by the CWRs into the crop members. For example, *Brassica fruticulosa* and *Erucastrum cardaminoides* were introgressed, via wide hybridization, including chromosome doubling and bridging species, to *B. juncea* with disease *R* genes against Sclerotinia stem rot [33,34]. In addition, *B. juncea*-*S. alba* hybrids were developed through somatic hybridization, which leads to the transfer of Alternaria black spot disease resistance to *B. juncea* [35]. The wild C genome of *Brassica incana* was also introduced to *B. napus* through interspecific hybridization and pyramiding for Sclerotinia stem rot resistance [36], while Alternaria black spot and white rust resistance from the wild crucifers *Diplotaxis erucoides* and *Brassica maurorum* were introduced into *B. rapa* with the aid of sequential ovary-ovule culture [37]. Lastly, *A. thaliana*, *B. insularis*, *B. atlantica*, *B. macrocarpa*, *Diplotaxis muralis*, *Eruca pinnatifia*, *Erucastrum gallicum*, *R. raphanistrum*, *Sinapsis arvensis*, *Sisymbrium loeselii* and *Thlaspi arvense* have been found with proteins/compounds that may enhance blackleg resistance in *B. napus* [38,39,40,41,42,43,44,45].

Plant disease *R* genes, also called resistance-gene analogs (RGAs), play a significant role in triggering the genetic resistance-defence response in crops [46] and are grouped into three main classes: nucleotide-binding site (NBS)-leucine rich repeats (LRR) (NLRs), receptor-like protein kinases (RLKs) and receptor-like proteins (RLPs). NLRs, with the subclasses coiled-coil (CC)-NBS (CN), CNL, NBS, NBS-LRR (NL), Toll/Interleukin-1 receptor (TIR)-NBS-LRR (TNL), TIR-NBS (TN), TIR with unknown domains (TX), NLR with other domains (Other-NLR), are generally involved in effector-triggered plant immunity (ETI) and plant defence [47,48,49,50]. On the other hand, RLKs, with the subclasses, including LRR-RLK, Lysin motif (LsyM) (LysM-RLK) and other receptor (Other-RLK) [51] and RLPs, with the subclasses, including LRR-RLP and LysM (LysM-RLP), are not only involved in the first line of defence by recognising pathogen elicitors [52,53], but also in plant development [54,55].

This study aimed to determine what RGAs are homologous to cloned fungal and bacterial *R* genes across 20 Brassicaceae genomes and to assess the retention and diversification of RGA domains in the homologs and their physical clustering patterns.

## 2. Results

### 2.1. Prediction of RGAs in Brassica cretica, Capsella bursa-pastoris and Sinapis alba

**Figure 1:**
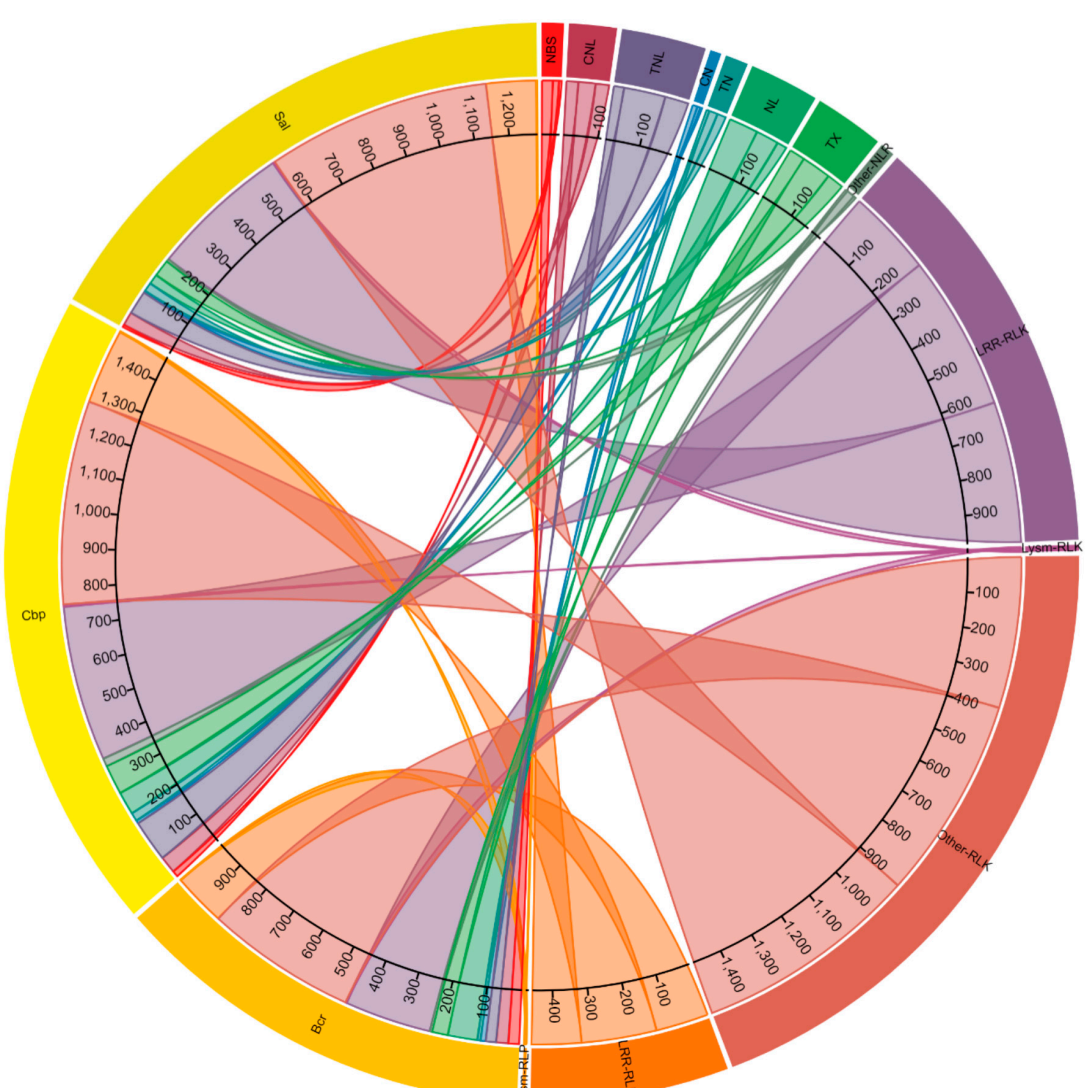
The number and distribution of resistance-gene analog (RGA) subclass nucleotide-binding site (NBS), coiled-coil (CC)-NBS or CN, CN-leucine rich repeats (LRR) or CNL, NBS-LRR or NL, Toll/Interleukin-1 receptor (TIR)-NBS-LRR or TNL, TIR-NBS or TN, TIR with unknown domains or TX, NBS-LRR with other domains or Other-NLR, LRR- receptor like kinase (RLK) or LRR-RLK, Lysin motif (LsyM)-RLK or LysM-RLK, RLK with other receptor or Other-RLK, LRR- receptor-like protein (RLP) or LRR-RLP and LysM-RLP in *Brassica cretica* (Bcr), *Capsella bursa-pastoris* (Cbp) and *Sinapis alba* (Sal) genomes.

RGAugury predicted a combined total of 3738 RGAs in *B. cretica* (982 RGAs; with 230 NLRs, 614 RLKs and 138 RLPs), *C. bursa-pastoris* (1474 RGAs; with 353 NLRs, 925 RLKs and 196 RLPs) and *S. alba* (1282 RGAs; with 208 NLRs, 943 RLKs and 131 RLPs) genomes (Figure 1, Table S1). Of these RGAs, 791 were NLRs (195 TNL, 161 NL, 161 TX, 110 CNL, 53 TN, 51 NBS, 26 CN and 34 Other-NLR), 2482 were RLKs (1486 Other-RLK, 982 LRR-RLK and 14 LysM-RLK) and 465 were RLPs (457 LRR-RLP and 8 LysM-RLP) (Figure 1).

### 2.2. Identification of CDRHs across the Study Species and Diseases

**Figure 2:**
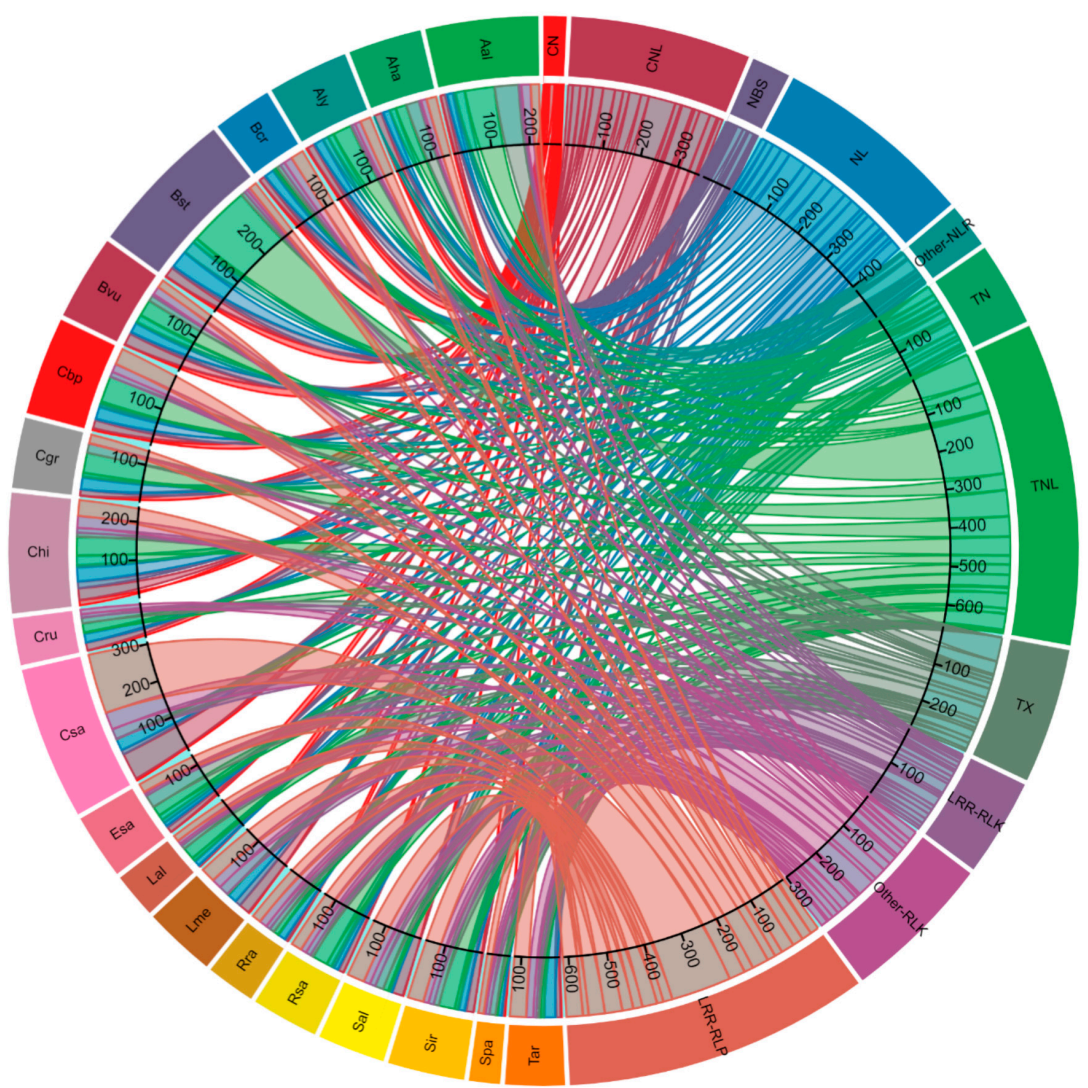
The number and distribution of cloned disease resistance gene homologs containing resistance domains including nucleotide-binding site (NBS), coiled-coil (CC)-NBS or CN, CN-leucine rich repeats (LRR) or CNL, NBS-LRR or NL, Toll/Interleukin-1 receptor (TIR)-NBS-LRR or TNL, TIR-NBS or TN, TIR with unknown domains or TX, NBS-LRR with other domains or Other-NLR, LRR- receptor like kinase (RLK) or LRR-RLK, Lysin motif (LsyM)-RLK or LysM-RLK, RLK with other receptor or Other-RLK, LRR- receptor like protein (RLP) or LRR-RLP and LysM-RLP in *Arabidopsis halleri* (Aha), *Arabidopsis lyrata* (Aly), *Arabis alpina* (Aal), *Barbarea vulgaris* (Bvu), *Boechera stricta* (Bst), *Brassica cretica* (Bcr), *Camelina sativa* (Csa), *Capsella grandiflora* (Cgr), *Capsella bursa-pastoris* (Cbp), *Capsella rubella* (Cru), *Cardamine hirsuta* (Chi), *Eutrema salsugineum* (Esa), *Leavenworthia alabamica* (Lal), *Lepidium meyenii* (Lme), *Raphanus raphanistrum* (Rra), *Raphanus sativus* (Rsa), *Sinapis alba* (Sal), *Sisymbrium irio* (Sir), *Schrenkiella parvula* (Spa) and *Thlaspi arvense* (Tar) genomes.

The 3172 cloned disease *R* gene homologs (CDRHs) identified were all RGAs: 2062 NLRs, 497 RLKs and 613 RLPs, with an average of 159 CDRHs (RGAs) in each of the 20 studied genomes/species (Figure 2, Table S2). *C. sativa* contained the highest number of CDRHs: 307, followed by *Boechera stricta* (296), *C. hirsuta* (240), *A. alpina* (226), *C. bursa-pastoris* (197), *B. vulgaris* (171) and *Arabidopsis lyrata* (162) (Figure 2). The rest of the studied Brassicaceae contained less than the average CDRHs per species, with the lowest in *Schrenkiella parvula* (62), *Leavenworthia alabamica* (91), *Capsella rubella* (94) and *R. raphanistrum* (99) (Figure 2). It should also be noted that *A. lyrata*, *C. bursa-pastoris* and *R. sativus* (135 CDRHs) had the highest number of CDRHs in their respective subfamilies (Figure 2).

**Figure 3:**
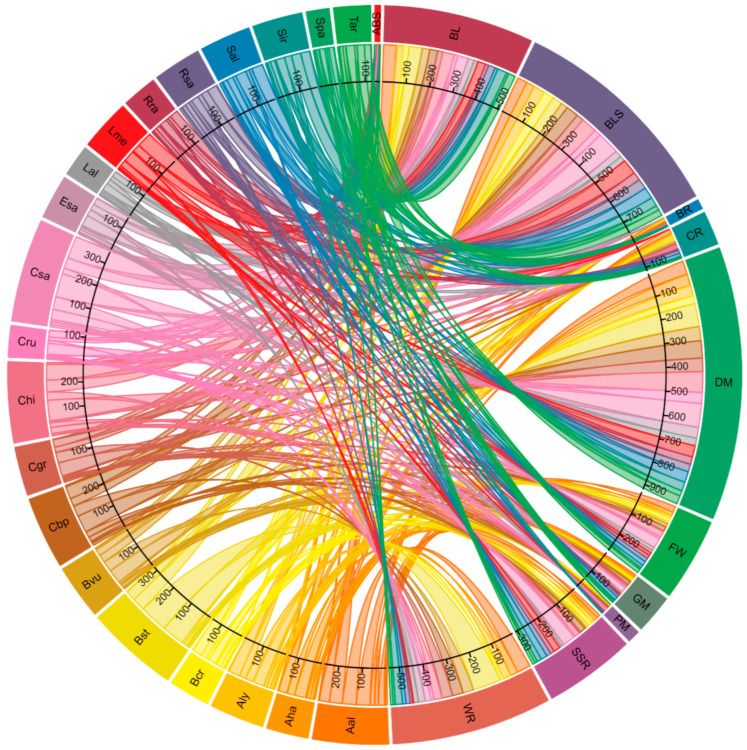
The number and distribution of cloned disease resistance gene homologs associated to Alternaria black spot (ABS), blackleg (BL), black rot (BR), bacterial leaf spot (BLS), clubroot (CR), downey mildew (DM), Fusarium wilt (FW), grey mould (GM), powdery mildew (PM), Sclerotinia stem rot (SSR) and white rust (WR) resistance in *Arabidopsis halleri* (Aha), *Arabidopsis lyrata* (Aly), *Arabis alpina* (Aal), *Barbarea vulgaris* (Bvu), *Boechera stricta* (Bst), *Brassica cretica* (Bcr), *Camelina sativa* (Csa), *Capsella grandiflora* (Cgr), *Capsella bursa-pastoris* (Cbp), *Capsella rubella* (Cru), *Cardamine hirsuta* (Chi), *Eutrema salsugineum* (Esa), *Leavenworthia alabamica* (Lal), *Lepidium meyenii* (Lme), *Raphanus raphanistrum* (Rra), *Raphanus sativus* (Rsa), *Sinapis alba* (Sal), *Sisymbrium irio* (Sir), *Schrenkiella parvula* (Spa) and *Thlaspi arvense* (Tar) genomes.

The cloned *R* genes against bacterial leaf spot (*At_ADR1*, *At_BAK1*, *At_FLS2*, *At_NDR1*, *At_NRG1a*, *At_NRG1b*, *At_PBS1*, *At_RLP30*, *At_RLP32*, *At_RPM1*, *At_RPS2*, *At_RPS4*, *At_RPS5*, *At_RRS1* and *At_SOBIR1*) had a total of 752 CDRHs (Figure 3). *C. sativa* had the highest number of CDRHs, 85, followed by 59 and 58 in *C. hirsuta* and *L. meyenii*, respectively (Figure 3). For the gene conferring resistance to another bacterial disease (black rot), *At_RLP1*, a total of 36 CDRHs were identified, with the highest numbers found in *C. hirsuta* and *C. bursa-pastoris* with 6 and 5, respectively (Figure 3).

In total, 921 CDRHs associated with cloned *R* genes against the fungal disease downey mildew (*At_ADR1*, *At*_*NRG1a*, *At_NRG1b*, *At_RLP42*, *At_RPP1*, *At_RPP2a*, *At_RPP2B*, *At_RPP4*, *At_RPP5*, *At_RPP7*, *At_RPP8*, *At_RPP13* and *At_RPP39*) were identified (Figure 3). Of these, 89 and 86 CDRHs were the highest numbers obtained in *C. sativa* and *B. stricta*, respectively. The cloned *R* genes against white rust (*Bju_WRR1*, *At_RAC1*, *At_WRR4a*, *At_WRR4b*, *At_WRR8*, *At_WRR9* and *At_WRR12*) (Table 1) were recorded having a total of 544 CDRHs (Figure 3). The highest count was found in *B. stricta:* 106 CDRHs, followed by *A. alpina* (52 CDRHs). For blackleg, the cloned *R* genes (*Bna_MAPk*, *Bna_LepR3/Rlm2*, *Bna_Rlm9/4/7*, *At_RLM1a*, *At_RLM1b* and *At_RLM3*) had a total of 509 CDRHs (Figure 3). Both *A. alpina* and *B. stricta* had the most CDRHs, with 49 each, followed by *C. hirsuta* with 44 and *C. sativa* with 40. For Sclerotinia stem rot, the cloned *R* genes (*At_BAK1*, *At_RLP23*, *At_RLP30* and *At_SOBIR1*) had a total of 310 CDRHs with the highest count observed in *C. sativa* with 48 (Figure 3).

The cloned *R* genes (*Bol_FocBo1*, *At_RFO1*, *At_RFO2* and *At_RFO3*) against Fusarium wilt had 283 CDRHs in total with the highest numbers being 38 (*C. sativa*) and 23 CDRHs (*C. hirsuta* and *A. alpina*) (Figure 3). The cloned *R* genes against grey mould (*At_RLP42* and *At_RLM3*) had a total of 134 CDRHs with the highest CDRHs obtained in *C. sativa* (21 CDRHs), *S. alba* (13 CDRHs) and *C. bursa-pastoris* (13 CDRHs) (Figure 3). The cloned *R* genes (*Bra_Crr1a* and *cRa/cRb*) against clubroot had a total of 117 CDRHs with *A. alpina* and *S. alba* containing the highest counts with 17 and 12 CDRHs, respectively (Figure 3). *At_ADR1* against powdery mildew had 45 CDRHs, with 7 CDRHs in *C. sativa* as the highest count. *At_RLM3* conferring resistance to Alternaria black spot had 22 CDRHs with 2 CDRHs as the highest in each of eight species (*A. alpina*, *B. stricta*, *B. vulgaris*, *C. bursa-pastoris*, *Capsella grandiflora*, *E. salsugineum*, *R. sativus* and *T. arvense*) (Figure 3).

### 2.3. Retention and Diversification of RGA Domains in CDRHs

In terms of RGA subclasses, CDRHs were composed of 647 TNL, 613 LRR-RLP, 402 NL, 361 CNL, 301 Other-RLK, 271 TX, 196 LRR-RLK, 168 TN, 89 Other-NLR, 78 NBS and 46 CN (Figure 2), which shows the variation in CDRHs throughout the Brassicaceae family.

The RGA domain retention in the CDRHs (same RGA domain compared to its reference cloned *R* gene) and diversification (different RGA domain compared to its reference cloned *R* gene) were also noted in this study (Table 1). In total, 1992 (63%) and 1180 (37%) out of the 3172 CDRHs had retained and diversified their RGA domain compared to their reference cloned *R* gene, respectively (Table 1). It can be noted that the cloned *R* genes classed as Other-RLK had their corresponding CDRHs also classified as Other-RLK (100%, 298 out of 298 CDRHs). The next highest numbers retaining the same RGA domain were 98%, 95% and 61% in CDRHs from the LRR-RLK (167 out of 170 CDRHs), LRR-RLP (599 out of 628 CDRHs) and CNL (204 out of 332 CDRHs) cloned *R* genes, respectively. The remaining CDRHs from the NL, TNL and TN cloned *R* genes had 49% (115 out of 236 CDRHs), 45% (604 out of 1356 CDRHs) and 38% (5 out of 13 CDRHs) RGA domain retention, respectively.

The gene diversification could either be through truncation (one or two domains omitted), addition (one or two domains were added) or the combination of truncation and addition of RGA domains. Of the diversification results in CDRHs, 100% (130 CDRHs) of the CDRHs from RNL cloned *R* genes did not have an RNL domain. Diversification was also observed in CDRHs from cloned *R* genes that were TN (62% or 8 out of 13 CDRHs), TNL (55% or 752 out of 1356 CDRHs), NL (51% or 121 out of 236 CDRHs), CNL (49% or 128 out of 332 CDRHs), LRR-RLP (5% or 29 out of 628 CDRHs) and LRR-RLK (2% or 3 out of 170 CDRHs). Of the cloned *R* genes, which were NLs, all the CDRHs (29) had additional RGA domains, while for the LRR-RLP cloned *R* genes 59% (71 out of 121 diversified CDRHs) had an additional one or two RGA domains. On the other hand, the combination of truncation and addition of RGA domains was observed in CDRHs from cloned *R* genes TN (63% or 5 out 8 diversified CDRHs), TNL (55% or 411 out of 752 diversified CDRHs) and RNL (54% or 70 out of 130 diversified CDRHs).

### 2.4. Identification of CDRH Clusters in Arabis alpina, Camelina sativa and Cardamine hirsuta

**Figure 4:**
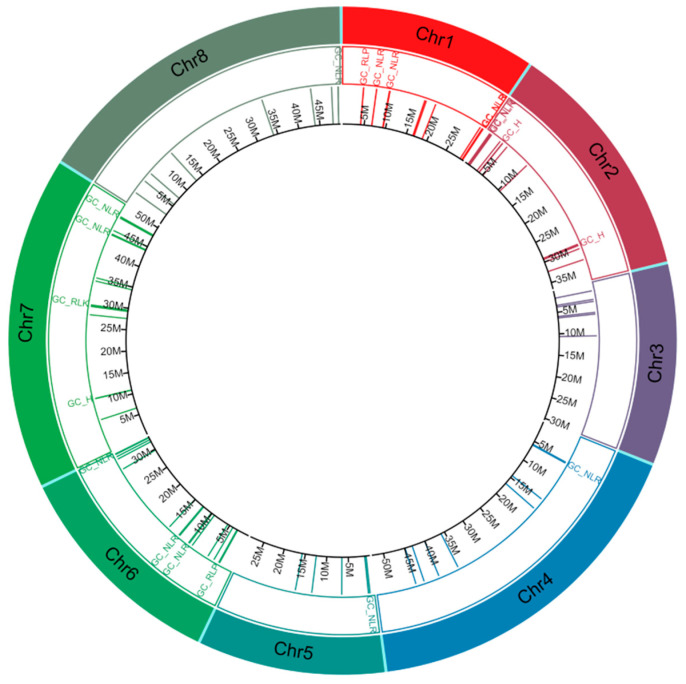
Distribution of cloned disease resistance gene homologs in *Arabis alpina* (1st inner layer in their corresponding position in *A. alpina* genome). The tracks in the circos plot, from outer to inner, show chromosome (Chr) number and types of gene cluster. GC_NLR = gene cluster (GC) with all nucleotide-binding site leucine rice repeats (NLR) members, GC_RLP = GC with all receptor-like proteins (RLP) members, GC_RLK = GC with all receptor-like kinase proteins (RLK) members, GC_H = GC with members are a mixture of NLR, RLK and/or RLP, Chr = chromosome and M = position in million base pairs.

**Figure 5:**
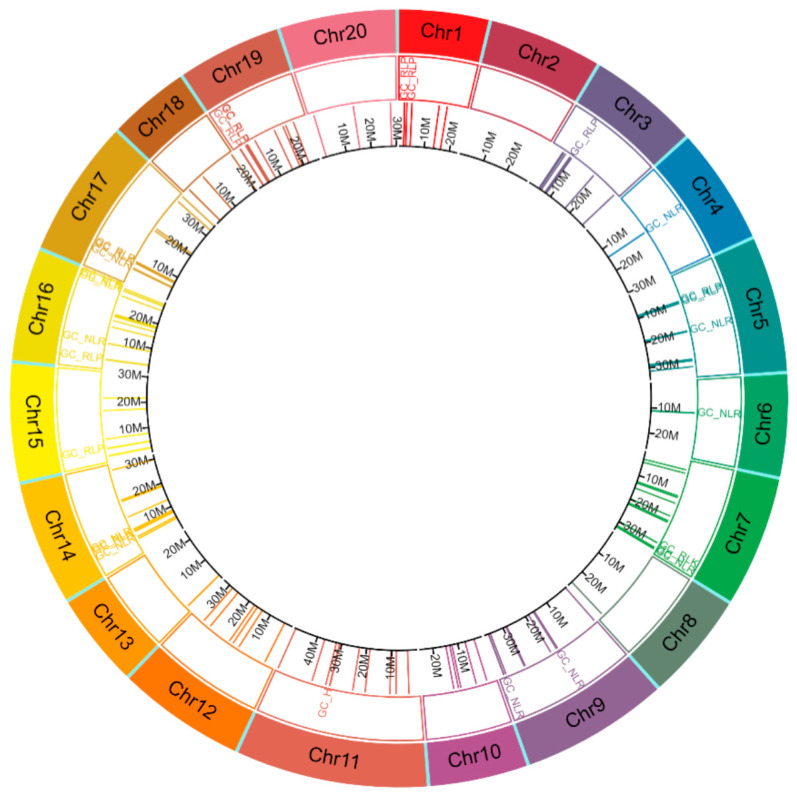
Distribution of cloned disease resistance gene homologs in *Camelina sativa* (1st inner layer in their corresponding position in *C. sativa* genome). The tracks in the circos plot, from outer to inner, show chromosome (Chr) number and types of gene cluster. GC_NLR = gene cluster (GC) with all nucleotide-binding site leucine rice repeats (NLR) members, GC_RLP = GC with all receptor-like proteins (RLP) members, GC_RLK = GC with all receptor-like kinase proteins (RLK) members, GC_H = GC with members are a mixture of NLR, RLK and/or RLP, Chr = chromosome and M = position in million base pairs.

**Figure 6:**
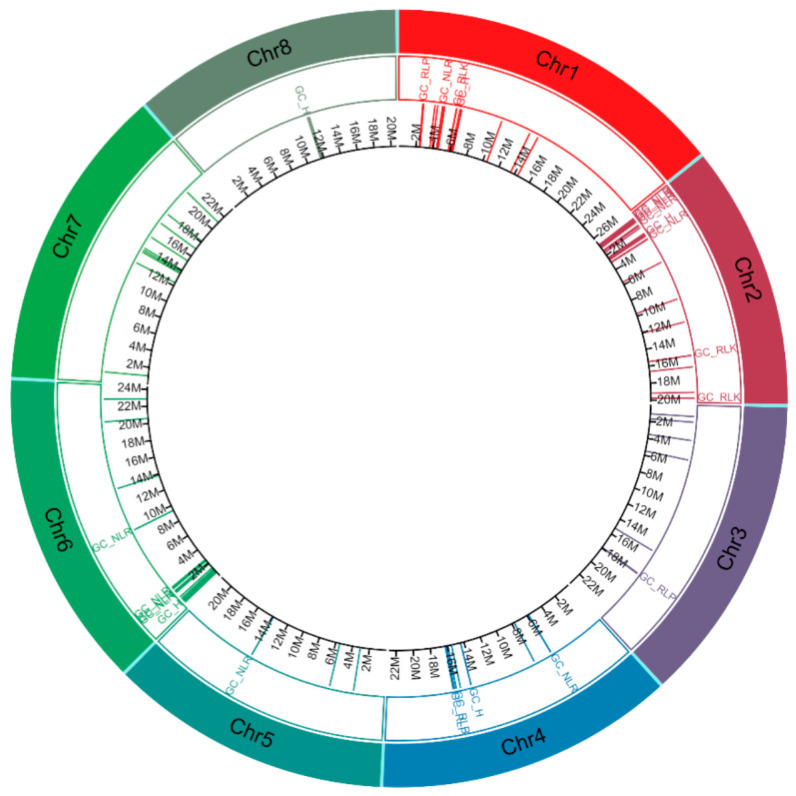
Distribution of cloned disease resistance gene homologs in *Cardamine hirsuta* (1st inner layer in their corresponding position in *C. hirsuta* genome). The tracks in the circos plot, from outer to inner, show chromosome (Chr) number and types of gene cluster. GC_NLR = gene cluster (GC) with all nucleotide-binding site leucine rice repeats (NLR) members, GC_RLP = GC with all receptor-like proteins (RLP) members, GC_RLK = GC with all receptor-like kinase proteins (RLK) members, GC_H = GC with members are a mixture of NLR, RLK and/or RLP, Chr = chromosome and M = position in million base pairs.

The organisation of CDRHs with RGA domains across chromosomes of *A. alpina*, *C. sativa* and *C. hirsuta* was studied to investigate the gene clustering of CDRHs in *Brassica* crop relatives. We identified a total of 72 gene clusters, consisting of 62 homogeneous RGA clusters (38 NLR, 17 RLK and 7 RLP clusters) and 10 heterogeneous RGA clusters (Figure 4, Figure 5 and Figure 6). *C. sativa* contained the highest number of gene clusters with 28 (Figure 5), followed by *C. hirsuta* with 24 gene clusters (Figure 6) and *A. alpina* with 20 gene clusters (Figure 4).

## 3. Discussion

By aligning the 49 cloned R genes from 11 diseases, across 20 Brassicaceae genomes (crop species *C. sativa*, *R. sativus* and *S. alba* and wild species *A. halleri*, *A. lyrata*, *A. alpina*, *B. vulgaris*, *B. stricta*, *B. cretica*, *C. grandiflora*, *C. bursa-pastoris*, *C. rubella*, *C. hirsuta*, *E. salsugineum*, *L. alabamica*, *L. meyenii*, *R. raphanistrum*, *Sisymbrium irio*, *S. parvula* and *T. arvense*), an inventory of specific RGAs associated with cloned R genes was found. This provides an opportunity to search for novel CDRHs, which may confer disease resistance (especially the CDRHs in wild species), which can be used for future crop improvement once function is established in the crop species. Once cloned, molecular markers can be developed as a diagnostic tool in screening additional germplasm to characterise further lines for resistance.

The RGAs in *B. cretica*, *C. bursa-pastoris* and *S. alba* genomes and specific RGAs (CDRHs) obtained here are additional gene resources to the previously identified Brassicaceae RGA repertoire [51,56,57]. The number of *S. alba* RGAs in this study was higher than the RGAs obtained in the 18 species: *Aethionema arabicum*, *A. halleri*, *A. lyrata*, *A. thaliana*, *A. alpina*, *B. vulgaris*, *B. stricta*, *B. rapa*, *C. grandiflora*, *C. rubella*, *C. hirsuta*, *E. salsugineum*, *L. alabamica*, *R. raphanistrum*, *R. sativus*, *S. irio*, *S. parvula* and *T. arvense* genomes; the number of *C. bursa-pastoris* RGAs identified in this study was higher than the number of RGAs in the 21 species: *Aethionema arabicum*, *A. halleri*, *A. lyrata*, *A. thaliana*, *A. alpina*, *B. vulgaris*, *B. stricta*, *B. rapa*, *B. nigra*, *B. oleracea*, *C. grandiflora*, *C. rubella*, *C. hirsuta*, *E. salsugineum*, *L. alabamica*, *R. raphanistrum*, *R. sativus*, *S. irio*, *S. parvula* and *T. arvense* genomes [51,58]. Only the tetraploid *Brassica* crops (*B. juncea*, *B. napus* and *B. carinata*), *C. sativa* (hexaploid) and the wild species *L. meyenii* (octaploid) had greater numbers of RGAs than *C. bursa-pastoris* (tetraploid) [51,56], indicating that polyploidisation is a factor leading to more RGAs in species in the Brassicaceae family. Polyploid plants also have a greater number of transposable elements, an evolution driver of genome expansion [59,60], compared to its progenitors [61,62].

*Brassica* crops have experienced extensive breeding and development to improve disease resistance due to their long history of domestication that may have been a factor for RGA number expansion [63]. A previous study showed an average of 1563 RGAs in 11 genomes of the domesticated species compared to the average of 863 RGAs in 19 genomes of the wild species [51]; a similar trend was observed in this study between the domesticated and wild species. The number of RGAs in *B. cretica* (wild species) in this study was lower compared to the number of RGAs found in domesticated *Brassica* crops. This was also the case with the specific RGAs for *R. sativus* and *R. raphanistrum* (CDRHs in this study) and the RGAs obtained in a previous study [51], where domesticated radish had more RGAs compared to wild radish. However, this is not always the case as *B. macrocarpa* (wild cabbage species) had more RGAs compared to 10 domesticated cabbage species in pangenome analysis [58]. Here, the lesser RGAs in *B. cretica* and *R. raphanistrum* than their domesticated counterpart species may also be due to the quality of genomes, as domesticated crops often have better genome qualities.

The domesticated Brassicaceae members (used in this study) have also been reported as excellent sources of disease resistance. For instance, *C. sativa* has been reported to have *R* genes providing resistance against Alternaria black spot, blackleg, downey mildew and Sclerotinia stem rot [40,64,65], *R. sativus* has resistance against black rot [66], clubroot [67,68], downey mildew [69,70], Fusarium wilt [71], white rust [72] and Turnip mosaic virus [73,74] and *S. alba* has resistance to blackleg [39,75], Turnip mosaic virus [76] and Sclerotinia stem rot [77,78]. However, further investigation is needed as to whether the RGAs we identified in these three species are associated with the resistant phenotype. Nevertheless, our study supports the previous findings and the RGAs we identified are a valuable reference for future studies.

Unlike the cultivated crops, information towards genetic disease resistance in Brassicaceae wild species is limited. Of the wild Brassicaceae species we included, a few of them have been reported previously as potential *R* gene source against a particular disease, for instance, *B. vulgaris* against Alternaria black spot and black rot [79], *B. cretica* against Verticillium wilt disease [80], *C. bursa-pastoris* against clubroot [81], Sclerotinia stem rot [82] and Alternaria black spot [83], *R. raphanistrum* against blackleg [38], clubroot [84], downey mildew [85] and Sclerotinia stem rot [86] and *T. arvense* against blackleg [42]. However, the association between the reported phenotypic disease resistance in these species and the identified RGAs here needs further research.

The retention and diversification of RGA domains in the Brassicaceae family are a result of evolutionary events, such as whole-genome triplication/duplication [87,88,89,90,91]. Homologs may confer similar or dissimilar function to the reference gene [92,93]. A functional study revealed the *A. lyrata* homologs *AL.MTP11A* and *AL.MTP11B* are redundant to *AT.MTP11* in *A. thaliana* [94], a gene involved in Mn^2+^ transport and tolerance [95]. Similarly, *AL.TSO2A* and *AL.TSO2B* in *A. lyrata* are homologous to *AT.TSO2* in *A. thaliana* [94], a gene functionally related to ribonucleotide reductase [96]. On the other hand, diversification in domains may indicate a different function of the original gene. For instance, the *At_RPP1* homolog *At_RPP1^Nd^* (Nd accession) recognises a single allele of *Avr* gene *ATR1^NdWsB^*, while *At_RPP1^WsB^* (WsB accession) also detects *ATR1^NdWsB^* plus three additional alleles with divergent sequences to confer resistance against downey mildew [97].

RGA domains have also been reported to be prone to alteration, such as truncation or even loss of function, as they respond to selection pressure (e.g., presence of virulent pathogens) [98,99]. Truncated *R* genes encoding two-part proteins, such as CN, TN and NL, are evolutionary gene reservoirs and they readily allow for the formation of new genes through duplications, translocation and fusions [100,101,102]. In an RGA, added LRR domains can indicate pathogen specificity. For instance, the LRR domain in *At_RPP1* directly interacts with *Avr ATR1* [103], much like the *L6* recognition of *AvrL567* and the *L11* recognition of *AvrL11* [104,105]. The LRR domain is also important for gene/protein stability [106]. Solo RGA domains could also confer resistance, as reports showed that the overexpression of NBS domains in a potato *R* gene *Rx* (CNL) resulted in an HR [107]. However, the case is different to the CC domain overexpression in *At_RPS5*, as it did not yield a hypersensitive response, but when both CC and NBS were overexpressed, it resulted in a hypersensitive response [108].

In gene clustering, *C. sativa* contained the highest total number of CDRHs clusters due to its higher number of chromosomes, 20, compared to 8 chromosomes of *A. alpina* and *C. hirsuta*. The RGA clusters are more prone to evolutionary processes, such as sequence exchanges, insertion or duplication, followed by neofunctionalisation [109,110,111,112]. The NLRs in a gene cluster can undergo mono or polymerisation, which results in massive expansions of pathogen recognition [111]. For instance, an NLR cluster with eight members contained two functionally characterised *R* genes, *At*_*RPP4* and *At*_*RPP5*, recognizing the *Avr* genes *ATR4* and *ATR5* in the downey mildew resistance response, respectively [113]. Furthermore, it has been shown that RLPs in a gene cluster are most likely pathogen responsive [114]. Two cloned RLP genes, *At_RLP30* and *At_RLP32*, which are involved in bacterial leaf spot resistance, form a gene cluster on At03 in *A. thaliana* [56,115,116], while a gene cluster on A10 in *B. napus* consists of *LepR3/Rlm2*, two alleles of a cloned RLP gene that confers blackleg resistance [117,118] and a homolog of *At_PBS1* [56]. On the other hand, 16 RLK clusters associated with disease resistance were found in *A. thaliana* and *Brassica* crops [56]. Heterogeneous gene clusters with members having RGA domains and including secreted peptides associated to blackleg and clubroot were also observed in *B. napus* [119]. Thus, the CDRHs obtained here, especially those that were clustered, are putative *R* genes that may confer disease resistance.

## 4. Materials and Methods

### 4.1. Mining the Protein Sequences of the Cloned Genes

In total, 49 cloned *R* genes identified in *Brassica* crop species and *A. thaliana* that confer resistance against fungal and bacterial diseases that affect Brassicaceae species (Table 2) were selected based on the following criteria set in a previous study [56]: (1) the *R* gene pairs to an effector or *Avr* gene in a gene-for-gene resistance or (2) confers resistance in the form of a hypersensitive response (usually observed early stage), indicating its involvement in a gene-for-gene interaction or (3) acts as a helper or accessory gene pairing to the existing *R–Avr* interaction. The protein sequences of the 49 cloned *R* genes were retrieved from the UniProtKb (https://www.uniprot.org/uniprot/, verified and accessed on 8 August 2022) [120] or NCBI (https://www.ncbi.nlm.nih.gov/, verified and accessed on 8 August 2022) website.

### 4.2. Mining of Resistance Gene Analogs

The list of predicted RGAs and their subclasses (CN, CNL, NBS, NL, TNL, TX, CNL, TN, Other-NLR, Other-RLK, LRR-RLK, Lysm-RLK, LRR-RLP and Lysm-RLP) derived from the RGAugury pipeline [174] in *A. alpina*, *A. halleri*, *A. lyrata*, *B. vulgaris*, *B. stricta*, *C. sativa*, *C. grandiflora*, *C. rubella*, *C. hirsuta*, *E. salsugineum*, *L. alabamica*, *L. meyenii*, *R. raphanistrum*, *R. sativus*, *S. irio*, *S. parvula* and *T. arvense* genomes were taken from a previous study [51], available at https://research-repository.uwa.edu.au/en/datasets/brassicaceae-rga-candidate-protein-sequences, accessed on 23 November 2020. The RGAugury pipeline was also used in this study to perform in silico prediction of RGAs and their subclasses in the genomes of *B. cretica* [175], *C. bursa-pastoris* [176] and *S. alba* [177].

### 4.3. Identification of Homologs

The RGAs from the 20 Brassicaceae genomes and the 49 cloned *R* genes were aligned using Protein Basic Local Alignment Search Tool (BLASTp) [178]. From the BLASTp results, the criteria of the previous studies in identifying homologous genes in plants were applied by removing hits with greater than E-45 [56,179,180,181] and less than 148 amino acid or aa (coverage) [56] from further analyses. We applied an additional criterion by removing any BLASTp results lower than 60% similarity from further analyses as the homology search was conducted between crop *R* genes and several wild species. Further classification of RGAs was undertaken, according to whether they had the similar resistance domain to their homologous cloned *R* gene counterpart or whether it was different [56].

### 4.4. Gene Cluster Analysis

Among the 20 Brassicaceae species used in this study, only three genomes, *A. alpina* [182], *C. hirsuta* [11] and *C. sativa* [183], were used for gene cluster analysis, due to the accessibility of their pseudo-chromosomes (assigned chromosomes), from which gene clusters were derived. Two types of gene clusters were then identified, with the first defined as a homogenous RGA cluster (having at least 2–8 RGAs of the same class either NLR, RLK or RLP) situated within a 200 kb region on the same chromosome [184,185]. The second was defined as a heterogeneous cluster, containing different classes of RGAs [184,185].

## 5. Conclusions

CWRs with exotic genetic libraries provide rare RGAs, which could be a GMO alternative in improving disease resistance in Brassicaceae crops. This study suggests several domesticated and wild species could be a potential *R* gene source for a particular disease resistance. Based on their CDRHs having RGA domains, *A. alpina* and *B. stricta*, *C. hirsuta* and *C. bursa-pastoris* and *C. sativa* are good sources of resistance against white rust, black rot and Sclerotinia stem rot, respectively. Though the challenge remains in the gene transfer, several methodologies, such as bridging crosses, chromosome doubling after hybrid crossing and somatic hybridization, have found success in Brassicaceae crop breeding. Several CDRHs have also been found in less-explored disease resistance, such as Alternaria black spot, bacterial leaf spot, black rot, grey mould and powdery mildew in Brassicaceae crops, and the RGAs obtained are a valuable starting reference for future studies. Lastly, the current findings of CDRHs in crops *C. sativa*, *R. sativus* and *S. alba* and the 17 wild Brassicaceae species and the previous findings of CDRHs in *A. thaliana* and *Brassica* crops [56] provide an opportunity to study the evolutionary differences in 49 cloned *R* genes (reference in this study) and their homologs throughout the Brassicaceae family.

## Figures and Tables

**Table 1:** Cloned genes with resistance against Brassicaceae diseases and their corresponding homologs (similar by sequence identity) along the homolog types across the 20 studied genomes.

Cloned Gene (RGA Subclass)	Same RGA Domain	Different RGA Domain (Total)	Total
*At_ADR1* (NL)	33 NL	5 CNL, 6 NBS, 1 TNL (12)	45
*At_BAK1* (LRR-RLK)	117 LRR-RLK	2 Other-RLK (2)	119
*At_FLS2* (LRR-RLK)	24 LRR-RLK	0	24
*At_NDR1* (TM)	0	0	0
*At_NRG1a* (RNL)	0	31 CNL, 28 NL, 1 LRR-RLP, 3 CN, 3 NBS (66)	66
*At_NRG1b* (RNL)	0	31 CNL, 26 NL, 1 LRR-RLP, 3 CN, 3 NBS (64)	64
*At_PBS1* (Other-RLK)	20 Other-RLK	0	20
*At_RAC1* (TNL)	48 TNL	10 NL, 3 NBS, 13 TN, 6 TX, 1 Other-NLR (33)	81
*At_RFO1* (Other-RLK)	119 Other-RLK	0	119
*At_RFO2* (LRR-RLP)	31 LRR-RLP	28 LRR-RLK (28)	59
*At_RFO3* (Other-RLK)	50 Other-RLK	0	50
*At_RIN4* (CC)	0	0	0
*At_RLM1a* (TNL)	61 TNL	5 NBS, 12 NL, 9 Other-NLR, 16 TN, 38 TX (80)	141
*At_RLM1b* (TNL)	81 TNL	4 NBS, 23 NL, 8 Other-NLR, 16 TN, 31 TX, 1 LRR-RLP (83)	164
*At_RLM3* (TN)	5 TN	3 NL, 2 NBS, 1 Other-NLR, 7 TNL, 4 TX (17)	22
*At_RLP1* (LRR-RLP)	36 LRR-RLP	0	36
*At_RLP23* (LRR-RLP)	117 LRR-RLP	0	117
*At_RLP30* (LRR-RLP)	47 LRR-RLP	0	47
*At_RLP32* (LRR-RLP)	159 LRR-RLP	1 LRR-RLK (1)	160
*At_RLP42* (LRR-RLP)	112 LRR-RLP	0	112
*At_RPM1* (NL)	14 NL	1 LRR-RLP, 1 NBS (2)	16
*At_RPP1* (TNL)	26 TNL	1 CNL, 22 Other-NLR, 2 NBS, 6 NL, 15 TN, 30 TX (76)	102
*At_RPP13* (CNL)	14 CNL	4 NBS, 1 CN, 16 NL (21)	35
*At_RPP2a* (TNL)	56 TNL	19 NL, 9 Other-NLR, 7 TN, 7 TX (42)	98
*At_RPP2b* (TNL)	20 TNL	1 CNL, 2 NBS, 3 NL, 4 Other-NLR (10)	30
*At_RPP39* (CNL)	71 CNL	11 CN, 3 NBS, 26 NL, 3 LRR-RLP (43)	114
*At_RPP4* (TNL)	8 TNL	3 NL, 2 Other-NLR, 5 TN, 5 TX (15)	23
*At_RPP5* (TNL)	8 TNL	2 NL, 3 Other-NLR, 6 TN, 11 TX (22)	30
*At_RPP7* (NL)	56 NL	1 CN, 12 CNL, 1 LRR-RLP, 10 NBS (24)	80
*At_RPP8* (CNL)	80 CNL	12 CN, 6 NBS, 24 NL (42)	122
*At_RPS2* (NL)	6 NL	18 CNL, 3 NBS (21)	27
*At_RPS4* (TNL)	32 TNL	1 NBS, 6 NL, 7 Other-NLR (14)	46
*At_RPS5* (TNL)	0	58 CNL, 6 CN, 7 NBS, 22 NL (93)	93
*At_Rpw8.1* (RNL)	0	0	0
*At_Rpw8.2* (RNL)	0	0	0
*At_RRS1* (TNL)	26 TNL	0 (15)	41
*At_SOBIR1* (LRR-RLK)	26 LRR-RLK	1 Other-RLK (1)	27
*At_WRR12* (TNL)	29 TNL	5 NL, 2 TX, 4 LRR-RLP (11)	40
*At_WRR4a* (TNL)	37 TNL	4 NL, 4 Other-NLR, 6 TN, 33 TX (47)	84
*At_WRR4b* (TNL)	51 TNL	2 LRR-RLP, 5 NL, 6 Other-NLR, 17 TN, 38 TX (68)	119
*At_WRR8* (TNL)	56 TNL	12 TN, 4 NBS, 11 NL, 2 Other-NLR, 6 TX (35)	91
*At_WRR9* (NL)	6 NL	1 NBS, 1 Other-NLR, 9 TN, 35 TNL, 16 TX (62)	68
*Bju_WRR1* (CNL)	39 CNL	10 NL, 9 CN, 3 NBS (22)	61
*Bna_LepR3/Rlm2* (LRR-RLP)	97 LRR-RLP	0	97
*Bna_MAPk* (Other-RLK)	8 Other-RLK	0	8
*Bna_Rlm9/4/7* (Other-RLK)	101 Other-RLK	0	101
*Bol_FocBo1* (TNL)	23 TNL	3 Other-NLR, 7 TN, 14 TX, 8 NL (32)	55
*Bra_cRa/cRb* (TNL)	14 TNL	1 Other-NLR, 5 TN, 1NBS, 7 TX (14)	28
*Bra_Crr1a* (TNL)	28 TNL	7 NL, 6 Other-NLR, 28 TN, 19 TX, 2 NBS (62)	90
Total	1992	1181	3172

*At* = *Arabidopsis thaliana*, *Bju* = *Brassica juncea*, *Bol* = *Brassica oleracea*, *Bra* = *Brassica rapa*, *Bna* = *Brassica napus*, Resistance-gene analogs (RGA) domain in comparison to the cloned gene. CN = coiled-coil (CC)-nucleotide-binding site (NBS), CNL = CC-NBS-leucine rice repeats (LRR), NL = NBS-LRR, TN = Toll/Interleukin-1 receptor (TIR)-LRR, TNL = Toll/Interleukin-1 receptor (TIR)-NBS-LRR, TX = Toll/Interleukin-1 receptor (TIR) with other domains, Other-NLR = NBS-LRR with other domains, RNL = resistance to powdery mildew 8 (Rpw8)-NBS-LRR, LRR-RLK = LRR-receptor-like kinase proteins (RLK), Other-RLK= RLK with other domains, LRR-RLP = LRR-receptor-like proteins, TM = transmembrane.

**Table 2:** The 49 cloned *R* genes from *Arabidopsis thaliana* (At), *Brassica juncea* (Bju), *Brassica napus* (Bna) and *Brassica rapa* (Bra) used for homology searches.

Gene (Accession ID/Reference)	Pathogen
*At_ADR1* (Q9FW44 ^U^) [121,122,123]	*Hyaloperonospora arabidopsidis* ^F^, *Erysiphe cichoracearum* ^F^ and *Pseudomonas syringae* ^B^
*At_BAK1* (Q94F62 ^U^) and *At_SOBIR1* (Q9SKB2 ^U^) [124,125] and *At_RLP30* (Q9MA83 ^U^) [115,126]	*P. syringae* and *Sclerotinia sclerotiorum* ^F^
*At_RPS2* (Q42484 ^U^) [127], *At_RPS4* (Q9XGM3 ^U^) [128] and *At_RPS5* (O64973 ^U^) [129], *At_FLS2* (Q9FL28 ^U^) [130,131], *At_NDR1* (O48915 ^U^) [132], *At_PBS1* (Q9FE20 ^U^) [133], *At_RLP32* (Q9M9X0 ^U^) [116], *At_RPM1* (Q39214 ^U^) [134,135], *At_RIN4* (Q8GYN5 ^U^) [136,137,138,139,140] and *At_RRS1* (P0DKH5 ^U^) [141,142]	*P. syringae*
*At_NGR1a* (Q9FKZ1 ^U^) and *At_NGR1b* (Q9FKZ0 ^U^) [122,123]	*Albugo candida*^F^, *H. arabidopsidis*, and *P. syringae*
*At_RFO1* (Q8RY17 ^U^) [143], *At_RFO2* (Q9SHI4 ^U^) [144] and *At_RFO3* (Q9LW83 ^U^) [145]	*Fusarium oxysporum matthioli* ^F^
*At_RLM1a* (F4I594 ^U^) and *At_RLM1b* (Q9CAK1 ^U^) [146], *Bna_MPK9* (A0A078IFE9 ^U^) [147], *Bna_LepR3/Rlm2* (I7C3X3 ^U^/A0A0B5L618 ^U^) [118,148], *Bna_Rlm9/4/7* (CDX67982.1 ^N^) [149,150]	*Leptosphaeria maculans* ^F^
*At_RLM3* (Q9FT77 ^U^) [151]	*L. maculans*, *Botrytis cinerea* ^F^, *Alternaria brassicicola* ^F^ and *A. brassicae* ^F^
*At_RLP1* (Q9LNV9 ^U^) [152,153]	*Xanthomonas* spp. ^B^
*At_RLP23* (O48849 ^U^) [125,154]	*S. sclerotiorum*
*At_RLP42* (Q9LJS0 ^U^) [155]	*B. cinerea* and *H. arabidopsidis*
*At_RPP1* (F4J339 ^U^) [156], *At_RPP2a* (F4JT78 ^U^) and *At_RPP2b* (F4JT80 ^U^) [157], *At_RPP4* (F4JNA9 ^U^) [158], *At_RPP5* (F4JNB7 ^U^) [159], *At_RPP7* (Q8W3K0 ^U^) [160,161], *At_RPP8* (Q8W4J9 ^U^) [162], *At_RPP13* (Q9M667 ^U^) [163] and *At_RPP39* (H9BPR9 ^U^) [164]	*H. arabidopsidis*
*At_Rpw8.1* (Q9C5Z7 ^U^) and *At_Rpw8.2* (Q9C5Z6 ^U^) [165]	*E. cichoracearum*
*At_RAC1* (Q6QX58 ^U^) [166], *At_WRR4a* (Q9C7X0 ^U^) and *At_WRR4b* (MK034466 ^N^) [167], *At_WRR8* (MK034463 ^N^), *At_WRR9* (MK034464 ^N^), *At_WRR12* (MK034462 ^N^) [168] and *Bju_WRR1* (A0A0B5L618 ^U^) [169]	*A. candida*
*Bra_cRa/cRb* (M5A8J3 ^U^) [170,171] and *Bra_Crr1a* (AB605024.1 ^N^) [172]	*Plasmodiophora brassicae* ^F^
*Bol_FocBo1* (BAQ21734.1 ^N^) [173]	*F. oxysporum* f. sp. *Conglutinans* ^F^

^F^ = fungus, ^B^ = bacteria, RGA = resistance-gene analog, ^U^ = https://www.uniprot.org/uniprot/, accessed on 10 October 2020) website, ^N^ = https://www.ncbi.nlm.nih.gov/ (accessed on 10 October 2020).

## Data Availability

The data used in this research are publicly available. The protein sequences of each cloned gene can be found at https://www.uniprot.org/uniprot/ (accessed on 10 October 2020) and https://www.ncbi.nlm.nih.gov/ (accessed on 10 October 2020). The data (results) presented in this research are available in the Supplementary Materials.

## Supplementary Materials

The following supporting information can be downloaded at: https://www.mdpi.com/article/10.3390/plants11223010/s1, Table S1: List of resistance-gene analogs (RGAs) in *Brassica cretica*, *Capsella bursa-pastoris* and *Sinapis alba* using RGAugury pipeline. Table S2: List of resistance-gene analogs (RGAs) homologous to cloned disease resistance genes (*R* genes) and the E-value and similarity basis.

## References

[1] Al-Shehbaz I.A. (1985). The Genera of Brassiceae (Cruciferae; Brassicaceae) in the Southeastern United States. J. Arnold Arbor..

[2] Tamokou J.D.D., Mbaveng A.T., Kuete V., Kuete V. (2017). Chapter 8-Antimicrobial Activities of African Medicinal Spices and Vegetables. Medicinal Spices and Vegetables from Africa.

[3] Warwick S.I., Mummenhoff K., Sauder C.A., Koch M.A., Al-Shehbaz I.A. (2010). Closing the gaps: Phylogenetic relationships in the Brassicaceae based on DNA sequence data of nuclear ribosomal ITS region. Plant Syst. Evol..

[4] Koornneef M., Meinke D. (2010). The development of *Arabidopsis* as a model plant. Plant J..

[5] Palmer C.E., Warwick S., Keller W. (2001). Brassicaceae (Cruciferae) family, plant biotechnology, and phytoremediation. Int. J. Phytoremed..

[6] Wötzel S., Andrello M., Albani M.C., Koch M.A., Coupland G., Gugerli F. (2022). *Arabis alpina*: A perennial model plant for ecological genomics and life-history evolution. Mol. Ecol. Resour..

[7] Nielsen N.J., Nielsen J., Staerk D. (2010). New resistance-correlated saponins from the insect-resistant crucifer *Barbarea vulgaris*. J. Agric. Food Chem..

[8] Brukhin V., Osadtchiy J.V., Florez-Rueda A.M., Smetanin D., Bakin E., Nobre M.S., Grossniklaus U. (2019). The *Boechera* Genus as a Resource for Apomixis Research. Front. Plant Sci..

[9] Nagaharu U. (1935). Genome analysis in *Brassica* with special reference to the experimental formation of *B. napus* and peculiar mode of fertilization. Jpn. J. Bot..

[10] Bansal S., Durrett T.P. (2016). *Camelina sativa*: An ideal platform for the metabolic engineering and field production of industrial lipids. Biochimie.

[11] Gan X., Hay A., Kwantes M., Haberer G., Hallab A., Ioio R.D., Hofhuis H., Pieper B., Cartolano M., Neumann U. (2016). The *Cardamine hirsuta* genome offers insight into the evolution of morphological diversity. Nat. Plants.

[12] Wu H.J., Zhang Z., Wang J.Y., Oh D.-H., Dassanayake M., Liu B., Huang Q., Sun H.X., Xia R., Wu Y. (2012). Insights into salt tolerance from the genome of *Thellungiella salsuginea*. Proc. Natl. Acad. Sci. USA.

[13] Lee J.Y., Mummenhoff K., Bowman J.L. (2002). Allopolyploidization and evolution of species with reduced floral structures in Lepidium L. (Brassicaceae). Proc. Natl. Acad. Sci. USA.

[14] Moser B.R., Evangelista R.L., Jham G. (2015). Fuel properties of *Brassica juncea* oil methyl esters blended with ultra-low sulfur diesel fuel. Renew. Energy.

[15] Wilkes M.A., Takei I., Caldwell R.A., Trethowan R.M. (2013). The effect of genotype and environment on biodiesel quality prepared from Indian mustard (*Brassica juncea*) grown in Australia. Ind. Crops Prod..

[16] Rahman M., Khatun A., Liu L., Barkla B.J. (2018). Brassicaceae Mustards: Traditional and Agronomic Uses in Australia and New Zealand. Molecules.

[17] Austin D. (2003). Dye Plants and Dyeing, Revised edition: Daniel F. Austin, Book Review Editor. Econ. Bot..

[18] Hamburger M. (2002). Isatis tinctoria–From the rediscovery of an ancient medicinal plant towards a novel anti-inflammatory phytopharmaceutical. Phytochem. Rev..

[19] Denisow B. (2008). Flowering and pollen production of several f. brassicaceae ornamentals. J. Apic. Sci..

[20] Raza A., Hafeez M.B., Zahra N., Shaukat K., Umbreen S., Tabassum J., Charagh S., Khan R.S., Hasanuzzaman M., Hasanuzzaman M. (2020). The Plant Family Brassicaceae: Introduction, Biology, And Importance. The Plant Family Brassicaceae.

[21] Barbetti M.J., Li C.X., Banga S.S., Banga S.K., Singh D., Sandhu P.S., Singh R., Liu S.Y., You M.P. (2015). New host resistances in *Brassica napus* and *Brassica juncea* from Australia, China and India: Key to managing Sclerotinia stem rot (*Sclerotinia sclerotiorum*) without fungicides. Crop Prot..

[22] Barbetti M.J., Li C.X., You M.P., Singh D., Agnihotri A., Banga S.K., Sandhu P.S., Singh R., Banga S.S. (2016). Valuable New Leaf or Inflorescence Resistances Ensure Improved Management of White Rust (*Albugo candida*) in Mustard (*Brassica juncea*) Crops. J. Phytopathol..

[23] Bhattacharya I., Dutta S., Mondal S., Mondal B. (2014). Clubroot disease on *Brassica* crops in India. Can. J. Plant Pathol..

[24] Chattopadhyay C., Kolte S.J., Waliyar F. (2015). Diseases of Edible Oilseed Crops.

[25] Mani A., Dutta P., Chatterjee S. (2020). Diseases in *Brassica* vegetable crops and their Integrated Disease Management (IDM). Agric. Food E-Newsl..

[26] Li C.X., Sivasithamparam K., Walton G., Salisbury P., Burton W., Banga S.S., Banga S., Chattopadhyay C., Kumar A., Singh R. (2007). Expression and relationships of resistance to white rust (*Albugo candida*) at cotyledonary, seedling, and flowering stages in *Brassica juncea* germplasm from Australia, China, and India. Aust. J. Agric. Res..

[27] Balesdent M.H., Barbetti M.J., Li H., Sivasithamparam K., Gout L., Rouxel T. (2005). Analysis of *Leptosphaeria maculans* Race Structure in a Worldwide Collection of Isolates. Phytopathology.

[28] Marcroft S.J., Elliott V.L., Cozijnsen A.J., Salisbury P.A., Howlett B.J., Van de Wouw A.P. (2012). Identifying resistance genes to in Australian cultivars based on reactions to isolates with known avirulence genotypes. Crop Pasture Sci..

[29] Jo S.J., Jang K.S., Choi Y.H., Kim J.C., Choi G.J. (2011). Development of convenient screening method for resistant radish to *Plasmodiophora brassicae*. Res. Plant Dis..

[30] Fredua-Agyeman R., Jiang J., Hwang S.-F., Strelkov S.E. (2020). QTL Mapping and Inheritance of Clubroot Resistance Genes Derived From *Brassica rapa* subsp. *rapifera* (ECD 02) Reveals Resistance Loci and Distorted Segregation Ratios in Two F2 Populations of Different Crosses. Front. Plant Sci..

[31] Gan C., Yan C., Pang W., Cui L., Fu P., Yu X., Qiu Z., Zhu M., Piao Z., Deng X. (2022). Identification of Novel Locus *RsCr6* Related to Clubroot Resistance in Radish (*Raphanus sativus* L.). Front. Plant Sci..

[32] Liu Y., Xu A., Liang F., Yao X., Wang Y., Liu X., Zhang Y., Dalelhan J., Zhang B., Qin M. (2018). Screening of clubroot-resistant varieties and transfer of clubroot resistance genes to *Brassica napus* using distant hybridization. Breed Sci..

[33] Atri C., Akhatar J., Gupta M., Gupta N., Goyal A., Rana K., Kaur R., Mittal M., Sharma A., Singh M.P. (2019). Molecular and genetic analysis of defensive responses of *Brassica juncea-B. fruticulosa* introgression lines to Sclerotinia infection. Sci. Rep..

[34] Rana K., Atri C., Akhatar J., Kaur R., Goyal A., Singh M.P., Kumar N., Sharma A., Sandhu P.S., Kaur G. (2019). Detection of First Marker Trait Associations for Resistance Against *Sclerotinia sclerotiorum* in *Brassica juncea–Erucastrum cardaminoides* Introgression Lines. Front. Plant Sci..

[35] Kumari P., Singh K.P., Bisht D., Kumar S. (2020). Somatic hybrids of *Sinapis alba + Brassica juncea*: Study of backcross progenies for morphological variations, chromosome constitution and reaction to *Alternaria brassicae*. Euphytica.

[36] Mei J., Shao C., Yang R., Feng Y., Gao Y., Ding Y., Li J., Qian W. (2020). Introgression and pyramiding of genetic loci from wild *Brassica oleracea* into *B. napus* for improving Sclerotinia resistance of rapeseed. Theor. Appl. Genet..

[37] Garg H., Banga S., Bansal P., Atri C., Banga S.S. (2007). Hybridizing *Brassica rapa* with wild crucifers *Diplotaxis erucoides* and *Brassica maurorum*. Euphytica.

[38] Chen C.Y., Séguin-Swartz G. (1999). Reaction of wild crucifers to *Leptosphaeria maculans*, the causal agent of blackleg of crucifers. Can. J. Plant Pathol..

[39] Gugel R.K., Séguin-Swartz G. Introgression of Blackleg Resistance from Sinapis alba into Brassica napus. Proceedings of the Brassica 97: International Symposium on Brassicas: 10th Crucifer Genetics Workshop.

[40] Li H., Barbetti M.J., Sivasithamparam K. (2005). Hazard from reliance on cruciferous hosts as sources of major gene-based resistance for managing blackleg (*Leptosphaeria maculans*) disease. Field Crops Res..

[41] Mithen R.F., Magrath R. (1992). Glucosinolates and Resistance to *Leptosphaeria maculans* in Wild and Cultivated *Brassica* Species. Plant Breed..

[42] Pedras M.S., Chumala P.B., Suchy M. (2003). Phytoalexins from *Thlaspi arvense*, a wild crucifer resistant to virulent *Leptosphaeria maculans*: Structures, syntheses and antifungal activity. Phytochemistry.

[43] Plümper B. (1995). Somatische und Sexuelle Hybridisierung für den Transfer von Krankheitsresistenzen auf Brassica napus L. Ph.D. Thesis.

[44] Tewari J.P., Bansal V.K., Tewari I., Gómez-Campo C., Stringam G.R., Thiagarajah M.R. (1996). Reactions of some wild and cultivated accessions of *Eruca* against *Leptosphaeria maculans*. Cruciferae Newslett..

[45] Winter H. (2004). Untersuchungen zur Introgression von Resistenzen gegen die Wurzelhals- und Stengelfäule [*Leptosphaeria maculans* (Desm.) Ces. et De Not.] aus Verwandten Arten in den Raps (*Brassica napus* L.). Ph.D. Thesis.

[46] Sekhwal M.K., Li P., Lam I., Wang X., Cloutier S., You F.M. (2015). Disease Resistance Gene Analogs (RGAs) in Plants. Int. J. Mol. Sci..

[47] Jones J.D., Dangl J.L. (2006). The plant immune system. Nature.

[48] Le Roux C., Huet G., Jauneau A., Camborde L., Trémousaygue D., Kraut A., Zhou B., Levaillant M., Adachi H., Yoshioka H. (2015). A receptor pair with an integrated decoy converts pathogen disabling of transcription factors to immunity. Cell.

[49] Ravensdale M., Bernoux M., Ve T., Kobe B., Thrall P.H., Ellis J.G., Dodds P.N. (2012). Intramolecular Interaction Influences Binding of the Flax *L5* and *L6* Resistance Proteins to their *AvrL567* Ligands. PLoS Pathog..

[50] Whitham S., Dinesh-Kumar S.P., Choi D., Hehl R., Corr C., Baker B. (1994). The product of the tobacco mosaic virus resistance gene N: Similarity to toll and the interleukin-1 receptor. Cell.

[51] Tirnaz S., Bayer P., Inturrisi F., Zhang F., Yang H., Dolatabadian A., Neik T., Severn-Ellis A., Patel D., Ibrahim M. (2020). Resistance gene analogs in the Brassicaceae: Identification, characterization, distribution and evolution. Plant Physiol..

[52] Afzal A.J., Wood A.J., Lightfoot D.A. (2008). Plant receptor-like serine threonine kinases: Roles in signaling and plant defense. Mol. Plant Microbe Interact..

[53] Chisholm S.T., Coaker G., Day B., Staskawicz B.J. (2006). Host-microbe interactions: Shaping the evolution of the plant immune response. Cell.

[54] Jeong S., Trotochaud A.E., Clark S.E. (1999). The *Arabidopsis CLAVATA2* gene encodes a receptor-like protein required for the stability of the *CLAVATA1* receptor-like kinase. Plant Cell.

[55] Nadeau J.A., Sack F.D. (2002). Control of stomatal distribution on the *Arabidopsis* leaf surface. Science.

[56] Cantila A.Y., Neik T.X., Tirnaz S., Thomas W.J.W., Bayer P.E., Edwards D., Batley J. (2022). Mining of Cloned Disease Resistance Gene Homologs (CDRHs) in *Brassica* Species and *Arabidopsis thaliana*. Biology.

[57] Wu P., Shao Z.Q., Wu X.Z., Wang Q., Wang B., Chen J.Q., Hang Y.Y., Xue J.Y. (2014). Loss retention and evolution of NBS-encoding genes upon whole genome triplication of *Brassica rapa*. Gene.

[58] Bayer P., Golicz A., Tirnaz S., Chan C.K.K., Edwards D., Batley J. (2019). Variation in abundance of predicted resistance genes in the *Brassica oleracea* pangenome. Plant Biotechnol. J..

[59] Fedoroff N. (2000). Transposons and genome evolution in plants. Proc. Natl. Acad. Sci. USA.

[60] Vicient C.M., Casacuberta J.M. (2017). Impact of transposable elements on polyploid plant genomes. Ann. Bot..

[61] Bayer P.E., Scheben A., Golicz A.A., Yuan Y., Faure S., Lee H., Chawla H.S., Anderson R., Bancroft I., Raman H. (2021). Modelling of gene loss propensity in the pangenomes of three *Brassica* species suggests different mechanisms between polyploids and diploids. Plant Biotechnol. J..

[62] Yaakov B., Meyer K., Ben-David S., Kashkush K. (2013). Copy number variation of transposable elements in *Triticum–Aegilops* genus suggests evolutionary and revolutionary dynamics following allopolyploidization. Plant Cell Rep..

[63] Dolatabadian A., Bayer P.E., Tirnaz S., Hurgobin B., Edwards D., Batley J. (2020). Characterization of disease resistance genes in the *Brassica napus* pangenome reveals significant structural variation. Plant Biotechnol. J..

[64] Jabeen N., Hasanuzzaman M. (2020). Agricultural, Economic and Societal Importance of Brassicaceae Plants. The Plant Family Brassicaceae.

[65] Séguin-Swartz G., Gugel R.K., Strelkov S., Olivier C., Li J.L., Klein-Gebbinck H., Borhan H., Caldwell C., Falk K.C. (2010). Diseases of *Camelina sativa* (false flax). Can. J. Plant Pathol..

[66] Duan Y.D.Y., Wang J.L., Wang H.P., Zhang X., Shen D., Song J.P., Li X. (2015). Genetic analysis on the resistance of different radish germplasm to black rot. J. Plant Genet. Resour..

[67] Zhan Z., Nwafor C.C., Hou Z., Gong J., Zhu B., Jiang Y., Zhou Y., Wu J., Piao Z., Tong Y. (2017). Cytological and morphological analysis of hybrids between *Brassicoraphanus*, and *Brassica napus* for introgression of clubroot resistant trait into *Brassica napus* L. PLoS ONE.

[68] Yang H., Yuan Y., Wei X., Zhang X., Wang H., Song J., Li X. (2021). A New Identification Method Reveals the Resistance of an Extensive-Source Radish Collection to *Plasmodiophora brassicae* Race 4. Agronomy.

[69] Coelho P., Valério L., Monteiro A. (2022). Comparing Cotyledon, Leaf and Root Resistance To Downy Mildew in Radish (*Raphanus sativus* L). Euphytica.

[70] Xu L., Jiang Q.W., Wu J., Wang Y., Gong Y.Q., Wang X.L., Limera C., Liu L.W. (2014). Identification and Molecular Mapping of the *RsDmR* Locus Conferring Resistance to Downy Mildew at Seedling Stage in Radish (*Raphanus sativus* L.). J. Integr. Agric..

[71] Lee O.N., Koo H., Yu J.W., Park H.Y. (2021). Genotyping-by-Sequencing-Based Genome-Wide Association Studies of Fusarium Wilt Resistance in Radishes (*Raphanus sativus* L.). Genes.

[72] Kolte S.J., Bordoloi D.K., Awasthi R.P. The search for resistance to major diseases of rapeseed and mustard in India. Proceedings of the GCIRC 8th International Rapeseed Congress.

[73] Nyalugwe E.P., Barbetti M.J., Jones R.A. (2015). Studies on resistance phenotypes to Turnip mosaic virus in five species of Brassicaceae, and identification of a virus resistance gene in *Brassica juncea*. Eur. J. Plant Pathol..

[74] Scholze P., Krämer R., Ryschka U., Klocke E., Schumann G. (2010). Somatic hybrids of vegetable brassicas as source for new resistances to fungal and virus diseases. Euphytica.

[75] Khangura R., Aberra M. (2006). Strains of *Leptosphaeria maculans* with the Capacity to Cause Crown Canker on *Brassica carinata* are Present in Western Australia. Plant Dis..

[76] Mamula D., Juretic N., Horvath J. (1997). Susceptibility of host plants to belladonna mottle and turnip yellow mosaic tymoviruses: Multiplication and distribution. Acta Phytopathol. Entomol. Hung..

[77] Kumari P., Singh K.P. (2019). Characterization of Stable Somatic Hybrids of *Sinapis alba* and *Brassica juncea* for Alternaria blight, *Sclerotinia sclerotiurum* Resistance and Heat Tolerance. Indian Res. J. Ext. Educ..

[78] Li A., Wei C., Jiang J., Zhang Y., Snowdon R.J., Wang Y. (2009). Phenotypic variation in progenies from somatic hybrids between *Brassica napus* and *Sinapis alba*. Euphytica.

[79] Westman A.L., Dickson M. (1998). Disease reaction to *Alternaria brassicicola* and *Xanthomonas campestris* pv. *campestris* in *Brassica nigra* and other weedy crucifers. Crucif. Newslett..

[80] Happstadius I., Ljungberg A., Kristiansson B., Dixelius C. (2003). Identification of *Brassica oleracea* germplasm with improved resistance to Verticillium wilt. Plant Breed..

[81] Siemens J. (2002). Interspecific Hybridisation between Wild Relatives and *Brassica napus* to Introduce New Resistance Traits into the Oilseed Rape Gene Pool. Czech J. Genet. Plant Breed..

[82] Chen H.F., Wang H., Li Z.Y. (2007). Production and genetic analysis of partial hybrids in intertribal crosses between *Brassica* species (*B. rapa*, *B. napus*) and *Capsella bursa-pastoris*. Plant Cell Rep..

[83] Tewari J.P., McGregor D. (1991). Current understanding of resistance to *Alternaria brassicae* in crucifers. Rapeseeds in a Changing World, Proceedings of the 8th International Rapeseed Congress, Saskatoon, SK, Canada, 9 July 1991.

[84] Crute I., Gray A., Crisp P., Buczacki S. (1980). Variation in *Plasmodiophora brassicae* and resistance to clubroot disease in brassicas and allied crops-a critical review. Plant Breed. Abstr..

[85] Mohammed A.E., You M.P., Al-lami H.F.D., Barbetti M.J. (2018). Pathotypes and phylogenetic variation determine downy mildew epidemics in *Brassica* spp. in Australia. Plant Pathol..

[86] Uloth M.B., You M.P., Finnegan P.M., Banga S.S., Banga S.K., Sandhu P.S., Yi H., Salisbury P.A., Barbetti M.J. (2013). New sources of resistance to *Sclerotinia sclerotiorum* for crucifer crops. Field Crops Res..

[87] Schmidt R., Bancroft I. (2010). Genetics and Genomics of the Brassicaceae.

[88] Bowers J.E., Chapman B.A., Rong J., Paterson A.H. (2003). Unravelling angiosperm genome evolution by phylogenetic analysis of chromosomal duplication events. Nature.

[89] Song X., Wei Y., Xiao D., Gong K., Sun P., Ren Y., Yuan J., Wu T., Yang Q., Li X. (2021). *Brassica carinata* genome characterization clarifies U’s triangle model of evolution and polyploidy in *Brassica*. Plant Physiol..

[90] Beilstein M.A., Nagalingum N.S., Clements M.D., Manchester S.R., Mathews S. (2010). Dated molecular phylogenies indicate a Miocene origin for *Arabidopsis thaliana*. Proc. Natl. Acad. Sci. USA.

[91] Lysak M.A., Koch M.A., Pecinka A., Schubert I. (2005). Chromosome triplication found across the tribe Brassiceae. Genome Res..

[92] Wu Q., Han T.-S., Chen X., Chen J.F., Zou Y.P., Li Z.W., Xu Y.C., Guo Y.L. (2017). Long-term balancing selection contributes to adaptation in *Arabidopsis* and its relatives. Genome Biol..

[93] Charlesworth D. (2006). Balancing Selection and Its Effects on Sequences in Nearby Genome Regions. PLoS Genet..

[94] Das M., Haberer G., Panda A., Das Laha S., Ghosh T.C., Schäffner A.R. (2016). Expression Pattern Similarities Support the Prediction of Orthologs Retaining Common Functions after Gene Duplication Events. Plant Physiol..

[95] Gustin J.L., Zanis M.J., Salt D.E. (2011). Salt, Structure and evolution of the plant cation diffusion facilitator family of ion transporters. BMC Evol. Biol..

[96] Wang C., Liu Z. (2006). Arabidopsis ribonucleotide reductases are critical for cell cycle progression, DNA damage repair, and plant development. Plant Cell.

[97] Rehmany A.P., Gordon A., Rose L.E., Allen R.L., Armstrong M.R., Whisson S.C., Kamoun S., Tyler B.M., Birch P.R.J., Beynon J.L. (2005). Differential Recognition of Highly Divergent Downy Mildew Avirulence Gene Alleles by *RPP1* Resistance Genes from Two *Arabidopsis* Lines. Plant Cell.

[98] Faulkner C., Robatzek S. (2012). Plants and pathogens: Putting infection strategies and defence mechanisms on the map. Curr. Opin. Plant Biol..

[99] Nepal M.P., Benson B.V. (2015). CNL disease resistance genes in soybean and their evolutionary divergence. Evol. Bioinform..

[100] Joshi R., Nayak S. (2013). Perspectives of genomic diversification and molecular recombination towards *R*-gene evolution in plants. Physiol. Mol. Biol. Plants.

[101] Yue J.X., Meyers B.C., Chen J.Q., Tian D., Yang S. (2012). Tracing the origin and evolutionary history of plant nucleotide-binding site–leucine-rich repeat NBS-LRR, genes. New Phytol..

[102] Jacob F., Vernaldi S., Maekawa T. (2013). Evolution and conservation of plant NLR functions. Front. Immunol..

[103] Steinbrenner A.D., Goritschnig S., Staskawicz B.J. (2015). Recognition and Activation Domains Contribute to Allele-Specific Responses of an Arabidopsis NLR Receptor to an Oomycete Effector Protein. PLoS Pathog..

[104] Ellis J., Dodds P., Pryor T. (2000). Structure, function and evolution of plant disease resistance genes. Curr. Opin. Plant Biol..

[105] Dodds P.N., Lawrence G.J., Catanzariti A.-M., Teh T., Wang C.-I., Ayliffe M.A., Kobe B., Ellis J.G. (2006). Direct protein interaction underlies gene-for-gene specificity and coevolution of the flax resistance genes and flax rust avirulence genes. Proc. Natl. Acad. Sci. USA.

[106] Zhang Y., Dorey S., Swiderski M., Jones J.D. (2004). Expression of *RPS4* in tobacco induces an *AvrRps4*-independent HR that requires *EDS1*, *SGT1* and *HSP90*. Plant J..

[107] Rairdan G.J., Collier S.M., Sacco M.A., Baldwin T.T., Boettrich T., Moffett P. (2008). The coiled-coil and nucleotide binding domains of the potato *Rx* disease resistance protein function in pathogen recognition and signaling. Plant Cell.

[108] Ade J., DeYoung B.J., Golstein C., Innes R.W. (2007). Indirect activation of a plant nucleotide binding site–leucine-rich repeat protein by a bacterial protease. Proc. Natl. Acad. Sci. USA.

[109] Michelmore R.W., Meyers B.C. (1998). Clusters of resistance genes in plants evolve by divergent selection and a birth-and-death process. Genome Res..

[110] Nützmann H.-W., Scazzocchio C., Osbourn A. (2018). Metabolic gene clusters in eukaryotes. Annu. Rev. Genet..

[111] van Wersch S., Li X. (2019). Stronger When Together: Clustering of Plant NLR Disease resistance Genes. Trends Plant Sci..

[112] Seah S., Telleen A.C., Williamson V.M. (2007). Introgressed and endogenous *Mi-1* gene clusters in tomato differ by complex rearrangements in flanking sequences and show sequence exchange and diversifying selection among homologues. Theor. Appl. Genet..

[113] Meyers B., Kozik A., Griego A., Kuang H., Michelmore R. (2003). Genome-wide analysis of NBS-LRR-encoding genes in *Arabidopsis*. Plant Cell.

[114] Steidele C.E., Stam R. (2021). Multi-omics approach highlights differences between RLP classes in *Arabidopsis thaliana*. BMC Genom..

[115] Wang G., Ellendorff U., Kemp B., Mansfield J.W., Forsyth A., Mitchell K., Bastas K., Liu C.-M., Woods-Tör A., Zipfel C. (2008). A Genome-Wide Functional Investigation into the Roles of Receptor-Like Proteins in *Arabidopsis*. Plant Physiol..

[116] Fan L., Fröhlich K., Melzer E., Albert I., Pruitt R.N., Zhang L., Albert M., Kim S.-T., Chae E., Weigel D. (2022). Genotyping-by-sequencing-based identification of *Arabidopsis* pattern recognition receptor *RLP32* recognizing proteobacterial translation initiation factor IF1. Nat. Commun..

[117] Larkan N., Lydiate D., Yu F., Rimmer S., Borhan H. (2014). Co-localisation of the blackleg resistance genes *Rlm2* and *LepR3* on *Brassica napus* chromosome A10. BMC Plant Biol..

[118] Larkan N.J., Ma L., Borhan M.H. (2015). The *Brassica napus* receptor-like protein *RLM2* is encoded by a second allele of the *LepR3/Rlm2* blackleg resistance locus. Plant Biotechnol. J..

[119] Stotz H.U., Harvey P.J., Haddadi P., Mashanova A., Kukol A., Larkan N.J., Borhan M.H., Fitt B.D.L. (2018). Genomic evidence for genes encoding leucine-rich repeat receptors linked to resistance against the eukaryotic extra- and intracellular *Brassica napus* pathogens *Leptosphaeria maculans* and *Plasmodiophora brassicae*. PLoS ONE.

[120] The UniProt (2021). UniProt: The universal protein knowledgebase in 2021. Nucleic Acids Res..

[121] Grant J.J., Chini A., Basu D., Loake G.J. (2003). Targeted Activation Tagging of the *Arabidopsis* NBS-LRR gene, *ADR1*, Conveys Resistance to Virulent Pathogens. Mol. Plant Microbe Interact..

[122] Castel B., Ngou P.M., Cevik V., Redkar A., Kim D.S., Yang Y., Ding P., Jones J.D.G. (2019). Diverse NLR immune receptors activate defence via the *RPW8*-NLR *NRG1*. New Phytol..

[123] Saile S.C., Jacob P., Castel B., Jubic L.M., Salas-Gonzáles I., Bäcker M., Jones J.D.G., Dangl J.L., El Kasmi F. (2020). Two unequally redundant "helper" immune receptor families mediate *Arabidopsis thaliana* intracellular "sensor" immune receptor functions. PLoS Biol..

[124] Gao M., Wang X., Wang D., Xu F., Ding X., Zhang Z., Bi D., Cheng Y.T., Chen S., Li X. (2009). Regulation of cell death and innate immunity by two receptor-like kinases in *Arabidopsis*. Cell Host Microbe.

[125] Albert I., Böhm H., Albert M., Feiler C.E., Imkampe J., Wallmeroth N., Brancato C., Raaymakers T.M., Oome S., Zhang H. (2015). An *RLP23–SOBIR1–BAK1* complex mediates NLP-triggered immunity. Nat. Plants.

[126] Zhang W., Fraiture M., Kolb D., Löffelhardt B., Desaki Y., Boutrot F.F.G., Tör M., Zipfel C., Gust A.A., Brunner F. (2013). *Arabidopsis RECEPTOR-LIKE PROTEIN30* and Receptor-Like Kinase *SUPPRESSOR OF BIR1-1/EVERSHED* Mediate Innate Immunity to Necrotrophic Fungi. Plant Cell.

[127] Bent A.F., Kunkel B.N., Dahlbeck D., Brown K.L., Schmidt R., Giraudat J., Leung J., Staskawicz B.J. (1994). *RPS2* of *Arabidopsis thaliana*: A leucine-rich repeat class of plant disease resistance genes. Science.

[128] Gassmann W., Hinsch M.E., Staskawicz B.J. (1999). The *Arabidopsis RPS4* bacterial-resistance gene is a member of the TIR-NBS-LRR family of disease-resistance genes. Plant J..

[129] Deslandes L., Olivier J., Theulieres F., Hirsch J., Feng D.X., Bittner-Eddy P., Beynon J., Marco Y. (2002). Resistance to *Ralstonia solanacearum* in *Arabidopsis thaliana* is conferred by the recessive *RRS1*-R gene, a member of a novel family of resistance genes. Proc. Natl. Acad. Sci. USA.

[130] Tabata S., Kaneko T., Nakamura Y., Kotani H., Kato T., Asamizu E., Miyajima N., Sasamoto S., Kimura T., Hosouchi T. (2000). Sequence and analysis of chromosome 5 of the plant *Arabidopsis thaliana*. Nature.

[131] Gómez-Gómez L., Boller T. (2000). *FLS2*: An LRR receptor-like kinase involved in the perception of the bacterial elicitor flagellin in *Arabidopsis*. Mol. Cell.

[132] Century K.S., Shapiro A.D., Repetti P.P., Dahlbeck D., Holub E., Staskawicz B.J. (1997). *NDR1*, a pathogen-induced component required for *Arabidopsis* disease resistance. Science.

[133] Swiderski M.R., Innes R.W. (2001). The *Arabidopsis PBS1* resistance gene encodes a member of a novel protein kinase subfamily. Plant J..

[134] Grant M.R., Godiard L., Straube E., Ashfield T., Lewald J., Sattler A., Innes R.W., Dangl J.L. (1995). Structure of the *Arabidopsis RPM1* gene enabling dual specificity disease resistance. Science.

[135] Tornero P., Chao R.A., Luthin W.N., Goff S.A., Dangl J.L. (2002). Large-scale structure-function analysis of the *Arabidopsis RPM1* disease resistance protein. Plant Cell.

[136] Axtell M.J., Staskawicz B.J. (2003). Initiation of *RPS2*-specified disease resistance in *Arabidopsis* is coupled to the *AvrRpt2*-directed elimination of *RIN4*. Cell.

[137] Day B., Dahlbeck D., Staskawicz B.J. (2006). *NDR1* interaction with *RIN4* mediates the differential activation of multiple disease resistance pathways in *Arabidopsis*. Plant Cell.

[138] Liu J., Elmore J.M., Lin Z.-J.D., Coaker G. (2011). A Receptor-like Cytoplasmic Kinase Phosphorylates the Host Target *RIN4*, Leading to the Activation of a Plant Innate Immune Receptor. Cell Host Microbe.

[139] Mackey D., Belkhadir Y., Alonso J.M., Ecker J.R., Dangl J.L. (2003). *Arabidopsis RIN4* is a target of the type III virulence effector *AvrRpt2* and modulates *RPS2*-mediated resistance. Cell.

[140] Mackey D., Holt B.F., Wiig A., Dangl J.L. (2002). *RIN4* Interacts with *Pseudomonas syringae* Type III Effector Molecules and Is Required for *RPM1*-Mediated Resistance in *Arabidopsis*. Cell.

[141] Warren R.F., Henk A., Mowery P., Holub E., Innes R.W. (1998). A Mutation within the Leucine-Rich Repeat Domain of the *Arabidopsis* Disease Resistance Gene *RPS5* Partially Suppresses Multiple Bacterial and Downy Mildew Resistance Genes. Plant Cell.

[142] Sarris P.F., Duxbury Z., Huh S.U., Ma Y., Segonzac C., Sklenar J., Derbyshire P., Cevik V., Rallapalli G., Saucet S.B. (2015). A Plant Immune Receptor Detects Pathogen Effectors that Target WRKY Transcription Factors. Cell.

[143] Diener A.C., Ausubel F.M. (2005). *RESISTANCE TO FUSARIUM OXYSPORUM 1*, a dominant *Arabidopsis* disease-resistance gene, is not race specific. Genetics.

[144] Shen Y., Diener A.C. (2013). *Arabidopsis thaliana RESISTANCE TO FUSARIUM OXYSPORUM 2* Implicates Tyrosine-Sulfated Peptide Signaling in Susceptibility and Resistance to Root Infection. PLoS Genet..

[145] Cole S.J., Diener A.C. (2013). Diversity in receptor-like kinase genes is a major determinant of quantitative resistance to *Fusarium oxysporum* f.sp. *matthioli*. New Phytol..

[146] Staal J., Kaliff M., Bohman S., Dixelius C. (2006). Transgressive segregation reveals two *Arabidopsis* TIR-NB-LRR resistance genes effective against *Leptosphaeria maculans*, causal agent of blackleg disease. Plant J..

[147] Ma L., Djavaheri M., Wang H., Larkan N.J., Haddadi P., Beynon E., Gropp G., Borhan M.H. (2018). *Leptosphaeria maculans* Effector Protein *AvrLm1* Modulates Plant Immunity by Enhancing MAP Kinase 9 Phosphorylation. iScience.

[148] Larkan N.J., Lydiate D.J., Parkin I.A., Nelson M.N., Epp D.J., Cowling W.A., Rimmer S.R., Borhan M.H. (2013). The Brassica napus blackleg resistance gene *LepR3* encodes a receptor-like protein triggered by the *Leptosphaeria maculans* effector *AVRLM1*. New Phytol..

[149] Larkan N.J., Ma L., Haddadi P., Buchwaldt M., Parkin I.A.P., Djavaheri M., Borhan M.H. (2020). The *Brassica napus* Wall-Associated Kinase-Like WAKL, gene *Rlm9* provides race-specific blackleg resistance. Plant J..

[150] Haddadi P., Larkan N.J., Van de Wouw A., Zhang Y., Neik T.X., Beynon E., Bayer P., Edwards D., Batley J., Borhan M.H. (2022). *Brassica napus* genes *Rlm4* and *Rlm7*, conferring resistance to *Leptosphaeria maculans*, are alleles of the *Rlm9* wall-associated kinase-like resistance locus. Plant Biotechnol. J..

[151] Staal J., Kaliff M., Dewaele E., Persson M., Dixelius C. (2008). *RLM3*, a TIR domain encoding gene involved in broad-range immunity of *Arabidopsis* to necrotrophic fungal pathogens. Plant J..

[152] Jehle A.K., Fürst U., Lipschis M., Albert M., Felix G. (2013). Perception of the novel MAMP eMax from different *Xanthomonas* species requires the *Arabidopsis* receptor-like protein *ReMAX* and the receptor kinase *SOBIR*. Plant Signal Behav..

[153] Jehle A.K., Lipschis M., Albert M., Fallahzadeh-Mamaghani V., Fürst U., Mueller K., Felix G. (2013). The Receptor-Like Protein *ReMAX* of *Arabidopsis* Detects the Microbe-Associated Molecular Pattern eMax from *Xanthomonas*. Plant Cell.

[154] Albert I., Zhang L., Bemm H., Nürnberger T. (2019). Structure-Function Analysis of Immune Receptor *AtRLP23* with Its Ligand *nlp20* and Coreceptors *AtSOBIR1* and *AtBAK1*. Mol. Plant Microbe Interact..

[155] Zhang L., Kars I., Essenstam B., Liebrand T.W.H., Wagemakers L., Elberse J., Tagkalaki P., Tjoitang D., van den Ackerveken G., van Kan J.A.L. (2014). Fungal Endopolygalacturonases Are Recognized as Microbe-Associated Molecular Patterns by the Arabidopsis Receptor-Like Protein *RESPONSIVENESS TO BOTRYTIS POLYGALACTURONASES1*. Plant Physiol..

[156] Botella M.A., Parker J.E., Frost L.N., Bittner-Eddy P.D., Beynon J.L., Daniels M.J., Holub E.B., Jones J.D.G. (1998). Three Genes of the *Arabidopsis RPP1* Complex Resistance Locus Recognize Distinct *Peronospora parasitica* Avirulence Determinants. Plant Cell.

[157] Sinapidou E., Williams K., Nott L., Bahkt S., Tör M., Crute I., Bittner-Eddy P., Beynon J. (2004). Two TIR:NB:LRR genes are required to specify resistance to *Peronospora parasitica* isolate Cala2 in *Arabidopsis*. Plant J..

[158] van der Biezen E.A., Freddie C.T., Kahn K., Parker J.E., Jones J.D. (2002). *Arabidopsis RPP4* is a member of the *RPP5* multigene family of TIR-NB-LRR genes and confers downy mildew resistance through multiple signalling components. Plant J..

[159] Parker J.E., Coleman M.J., Szabò V., Frost L.N., Schmidt R., van der Biezen E.A., Moores T., Dean C., Daniels M.J., Jones J.D. (1997). The Arabidopsis downy mildew resistance gene *RPP5* shares similarity to the toll and interleukin-1 receptors with *N* and *L6*. Plant Cell.

[160] Barragan C.A., Wu R., Kim S.-T., Xi W., Habring A., Hagmann J., Van de Weyer A.-L., Zaidem M., Ho W.W.H., Wang G. (2019). *RPW8/HR* repeats control NLR activation in *Arabidopsis thaliana*. PLoS Genet..

[161] Tsuchiya T., Eulgem T. (2013). An alternative polyadenylation mechanism coopted to the *Arabidopsis RPP7* gene through intronic retrotransposon domestication. Proc. Natl. Acad. Sci. USA.

[162] McDowell J.M., Dhandaydham M., Long T.A., Aarts M.G., Goff S., Holub E.B., Dangl J.L. (1998). Intragenic recombination and diversifying selection contribute to the evolution of downy mildew resistance at the *RPP8* locus of *Arabidopsis*. Plant Cell.

[163] Bittner-Eddy P.D., Crute I.R., Holub E.B., Beynon J.L. (2000). *RPP13* is a simple locus in *Arabidopsis thaliana* for alleles that specify downy mildew resistance to different avirulence determinants in *Peronospora parasitica*. Plant J..

[164] Goritschnig S., Krasileva K.V., Dahlbeck D., Staskawicz B.J. (2012). Computational Prediction and Molecular Characterization of an Oomycete Effector and the Cognate *Arabidopsis* Resistance Gene. PLoS Genet..

[165] Xiao S., Ellwood S., Calis O., Patrick E., Li T., Coleman M., Turner J.G. (2001). Broad-spectrum mildew resistance *in Arabidopsis thaliana* mediated by *RPW8*. Science.

[166] Borhan M.H., Holub E.B., Beynon J.L., Rozwadowski K., Rimmer S.R. (2004). The *Arabidopsis* TIR-NB-LRR gene *RAC1* confers resistance to *Albugo candida* white rust, and is dependent on *EDS1* but not *PAD4*. Mol. Plant Microbe Interact..

[167] Borhan M.H., Gunn N., Cooper A., Gulden S., Tör M., Rimmer S.R., Holub E.B. (2008). *WRR4* encodes a TIR-NB-LRR protein that confers broad-spectrum white rust resistance in *Arabidopsis thaliana* to four physiological races of *Albugo candida*. Mol. Plant Microbe Interact..

[168] Cevik V., Boutrot F., Apel W., Robert-Seilaniantz A., Furzer O.J., Redkar A., Castel B., Kover P.X., Prince D.C., Holub E.B. (2019). Transgressive segregation reveals mechanisms of *Arabidopsis* immunity to Brassica-infecting races of white rust *Albugo candida*. Proc. Natl. Acad. Sci. USA.

[169] Arora H., Padmaja K.L., Paritosh K., Mukhi N., Tewari A.K., Mukhopadhyay A., Gupta V., Pradhan A.K., Pental D. (2019). *BjuWRR1*, a CC-NB-LRR gene identified in *Brassica juncea*, confers resistance to white rust caused by *Albugo candida*. Theor. Appl. Genet..

[170] Hatakeyama K., Niwa T., Kato T., Ohara T., Kakizaki T., Matsumoto S. (2017). The tandem repeated organization of NB-LRR genes in the clubroot-resistant *CRb* locus in *Brassica rapa* L. Mol. Genet. Genom..

[171] Ueno H., Matsumoto E., Aruga D., Kitagawa S., Matsumura H., Hayashida N. (2012). Molecular characterization of the *CRa* gene conferring clubroot resistance in *Brassica rapa*. Plant Mol. Biol..

[172] Hatakeyama K., Suwabe K., Tomita R.N., Kato T., Nunome T., Fukuoka H., Matsumoto S. (2013). Identification and Characterization of *Crr1a*, a Gene for Resistance to Clubroot Disease *Plasmodiophora brassicae* Woronin, in *Brassica rapa* L. PLoS ONE.

[173] Shimizu M., Pu Z.J., Kawanabe T., Kitashiba H., Matsumoto S., Ebe Y., Sano M., Funaki T., Fukai E., Fujimoto R. (2015). Map-based cloning of a candidate gene conferring Fusarium yellows resistance in *Brassica oleracea*. Theor. Appl. Genet..

[174] Li P., Quan X., Jia G., Xiao J., Cloutier S., You F.M. (2016). RGAugury: A pipeline for genome-wide prediction of resistance gene analogs RGAs, in plants. BMC Genom..

[175] Kioukis A., Michalopoulou V.A., Briers L., Pirintsos S., Studholme D.J., Pavlidis P., Sarris P.F. (2020). Intraspecific diversification of the crop wild relative *Brassica cretica* Lam. using demographic model selection. BMC Genom..

[176] Kasianov A.S., Klepikova A.V., Kulakovskiy I.V., Gerasimov E.S., Fedotova A.V., Besedina E.G., Kondrashov A.S., Logacheva M.D., Penin A.A. (2017). High-quality genome assembly of *Capsella bursa-pastoris* reveals asymmetry of regulatory elements at early stages of polyploid genome evolution. Plant J..

[177] Platts A., Shu S., Wright S., Barry K., Edger P., Pires J.C., Schmutz J. (2020). WGS Assembly of *Sinapis alba*.

[178] Altschul S.F., Madden T.L., Schäffer A.A., Zhang J., Zhang Z., Miller W., Lipman D.J. (1997). Gapped BLAST and PSI-BLAST: A new generation of protein database search programs. Nucleic Acids Res.

[179] Rameneni J.J., Lee Y., Dhandapani V., Yu X., Choi S.R., Oh M.-H., Lim Y.P. (2015). Genomic and Post-Translational Modification Analysis of Leucine-Rich-Repeat Receptor-Like Kinases in *Brassica rapa*. PLoS ONE.

[180] Wei Z., Wang J., Yang S., Song Y. (2015). Identification and expression analysis of the LRR-RLK gene family in tomato *Solanum lycopersicum*, Heinz 1706. Genome.

[181] Yang H., Bayer P.E., Tirnaz S., Edwards D., Batley J. (2021). Genome-Wide Identification and Evolution of Receptor-Like Kinases RLKs, and Receptor like Proteins RLPs in *Brassica juncea*. Biology.

[182] Willing E.-M., Rawat V., Mandáková T., Maumus F., James G.V., Nordström K.J., Becker C., Warthmann N., Chica C., Szarzynska B. (2015). Genome expansion of *Arabis alpina* linked with retrotransposition and reduced symmetric DNA methylation. Nat. Plants.

[183] Kagale S., Koh C., Nixon J., Bollina V., Clarke W.E., Tuteja R., Spillane C., Robinson S.J., Links M.G., Clarke C. (2014). The emerging biofuel crop *Camelina sativa* retains a highly undifferentiated hexaploid genome structure. Nat. Commun..

[184] Holub E.B. (2001). The arms race is ancient history in *Arabidopsis*, the wildflower. Nat. Rev. Genet..

[185] Jupe F., Pritchard L., Etherington G.J., Mackenzie K., Cock P.J., Wright F., Sharma S.K., Bolser D., Bryan G.J., Jones J.D. (2012). Identification and localisation of the NB-LRR gene family within the potato genome. BMC Genom..

