# Supplementary material for: Predicting Cloned Disease Resistance Gene Homologs (CDRHs) in Radish, Underutilised Oilseeds, and Wild Brassicaceae Species
Source: Plants (Basel). 2022 Nov 8;11(22):3010. doi: 10.3390/plants11223010 (PMC9693284; doi:10.3390/plants11223010)
Supplement: Supplementary file 1 [file plants-11-03010-s001.zip › plants-2006666-supplementary.pdf]

Table S1. List of resistance gene analogs (RGAs) in *Brassica cretica, Capsella bursa-pastoris* , and *Sinapis alba* using the in-silico prediction of RGAugury pipeline.

Species	Gene name	RGA main class	RGA subclass
Sinapis alba	KAF8088211.1	NLR	CN
Sinapis alba	KAF8084614.1	NLR	CN
Sinapis alba	KAF8044555.1	NLR	CN
Sinapis alba	KAF8048787.1	NLR	CN
Sinapis alba	KAF8044716.1	NLR	CN
Sinapis alba	KAF8068139.1	NLR	CNL
Sinapis alba	KAF8046585.1	NLR	CNL
Sinapis alba	KAF8102307.1	NLR	CNL
Sinapis alba	KAF8064789.1	NLR	CNL
Sinapis alba	KAF8080154.1	NLR	CNL
Sinapis alba	KAF8114000.1	NLR	CNL
Sinapis alba	KAF8098470.1	NLR	CNL
Sinapis alba	KAF8058996.1	NLR	CNL
Sinapis alba	KAF8116723.1	NLR	CNL
Sinapis alba	KAF8098474.1	NLR	CNL
Sinapis alba	KAF8084217.1	NLR	CNL
Sinapis alba	KAF8081474.1	NLR	CNL
Sinapis alba	KAF8101058.1	NLR	CNL
Sinapis alba	KAF8101775.1	NLR	CNL
Sinapis alba	KAF8105611.1	NLR	CNL
Sinapis alba	KAF8105615.1	NLR	CNL
Sinapis alba	KAF8105614.1	NLR	CNL
Sinapis alba	KAF8088207.1	NLR	CNL
Sinapis alba	KAF8118946.1	NLR	CNL
Sinapis alba	KAF8093800.1	NLR	CNL
Sinapis alba	KAF8111125.1	NLR	CNL
Sinapis alba	KAF8098363.1	NLR	CNL
Sinapis alba	KAF8111118.1	NLR	CNL
Sinapis alba	KAF8051409.1	NLR	CNL
Sinapis alba	KAF8101461.1	NLR	CNL
Sinapis alba	KAF8116607.1	NLR	CNL
Sinapis alba	KAF8111124.1	NLR	CNL
Sinapis alba	KAF8096637.1	NLR	CNL
Sinapis alba	KAF8087363.1	NLR	CNL
Sinapis alba	KAF8048790.1	NLR	CNL
Sinapis alba	KAF8117183.1	NLR	CNL
Sinapis alba	KAF8114226.1	NLR	CNL
Sinapis alba	KAF8062888.1	NLR	CNL
Sinapis alba	KAF8075667.1	NLR	CNL
Sinapis alba	KAF8112165.1	NLR	CNL
Sinapis alba	KAF8075663.1	NLR	NBS
Sinapis alba	KAF8116610.1	NLR	NBS
Sinapis alba	KAF8087782.1	NLR	NBS
Sinapis alba	KAF8117245.1	NLR	NBS
Sinapis alba	KAF8045329.1	NLR	NBS
Sinapis alba	KAF8092035.1	NLR	NBS
Sinapis alba	KAF8090023.1	NLR	NBS
Sinapis alba	KAF8050205.1	NLR	NL
Sinapis alba	KAF8090380.1	NLR	NL
Sinapis alba	KAF8085476.1	NLR	NL
Sinapis alba	KAF8117260.1	NLR	NL
Sinapis alba	KAF8045188.1	NLR	NL
Sinapis alba	KAF8048786.1	NLR	NL
Sinapis alba	KAF8092678.1	NLR	NL
Sinapis alba	KAF8090867.1	NLR	NL
Sinapis alba	KAF8058994.1	NLR	NL
Sinapis alba	KAF8115254.1	NLR	NL
Sinapis alba	KAF8088212.1	NLR	NL
Sinapis alba	KAF8086877.1	NLR	NL

Sinapis alba	KAF8081484.1	NLR	NL
Sinapis alba	KAF8087542.1	NLR	NL
Sinapis alba	KAF8085594.1	NLR	NL
Sinapis alba	KAF8092033.1	NLR	NL
Sinapis alba	KAF8085331.1	NLR	NL
Sinapis alba	KAF8101662.1	NLR	NL
Sinapis alba	KAF8094635.1	NLR	NL
Sinapis alba	KAF8087560.1	NLR	NL
Sinapis alba	KAF8048268.1	NLR	NL
Sinapis alba	KAF8097061.1	NLR	NL
Sinapis alba	KAF8093454.1	NLR	NL
Sinapis alba	KAF8117197.1	NLR	NL
Sinapis alba	KAF8087218.1	NLR	NL
Sinapis alba	KAF8117264.1	NLR	NL
Sinapis alba	KAF8115077.1	NLR	Other-NLR
Sinapis alba	KAF8084593.1	NLR	Other-NLR
Sinapis alba	KAF8114969.1	NLR	Other-NLR
Sinapis alba	KAF8092032.1	NLR	Other-NLR
Sinapis alba	KAF8049004.1	NLR	Other-NLR
Sinapis alba	KAF8114287.1	NLR	Other-NLR
Sinapis alba	KAF8045627.1	NLR	Other-NLR
Sinapis alba	KAF8051100.1	NLR	TN
Sinapis alba	KAF8051466.1	NLR	TN
Sinapis alba	KAF8079928.1	NLR	TN
Sinapis alba	KAF8044651.1	NLR	TN
Sinapis alba	KAF8087785.1	NLR	TN
Sinapis alba	KAF8087787.1	NLR	TN
Sinapis alba	KAF8111292.1	NLR	TN
Sinapis alba	KAF8087781.1	NLR	TN
Sinapis alba	KAF8099129.1	NLR	TN
Sinapis alba	KAF8089379.1	NLR	TN
Sinapis alba	KAF8111293.1	NLR	TN
Sinapis alba	KAF8086279.1	NLR	TN
Sinapis alba	KAF8089377.1	NLR	TN
Sinapis alba	KAF8048616.1	NLR	TN
Sinapis alba	KAF8074292.1	NLR	TN
Sinapis alba	KAF8087792.1	NLR	TN
Sinapis alba	KAF8077334.1	NLR	TN
Sinapis alba	KAF8052724.1	NLR	TN
Sinapis alba	KAF8084295.1	NLR	TN
Sinapis alba	KAF8117268.1	NLR	TN
Sinapis alba	KAF8087964.1	NLR	TN
Sinapis alba	KAF8089378.1	NLR	TN
Sinapis alba	KAF8090866.1	NLR	TN
Sinapis alba	KAF8115160.1	NLR	TNL
Sinapis alba	KAF8077026.1	NLR	TNL
Sinapis alba	KAF8117269.1	NLR	TNL
Sinapis alba	KAF8083605.1	NLR	TNL
Sinapis alba	KAF8084537.1	NLR	TNL
Sinapis alba	KAF8115078.1	NLR	TNL
Sinapis alba	KAF8087540.1	NLR	TNL
Sinapis alba	KAF8107619.1	NLR	TNL
Sinapis alba	KAF8093263.1	NLR	TNL
Sinapis alba	KAF8082878.1	NLR	TNL
Sinapis alba	KAF8054566.1	NLR	TNL
Sinapis alba	KAF8081015.1	NLR	TNL
Sinapis alba	KAF8081016.1	NLR	TNL
Sinapis alba	KAF8094299.1	NLR	TNL
Sinapis alba	KAF8085334.1	NLR	TNL
Sinapis alba	KAF8086735.1	NLR	TNL
Sinapis alba	KAF8085330.1	NLR	TNL
Sinapis alba	KAF8085335.1	NLR	TNL

Sinapis alba	KAF8107352.1	NLR	TNL
Sinapis alba	KAF8079927.1	NLR	TNL
Sinapis alba	KAF8085333.1	NLR	TNL
Sinapis alba	KAF8112756.1	NLR	TNL
Sinapis alba	KAF8087793.1	NLR	TNL
Sinapis alba	KAF8094300.1	NLR	TNL
Sinapis alba	KAF8117196.1	NLR	TNL
Sinapis alba	KAF8103319.1	NLR	TNL
Sinapis alba	KAF8102896.1	NLR	TNL
Sinapis alba	KAF8092526.1	NLR	TNL
Sinapis alba	KAF8087561.1	NLR	TNL
Sinapis alba	KAF8117174.1	NLR	TNL
Sinapis alba	KAF8044711.1	NLR	TNL
Sinapis alba	KAF8101661.1	NLR	TNL
Sinapis alba	KAF8102895.1	NLR	TNL
Sinapis alba	KAF8082977.1	NLR	TNL
Sinapis alba	KAF8113217.1	NLR	TNL
Sinapis alba	KAF8052281.1	NLR	TNL
Sinapis alba	KAF8107617.1	NLR	TNL
Sinapis alba	KAF8101393.1	NLR	TNL
Sinapis alba	KAF8102897.1	NLR	TNL
Sinapis alba	KAF8087558.1	NLR	TNL
Sinapis alba	KAF8107618.1	NLR	TNL
Sinapis alba	KAF8091619.1	NLR	TNL
Sinapis alba	KAF8085329.1	NLR	TNL
Sinapis alba	KAF8086734.1	NLR	TNL
Sinapis alba	KAF8107622.1	NLR	TNL
Sinapis alba	KAF8063929.1	NLR	TNL
Sinapis alba	KAF8118181.1	NLR	TNL
Sinapis alba	KAF8107623.1	NLR	TNL
Sinapis alba	KAF8114288.1	NLR	TNL
Sinapis alba	KAF8112851.1	NLR	TNL
Sinapis alba	KAF8090420.1	NLR	TNL
Sinapis alba	KAF8087559.1	NLR	TNL
Sinapis alba	KAF8088333.1	NLR	TNL
Sinapis alba	KAF8110122.1	NLR	TNL
Sinapis alba	KAF8103454.1	NLR	TNL
Sinapis alba	KAF8085338.1	NLR	TNL
Sinapis alba	KAF8117194.1	NLR	TNL
Sinapis alba	KAF8075560.1	NLR	TNL
Sinapis alba	KAF8113643.1	NLR	TNL
Sinapis alba	KAF8087049.1	NLR	TNL
Sinapis alba	KAF8114966.1	NLR	TNL
Sinapis alba	KAF8051630.1	NLR	TX
Sinapis alba	KAF8114188.1	NLR	TX
Sinapis alba	KAF8044993.1	NLR	TX
Sinapis alba	KAF8116992.1	NLR	TX
Sinapis alba	KAF8077328.1	NLR	TX
Sinapis alba	KAF8118622.1	NLR	TX
Sinapis alba	KAF8081485.1	NLR	TX
Sinapis alba	KAF8044634.1	NLR	TX
Sinapis alba	KAF8084600.1	NLR	TX
Sinapis alba	KAF8048254.1	NLR	TX
Sinapis alba	KAF8084220.1	NLR	TX
Sinapis alba	KAF8079625.1	NLR	TX
Sinapis alba	KAF8053077.1	NLR	TX
Sinapis alba	KAF8077018.1	NLR	TX
Sinapis alba	KAF8062891.1	NLR	TX
Sinapis alba	KAF8117581.1	NLR	TX
Sinapis alba	KAF8085332.1	NLR	TX
Sinapis alba	KAF8110078.1	NLR	TX
Sinapis alba	KAF8091102.1	NLR	TX

Sinapis alba	KAF8074230.1	NLR	TX
Sinapis alba	KAF8109700.1	NLR	TX
Sinapis alba	KAF8092034.1	NLR	TX
Sinapis alba	KAF8087555.1	NLR	TX
Sinapis alba	KAF8087525.1	NLR	TX
Sinapis alba	KAF8085339.1	NLR	TX
Sinapis alba	KAF8102891.1	NLR	TX
Sinapis alba	KAF8117271.1	NLR	TX
Sinapis alba	KAF8086733.1	NLR	TX
Sinapis alba	KAF8045278.1	NLR	TX
Sinapis alba	KAF8087217.1	NLR	TX
Sinapis alba	KAF8084567.1	NLR	TX
Sinapis alba	KAF8094551.1	NLR	TX
Sinapis alba	KAF8061229.1	NLR	TX
Sinapis alba	KAF8077327.1	NLR	TX
Sinapis alba	KAF8084596.1	NLR	TX
Sinapis alba	KAF8051322.1	NLR	TX
Sinapis alba	KAF8064811.1	NLR	TX
Sinapis alba	KAF8117262.1	NLR	TX
Sinapis alba	KAF8118565.1	NLR	TX
Sinapis alba	KAF8087783.1	NLR	TX
Sinapis alba	KAF8046000.1	NLR	TX
Sinapis alba	KAF8050416.1	NLR	TX
Sinapis alba	KAF8054526.1	NLR	TX
Sinapis alba	KAF8117263.1	NLR	TX
Sinapis alba	KAF8044581.1	RLK	Other-RLK
Sinapis alba	KAF8044605.1	RLK	LRR
Sinapis alba	KAF8044830.1	RLK	Other-RLK
Sinapis alba	KAF8045075.1	RLK	Other-RLK
Sinapis alba	KAF8045097.1	RLK	LRR
Sinapis alba	KAF8045121.1	RLK	LRR
Sinapis alba	KAF8045145.1	RLK	Other-RLK
Sinapis alba	KAF8045146.1	RLK	Other-RLK
Sinapis alba	KAF8045147.1	RLK	Other-RLK
Sinapis alba	KAF8045148.1	RLK	Other-RLK
Sinapis alba	KAF8045149.1	RLK	Other-RLK
Sinapis alba	KAF8045150.1	RLK	Other-RLK
Sinapis alba	KAF8045175.1	RLK	Other-RLK
Sinapis alba	KAF8045406.1	RLK	Other-RLK
Sinapis alba	KAF8045471.1	RLK	Other-RLK
Sinapis alba	KAF8045476.1	RLK	Other-RLK
Sinapis alba	KAF8045724.1	RLK	LRR
Sinapis alba	KAF8045727.1	RLK	LRR
Sinapis alba	KAF8045753.1	RLK	Other-RLK
Sinapis alba	KAF8045754.1	RLK	Other-RLK
Sinapis alba	KAF8045774.1	RLK	LRR
Sinapis alba	KAF8045781.1	RLK	LRR
Sinapis alba	KAF8045826.1	RLK	LRR
Sinapis alba	KAF8045977.1	RLK	Other-RLK
Sinapis alba	KAF8046042.1	RLK	LRR
Sinapis alba	KAF8046082.1	RLK	Other-RLK
Sinapis alba	KAF8046083.1	RLK	Other-RLK
Sinapis alba	KAF8046134.1	RLK	Other-RLK
Sinapis alba	KAF8046138.1	RLK	Other-RLK
Sinapis alba	KAF8046159.1	RLK	LRR
Sinapis alba	KAF8046254.1	RLK	Other-RLK
Sinapis alba	KAF8046256.1	RLK	LRR
Sinapis alba	KAF8046393.1	RLK	Other-RLK
Sinapis alba	KAF8046394.1	RLK	Other-RLK
Sinapis alba	KAF8046395.1	RLK	Other-RLK
Sinapis alba	KAF8046438.1	RLK	Other-RLK
Sinapis alba	KAF8046613.1	RLK	LRR

Sinapis alba	KAF8046626.1	RLK	Other-RLK
Sinapis alba	KAF8046873.1	RLK	Other-RLK
Sinapis alba	KAF8046876.1	RLK	Other-RLK
Sinapis alba	KAF8046877.1	RLK	Other-RLK
Sinapis alba	KAF8046878.1	RLK	Other-RLK
Sinapis alba	KAF8046896.1	RLK	Other-RLK
Sinapis alba	KAF8046954.1	RLK	Other-RLK
Sinapis alba	KAF8047075.1	RLK	Other-RLK
Sinapis alba	KAF8047283.1	RLK	Other-RLK
Sinapis alba	KAF8047285.1	RLK	Other-RLK
Sinapis alba	KAF8047286.1	RLK	Other-RLK
Sinapis alba	KAF8047287.1	RLK	Other-RLK
Sinapis alba	KAF8047296.1	RLK	Other-RLK
Sinapis alba	KAF8047306.1	RLK	Other-RLK
Sinapis alba	KAF8047419.1	RLK	Other-RLK
Sinapis alba	KAF8047494.1	RLK	Other-RLK
Sinapis alba	KAF8047545.1	RLK	Other-RLK
Sinapis alba	KAF8047590.1	RLK	Other-RLK
Sinapis alba	KAF8047594.1	RLK	Other-RLK
Sinapis alba	KAF8047650.1	RLK	LRR
Sinapis alba	KAF8047699.1	RLK	LRR
Sinapis alba	KAF8047710.1	RLK	Other-RLK
Sinapis alba	KAF8047997.1	RLK	Other-RLK
Sinapis alba	KAF8047998.1	RLK	Other-RLK
Sinapis alba	KAF8048027.1	RLK	LRR
Sinapis alba	KAF8048057.1	RLK	LRR
Sinapis alba	KAF8048129.1	RLK	Other-RLK
Sinapis alba	KAF8048194.1	RLK	LRR
Sinapis alba	KAF8048275.1	RLK	Other-RLK
Sinapis alba	KAF8048434.1	RLK	LRR
Sinapis alba	KAF8048439.1	RLK	LRR
Sinapis alba	KAF8048467.1	RLK	Other-RLK
Sinapis alba	KAF8048816.1	RLK	Other-RLK
Sinapis alba	KAF8049075.1	RLK	LRR
Sinapis alba	KAF8049083.1	RLK	LRR
Sinapis alba	KAF8049134.1	RLK	LRR
Sinapis alba	KAF8049135.1	RLK	LRR
Sinapis alba	KAF8049145.1	RLK	LRR
Sinapis alba	KAF8049152.1	RLK	Other-RLK
Sinapis alba	KAF8049180.1	RLK	LRR
Sinapis alba	KAF8049374.1	RLK	LRR
Sinapis alba	KAF8049426.1	RLK	LRR
Sinapis alba	KAF8049604.1	RLK	Other-RLK
Sinapis alba	KAF8049745.1	RLK	Other-RLK
Sinapis alba	KAF8049847.1	RLK	LRR
Sinapis alba	KAF8049912.1	RLK	Other-RLK
Sinapis alba	KAF8049913.1	RLK	Other-RLK
Sinapis alba	KAF8049920.1	RLK	LRR
Sinapis alba	KAF8049936.1	RLK	LRR
Sinapis alba	KAF8050044.1	RLK	LRR
Sinapis alba	KAF8050072.1	RLK	Other-RLK
Sinapis alba	KAF8050073.1	RLK	Other-RLK
Sinapis alba	KAF8050102.1	RLK	Other-RLK
Sinapis alba	KAF8050103.1	RLK	LRR
Sinapis alba	KAF8050104.1	RLK	LRR
Sinapis alba	KAF8050156.1	RLK	LRR
Sinapis alba	KAF8050182.1	RLK	Other-RLK
Sinapis alba	KAF8050199.1	RLK	LRR
Sinapis alba	KAF8050207.1	RLK	LRR
Sinapis alba	KAF8050222.1	RLK	Other-RLK
Sinapis alba	KAF8050228.1	RLK	LRR
Sinapis alba	KAF8050229.1	RLK	LRR

Sinapis alba	KAF8050270.1	RLK	LRR
Sinapis alba	KAF8050319.1	RLK	LRR
Sinapis alba	KAF8050373.1	RLK	Other-RLK
Sinapis alba	KAF8050383.1	RLK	Other-RLK
Sinapis alba	KAF8050435.1	RLK	Other-RLK
Sinapis alba	KAF8050490.1	RLK	LRR
Sinapis alba	KAF8050599.1	RLK	LRR
Sinapis alba	KAF8050616.1	RLK	LRR
Sinapis alba	KAF8050898.1	RLK	LRR
Sinapis alba	KAF8050994.1	RLK	Other-RLK
Sinapis alba	KAF8051032.1	RLK	LRR
Sinapis alba	KAF8051069.1	RLK	Other-RLK
Sinapis alba	KAF8051075.1	RLK	Other-RLK
Sinapis alba	KAF8051096.1	RLK	LRR
Sinapis alba	KAF8051136.1	RLK	Other-RLK
Sinapis alba	KAF8051159.1	RLK	Other-RLK
Sinapis alba	KAF8051172.1	RLK	Other-RLK
Sinapis alba	KAF8051231.1	RLK	Other-RLK
Sinapis alba	KAF8051232.1	RLK	Other-RLK
Sinapis alba	KAF8051299.1	RLK	Other-RLK
Sinapis alba	KAF8051416.1	RLK	LRR
Sinapis alba	KAF8051453.1	RLK	Other-RLK
Sinapis alba	KAF8051478.1	RLK	LRR
Sinapis alba	KAF8051686.1	RLK	Other-RLK
Sinapis alba	KAF8051705.1	RLK	Other-RLK
Sinapis alba	KAF8051827.1	RLK	Other-RLK
Sinapis alba	KAF8051828.1	RLK	Other-RLK
Sinapis alba	KAF8051848.1	RLK	Other-RLK
Sinapis alba	KAF8051911.1	RLK	Other-RLK
Sinapis alba	KAF8052084.1	RLK	Other-RLK
Sinapis alba	KAF8052214.1	RLK	Other-RLK
Sinapis alba	KAF8052271.1	RLK	Other-RLK
Sinapis alba	KAF8052272.1	RLK	Other-RLK
Sinapis alba	KAF8052368.1	RLK	LRR
Sinapis alba	KAF8052393.1	RLK	Other-RLK
Sinapis alba	KAF8052477.1	RLK	LRR
Sinapis alba	KAF8052546.1	RLK	Other-RLK
Sinapis alba	KAF8052555.1	RLK	LRR
Sinapis alba	KAF8052678.1	RLK	Other-RLK
Sinapis alba	KAF8052744.1	RLK	Other-RLK
Sinapis alba	KAF8052749.1	RLK	LRR
Sinapis alba	KAF8052812.1	RLK	Other-RLK
Sinapis alba	KAF8052813.1	RLK	Other-RLK
Sinapis alba	KAF8052845.1	RLK	LRR
Sinapis alba	KAF8052887.1	RLK	Other-RLK
Sinapis alba	KAF8052888.1	RLK	Other-RLK
Sinapis alba	KAF8052944.1	RLK	LRR
Sinapis alba	KAF8053345.1	RLK	Other-RLK
Sinapis alba	KAF8053368.1	RLK	LRR
Sinapis alba	KAF8053444.1	RLK	Other-RLK
Sinapis alba	KAF8053511.1	RLK	Other-RLK
Sinapis alba	KAF8053513.1	RLK	LRR
Sinapis alba	KAF8053557.1	RLK	Other-RLK
Sinapis alba	KAF8053680.1	RLK	LRR
Sinapis alba	KAF8053693.1	RLK	LRR
Sinapis alba	KAF8053832.1	RLK	Other-RLK
Sinapis alba	KAF8053910.1	RLK	Other-RLK
Sinapis alba	KAF8054040.1	RLK	LRR
Sinapis alba	KAF8054820.1	RLK	Other-RLK
Sinapis alba	KAF8054821.1	RLK	Other-RLK
Sinapis alba	KAF8055071.1	RLK	Other-RLK
Sinapis alba	KAF8055134.1	RLK	Other-RLK

Sinapis alba	KAF8055259.1	RLK	LRR
Sinapis alba	KAF8055713.1	RLK	Other-RLK
Sinapis alba	KAF8055715.1	RLK	Other-RLK
Sinapis alba	KAF8055982.1	RLK	Other-RLK
Sinapis alba	KAF8056422.1	RLK	LRR
Sinapis alba	KAF8057226.1	RLK	Other-RLK
Sinapis alba	KAF8057305.1	RLK	Other-RLK
Sinapis alba	KAF8057339.1	RLK	LRR
Sinapis alba	KAF8059007.1	RLK	LRR
Sinapis alba	KAF8059553.1	RLK	LRR
Sinapis alba	KAF8059953.1	RLK	LRR
Sinapis alba	KAF8060397.1	RLK	LRR
Sinapis alba	KAF8060448.1	RLK	Other-RLK
Sinapis alba	KAF8061186.1	RLK	LRR
Sinapis alba	KAF8061219.1	RLK	Other-RLK
Sinapis alba	KAF8061267.1	RLK	Other-RLK
Sinapis alba	KAF8062596.1	RLK	Other-RLK
Sinapis alba	KAF8062597.1	RLK	Other-RLK
Sinapis alba	KAF8062600.1	RLK	Other-RLK
Sinapis alba	KAF8062607.1	RLK	Other-RLK
Sinapis alba	KAF8062871.1	RLK	LRR
Sinapis alba	KAF8063122.1	RLK	Other-RLK
Sinapis alba	KAF8064809.1	RLK	Other-RLK
Sinapis alba	KAF8065678.1	RLK	LRR
Sinapis alba	KAF8066148.1	RLK	LRR
Sinapis alba	KAF8068469.1	RLK	LRR
Sinapis alba	KAF8068506.1	RLK	Other-RLK
Sinapis alba	KAF8069360.1	RLK	Other-RLK
Sinapis alba	KAF8069776.1	RLK	LRR
Sinapis alba	KAF8069777.1	RLK	LRR
Sinapis alba	KAF8073271.1	RLK	LRR
Sinapis alba	KAF8073295.1	RLK	Other-RLK
Sinapis alba	KAF8074393.1	RLK	LRR
Sinapis alba	KAF8075444.1	RLK	Other-RLK
Sinapis alba	KAF8075480.1	RLK	LRR
Sinapis alba	KAF8075481.1	RLK	LRR
Sinapis alba	KAF8075515.1	RLK	LRR
Sinapis alba	KAF8075516.1	RLK	LRR
Sinapis alba	KAF8075517.1	RLK	LRR
Sinapis alba	KAF8075518.1	RLK	LRR
Sinapis alba	KAF8075519.1	RLK	LRR
Sinapis alba	KAF8075543.1	RLK	Other-RLK
Sinapis alba	KAF8075650.1	RLK	Other-RLK
Sinapis alba	KAF8077040.1	RLK	Other-RLK
Sinapis alba	KAF8077064.1	RLK	LRR
Sinapis alba	KAF8077080.1	RLK	LRR
Sinapis alba	KAF8077090.1	RLK	Other-RLK
Sinapis alba	KAF8077174.1	RLK	Other-RLK
Sinapis alba	KAF8077189.1	RLK	Other-RLK
Sinapis alba	KAF8077211.1	RLK	Other-RLK
Sinapis alba	KAF8077212.1	RLK	Other-RLK
Sinapis alba	KAF8077213.1	RLK	Other-RLK
Sinapis alba	KAF8077216.1	RLK	Other-RLK
Sinapis alba	KAF8077347.1	RLK	Other-RLK
Sinapis alba	KAF8077372.1	RLK	Other-RLK
Sinapis alba	KAF8077543.1	RLK	Other-RLK
Sinapis alba	KAF8079542.1	RLK	LRR
Sinapis alba	KAF8079950.1	RLK	Other-RLK
Sinapis alba	KAF8080065.1	RLK	LRR
Sinapis alba	KAF8080098.1	RLK	Other-RLK
Sinapis alba	KAF8080140.1	RLK	LRR
Sinapis alba	KAF8080151.1	RLK	LRR

Sinapis alba	KAF8080165.1	RLK	Other-RLK
Sinapis alba	KAF8080188.1	RLK	Other-RLK
Sinapis alba	KAF8080192.1	RLK	Other-RLK
Sinapis alba	KAF8080195.1	RLK	Other-RLK
Sinapis alba	KAF8080206.1	RLK	Other-RLK
Sinapis alba	KAF8080264.1	RLK	Other-RLK
Sinapis alba	KAF8080361.1	RLK	LRR
Sinapis alba	KAF8080368.1	RLK	Other-RLK
Sinapis alba	KAF8080475.1	RLK	LRR
Sinapis alba	KAF8080476.1	RLK	LRR
Sinapis alba	KAF8080588.1	RLK	Other-RLK
Sinapis alba	KAF8080603.1	RLK	Other-RLK
Sinapis alba	KAF8080624.1	RLK	LRR
Sinapis alba	KAF8080642.1	RLK	Other-RLK
Sinapis alba	KAF8080655.1	RLK	LRR
Sinapis alba	KAF8080741.1	RLK	Other-RLK
Sinapis alba	KAF8080815.1	RLK	Other-RLK
Sinapis alba	KAF8080816.1	RLK	Other-RLK
Sinapis alba	KAF8080859.1	RLK	LRR
Sinapis alba	KAF8080989.1	RLK	LRR
Sinapis alba	KAF8081036.1	RLK	Other-RLK
Sinapis alba	KAF8081096.1	RLK	Other-RLK
Sinapis alba	KAF8081155.1	RLK	Other-RLK
Sinapis alba	KAF8081344.1	RLK	Other-RLK
Sinapis alba	KAF8081345.1	RLK	Other-RLK
Sinapis alba	KAF8081493.1	RLK	Other-RLK
Sinapis alba	KAF8081621.1	RLK	LRR
Sinapis alba	KAF8081624.1	RLK	Other-RLK
Sinapis alba	KAF8081625.1	RLK	LRR
Sinapis alba	KAF8081629.1	RLK	LRR
Sinapis alba	KAF8081804.1	RLK	Lysm
Sinapis alba	KAF8081855.1	RLK	LRR
Sinapis alba	KAF8081880.1	RLK	Other-RLK
Sinapis alba	KAF8081949.1	RLK	Other-RLK
Sinapis alba	KAF8081958.1	RLK	Other-RLK
Sinapis alba	KAF8081966.1	RLK	Other-RLK
Sinapis alba	KAF8082095.1	RLK	LRR
Sinapis alba	KAF8082408.1	RLK	LRR
Sinapis alba	KAF8082439.1	RLK	Other-RLK
Sinapis alba	KAF8082440.1	RLK	Other-RLK
Sinapis alba	KAF8082441.1	RLK	Other-RLK
Sinapis alba	KAF8082442.1	RLK	Other-RLK
Sinapis alba	KAF8082444.1	RLK	Other-RLK
Sinapis alba	KAF8082445.1	RLK	Other-RLK
Sinapis alba	KAF8082446.1	RLK	Other-RLK
Sinapis alba	KAF8082447.1	RLK	Other-RLK
Sinapis alba	KAF8082448.1	RLK	Other-RLK
Sinapis alba	KAF8082449.1	RLK	Other-RLK
Sinapis alba	KAF8082542.1	RLK	Other-RLK
Sinapis alba	KAF8082673.1	RLK	LRR
Sinapis alba	KAF8082692.1	RLK	Other-RLK
Sinapis alba	KAF8082693.1	RLK	Other-RLK
Sinapis alba	KAF8082703.1	RLK	LRR
Sinapis alba	KAF8082704.1	RLK	LRR
Sinapis alba	KAF8082705.1	RLK	LRR
Sinapis alba	KAF8082706.1	RLK	LRR
Sinapis alba	KAF8082707.1	RLK	LRR
Sinapis alba	KAF8082723.1	RLK	LRR
Sinapis alba	KAF8082753.1	RLK	Other-RLK
Sinapis alba	KAF8082754.1	RLK	Other-RLK
Sinapis alba	KAF8082755.1	RLK	Other-RLK
Sinapis alba	KAF8082797.1	RLK	Other-RLK

Sinapis alba	KAF8082914.1	RLK	LRR
Sinapis alba	KAF8083036.1	RLK	LRR
Sinapis alba	KAF8083132.1	RLK	LRR
Sinapis alba	KAF8083319.1	RLK	Other-RLK
Sinapis alba	KAF8083382.1	RLK	Other-RLK
Sinapis alba	KAF8083387.1	RLK	Other-RLK
Sinapis alba	KAF8083388.1	RLK	Other-RLK
Sinapis alba	KAF8083389.1	RLK	Other-RLK
Sinapis alba	KAF8083408.1	RLK	Lysm
Sinapis alba	KAF8083409.1	RLK	LRR
Sinapis alba	KAF8083411.1	RLK	LRR
Sinapis alba	KAF8083413.1	RLK	Other-RLK
Sinapis alba	KAF8083478.1	RLK	LRR
Sinapis alba	KAF8083492.1	RLK	Lysm
Sinapis alba	KAF8083604.1	RLK	Other-RLK
Sinapis alba	KAF8083632.1	RLK	Other-RLK
Sinapis alba	KAF8083637.1	RLK	Other-RLK
Sinapis alba	KAF8083639.1	RLK	LRR
Sinapis alba	KAF8083760.1	RLK	Other-RLK
Sinapis alba	KAF8083788.1	RLK	LRR
Sinapis alba	KAF8083789.1	RLK	LRR
Sinapis alba	KAF8083790.1	RLK	LRR
Sinapis alba	KAF8084036.1	RLK	Other-RLK
Sinapis alba	KAF8084052.1	RLK	LRR
Sinapis alba	KAF8084053.1	RLK	LRR
Sinapis alba	KAF8084054.1	RLK	LRR
Sinapis alba	KAF8084113.1	RLK	Other-RLK
Sinapis alba	KAF8084133.1	RLK	Other-RLK
Sinapis alba	KAF8084271.1	RLK	Other-RLK
Sinapis alba	KAF8084443.1	RLK	LRR
Sinapis alba	KAF8084514.1	RLK	Other-RLK
Sinapis alba	KAF8084594.1	RLK	Other-RLK
Sinapis alba	KAF8084787.1	RLK	LRR
Sinapis alba	KAF8084859.1	RLK	Other-RLK
Sinapis alba	KAF8085023.1	RLK	Other-RLK
Sinapis alba	KAF8085193.1	RLK	Other-RLK
Sinapis alba	KAF8085212.1	RLK	Other-RLK
Sinapis alba	KAF8085491.1	RLK	Other-RLK
Sinapis alba	KAF8085492.1	RLK	Other-RLK
Sinapis alba	KAF8085508.1	RLK	Other-RLK
Sinapis alba	KAF8085672.1	RLK	Other-RLK
Sinapis alba	KAF8085676.1	RLK	LRR
Sinapis alba	KAF8085722.1	RLK	Other-RLK
Sinapis alba	KAF8085741.1	RLK	Other-RLK
Sinapis alba	KAF8085807.1	RLK	Other-RLK
Sinapis alba	KAF8085838.1	RLK	Other-RLK
Sinapis alba	KAF8085879.1	RLK	Other-RLK
Sinapis alba	KAF8085881.1	RLK	Other-RLK
Sinapis alba	KAF8085882.1	RLK	Other-RLK
Sinapis alba	KAF8085883.1	RLK	Other-RLK
Sinapis alba	KAF8085884.1	RLK	Other-RLK
Sinapis alba	KAF8085885.1	RLK	Other-RLK
Sinapis alba	KAF8085886.1	RLK	Other-RLK
Sinapis alba	KAF8085887.1	RLK	Other-RLK
Sinapis alba	KAF8085888.1	RLK	Other-RLK
Sinapis alba	KAF8086055.1	RLK	Other-RLK
Sinapis alba	KAF8086116.1	RLK	Other-RLK
Sinapis alba	KAF8086117.1	RLK	Other-RLK
Sinapis alba	KAF8086163.1	RLK	LRR
Sinapis alba	KAF8086233.1	RLK	LRR
Sinapis alba	KAF8086238.1	RLK	LRR
Sinapis alba	KAF8086311.1	RLK	LRR

Sinapis alba	KAF8086456.1	RLK	Other-RLK
Sinapis alba	KAF8086457.1	RLK	Other-RLK
Sinapis alba	KAF8086459.1	RLK	Other-RLK
Sinapis alba	KAF8086507.1	RLK	Other-RLK
Sinapis alba	KAF8086538.1	RLK	Other-RLK
Sinapis alba	KAF8086736.1	RLK	LRR
Sinapis alba	KAF8086780.1	RLK	Other-RLK
Sinapis alba	KAF8086781.1	RLK	Other-RLK
Sinapis alba	KAF8086784.1	RLK	Other-RLK
Sinapis alba	KAF8086785.1	RLK	Other-RLK
Sinapis alba	KAF8086798.1	RLK	Other-RLK
Sinapis alba	KAF8086799.1	RLK	Other-RLK
Sinapis alba	KAF8086800.1	RLK	Other-RLK
Sinapis alba	KAF8086801.1	RLK	Other-RLK
Sinapis alba	KAF8086862.1	RLK	Other-RLK
Sinapis alba	KAF8086868.1	RLK	Other-RLK
Sinapis alba	KAF8086968.1	RLK	LRR
Sinapis alba	KAF8087059.1	RLK	Other-RLK
Sinapis alba	KAF8087067.1	RLK	Other-RLK
Sinapis alba	KAF8087215.1	RLK	LRR
Sinapis alba	KAF8087242.1	RLK	LRR
Sinapis alba	KAF8087245.1	RLK	Other-RLK
Sinapis alba	KAF8087246.1	RLK	Other-RLK
Sinapis alba	KAF8087314.1	RLK	LRR
Sinapis alba	KAF8087394.1	RLK	LRR
Sinapis alba	KAF8087395.1	RLK	LRR
Sinapis alba	KAF8087571.1	RLK	Other-RLK
Sinapis alba	KAF8087671.1	RLK	LRR
Sinapis alba	KAF8087893.1	RLK	Other-RLK
Sinapis alba	KAF8087904.1	RLK	Other-RLK
Sinapis alba	KAF8087905.1	RLK	Other-RLK
Sinapis alba	KAF8087986.1	RLK	Other-RLK
Sinapis alba	KAF8088030.1	RLK	LRR
Sinapis alba	KAF8088056.1	RLK	Other-RLK
Sinapis alba	KAF8088085.1	RLK	Other-RLK
Sinapis alba	KAF8088157.1	RLK	Other-RLK
Sinapis alba	KAF8088256.1	RLK	Other-RLK
Sinapis alba	KAF8088282.1	RLK	Other-RLK
Sinapis alba	KAF8088326.1	RLK	Other-RLK
Sinapis alba	KAF8088327.1	RLK	Other-RLK
Sinapis alba	KAF8088382.1	RLK	LRR
Sinapis alba	KAF8088435.1	RLK	Other-RLK
Sinapis alba	KAF8088436.1	RLK	Other-RLK
Sinapis alba	KAF8088437.1	RLK	Other-RLK
Sinapis alba	KAF8088804.1	RLK	LRR
Sinapis alba	KAF8088822.1	RLK	Other-RLK
Sinapis alba	KAF8088834.1	RLK	Other-RLK
Sinapis alba	KAF8089027.1	RLK	Other-RLK
Sinapis alba	KAF8089073.1	RLK	LRR
Sinapis alba	KAF8089095.1	RLK	LRR
Sinapis alba	KAF8089097.1	RLK	LRR
Sinapis alba	KAF8089125.1	RLK	LRR
Sinapis alba	KAF8089127.1	RLK	LRR
Sinapis alba	KAF8089128.1	RLK	LRR
Sinapis alba	KAF8089129.1	RLK	LRR
Sinapis alba	KAF8089131.1	RLK	LRR
Sinapis alba	KAF8089132.1	RLK	LRR
Sinapis alba	KAF8089134.1	RLK	LRR
Sinapis alba	KAF8089271.1	RLK	Other-RLK
Sinapis alba	KAF8089274.1	RLK	LRR
Sinapis alba	KAF8089316.1	RLK	LRR
Sinapis alba	KAF8089369.1	RLK	LRR

Sinapis alba	KAF8089456.1	RLK	Other-RLK
Sinapis alba	KAF8089493.1	RLK	LRR
Sinapis alba	KAF8089794.1	RLK	LRR
Sinapis alba	KAF8089814.1	RLK	Other-RLK
Sinapis alba	KAF8089815.1	RLK	Other-RLK
Sinapis alba	KAF8089890.1	RLK	LRR
Sinapis alba	KAF8089891.1	RLK	Other-RLK
Sinapis alba	KAF8089950.1	RLK	Other-RLK
Sinapis alba	KAF8089979.1	RLK	Other-RLK
Sinapis alba	KAF8089986.1	RLK	Other-RLK
Sinapis alba	KAF8090139.1	RLK	Other-RLK
Sinapis alba	KAF8090261.1	RLK	LRR
Sinapis alba	KAF8090271.1	RLK	LRR
Sinapis alba	KAF8090323.1	RLK	Other-RLK
Sinapis alba	KAF8090414.1	RLK	LRR
Sinapis alba	KAF8090446.1	RLK	Other-RLK
Sinapis alba	KAF8090522.1	RLK	Other-RLK
Sinapis alba	KAF8090601.1	RLK	LRR
Sinapis alba	KAF8090664.1	RLK	Other-RLK
Sinapis alba	KAF8090675.1	RLK	Other-RLK
Sinapis alba	KAF8090676.1	RLK	Other-RLK
Sinapis alba	KAF8090678.1	RLK	Other-RLK
Sinapis alba	KAF8090679.1	RLK	Other-RLK
Sinapis alba	KAF8090680.1	RLK	Other-RLK
Sinapis alba	KAF8090687.1	RLK	Other-RLK
Sinapis alba	KAF8090688.1	RLK	Other-RLK
Sinapis alba	KAF8090697.1	RLK	Other-RLK
Sinapis alba	KAF8090776.1	RLK	LRR
Sinapis alba	KAF8090841.1	RLK	Other-RLK
Sinapis alba	KAF8090843.1	RLK	Other-RLK
Sinapis alba	KAF8090927.1	RLK	LRR
Sinapis alba	KAF8091178.1	RLK	Other-RLK
Sinapis alba	KAF8091180.1	RLK	Other-RLK
Sinapis alba	KAF8091190.1	RLK	LRR
Sinapis alba	KAF8091352.1	RLK	Other-RLK
Sinapis alba	KAF8091387.1	RLK	LRR
Sinapis alba	KAF8091437.1	RLK	Other-RLK
Sinapis alba	KAF8091438.1	RLK	Other-RLK
Sinapis alba	KAF8091446.1	RLK	Other-RLK
Sinapis alba	KAF8091612.1	RLK	Other-RLK
Sinapis alba	KAF8091613.1	RLK	Other-RLK
Sinapis alba	KAF8091614.1	RLK	Other-RLK
Sinapis alba	KAF8091616.1	RLK	Other-RLK
Sinapis alba	KAF8091617.1	RLK	Other-RLK
Sinapis alba	KAF8091622.1	RLK	LRR
Sinapis alba	KAF8091723.1	RLK	Other-RLK
Sinapis alba	KAF8091788.1	RLK	Other-RLK
Sinapis alba	KAF8091912.1	RLK	LRR
Sinapis alba	KAF8091913.1	RLK	LRR
Sinapis alba	KAF8091927.1	RLK	LRR
Sinapis alba	KAF8091942.1	RLK	Other-RLK
Sinapis alba	KAF8091982.1	RLK	Other-RLK
Sinapis alba	KAF8091992.1	RLK	Other-RLK
Sinapis alba	KAF8091997.1	RLK	LRR
Sinapis alba	KAF8092163.1	RLK	Other-RLK
Sinapis alba	KAF8092175.1	RLK	LRR
Sinapis alba	KAF8092265.1	RLK	LRR
Sinapis alba	KAF8092471.1	RLK	LRR
Sinapis alba	KAF8092533.1	RLK	Other-RLK
Sinapis alba	KAF8092717.1	RLK	Lysm
Sinapis alba	KAF8092753.1	RLK	LRR
Sinapis alba	KAF8092776.1	RLK	LRR

Sinapis alba	KAF8092801.1	RLK	Other-RLK
Sinapis alba	KAF8092822.1	RLK	Other-RLK
Sinapis alba	KAF8092883.1	RLK	Other-RLK
Sinapis alba	KAF8092948.1	RLK	Other-RLK
Sinapis alba	KAF8093016.1	RLK	Other-RLK
Sinapis alba	KAF8093019.1	RLK	Other-RLK
Sinapis alba	KAF8093020.1	RLK	Other-RLK
Sinapis alba	KAF8093021.1	RLK	Other-RLK
Sinapis alba	KAF8093022.1	RLK	Other-RLK
Sinapis alba	KAF8093023.1	RLK	Other-RLK
Sinapis alba	KAF8093024.1	RLK	Other-RLK
Sinapis alba	KAF8093091.1	RLK	Other-RLK
Sinapis alba	KAF8093132.1	RLK	Other-RLK
Sinapis alba	KAF8093164.1	RLK	LRR
Sinapis alba	KAF8093170.1	RLK	LRR
Sinapis alba	KAF8093180.1	RLK	Other-RLK
Sinapis alba	KAF8093324.1	RLK	LRR
Sinapis alba	KAF8093507.1	RLK	Other-RLK
Sinapis alba	KAF8093549.1	RLK	Other-RLK
Sinapis alba	KAF8093834.1	RLK	LRR
Sinapis alba	KAF8093842.1	RLK	LRR
Sinapis alba	KAF8093843.1	RLK	LRR
Sinapis alba	KAF8093891.1	RLK	Other-RLK
Sinapis alba	KAF8093935.1	RLK	LRR
Sinapis alba	KAF8094022.1	RLK	Other-RLK
Sinapis alba	KAF8094052.1	RLK	LRR
Sinapis alba	KAF8094086.1	RLK	Other-RLK
Sinapis alba	KAF8094102.1	RLK	LRR
Sinapis alba	KAF8094127.1	RLK	LRR
Sinapis alba	KAF8094155.1	RLK	LRR
Sinapis alba	KAF8094156.1	RLK	LRR
Sinapis alba	KAF8094336.1	RLK	Other-RLK
Sinapis alba	KAF8094497.1	RLK	Other-RLK
Sinapis alba	KAF8094531.1	RLK	Other-RLK
Sinapis alba	KAF8094532.1	RLK	Other-RLK
Sinapis alba	KAF8094539.1	RLK	Other-RLK
Sinapis alba	KAF8094584.1	RLK	Other-RLK
Sinapis alba	KAF8094683.1	RLK	LRR
Sinapis alba	KAF8094729.1	RLK	Other-RLK
Sinapis alba	KAF8094747.1	RLK	LRR
Sinapis alba	KAF8094768.1	RLK	Other-RLK
Sinapis alba	KAF8094794.1	RLK	LRR
Sinapis alba	KAF8094795.1	RLK	LRR
Sinapis alba	KAF8094796.1	RLK	LRR
Sinapis alba	KAF8094797.1	RLK	LRR
Sinapis alba	KAF8094798.1	RLK	LRR
Sinapis alba	KAF8095064.1	RLK	LRR
Sinapis alba	KAF8095306.1	RLK	Other-RLK
Sinapis alba	KAF8095362.1	RLK	Other-RLK
Sinapis alba	KAF8095409.1	RLK	LRR
Sinapis alba	KAF8095467.1	RLK	Other-RLK
Sinapis alba	KAF8095610.1	RLK	LRR
Sinapis alba	KAF8095624.1	RLK	LRR
Sinapis alba	KAF8095625.1	RLK	LRR
Sinapis alba	KAF8095626.1	RLK	LRR
Sinapis alba	KAF8095690.1	RLK	LRR
Sinapis alba	KAF8095731.1	RLK	Other-RLK
Sinapis alba	KAF8095790.1	RLK	Other-RLK
Sinapis alba	KAF8095791.1	RLK	Other-RLK
Sinapis alba	KAF8095794.1	RLK	Other-RLK
Sinapis alba	KAF8095859.1	RLK	Other-RLK
Sinapis alba	KAF8095865.1	RLK	LRR

Sinapis alba	KAF8095876.1	RLK	Other-RLK
Sinapis alba	KAF8095877.1	RLK	Other-RLK
Sinapis alba	KAF8095939.1	RLK	Other-RLK
Sinapis alba	KAF8095940.1	RLK	Other-RLK
Sinapis alba	KAF8095941.1	RLK	Other-RLK
Sinapis alba	KAF8095942.1	RLK	LRR
Sinapis alba	KAF8095943.1	RLK	Other-RLK
Sinapis alba	KAF8095948.1	RLK	Other-RLK
Sinapis alba	KAF8095949.1	RLK	Other-RLK
Sinapis alba	KAF8095950.1	RLK	Other-RLK
Sinapis alba	KAF8096053.1	RLK	LRR
Sinapis alba	KAF8096107.1	RLK	LRR
Sinapis alba	KAF8096113.1	RLK	LRR
Sinapis alba	KAF8096127.1	RLK	LRR
Sinapis alba	KAF8096204.1	RLK	Other-RLK
Sinapis alba	KAF8096329.1	RLK	LRR
Sinapis alba	KAF8096534.1	RLK	Other-RLK
Sinapis alba	KAF8096575.1	RLK	LRR
Sinapis alba	KAF8096577.1	RLK	LRR
Sinapis alba	KAF8096684.1	RLK	LRR
Sinapis alba	KAF8096699.1	RLK	LRR
Sinapis alba	KAF8096733.1	RLK	Other-RLK
Sinapis alba	KAF8096843.1	RLK	LRR
Sinapis alba	KAF8096888.1	RLK	LRR
Sinapis alba	KAF8096976.1	RLK	Other-RLK
Sinapis alba	KAF8096977.1	RLK	Other-RLK
Sinapis alba	KAF8096978.1	RLK	Other-RLK
Sinapis alba	KAF8096988.1	RLK	Other-RLK
Sinapis alba	KAF8097069.1	RLK	LRR
Sinapis alba	KAF8097402.1	RLK	Other-RLK
Sinapis alba	KAF8097443.1	RLK	LRR
Sinapis alba	KAF8097448.1	RLK	Other-RLK
Sinapis alba	KAF8097675.1	RLK	Other-RLK
Sinapis alba	KAF8097676.1	RLK	Other-RLK
Sinapis alba	KAF8097764.1	RLK	Other-RLK
Sinapis alba	KAF8097816.1	RLK	LRR
Sinapis alba	KAF8097840.1	RLK	Other-RLK
Sinapis alba	KAF8097919.1	RLK	LRR
Sinapis alba	KAF8097942.1	RLK	Other-RLK
Sinapis alba	KAF8098018.1	RLK	LRR
Sinapis alba	KAF8098153.1	RLK	LRR
Sinapis alba	KAF8098160.1	RLK	Other-RLK
Sinapis alba	KAF8098161.1	RLK	Other-RLK
Sinapis alba	KAF8098163.1	RLK	Other-RLK
Sinapis alba	KAF8098298.1	RLK	Other-RLK
Sinapis alba	KAF8098353.1	RLK	LRR
Sinapis alba	KAF8098386.1	RLK	LRR
Sinapis alba	KAF8098420.1	RLK	Other-RLK
Sinapis alba	KAF8098421.1	RLK	Other-RLK
Sinapis alba	KAF8098493.1	RLK	LRR
Sinapis alba	KAF8098522.1	RLK	LRR
Sinapis alba	KAF8098541.1	RLK	Other-RLK
Sinapis alba	KAF8098633.1	RLK	Other-RLK
Sinapis alba	KAF8098634.1	RLK	Other-RLK
Sinapis alba	KAF8098635.1	RLK	Other-RLK
Sinapis alba	KAF8098763.1	RLK	Other-RLK
Sinapis alba	KAF8099085.1	RLK	Other-RLK
Sinapis alba	KAF8099107.1	RLK	LRR
Sinapis alba	KAF8099237.1	RLK	LRR
Sinapis alba	KAF8099515.1	RLK	LRR
Sinapis alba	KAF8099526.1	RLK	LRR
Sinapis alba	KAF8099679.1	RLK	LRR

Sinapis alba	KAF8099804.1	RLK	Other-RLK
Sinapis alba	KAF8099882.1	RLK	Other-RLK
Sinapis alba	KAF8099903.1	RLK	Other-RLK
Sinapis alba	KAF8099968.1	RLK	LRR
Sinapis alba	KAF8099989.1	RLK	LRR
Sinapis alba	KAF8100021.1	RLK	Other-RLK
Sinapis alba	KAF8100024.1	RLK	Other-RLK
Sinapis alba	KAF8100059.1	RLK	Other-RLK
Sinapis alba	KAF8100158.1	RLK	Other-RLK
Sinapis alba	KAF8100177.1	RLK	Other-RLK
Sinapis alba	KAF8100237.1	RLK	LRR
Sinapis alba	KAF8100324.1	RLK	LRR
Sinapis alba	KAF8100325.1	RLK	LRR
Sinapis alba	KAF8100387.1	RLK	Other-RLK
Sinapis alba	KAF8100418.1	RLK	Other-RLK
Sinapis alba	KAF8100419.1	RLK	Other-RLK
Sinapis alba	KAF8100420.1	RLK	Other-RLK
Sinapis alba	KAF8100421.1	RLK	Other-RLK
Sinapis alba	KAF8100595.1	RLK	LRR
Sinapis alba	KAF8100770.1	RLK	LRR
Sinapis alba	KAF8100914.1	RLK	Other-RLK
Sinapis alba	KAF8100968.1	RLK	LRR
Sinapis alba	KAF8101064.1	RLK	LRR
Sinapis alba	KAF8101130.1	RLK	LRR
Sinapis alba	KAF8101333.1	RLK	LRR
Sinapis alba	KAF8101342.1	RLK	Other-RLK
Sinapis alba	KAF8101370.1	RLK	Other-RLK
Sinapis alba	KAF8101603.1	RLK	Other-RLK
Sinapis alba	KAF8101620.1	RLK	LRR
Sinapis alba	KAF8101733.1	RLK	LRR
Sinapis alba	KAF8101829.1	RLK	LRR
Sinapis alba	KAF8101884.1	RLK	Other-RLK
Sinapis alba	KAF8102006.1	RLK	Other-RLK
Sinapis alba	KAF8102016.1	RLK	Other-RLK
Sinapis alba	KAF8102026.1	RLK	LRR
Sinapis alba	KAF8102049.1	RLK	LRR
Sinapis alba	KAF8102167.1	RLK	LRR
Sinapis alba	KAF8102232.1	RLK	Other-RLK
Sinapis alba	KAF8102251.1	RLK	Other-RLK
Sinapis alba	KAF8102253.1	RLK	Other-RLK
Sinapis alba	KAF8102262.1	RLK	LRR
Sinapis alba	KAF8102363.1	RLK	Other-RLK
Sinapis alba	KAF8102366.1	RLK	Other-RLK
Sinapis alba	KAF8102367.1	RLK	Other-RLK
Sinapis alba	KAF8102368.1	RLK	Other-RLK
Sinapis alba	KAF8102369.1	RLK	Other-RLK
Sinapis alba	KAF8102371.1	RLK	Other-RLK
Sinapis alba	KAF8102372.1	RLK	Other-RLK
Sinapis alba	KAF8102375.1	RLK	Other-RLK
Sinapis alba	KAF8102413.1	RLK	Other-RLK
Sinapis alba	KAF8102414.1	RLK	LRR
Sinapis alba	KAF8102417.1	RLK	LRR
Sinapis alba	KAF8102418.1	RLK	LRR
Sinapis alba	KAF8102461.1	RLK	Other-RLK
Sinapis alba	KAF8102505.1	RLK	Other-RLK
Sinapis alba	KAF8102520.1	RLK	Other-RLK
Sinapis alba	KAF8102646.1	RLK	Other-RLK
Sinapis alba	KAF8102730.1	RLK	Other-RLK
Sinapis alba	KAF8102742.1	RLK	LRR
Sinapis alba	KAF8102832.1	RLK	LRR
Sinapis alba	KAF8102855.1	RLK	LRR
Sinapis alba	KAF8102857.1	RLK	Other-RLK

Sinapis alba	KAF8102880.1	RLK	Other-RLK
Sinapis alba	KAF8103088.1	RLK	Other-RLK
Sinapis alba	KAF8103167.1	RLK	Other-RLK
Sinapis alba	KAF8103307.1	RLK	LRR
Sinapis alba	KAF8103396.1	RLK	Other-RLK
Sinapis alba	KAF8103426.1	RLK	Other-RLK
Sinapis alba	KAF8103475.1	RLK	LRR
Sinapis alba	KAF8103512.1	RLK	Other-RLK
Sinapis alba	KAF8103635.1	RLK	Other-RLK
Sinapis alba	KAF8103755.1	RLK	LRR
Sinapis alba	KAF8103763.1	RLK	Other-RLK
Sinapis alba	KAF8103768.1	RLK	Other-RLK
Sinapis alba	KAF8103875.1	RLK	Other-RLK
Sinapis alba	KAF8104200.1	RLK	Other-RLK
Sinapis alba	KAF8104201.1	RLK	Other-RLK
Sinapis alba	KAF8104232.1	RLK	Other-RLK
Sinapis alba	KAF8104790.1	RLK	Other-RLK
Sinapis alba	KAF8105097.1	RLK	Other-RLK
Sinapis alba	KAF8105106.1	RLK	Other-RLK
Sinapis alba	KAF8105394.1	RLK	Other-RLK
Sinapis alba	KAF8105546.1	RLK	Other-RLK
Sinapis alba	KAF8105612.1	RLK	LRR
Sinapis alba	KAF8105627.1	RLK	LRR
Sinapis alba	KAF8105656.1	RLK	LRR
Sinapis alba	KAF8105748.1	RLK	LRR
Sinapis alba	KAF8105807.1	RLK	Other-RLK
Sinapis alba	KAF8106008.1	RLK	Other-RLK
Sinapis alba	KAF8106009.1	RLK	Other-RLK
Sinapis alba	KAF8106010.1	RLK	Other-RLK
Sinapis alba	KAF8106039.1	RLK	LRR
Sinapis alba	KAF8106057.1	RLK	Other-RLK
Sinapis alba	KAF8106109.1	RLK	Other-RLK
Sinapis alba	KAF8106180.1	RLK	Other-RLK
Sinapis alba	KAF8106284.1	RLK	LRR
Sinapis alba	KAF8106291.1	RLK	Other-RLK
Sinapis alba	KAF8106351.1	RLK	Other-RLK
Sinapis alba	KAF8106352.1	RLK	Other-RLK
Sinapis alba	KAF8106353.1	RLK	Other-RLK
Sinapis alba	KAF8106413.1	RLK	LRR
Sinapis alba	KAF8106532.1	RLK	Other-RLK
Sinapis alba	KAF8106731.1	RLK	LRR
Sinapis alba	KAF8106732.1	RLK	LRR
Sinapis alba	KAF8107083.1	RLK	Other-RLK
Sinapis alba	KAF8107113.1	RLK	Other-RLK
Sinapis alba	KAF8107252.1	RLK	LRR
Sinapis alba	KAF8107291.1	RLK	Other-RLK
Sinapis alba	KAF8107361.1	RLK	Other-RLK
Sinapis alba	KAF8107373.1	RLK	Other-RLK
Sinapis alba	KAF8107482.1	RLK	Other-RLK
Sinapis alba	KAF8107514.1	RLK	Other-RLK
Sinapis alba	KAF8107515.1	RLK	Other-RLK
Sinapis alba	KAF8107552.1	RLK	Other-RLK
Sinapis alba	KAF8107793.1	RLK	LRR
Sinapis alba	KAF8107794.1	RLK	LRR
Sinapis alba	KAF8107795.1	RLK	LRR
Sinapis alba	KAF8107798.1	RLK	LRR
Sinapis alba	KAF8107801.1	RLK	LRR
Sinapis alba	KAF8107802.1	RLK	LRR
Sinapis alba	KAF8107870.1	RLK	LRR
Sinapis alba	KAF8107970.1	RLK	LRR
Sinapis alba	KAF8107985.1	RLK	Other-RLK
Sinapis alba	KAF8108036.1	RLK	LRR

Sinapis alba	KAF8108076.1	RLK	Other-RLK
Sinapis alba	KAF8108138.1	RLK	Other-RLK
Sinapis alba	KAF8108175.1	RLK	Other-RLK
Sinapis alba	KAF8108219.1	RLK	Other-RLK
Sinapis alba	KAF8108296.1	RLK	Other-RLK
Sinapis alba	KAF8108311.1	RLK	Other-RLK
Sinapis alba	KAF8108504.1	RLK	Other-RLK
Sinapis alba	KAF8108582.1	RLK	Other-RLK
Sinapis alba	KAF8108583.1	RLK	LRR
Sinapis alba	KAF8108584.1	RLK	LRR
Sinapis alba	KAF8108623.1	RLK	LRR
Sinapis alba	KAF8108642.1	RLK	Other-RLK
Sinapis alba	KAF8108643.1	RLK	Other-RLK
Sinapis alba	KAF8108666.1	RLK	Other-RLK
Sinapis alba	KAF8108670.1	RLK	Other-RLK
Sinapis alba	KAF8108671.1	RLK	Other-RLK
Sinapis alba	KAF8108800.1	RLK	LRR
Sinapis alba	KAF8108838.1	RLK	Lysm
Sinapis alba	KAF8108950.1	RLK	LRR
Sinapis alba	KAF8109116.1	RLK	LRR
Sinapis alba	KAF8109131.1	RLK	LRR
Sinapis alba	KAF8109411.1	RLK	Other-RLK
Sinapis alba	KAF8109435.1	RLK	LRR
Sinapis alba	KAF8109558.1	RLK	Other-RLK
Sinapis alba	KAF8109680.1	RLK	Other-RLK
Sinapis alba	KAF8109690.1	RLK	LRR
Sinapis alba	KAF8109708.1	RLK	Other-RLK
Sinapis alba	KAF8109709.1	RLK	Other-RLK
Sinapis alba	KAF8109710.1	RLK	Other-RLK
Sinapis alba	KAF8109716.1	RLK	Other-RLK
Sinapis alba	KAF8109717.1	RLK	Other-RLK
Sinapis alba	KAF8109718.1	RLK	Other-RLK
Sinapis alba	KAF8109719.1	RLK	Other-RLK
Sinapis alba	KAF8109720.1	RLK	Other-RLK
Sinapis alba	KAF8109721.1	RLK	Other-RLK
Sinapis alba	KAF8109723.1	RLK	Other-RLK
Sinapis alba	KAF8109725.1	RLK	LRR
Sinapis alba	KAF8109991.1	RLK	Other-RLK
Sinapis alba	KAF8110100.1	RLK	Other-RLK
Sinapis alba	KAF8110216.1	RLK	Other-RLK
Sinapis alba	KAF8110217.1	RLK	Other-RLK
Sinapis alba	KAF8110281.1	RLK	LRR
Sinapis alba	KAF8110282.1	RLK	Other-RLK
Sinapis alba	KAF8110333.1	RLK	Other-RLK
Sinapis alba	KAF8110428.1	RLK	Other-RLK
Sinapis alba	KAF8110430.1	RLK	Other-RLK
Sinapis alba	KAF8110466.1	RLK	LRR
Sinapis alba	KAF8110467.1	RLK	LRR
Sinapis alba	KAF8110468.1	RLK	LRR
Sinapis alba	KAF8110503.1	RLK	LRR
Sinapis alba	KAF8110552.1	RLK	Other-RLK
Sinapis alba	KAF8110589.1	RLK	Other-RLK
Sinapis alba	KAF8110694.1	RLK	Other-RLK
Sinapis alba	KAF8110715.1	RLK	Other-RLK
Sinapis alba	KAF8110728.1	RLK	Other-RLK
Sinapis alba	KAF8110815.1	RLK	Other-RLK
Sinapis alba	KAF8110824.1	RLK	Other-RLK
Sinapis alba	KAF8110825.1	RLK	Other-RLK
Sinapis alba	KAF8110834.1	RLK	LRR
Sinapis alba	KAF8110835.1	RLK	LRR
Sinapis alba	KAF8110885.1	RLK	Other-RLK
Sinapis alba	KAF8110886.1	RLK	Other-RLK

Sinapis alba	KAF8110904.1	RLK	Other-RLK
Sinapis alba	KAF8110905.1	RLK	Other-RLK
Sinapis alba	KAF8110913.1	RLK	Other-RLK
Sinapis alba	KAF8111008.1	RLK	LRR
Sinapis alba	KAF8111056.1	RLK	Other-RLK
Sinapis alba	KAF8111059.1	RLK	LRR
Sinapis alba	KAF8111067.1	RLK	Other-RLK
Sinapis alba	KAF8111068.1	RLK	Other-RLK
Sinapis alba	KAF8111069.1	RLK	Other-RLK
Sinapis alba	KAF8111133.1	RLK	LRR
Sinapis alba	KAF8111143.1	RLK	LRR
Sinapis alba	KAF8111193.1	RLK	Other-RLK
Sinapis alba	KAF8111194.1	RLK	Other-RLK
Sinapis alba	KAF8111199.1	RLK	LRR
Sinapis alba	KAF8111233.1	RLK	LRR
Sinapis alba	KAF8111343.1	RLK	Other-RLK
Sinapis alba	KAF8111400.1	RLK	LRR
Sinapis alba	KAF8111519.1	RLK	Other-RLK
Sinapis alba	KAF8111930.1	RLK	LRR
Sinapis alba	KAF8111972.1	RLK	Other-RLK
Sinapis alba	KAF8112138.1	RLK	Other-RLK
Sinapis alba	KAF8112144.1	RLK	Other-RLK
Sinapis alba	KAF8112169.1	RLK	Other-RLK
Sinapis alba	KAF8112283.1	RLK	Other-RLK
Sinapis alba	KAF8112288.1	RLK	Other-RLK
Sinapis alba	KAF8112314.1	RLK	LRR
Sinapis alba	KAF8112364.1	RLK	Other-RLK
Sinapis alba	KAF8112365.1	RLK	Other-RLK
Sinapis alba	KAF8112520.1	RLK	LRR
Sinapis alba	KAF8112521.1	RLK	LRR
Sinapis alba	KAF8112522.1	RLK	LRR
Sinapis alba	KAF8112523.1	RLK	LRR
Sinapis alba	KAF8112524.1	RLK	LRR
Sinapis alba	KAF8112525.1	RLK	LRR
Sinapis alba	KAF8112589.1	RLK	LRR
Sinapis alba	KAF8112629.1	RLK	LRR
Sinapis alba	KAF8112630.1	RLK	LRR
Sinapis alba	KAF8112671.1	RLK	Other-RLK
Sinapis alba	KAF8112741.1	RLK	LRR
Sinapis alba	KAF8112805.1	RLK	LRR
Sinapis alba	KAF8112824.1	RLK	Other-RLK
Sinapis alba	KAF8112826.1	RLK	Other-RLK
Sinapis alba	KAF8112827.1	RLK	Other-RLK
Sinapis alba	KAF8112831.1	RLK	Other-RLK
Sinapis alba	KAF8112832.1	RLK	Other-RLK
Sinapis alba	KAF8112874.1	RLK	Other-RLK
Sinapis alba	KAF8112887.1	RLK	LRR
Sinapis alba	KAF8113004.1	RLK	Other-RLK
Sinapis alba	KAF8113020.1	RLK	Other-RLK
Sinapis alba	KAF8113236.1	RLK	Other-RLK
Sinapis alba	KAF8113278.1	RLK	LRR
Sinapis alba	KAF8113281.1	RLK	LRR
Sinapis alba	KAF8113366.1	RLK	Other-RLK
Sinapis alba	KAF8113367.1	RLK	Other-RLK
Sinapis alba	KAF8113369.1	RLK	Other-RLK
Sinapis alba	KAF8113411.1	RLK	LRR
Sinapis alba	KAF8113426.1	RLK	LRR
Sinapis alba	KAF8113479.1	RLK	Other-RLK
Sinapis alba	KAF8113640.1	RLK	LRR
Sinapis alba	KAF8113646.1	RLK	Other-RLK
Sinapis alba	KAF8113647.1	RLK	Other-RLK
Sinapis alba	KAF8113648.1	RLK	Other-RLK

Sinapis alba	KAF8113662.1	RLK	Other-RLK
Sinapis alba	KAF8113664.1	RLK	Other-RLK
Sinapis alba	KAF8113665.1	RLK	Other-RLK
Sinapis alba	KAF8113752.1	RLK	LRR
Sinapis alba	KAF8113892.1	RLK	LRR
Sinapis alba	KAF8113956.1	RLK	LRR
Sinapis alba	KAF8114145.1	RLK	Other-RLK
Sinapis alba	KAF8114153.1	RLK	Other-RLK
Sinapis alba	KAF8114156.1	RLK	Other-RLK
Sinapis alba	KAF8114158.1	RLK	Other-RLK
Sinapis alba	KAF8114159.1	RLK	Other-RLK
Sinapis alba	KAF8114160.1	RLK	Other-RLK
Sinapis alba	KAF8114161.1	RLK	Other-RLK
Sinapis alba	KAF8114223.1	RLK	LRR
Sinapis alba	KAF8114284.1	RLK	Other-RLK
Sinapis alba	KAF8114311.1	RLK	Other-RLK
Sinapis alba	KAF8114365.1	RLK	Other-RLK
Sinapis alba	KAF8114366.1	RLK	Other-RLK
Sinapis alba	KAF8114468.1	RLK	Other-RLK
Sinapis alba	KAF8114470.1	RLK	LRR
Sinapis alba	KAF8114482.1	RLK	LRR
Sinapis alba	KAF8114591.1	RLK	Other-RLK
Sinapis alba	KAF8114592.1	RLK	Other-RLK
Sinapis alba	KAF8114698.1	RLK	Other-RLK
Sinapis alba	KAF8114832.1	RLK	Other-RLK
Sinapis alba	KAF8114849.1	RLK	LRR
Sinapis alba	KAF8114962.1	RLK	LRR
Sinapis alba	KAF8114983.1	RLK	LRR
Sinapis alba	KAF8115004.1	RLK	Other-RLK
Sinapis alba	KAF8115005.1	RLK	Other-RLK
Sinapis alba	KAF8115044.1	RLK	LRR
Sinapis alba	KAF8115114.1	RLK	Other-RLK
Sinapis alba	KAF8115135.1	RLK	Other-RLK
Sinapis alba	KAF8115137.1	RLK	Other-RLK
Sinapis alba	KAF8115215.1	RLK	LRR
Sinapis alba	KAF8115369.1	RLK	LRR
Sinapis alba	KAF8115536.1	RLK	LRR
Sinapis alba	KAF8115543.1	RLK	LRR
Sinapis alba	KAF8115552.1	RLK	Other-RLK
Sinapis alba	KAF8115579.1	RLK	LRR
Sinapis alba	KAF8115589.1	RLK	LRR
Sinapis alba	KAF8115591.1	RLK	LRR
Sinapis alba	KAF8115597.1	RLK	Other-RLK
Sinapis alba	KAF8115598.1	RLK	Other-RLK
Sinapis alba	KAF8115764.1	RLK	LRR
Sinapis alba	KAF8115808.1	RLK	Other-RLK
Sinapis alba	KAF8115809.1	RLK	Other-RLK
Sinapis alba	KAF8115842.1	RLK	Other-RLK
Sinapis alba	KAF8115934.1	RLK	Other-RLK
Sinapis alba	KAF8116034.1	RLK	Other-RLK
Sinapis alba	KAF8116265.1	RLK	LRR
Sinapis alba	KAF8116327.1	RLK	LRR
Sinapis alba	KAF8116439.1	RLK	LRR
Sinapis alba	KAF8116499.1	RLK	Other-RLK
Sinapis alba	KAF8116518.1	RLK	Other-RLK
Sinapis alba	KAF8116519.1	RLK	Other-RLK
Sinapis alba	KAF8116524.1	RLK	Other-RLK
Sinapis alba	KAF8116725.1	RLK	LRR
Sinapis alba	KAF8116755.1	RLK	Other-RLK
Sinapis alba	KAF8116823.1	RLK	LRR
Sinapis alba	KAF8116864.1	RLK	Other-RLK
Sinapis alba	KAF8116874.1	RLK	Other-RLK

Sinapis alba	KAF8116875.1	RLK	Other-RLK
Sinapis alba	KAF8116968.1	RLK	LRR
Sinapis alba	KAF8117029.1	RLK	LRR
Sinapis alba	KAF8117050.1	RLK	LRR
Sinapis alba	KAF8117201.1	RLK	LRR
Sinapis alba	KAF8117233.1	RLK	LRR
Sinapis alba	KAF8117234.1	RLK	Other-RLK
Sinapis alba	KAF8117308.1	RLK	Other-RLK
Sinapis alba	KAF8117492.1	RLK	LRR
Sinapis alba	KAF8117505.1	RLK	LRR
Sinapis alba	KAF8117524.1	RLK	LRR
Sinapis alba	KAF8117549.1	RLK	LRR
Sinapis alba	KAF8117626.1	RLK	LRR
Sinapis alba	KAF8117650.1	RLK	LRR
Sinapis alba	KAF8117974.1	RLK	LRR
Sinapis alba	KAF8118001.1	RLK	LRR
Sinapis alba	KAF8118080.1	RLK	LRR
Sinapis alba	KAF8118138.1	RLK	Other-RLK
Sinapis alba	KAF8118183.1	RLK	Other-RLK
Sinapis alba	KAF8118264.1	RLK	Other-RLK
Sinapis alba	KAF8118269.1	RLK	LRR
Sinapis alba	KAF8118306.1	RLK	Other-RLK
Sinapis alba	KAF8118601.1	RLK	Other-RLK
Sinapis alba	KAF8118613.1	RLK	LRR
Sinapis alba	KAF8118614.1	RLK	LRR
Sinapis alba	KAF8118617.1	RLK	Other-RLK
Sinapis alba	KAF8118619.1	RLK	Other-RLK
Sinapis alba	KAF8118621.1	RLK	LRR
Sinapis alba	KAF8118721.1	RLK	Other-RLK
Sinapis alba	KAF8118755.1	RLK	LRR
Sinapis alba	KAF8118756.1	RLK	LRR
Sinapis alba	KAF8118919.1	RLK	Other-RLK
Sinapis alba	KAF8118948.1	RLK	LRR
Sinapis alba	KAF8119012.1	RLK	Other-RLK
Sinapis alba	KAF8119053.1	RLK	LRR
Sinapis alba	KAF8119059.1	RLK	Other-RLK
Sinapis alba	KAF8119066.1	RLK	Other-RLK
Sinapis alba	KAF8119120.1	RLK	Other-RLK
Sinapis alba	KAF8044821.1	RLP	LRR
Sinapis alba	KAF8045120.1	RLP	LRR
Sinapis alba	KAF8045221.1	RLP	LRR
Sinapis alba	KAF8045493.1	RLP	LRR
Sinapis alba	KAF8045895.1	RLP	LRR
Sinapis alba	KAF8046449.1	RLP	LRR
Sinapis alba	KAF8046956.1	RLP	Lysm
Sinapis alba	KAF8047904.1	RLP	LRR
Sinapis alba	KAF8048753.1	RLP	LRR
Sinapis alba	KAF8048754.1	RLP	LRR
Sinapis alba	KAF8049633.1	RLP	LRR
Sinapis alba	KAF8049889.1	RLP	LRR
Sinapis alba	KAF8050784.1	RLP	LRR
Sinapis alba	KAF8051500.1	RLP	LRR
Sinapis alba	KAF8051803.1	RLP	LRR
Sinapis alba	KAF8052573.1	RLP	LRR
Sinapis alba	KAF8053316.1	RLP	LRR
Sinapis alba	KAF8053593.1	RLP	LRR
Sinapis alba	KAF8053603.1	RLP	LRR
Sinapis alba	KAF8054047.1	RLP	LRR
Sinapis alba	KAF8054888.1	RLP	LRR
Sinapis alba	KAF8055604.1	RLP	LRR
Sinapis alba	KAF8062126.1	RLP	LRR
Sinapis alba	KAF8067433.1	RLP	LRR

Sinapis alba	KAF8068486.1	RLP	LRR
Sinapis alba	KAF8074312.1	RLP	LRR
Sinapis alba	KAF8074427.1	RLP	LRR
Sinapis alba	KAF8074431.1	RLP	LRR
Sinapis alba	KAF8074432.1	RLP	LRR
Sinapis alba	KAF8079461.1	RLP	LRR
Sinapis alba	KAF8079511.1	RLP	LRR
Sinapis alba	KAF8079512.1	RLP	LRR
Sinapis alba	KAF8080090.1	RLP	LRR
Sinapis alba	KAF8080091.1	RLP	LRR
Sinapis alba	KAF8080217.1	RLP	LRR
Sinapis alba	KAF8080653.1	RLP	LRR
Sinapis alba	KAF8080806.1	RLP	LRR
Sinapis alba	KAF8080834.1	RLP	LRR
Sinapis alba	KAF8081125.1	RLP	LRR
Sinapis alba	KAF8082066.1	RLP	LRR
Sinapis alba	KAF8083537.1	RLP	LRR
Sinapis alba	KAF8083882.1	RLP	LRR
Sinapis alba	KAF8084401.1	RLP	LRR
Sinapis alba	KAF8085518.1	RLP	LRR
Sinapis alba	KAF8085519.1	RLP	LRR
Sinapis alba	KAF8085554.1	RLP	LRR
Sinapis alba	KAF8085575.1	RLP	LRR
Sinapis alba	KAF8085576.1	RLP	LRR
Sinapis alba	KAF8086480.1	RLP	Lysm
Sinapis alba	KAF8086610.1	RLP	LRR
Sinapis alba	KAF8087431.1	RLP	LRR
Sinapis alba	KAF8088459.1	RLP	Lysm
Sinapis alba	KAF8088559.1	RLP	LRR
Sinapis alba	KAF8089464.1	RLP	LRR
Sinapis alba	KAF8090082.1	RLP	LRR
Sinapis alba	KAF8090138.1	RLP	LRR
Sinapis alba	KAF8090145.1	RLP	LRR
Sinapis alba	KAF8092002.1	RLP	LRR
Sinapis alba	KAF8092574.1	RLP	LRR
Sinapis alba	KAF8092581.1	RLP	LRR
Sinapis alba	KAF8092938.1	RLP	LRR
Sinapis alba	KAF8093213.1	RLP	LRR
Sinapis alba	KAF8095127.1	RLP	LRR
Sinapis alba	KAF8095496.1	RLP	LRR
Sinapis alba	KAF8096085.1	RLP	LRR
Sinapis alba	KAF8096090.1	RLP	LRR
Sinapis alba	KAF8096091.1	RLP	LRR
Sinapis alba	KAF8096567.1	RLP	LRR
Sinapis alba	KAF8096659.1	RLP	LRR
Sinapis alba	KAF8097181.1	RLP	LRR
Sinapis alba	KAF8097453.1	RLP	LRR
Sinapis alba	KAF8097590.1	RLP	LRR
Sinapis alba	KAF8097591.1	RLP	LRR
Sinapis alba	KAF8097592.1	RLP	LRR
Sinapis alba	KAF8097915.1	RLP	LRR
Sinapis alba	KAF8098072.1	RLP	LRR
Sinapis alba	KAF8098726.1	RLP	LRR
Sinapis alba	KAF8099627.1	RLP	LRR
Sinapis alba	KAF8099659.1	RLP	LRR
Sinapis alba	KAF8099869.1	RLP	LRR
Sinapis alba	KAF8100137.1	RLP	LRR
Sinapis alba	KAF8100413.1	RLP	LRR
Sinapis alba	KAF8100435.1	RLP	LRR
Sinapis alba	KAF8100623.1	RLP	LRR
Sinapis alba	KAF8100624.1	RLP	LRR
Sinapis alba	KAF8100917.1	RLP	LRR

Sinapis alba	KAF8100970.1	RLP	LRR
Sinapis alba	KAF8101499.1	RLP	LRR
Sinapis alba	KAF8102001.1	RLP	LRR
Sinapis alba	KAF8102695.1	RLP	LRR
Sinapis alba	KAF8103832.1	RLP	LRR
Sinapis alba	KAF8103883.1	RLP	LRR
Sinapis alba	KAF8104053.1	RLP	LRR
Sinapis alba	KAF8104264.1	RLP	LRR
Sinapis alba	KAF8104450.1	RLP	LRR
Sinapis alba	KAF8104908.1	RLP	LRR
Sinapis alba	KAF8105233.1	RLP	LRR
Sinapis alba	KAF8105234.1	RLP	LRR
Sinapis alba	KAF8105293.1	RLP	LRR
Sinapis alba	KAF8105661.1	RLP	LRR
Sinapis alba	KAF8106027.1	RLP	LRR
Sinapis alba	KAF8106071.1	RLP	LRR
Sinapis alba	KAF8106130.1	RLP	LRR
Sinapis alba	KAF8106283.1	RLP	LRR
Sinapis alba	KAF8106473.1	RLP	LRR
Sinapis alba	KAF8106575.1	RLP	LRR
Sinapis alba	KAF8107503.1	RLP	LRR
Sinapis alba	KAF8108000.1	RLP	LRR
Sinapis alba	KAF8108070.1	RLP	LRR
Sinapis alba	KAF8108121.1	RLP	LRR
Sinapis alba	KAF8108771.1	RLP	LRR
Sinapis alba	KAF8108772.1	RLP	LRR
Sinapis alba	KAF8108774.1	RLP	LRR
Sinapis alba	KAF8109087.1	RLP	LRR
Sinapis alba	KAF8109279.1	RLP	LRR
Sinapis alba	KAF8109281.1	RLP	LRR
Sinapis alba	KAF8109282.1	RLP	LRR
Sinapis alba	KAF8111633.1	RLP	LRR
Sinapis alba	KAF8112028.1	RLP	LRR
Sinapis alba	KAF8112777.1	RLP	LRR
Sinapis alba	KAF8112779.1	RLP	LRR
Sinapis alba	KAF8113251.1	RLP	LRR
Sinapis alba	KAF8114266.1	RLP	LRR
Sinapis alba	KAF8114475.1	RLP	LRR
Sinapis alba	KAF8115111.1	RLP	LRR
Sinapis alba	KAF8116764.1	RLP	LRR
Sinapis alba	KAF8117198.1	RLP	LRR
Sinapis alba	KAF8117199.1	RLP	LRR
Sinapis alba	KAF8117940.1	RLP	LRR
Sinapis alba	KAF8118680.1	RLP	LRR
Sinapis alba	KAF8119041.1	RLP	LRR
Capsella bursa-pastoris	Cbp19577	NLR	CN
Capsella bursa-pastoris	Cbp47816	NLR	CN
Capsella bursa-pastoris	Cbp51319	NLR	CN
Capsella bursa-pastoris	Cbp52173	NLR	CN
Capsella bursa-pastoris	Cbp17689	NLR	CN
Capsella bursa-pastoris	Cbp47817	NLR	CN
Capsella bursa-pastoris	Cbp45485	NLR	CN
Capsella bursa-pastoris	Cbp13279	NLR	CN
Capsella bursa-pastoris	Cbp33711	NLR	CNL
Capsella bursa-pastoris	Cbp46917	NLR	CNL
Capsella bursa-pastoris	Cbp19028	NLR	CNL
Capsella bursa-pastoris	Cbp32207	NLR	CNL
Capsella bursa-pastoris	Cbp12124	NLR	CNL
Capsella bursa-pastoris	Cbp14225	NLR	CNL
Capsella bursa-pastoris	Cbp19189	NLR	CNL
Capsella bursa-pastoris	Cbp45469	NLR	CNL
Capsella bursa-pastoris	Cbp33577	NLR	CNL

Capsella bursa-pastoris	Cbp47695	NLR	CNL
Capsella bursa-pastoris	Cbp32219	NLR	CNL
Capsella bursa-pastoris	Cbp21377	NLR	CNL
Capsella bursa-pastoris	Cbp34553	NLR	CNL
Capsella bursa-pastoris	Cbp11464	NLR	CNL
Capsella bursa-pastoris	Cbp44162	NLR	CNL
Capsella bursa-pastoris	Cbp18997	NLR	CNL
Capsella bursa-pastoris	Cbp33511	NLR	CNL
Capsella bursa-pastoris	Cbp26414	NLR	CNL
Capsella bursa-pastoris	Cbp16103	NLR	CNL
Capsella bursa-pastoris	Cbp23231	NLR	CNL
Capsella bursa-pastoris	Cbp51187	NLR	CNL
Capsella bursa-pastoris	Cbp25969	NLR	CNL
Capsella bursa-pastoris	Cbp33720	NLR	CNL
Capsella bursa-pastoris	Cbp8106	NLR	CNL
Capsella bursa-pastoris	Cbp53365	NLR	CNL
Capsella bursa-pastoris	Cbp34552	NLR	CNL
Capsella bursa-pastoris	Cbp811	NLR	CNL
Capsella bursa-pastoris	Cbp28795	NLR	CNL
Capsella bursa-pastoris	Cbp52108	NLR	CNL
Capsella bursa-pastoris	Cbp41750	NLR	CNL
Capsella bursa-pastoris	Cbp50135	NLR	CNL
Capsella bursa-pastoris	Cbp45470	NLR	CNL
Capsella bursa-pastoris	Cbp46346	NLR	CNL
Capsella bursa-pastoris	Cbp18467	NLR	CNL
Capsella bursa-pastoris	Cbp33852	NLR	CNL
Capsella bursa-pastoris	Cbp47693	NLR	CNL
Capsella bursa-pastoris	Cbp53094	NLR	CNL
Capsella bursa-pastoris	Cbp52166	NLR	CNL
Capsella bursa-pastoris	Cbp42070	NLR	CNL
Capsella bursa-pastoris	Cbp51318	NLR	CNL
Capsella bursa-pastoris	Cbp48114	NLR	CNL
Capsella bursa-pastoris	Cbp51186	NLR	CNL
Capsella bursa-pastoris	Cbp15059	NLR	CNL
Capsella bursa-pastoris	Cbp21382	NLR	CNL
Capsella bursa-pastoris	Cbp13283	NLR	NBS
Capsella bursa-pastoris	Cbp32957	NLR	NBS
Capsella bursa-pastoris	Cbp19079	NLR	NBS
Capsella bursa-pastoris	Cbp6846	NLR	NBS
Capsella bursa-pastoris	Cbp50076	NLR	NBS
Capsella bursa-pastoris	Cbp41284	NLR	NBS
Capsella bursa-pastoris	Cbp4034	NLR	NBS
Capsella bursa-pastoris	Cbp51754	NLR	NBS
Capsella bursa-pastoris	Cbp50281	NLR	NBS
Capsella bursa-pastoris	Cbp26897	NLR	NBS
Capsella bursa-pastoris	Cbp45467	NLR	NBS
Capsella bursa-pastoris	Cbp46264	NLR	NBS
Capsella bursa-pastoris	Cbp25157	NLR	NBS
Capsella bursa-pastoris	Cbp32205	NLR	NBS
Capsella bursa-pastoris	Cbp13280	NLR	NBS
Capsella bursa-pastoris	Cbp4866	NLR	NBS
Capsella bursa-pastoris	Cbp45910	NLR	NL
Capsella bursa-pastoris	Cbp5868	NLR	NL
Capsella bursa-pastoris	Cbp519	NLR	NL
Capsella bursa-pastoris	Cbp47529	NLR	NL
Capsella bursa-pastoris	Cbp48437	NLR	NL
Capsella bursa-pastoris	Cbp32218	NLR	NL
Capsella bursa-pastoris	Cbp46128	NLR	NL
Capsella bursa-pastoris	Cbp14253	NLR	NL
Capsella bursa-pastoris	Cbp11721	NLR	NL
Capsella bursa-pastoris	Cbp25968	NLR	NL
Capsella bursa-pastoris	Cbp34754	NLR	NL

Capsella bursa-pastoris	Cbp31703	NLR	NL
Capsella bursa-pastoris	Cbp50136	NLR	NL
Capsella bursa-pastoris	Cbp37986	NLR	NL
Capsella bursa-pastoris	Cbp38087	NLR	NL
Capsella bursa-pastoris	Cbp19029	NLR	NL
Capsella bursa-pastoris	Cbp39607	NLR	NL
Capsella bursa-pastoris	Cbp17170	NLR	NL
Capsella bursa-pastoris	Cbp5768	NLR	NL
Capsella bursa-pastoris	Cbp53366	NLR	NL
Capsella bursa-pastoris	Cbp46916	NLR	NL
Capsella bursa-pastoris	Cbp33710	NLR	NL
Capsella bursa-pastoris	Cbp22113	NLR	NL
Capsella bursa-pastoris	Cbp36244	NLR	NL
Capsella bursa-pastoris	Cbp19041	NLR	NL
Capsella bursa-pastoris	Cbp16342	NLR	NL
Capsella bursa-pastoris	Cbp38086	NLR	NL
Capsella bursa-pastoris	Cbp19051	NLR	NL
Capsella bursa-pastoris	Cbp29059	NLR	NL
Capsella bursa-pastoris	Cbp46906	NLR	NL
Capsella bursa-pastoris	Cbp47136	NLR	NL
Capsella bursa-pastoris	Cbp52531	NLR	NL
Capsella bursa-pastoris	Cbp19065	NLR	NL
Capsella bursa-pastoris	Cbp47963	NLR	NL
Capsella bursa-pastoris	Cbp6345	NLR	NL
Capsella bursa-pastoris	Cbp11946	NLR	NL
Capsella bursa-pastoris	Cbp19077	NLR	NL
Capsella bursa-pastoris	Cbp22745	NLR	NL
Capsella bursa-pastoris	Cbp16664	NLR	NL
Capsella bursa-pastoris	Cbp19042	NLR	NL
Capsella bursa-pastoris	Cbp52986	NLR	NL
Capsella bursa-pastoris	Cbp13284	NLR	NL
Capsella bursa-pastoris	Cbp33712	NLR	NL
Capsella bursa-pastoris	Cbp38624	NLR	NL
Capsella bursa-pastoris	Cbp28794	NLR	NL
Capsella bursa-pastoris	Cbp34664	NLR	NL
Capsella bursa-pastoris	Cbp520	NLR	NL
Capsella bursa-pastoris	Cbp45534	NLR	NL
Capsella bursa-pastoris	Cbp21935	NLR	NL
Capsella bursa-pastoris	Cbp33161	NLR	NL
Capsella bursa-pastoris	Cbp51741	NLR	NL
Capsella bursa-pastoris	Cbp33510	NLR	NL
Capsella bursa-pastoris	Cbp39399	NLR	NL
Capsella bursa-pastoris	Cbp11530	NLR	NL
Capsella bursa-pastoris	Cbp45210	NLR	NL
Capsella bursa-pastoris	Cbp39590	NLR	NL
Capsella bursa-pastoris	Cbp45504	NLR	NL
Capsella bursa-pastoris	Cbp19083	NLR	NL
Capsella bursa-pastoris	Cbp32206	NLR	NL
Capsella bursa-pastoris	Cbp50214	NLR	Other-NLR
Capsella bursa-pastoris	Cbp23672	NLR	Other-NLR
Capsella bursa-pastoris	Cbp26886	NLR	Other-NLR
Capsella bursa-pastoris	Cbp9266	NLR	Other-NLR
Capsella bursa-pastoris	Cbp45547	NLR	Other-NLR
Capsella bursa-pastoris	Cbp47127	NLR	Other-NLR
Capsella bursa-pastoris	Cbp19429	NLR	Other-NLR
Capsella bursa-pastoris	Cbp33444	NLR	Other-NLR
Capsella bursa-pastoris	Cbp11461	NLR	Other-NLR
Capsella bursa-pastoris	Cbp45486	NLR	Other-NLR
Capsella bursa-pastoris	Cbp11526	NLR	Other-NLR
Capsella bursa-pastoris	Cbp30474	NLR	Other-NLR
Capsella bursa-pastoris	Cbp20142	NLR	Other-NLR
Capsella bursa-pastoris	Cbp33422	NLR	Other-NLR

Capsella bursa-pastoris	Cbp45500	NLR	Other-NLR
Capsella bursa-pastoris	Cbp33421	NLR	Other-NLR
Capsella bursa-pastoris	Cbp33448	NLR	Other-NLR
Capsella bursa-pastoris	Cbp44277	NLR	Other-NLR
Capsella bursa-pastoris	Cbp26840	NLR	Other-NLR
Capsella bursa-pastoris	Cbp44279	NLR	Other-NLR
Capsella bursa-pastoris	Cbp13417	NLR	Other-NLR
Capsella bursa-pastoris	Cbp9004	NLR	TN
Capsella bursa-pastoris	Cbp31978	NLR	TN
Capsella bursa-pastoris	Cbp52558	NLR	TN
Capsella bursa-pastoris	Cbp42205	NLR	TN
Capsella bursa-pastoris	Cbp24034	NLR	TN
Capsella bursa-pastoris	Cbp23373	NLR	TN
Capsella bursa-pastoris	Cbp22904	NLR	TN
Capsella bursa-pastoris	Cbp50210	NLR	TN
Capsella bursa-pastoris	Cbp47128	NLR	TN
Capsella bursa-pastoris	Cbp25833	NLR	TN
Capsella bursa-pastoris	Cbp3036	NLR	TN
Capsella bursa-pastoris	Cbp11459	NLR	TN
Capsella bursa-pastoris	Cbp47129	NLR	TN
Capsella bursa-pastoris	Cbp50211	NLR	TN
Capsella bursa-pastoris	Cbp42207	NLR	TN
Capsella bursa-pastoris	Cbp27624	NLR	TN
Capsella bursa-pastoris	Cbp42203	NLR	TN
Capsella bursa-pastoris	Cbp19430	NLR	TN
Capsella bursa-pastoris	Cbp47132	NLR	TN
Capsella bursa-pastoris	Cbp9005	NLR	TN
Capsella bursa-pastoris	Cbp8736	NLR	TN
Capsella bursa-pastoris	Cbp13366	NLR	TN
Capsella bursa-pastoris	Cbp16373	NLR	TNL
Capsella bursa-pastoris	Cbp26994	NLR	TNL
Capsella bursa-pastoris	Cbp52774	NLR	TNL
Capsella bursa-pastoris	Cbp12293	NLR	TNL
Capsella bursa-pastoris	Cbp11117	NLR	TNL
Capsella bursa-pastoris	Cbp9219	NLR	TNL
Capsella bursa-pastoris	Cbp7304	NLR	TNL
Capsella bursa-pastoris	Cbp13412	NLR	TNL
Capsella bursa-pastoris	Cbp13253	NLR	TNL
Capsella bursa-pastoris	Cbp49916	NLR	TNL
Capsella bursa-pastoris	Cbp49921	NLR	TNL
Capsella bursa-pastoris	Cbp4127	NLR	TNL
Capsella bursa-pastoris	Cbp26881	NLR	TNL
Capsella bursa-pastoris	Cbp2498	NLR	TNL
Capsella bursa-pastoris	Cbp34461	NLR	TNL
Capsella bursa-pastoris	Cbp10968	NLR	TNL
Capsella bursa-pastoris	Cbp49446	NLR	TNL
Capsella bursa-pastoris	Cbp34462	NLR	TNL
Capsella bursa-pastoris	Cbp45487	NLR	TNL
Capsella bursa-pastoris	Cbp38995	NLR	TNL
Capsella bursa-pastoris	Cbp39406	NLR	TNL
Capsella bursa-pastoris	Cbp13411	NLR	TNL
Capsella bursa-pastoris	Cbp11062	NLR	TNL
Capsella bursa-pastoris	Cbp26883	NLR	TNL
Capsella bursa-pastoris	Cbp44935	NLR	TNL
Capsella bursa-pastoris	Cbp26861	NLR	TNL
Capsella bursa-pastoris	Cbp52775	NLR	TNL
Capsella bursa-pastoris	Cbp11061	NLR	TNL
Capsella bursa-pastoris	Cbp19204	NLR	TNL
Capsella bursa-pastoris	Cbp13132	NLR	TNL
Capsella bursa-pastoris	Cbp29527	NLR	TNL
Capsella bursa-pastoris	Cbp48441	NLR	TNL
Capsella bursa-pastoris	Cbp12814	NLR	TNL

Capsella bursa-pastoris	Cbp14179	NLR	TNL
Capsella bursa-pastoris	Cbp14210	NLR	TNL
Capsella bursa-pastoris	Cbp20745	NLR	TNL
Capsella bursa-pastoris	Cbp27058	NLR	TNL
Capsella bursa-pastoris	Cbp11527	NLR	TNL
Capsella bursa-pastoris	Cbp9265	NLR	TNL
Capsella bursa-pastoris	Cbp19432	NLR	TNL
Capsella bursa-pastoris	Cbp10076	NLR	TNL
Capsella bursa-pastoris	Cbp49899	NLR	TNL
Capsella bursa-pastoris	Cbp45533	NLR	TNL
Capsella bursa-pastoris	Cbp19206	NLR	TNL
Capsella bursa-pastoris	Cbp13410	NLR	TNL
Capsella bursa-pastoris	Cbp45527	NLR	TNL
Capsella bursa-pastoris	Cbp43581	NLR	TNL
Capsella bursa-pastoris	Cbp9286	NLR	TNL
Capsella bursa-pastoris	Cbp50075	NLR	TNL
Capsella bursa-pastoris	Cbp50215	NLR	TNL
Capsella bursa-pastoris	Cbp36884	NLR	TNL
Capsella bursa-pastoris	Cbp26447	NLR	TNL
Capsella bursa-pastoris	Cbp13367	NLR	TNL
Capsella bursa-pastoris	Cbp27040	NLR	TNL
Capsella bursa-pastoris	Cbp34753	NLR	TNL
Capsella bursa-pastoris	Cbp26884	NLR	TNL
Capsella bursa-pastoris	Cbp27017	NLR	TNL
Capsella bursa-pastoris	Cbp26860	NLR	TNL
Capsella bursa-pastoris	Cbp11528	NLR	TNL
Capsella bursa-pastoris	Cbp7866	NLR	TNL
Capsella bursa-pastoris	Cbp45728	NLR	TNL
Capsella bursa-pastoris	Cbp49279	NLR	TNL
Capsella bursa-pastoris	Cbp47917	NLR	TNL
Capsella bursa-pastoris	Cbp30618	NLR	TNL
Capsella bursa-pastoris	Cbp19202	NLR	TNL
Capsella bursa-pastoris	Cbp3371	NLR	TNL
Capsella bursa-pastoris	Cbp7305	NLR	TNL
Capsella bursa-pastoris	Cbp9251	NLR	TNL
Capsella bursa-pastoris	Cbp11468	NLR	TNL
Capsella bursa-pastoris	Cbp4476	NLR	TNL
Capsella bursa-pastoris	Cbp8082	NLR	TNL
Capsella bursa-pastoris	Cbp9970	NLR	TNL
Capsella bursa-pastoris	Cbp10000	NLR	TNL
Capsella bursa-pastoris	Cbp9263	NLR	TNL
Capsella bursa-pastoris	Cbp34706	NLR	TNL
Capsella bursa-pastoris	Cbp19163	NLR	TNL
Capsella bursa-pastoris	Cbp19971	NLR	TNL
Capsella bursa-pastoris	Cbp11064	NLR	TNL
Capsella bursa-pastoris	Cbp47916	NLR	TNL
Capsella bursa-pastoris	Cbp48776	NLR	TNL
Capsella bursa-pastoris	Cbp11460	NLR	TNL
Capsella bursa-pastoris	Cbp24904	NLR	TNL
Capsella bursa-pastoris	Cbp19164	NLR	TNL
Capsella bursa-pastoris	Cbp36899	NLR	TNL
Capsella bursa-pastoris	Cbp49914	NLR	TNL
Capsella bursa-pastoris	Cbp21984	NLR	TNL
Capsella bursa-pastoris	Cbp51486	NLR	TNL
Capsella bursa-pastoris	Cbp3372	NLR	TNL
Capsella bursa-pastoris	Cbp39404	NLR	TNL
Capsella bursa-pastoris	Cbp46000	NLR	TNL
Capsella bursa-pastoris	Cbp35079	NLR	TNL
Capsella bursa-pastoris	Cbp2517	NLR	TNL
Capsella bursa-pastoris	Cbp27012	NLR	TNL
Capsella bursa-pastoris	Cbp51101	NLR	TNL
Capsella bursa-pastoris	Cbp42255	NLR	TNL

Capsella bursa-pastoris	Cbp19203	NLR	TNL
Capsella bursa-pastoris	Cbp48777	NLR	TNL
Capsella bursa-pastoris	Cbp45499	NLR	TNL
Capsella bursa-pastoris	Cbp12437	NLR	TNL
Capsella bursa-pastoris	Cbp33418	NLR	TNL
Capsella bursa-pastoris	Cbp49922	NLR	TNL
Capsella bursa-pastoris	Cbp34755	NLR	TNL
Capsella bursa-pastoris	Cbp26887	NLR	TNL
Capsella bursa-pastoris	Cbp1593	NLR	TNL
Capsella bursa-pastoris	Cbp1642	NLR	TNL
Capsella bursa-pastoris	Cbp33420	NLR	TNL
Capsella bursa-pastoris	Cbp52985	NLR	TNL
Capsella bursa-pastoris	Cbp26865	NLR	TX
Capsella bursa-pastoris	Cbp45498	NLR	TX
Capsella bursa-pastoris	Cbp44288	NLR	TX
Capsella bursa-pastoris	Cbp46734	NLR	TX
Capsella bursa-pastoris	Cbp35994	NLR	TX
Capsella bursa-pastoris	Cbp16413	NLR	TX
Capsella bursa-pastoris	Cbp39986	NLR	TX
Capsella bursa-pastoris	Cbp44287	NLR	TX
Capsella bursa-pastoris	Cbp44283	NLR	TX
Capsella bursa-pastoris	Cbp46730	NLR	TX
Capsella bursa-pastoris	Cbp44284	NLR	TX
Capsella bursa-pastoris	Cbp34955	NLR	TX
Capsella bursa-pastoris	Cbp25840	NLR	TX
Capsella bursa-pastoris	Cbp26866	NLR	TX
Capsella bursa-pastoris	Cbp22180	NLR	TX
Capsella bursa-pastoris	Cbp1671	NLR	TX
Capsella bursa-pastoris	Cbp44278	NLR	TX
Capsella bursa-pastoris	Cbp8728	NLR	TX
Capsella bursa-pastoris	Cbp41699	NLR	TX
Capsella bursa-pastoris	Cbp45523	NLR	TX
Capsella bursa-pastoris	Cbp13408	NLR	TX
Capsella bursa-pastoris	Cbp45520	NLR	TX
Capsella bursa-pastoris	Cbp26898	NLR	TX
Capsella bursa-pastoris	Cbp26864	NLR	TX
Capsella bursa-pastoris	Cbp13338	NLR	TX
Capsella bursa-pastoris	Cbp26841	NLR	TX
Capsella bursa-pastoris	Cbp45529	NLR	TX
Capsella bursa-pastoris	Cbp48438	NLR	TX
Capsella bursa-pastoris	Cbp47135	NLR	TX
Capsella bursa-pastoris	Cbp46731	NLR	TX
Capsella bursa-pastoris	Cbp26882	NLR	TX
Capsella bursa-pastoris	Cbp9003	NLR	TX
Capsella bursa-pastoris	Cbp25967	NLR	TX
Capsella bursa-pastoris	Cbp45484	NLR	TX
Capsella bursa-pastoris	Cbp13418	NLR	TX
Capsella bursa-pastoris	Cbp9006	NLR	TX
Capsella bursa-pastoris	Cbp13336	NLR	TX
Capsella bursa-pastoris	Cbp1643	NLR	TX
Capsella bursa-pastoris	Cbp8727	NLR	TX
Capsella bursa-pastoris	Cbp9007	NLR	TX
Capsella bursa-pastoris	Cbp45046	NLR	TX
Capsella bursa-pastoris	Cbp52902	NLR	TX
Capsella bursa-pastoris	Cbp45521	NLR	TX
Capsella bursa-pastoris	Cbp25841	NLR	TX
Capsella bursa-pastoris	Cbp33699	NLR	TX
Capsella bursa-pastoris	Cbp46928	NLR	TX
Capsella bursa-pastoris	Cbp33449	NLR	TX
Capsella bursa-pastoris	Cbp46736	NLR	TX
Capsella bursa-pastoris	Cbp46301	NLR	TX
Capsella bursa-pastoris	Cbp46732	NLR	TX

Capsella bursa-pastoris	Cbp44281	NLR	TX
Capsella bursa-pastoris	Cbp44286	NLR	TX
Capsella bursa-pastoris	Cbp32710	NLR	TX
Capsella bursa-pastoris	Cbp39398	NLR	TX
Capsella bursa-pastoris	Cbp33451	NLR	TX
Capsella bursa-pastoris	Cbp18474	NLR	TX
Capsella bursa-pastoris	Cbp39109	NLR	TX
Capsella bursa-pastoris	Cbp9252	NLR	TX
Capsella bursa-pastoris	Cbp14326	NLR	TX
Capsella bursa-pastoris	Cbp46733	NLR	TX
Capsella bursa-pastoris	Cbp8729	NLR	TX
Capsella bursa-pastoris	Cbp32921	NLR	TX
Capsella bursa-pastoris	Cbp8893	NLR	TX
Capsella bursa-pastoris	Cbp16414	NLR	TX
Capsella bursa-pastoris	Cbp45525	NLR	TX
Capsella bursa-pastoris	Cbp46737	NLR	TX
Capsella bursa-pastoris	Cbp45530	NLR	TX
Capsella bursa-pastoris	Cbp44282	NLR	TX
Capsella bursa-pastoris	Cbp33445	NLR	TX
Capsella bursa-pastoris	Cbp26888	NLR	TX
Capsella bursa-pastoris	Cbp4608	NLR	TX
Capsella bursa-pastoris	Cbp20141	NLR	TX
Capsella bursa-pastoris	Cbp42204	NLR	TX
Capsella bursa-pastoris	Cbp22181	NLR	TX
Capsella bursa-pastoris	Cbp27954	NLR	TX
Capsella bursa-pastoris	Cbp45524	NLR	TX
Capsella bursa-pastoris	Cbp10021	RLK	LRR
Capsella bursa-pastoris	Cbp10081	RLK	LRR
Capsella bursa-pastoris	Cbp10104	RLK	LRR
Capsella bursa-pastoris	Cbp10122	RLK	LRR
Capsella bursa-pastoris	Cbp10139	RLK	Lysm
Capsella bursa-pastoris	Cbp10167	RLK	Other-RLK
Capsella bursa-pastoris	Cbp10218	RLK	LRR
Capsella bursa-pastoris	Cbp10282	RLK	LRR
Capsella bursa-pastoris	Cbp10649	RLK	Other-RLK
Capsella bursa-pastoris	Cbp10679	RLK	Other-RLK
Capsella bursa-pastoris	Cbp10686	RLK	Other-RLK
Capsella bursa-pastoris	Cbp10688	RLK	LRR
Capsella bursa-pastoris	Cbp10723	RLK	Other-RLK
Capsella bursa-pastoris	Cbp10791	RLK	Other-RLK
Capsella bursa-pastoris	Cbp10858	RLK	LRR
Capsella bursa-pastoris	Cbp1094	RLK	Other-RLK
Capsella bursa-pastoris	Cbp11040	RLK	LRR
Capsella bursa-pastoris	Cbp11126	RLK	Other-RLK
Capsella bursa-pastoris	Cbp11226	RLK	Other-RLK
Capsella bursa-pastoris	Cbp11252	RLK	Other-RLK
Capsella bursa-pastoris	Cbp1129	RLK	LRR
Capsella bursa-pastoris	Cbp11317	RLK	Other-RLK
Capsella bursa-pastoris	Cbp11357	RLK	Other-RLK
Capsella bursa-pastoris	Cbp11359	RLK	Other-RLK
Capsella bursa-pastoris	Cbp11374	RLK	Other-RLK
Capsella bursa-pastoris	Cbp116	RLK	LRR
Capsella bursa-pastoris	Cbp11612	RLK	Other-RLK
Capsella bursa-pastoris	Cbp11614	RLK	Other-RLK
Capsella bursa-pastoris	Cbp11615	RLK	Other-RLK
Capsella bursa-pastoris	Cbp11616	RLK	Other-RLK
Capsella bursa-pastoris	Cbp11659	RLK	Other-RLK
Capsella bursa-pastoris	Cbp11660	RLK	Other-RLK
Capsella bursa-pastoris	Cbp11706	RLK	Other-RLK
Capsella bursa-pastoris	Cbp11710	RLK	LRR
Capsella bursa-pastoris	Cbp11712	RLK	LRR
Capsella bursa-pastoris	Cbp11713	RLK	Other-RLK

Capsella bursa-pastoris	Cbp11769	RLK	LRR
Capsella bursa-pastoris	Cbp11798	RLK	LRR
Capsella bursa-pastoris	Cbp11800	RLK	LRR
Capsella bursa-pastoris	Cbp11801	RLK	LRR
Capsella bursa-pastoris	Cbp12005	RLK	LRR
Capsella bursa-pastoris	Cbp1204	RLK	Other-RLK
Capsella bursa-pastoris	Cbp12040	RLK	Other-RLK
Capsella bursa-pastoris	Cbp12059	RLK	LRR
Capsella bursa-pastoris	Cbp1210	RLK	Other-RLK
Capsella bursa-pastoris	Cbp1211	RLK	Other-RLK
Capsella bursa-pastoris	Cbp1212	RLK	Other-RLK
Capsella bursa-pastoris	Cbp12172	RLK	Other-RLK
Capsella bursa-pastoris	Cbp12185	RLK	LRR
Capsella bursa-pastoris	Cbp12446	RLK	Other-RLK
Capsella bursa-pastoris	Cbp12447	RLK	Other-RLK
Capsella bursa-pastoris	Cbp1246	RLK	Other-RLK
Capsella bursa-pastoris	Cbp12496	RLK	Other-RLK
Capsella bursa-pastoris	Cbp12609	RLK	Other-RLK
Capsella bursa-pastoris	Cbp12626	RLK	LRR
Capsella bursa-pastoris	Cbp1269	RLK	LRR
Capsella bursa-pastoris	Cbp12713	RLK	Other-RLK
Capsella bursa-pastoris	Cbp12807	RLK	LRR
Capsella bursa-pastoris	Cbp12878	RLK	LRR
Capsella bursa-pastoris	Cbp12883	RLK	Other-RLK
Capsella bursa-pastoris	Cbp1290	RLK	Other-RLK
Capsella bursa-pastoris	Cbp12946	RLK	Other-RLK
Capsella bursa-pastoris	Cbp13044	RLK	Other-RLK
Capsella bursa-pastoris	Cbp13171	RLK	Other-RLK
Capsella bursa-pastoris	Cbp13180	RLK	LRR
Capsella bursa-pastoris	Cbp13318	RLK	Other-RLK
Capsella bursa-pastoris	Cbp13378	RLK	Other-RLK
Capsella bursa-pastoris	Cbp13489	RLK	Other-RLK
Capsella bursa-pastoris	Cbp13589	RLK	Other-RLK
Capsella bursa-pastoris	Cbp13711	RLK	LRR
Capsella bursa-pastoris	Cbp13720	RLK	Other-RLK
Capsella bursa-pastoris	Cbp13722	RLK	Other-RLK
Capsella bursa-pastoris	Cbp13751	RLK	LRR
Capsella bursa-pastoris	Cbp13789	RLK	Other-RLK
Capsella bursa-pastoris	Cbp13820	RLK	Other-RLK
Capsella bursa-pastoris	Cbp13885	RLK	Other-RLK
Capsella bursa-pastoris	Cbp13969	RLK	LRR
Capsella bursa-pastoris	Cbp14006	RLK	LRR
Capsella bursa-pastoris	Cbp14007	RLK	LRR
Capsella bursa-pastoris	Cbp1409	RLK	Other-RLK
Capsella bursa-pastoris	Cbp14140	RLK	LRR
Capsella bursa-pastoris	Cbp14207	RLK	Other-RLK
Capsella bursa-pastoris	Cbp14212	RLK	LRR
Capsella bursa-pastoris	Cbp1432	RLK	Other-RLK
Capsella bursa-pastoris	Cbp14427	RLK	LRR
Capsella bursa-pastoris	Cbp14454	RLK	LRR
Capsella bursa-pastoris	Cbp14461	RLK	Other-RLK
Capsella bursa-pastoris	Cbp14471	RLK	Other-RLK
Capsella bursa-pastoris	Cbp14518	RLK	Other-RLK
Capsella bursa-pastoris	Cbp1459	RLK	Other-RLK
Capsella bursa-pastoris	Cbp14633	RLK	LRR
Capsella bursa-pastoris	Cbp14650	RLK	LRR
Capsella bursa-pastoris	Cbp1477	RLK	Other-RLK
Capsella bursa-pastoris	Cbp1478	RLK	Other-RLK
Capsella bursa-pastoris	Cbp1479	RLK	Other-RLK
Capsella bursa-pastoris	Cbp1480	RLK	Other-RLK
Capsella bursa-pastoris	Cbp1481	RLK	Other-RLK
Capsella bursa-pastoris	Cbp1482	RLK	Other-RLK

Capsella bursa-pastoris	Cbp1485	RLK	Other-RLK
Capsella bursa-pastoris	Cbp1486	RLK	Other-RLK
Capsella bursa-pastoris	Cbp1487	RLK	Other-RLK
Capsella bursa-pastoris	Cbp1488	RLK	Other-RLK
Capsella bursa-pastoris	Cbp1489	RLK	Other-RLK
Capsella bursa-pastoris	Cbp14892	RLK	Other-RLK
Capsella bursa-pastoris	Cbp14995	RLK	Other-RLK
Capsella bursa-pastoris	Cbp15039	RLK	Other-RLK
Capsella bursa-pastoris	Cbp15040	RLK	Other-RLK
Capsella bursa-pastoris	Cbp15058	RLK	Other-RLK
Capsella bursa-pastoris	Cbp15219	RLK	Other-RLK
Capsella bursa-pastoris	Cbp15427	RLK	Other-RLK
Capsella bursa-pastoris	Cbp15461	RLK	Other-RLK
Capsella bursa-pastoris	Cbp15487	RLK	LRR
Capsella bursa-pastoris	Cbp15624	RLK	LRR
Capsella bursa-pastoris	Cbp15710	RLK	LRR
Capsella bursa-pastoris	Cbp15738	RLK	Other-RLK
Capsella bursa-pastoris	Cbp1604	RLK	Other-RLK
Capsella bursa-pastoris	Cbp1605	RLK	Other-RLK
Capsella bursa-pastoris	Cbp16052	RLK	Other-RLK
Capsella bursa-pastoris	Cbp16115	RLK	Other-RLK
Capsella bursa-pastoris	Cbp16116	RLK	Other-RLK
Capsella bursa-pastoris	Cbp16117	RLK	Other-RLK
Capsella bursa-pastoris	Cbp16130	RLK	Other-RLK
Capsella bursa-pastoris	Cbp16263	RLK	Other-RLK
Capsella bursa-pastoris	Cbp16300	RLK	LRR
Capsella bursa-pastoris	Cbp16305	RLK	Other-RLK
Capsella bursa-pastoris	Cbp16496	RLK	Other-RLK
Capsella bursa-pastoris	Cbp165	RLK	LRR
Capsella bursa-pastoris	Cbp1651	RLK	Other-RLK
Capsella bursa-pastoris	Cbp1652	RLK	Other-RLK
Capsella bursa-pastoris	Cbp1655	RLK	Other-RLK
Capsella bursa-pastoris	Cbp16622	RLK	LRR
Capsella bursa-pastoris	Cbp16810	RLK	Other-RLK
Capsella bursa-pastoris	Cbp16811	RLK	Other-RLK
Capsella bursa-pastoris	Cbp16813	RLK	Other-RLK
Capsella bursa-pastoris	Cbp17212	RLK	LRR
Capsella bursa-pastoris	Cbp17348	RLK	Other-RLK
Capsella bursa-pastoris	Cbp17356	RLK	LRR
Capsella bursa-pastoris	Cbp17369	RLK	LRR
Capsella bursa-pastoris	Cbp17370	RLK	LRR
Capsella bursa-pastoris	Cbp17381	RLK	Other-RLK
Capsella bursa-pastoris	Cbp17386	RLK	LRR
Capsella bursa-pastoris	Cbp17397	RLK	LRR
Capsella bursa-pastoris	Cbp17427	RLK	Other-RLK
Capsella bursa-pastoris	Cbp17510	RLK	LRR
Capsella bursa-pastoris	Cbp17726	RLK	Other-RLK
Capsella bursa-pastoris	Cbp17786	RLK	Other-RLK
Capsella bursa-pastoris	Cbp17936	RLK	LRR
Capsella bursa-pastoris	Cbp18066	RLK	Other-RLK
Capsella bursa-pastoris	Cbp18087	RLK	LRR
Capsella bursa-pastoris	Cbp18264	RLK	Other-RLK
Capsella bursa-pastoris	Cbp18353	RLK	LRR
Capsella bursa-pastoris	Cbp18355	RLK	Other-RLK
Capsella bursa-pastoris	Cbp1836	RLK	LRR
Capsella bursa-pastoris	Cbp18362	RLK	Other-RLK
Capsella bursa-pastoris	Cbp18394	RLK	Other-RLK
Capsella bursa-pastoris	Cbp18547	RLK	Other-RLK
Capsella bursa-pastoris	Cbp18549	RLK	Other-RLK
Capsella bursa-pastoris	Cbp18595	RLK	LRR
Capsella bursa-pastoris	Cbp18613	RLK	LRR
Capsella bursa-pastoris	Cbp18614	RLK	LRR

Capsella bursa-pastoris	Cbp18714	RLK	LRR
Capsella bursa-pastoris	Cbp18738	RLK	LRR
Capsella bursa-pastoris	Cbp18745	RLK	Other-RLK
Capsella bursa-pastoris	Cbp18755	RLK	Other-RLK
Capsella bursa-pastoris	Cbp18924	RLK	LRR
Capsella bursa-pastoris	Cbp18943	RLK	Other-RLK
Capsella bursa-pastoris	Cbp18973	RLK	Other-RLK
Capsella bursa-pastoris	Cbp19117	RLK	Other-RLK
Capsella bursa-pastoris	Cbp19174	RLK	Other-RLK
Capsella bursa-pastoris	Cbp19243	RLK	LRR
Capsella bursa-pastoris	Cbp1926	RLK	Other-RLK
Capsella bursa-pastoris	Cbp19268	RLK	LRR
Capsella bursa-pastoris	Cbp19332	RLK	LRR
Capsella bursa-pastoris	Cbp19342	RLK	Other-RLK
Capsella bursa-pastoris	Cbp19459	RLK	LRR
Capsella bursa-pastoris	Cbp19503	RLK	LRR
Capsella bursa-pastoris	Cbp19547	RLK	Other-RLK
Capsella bursa-pastoris	Cbp19637	RLK	Other-RLK
Capsella bursa-pastoris	Cbp19642	RLK	Other-RLK
Capsella bursa-pastoris	Cbp19643	RLK	Other-RLK
Capsella bursa-pastoris	Cbp19780	RLK	LRR
Capsella bursa-pastoris	Cbp19878	RLK	LRR
Capsella bursa-pastoris	Cbp19964	RLK	LRR
Capsella bursa-pastoris	Cbp20139	RLK	LRR
Capsella bursa-pastoris	Cbp20232	RLK	LRR
Capsella bursa-pastoris	Cbp2052	RLK	LRR
Capsella bursa-pastoris	Cbp20587	RLK	LRR
Capsella bursa-pastoris	Cbp20620	RLK	LRR
Capsella bursa-pastoris	Cbp20654	RLK	LRR
Capsella bursa-pastoris	Cbp20890	RLK	Other-RLK
Capsella bursa-pastoris	Cbp20914	RLK	Other-RLK
Capsella bursa-pastoris	Cbp20915	RLK	Other-RLK
Capsella bursa-pastoris	Cbp20939	RLK	LRR
Capsella bursa-pastoris	Cbp21084	RLK	LRR
Capsella bursa-pastoris	Cbp21226	RLK	LRR
Capsella bursa-pastoris	Cbp2151	RLK	LRR
Capsella bursa-pastoris	Cbp21514	RLK	LRR
Capsella bursa-pastoris	Cbp21603	RLK	LRR
Capsella bursa-pastoris	Cbp21604	RLK	LRR
Capsella bursa-pastoris	Cbp21605	RLK	LRR
Capsella bursa-pastoris	Cbp21653	RLK	Other-RLK
Capsella bursa-pastoris	Cbp21682	RLK	Other-RLK
Capsella bursa-pastoris	Cbp21768	RLK	LRR
Capsella bursa-pastoris	Cbp21919	RLK	LRR
Capsella bursa-pastoris	Cbp22009	RLK	Other-RLK
Capsella bursa-pastoris	Cbp22033	RLK	Other-RLK
Capsella bursa-pastoris	Cbp22074	RLK	LRR
Capsella bursa-pastoris	Cbp22078	RLK	Other-RLK
Capsella bursa-pastoris	Cbp22202	RLK	LRR
Capsella bursa-pastoris	Cbp22231	RLK	Other-RLK
Capsella bursa-pastoris	Cbp22239	RLK	LRR
Capsella bursa-pastoris	Cbp22252	RLK	Other-RLK
Capsella bursa-pastoris	Cbp22339	RLK	Other-RLK
Capsella bursa-pastoris	Cbp22340	RLK	Other-RLK
Capsella bursa-pastoris	Cbp22341	RLK	Other-RLK
Capsella bursa-pastoris	Cbp22342	RLK	Other-RLK
Capsella bursa-pastoris	Cbp22397	RLK	Other-RLK
Capsella bursa-pastoris	Cbp22398	RLK	LRR
Capsella bursa-pastoris	Cbp22399	RLK	LRR
Capsella bursa-pastoris	Cbp22400	RLK	LRR
Capsella bursa-pastoris	Cbp22456	RLK	Other-RLK
Capsella bursa-pastoris	Cbp22534	RLK	Other-RLK

Capsella bursa-pastoris	Cbp22619	RLK	Other-RLK
Capsella bursa-pastoris	Cbp2262	RLK	Other-RLK
Capsella bursa-pastoris	Cbp22669	RLK	Other-RLK
Capsella bursa-pastoris	Cbp22678	RLK	LRR
Capsella bursa-pastoris	Cbp22814	RLK	Other-RLK
Capsella bursa-pastoris	Cbp22874	RLK	Other-RLK
Capsella bursa-pastoris	Cbp22962	RLK	Other-RLK
Capsella bursa-pastoris	Cbp23106	RLK	LRR
Capsella bursa-pastoris	Cbp23247	RLK	Other-RLK
Capsella bursa-pastoris	Cbp23248	RLK	Other-RLK
Capsella bursa-pastoris	Cbp23249	RLK	Other-RLK
Capsella bursa-pastoris	Cbp23257	RLK	Other-RLK
Capsella bursa-pastoris	Cbp23342	RLK	LRR
Capsella bursa-pastoris	Cbp23367	RLK	Other-RLK
Capsella bursa-pastoris	Cbp23385	RLK	LRR
Capsella bursa-pastoris	Cbp23413	RLK	Other-RLK
Capsella bursa-pastoris	Cbp23431	RLK	Other-RLK
Capsella bursa-pastoris	Cbp23492	RLK	Other-RLK
Capsella bursa-pastoris	Cbp23580	RLK	Other-RLK
Capsella bursa-pastoris	Cbp23611	RLK	LRR
Capsella bursa-pastoris	Cbp23644	RLK	LRR
Capsella bursa-pastoris	Cbp23752	RLK	Other-RLK
Capsella bursa-pastoris	Cbp2376	RLK	Other-RLK
Capsella bursa-pastoris	Cbp23885	RLK	Other-RLK
Capsella bursa-pastoris	Cbp23912	RLK	Other-RLK
Capsella bursa-pastoris	Cbp23973	RLK	Other-RLK
Capsella bursa-pastoris	Cbp2399	RLK	Other-RLK
Capsella bursa-pastoris	Cbp24023	RLK	LRR
Capsella bursa-pastoris	Cbp24040	RLK	Other-RLK
Capsella bursa-pastoris	Cbp24065	RLK	LRR
Capsella bursa-pastoris	Cbp24175	RLK	LRR
Capsella bursa-pastoris	Cbp24192	RLK	LRR
Capsella bursa-pastoris	Cbp24274	RLK	LRR
Capsella bursa-pastoris	Cbp24330	RLK	LRR
Capsella bursa-pastoris	Cbp24393	RLK	Other-RLK
Capsella bursa-pastoris	Cbp24402	RLK	LRR
Capsella bursa-pastoris	Cbp24415	RLK	Other-RLK
Capsella bursa-pastoris	Cbp24442	RLK	Other-RLK
Capsella bursa-pastoris	Cbp24527	RLK	LRR
Capsella bursa-pastoris	Cbp24528	RLK	LRR
Capsella bursa-pastoris	Cbp24531	RLK	LRR
Capsella bursa-pastoris	Cbp24579	RLK	Other-RLK
Capsella bursa-pastoris	Cbp24597	RLK	LRR
Capsella bursa-pastoris	Cbp24659	RLK	LRR
Capsella bursa-pastoris	Cbp24732	RLK	LRR
Capsella bursa-pastoris	Cbp24880	RLK	Other-RLK
Capsella bursa-pastoris	Cbp25037	RLK	LRR
Capsella bursa-pastoris	Cbp2522	RLK	LRR
Capsella bursa-pastoris	Cbp25330	RLK	LRR
Capsella bursa-pastoris	Cbp25386	RLK	Other-RLK
Capsella bursa-pastoris	Cbp2543	RLK	LRR
Capsella bursa-pastoris	Cbp25509	RLK	Other-RLK
Capsella bursa-pastoris	Cbp25582	RLK	LRR
Capsella bursa-pastoris	Cbp25638	RLK	Other-RLK
Capsella bursa-pastoris	Cbp25658	RLK	Other-RLK
Capsella bursa-pastoris	Cbp25664	RLK	LRR
Capsella bursa-pastoris	Cbp25671	RLK	Other-RLK
Capsella bursa-pastoris	Cbp25702	RLK	Other-RLK
Capsella bursa-pastoris	Cbp25766	RLK	LRR
Capsella bursa-pastoris	Cbp2579	RLK	Lysm
Capsella bursa-pastoris	Cbp25808	RLK	LRR
Capsella bursa-pastoris	Cbp25853	RLK	Other-RLK

Capsella bursa-pastoris	Cbp25854	RLK	Other-RLK
Capsella bursa-pastoris	Cbp25855	RLK	Other-RLK
Capsella bursa-pastoris	Cbp25856	RLK	Other-RLK
Capsella bursa-pastoris	Cbp25861	RLK	Other-RLK
Capsella bursa-pastoris	Cbp25862	RLK	Other-RLK
Capsella bursa-pastoris	Cbp25863	RLK	Other-RLK
Capsella bursa-pastoris	Cbp25864	RLK	Other-RLK
Capsella bursa-pastoris	Cbp25865	RLK	Other-RLK
Capsella bursa-pastoris	Cbp25866	RLK	Other-RLK
Capsella bursa-pastoris	Cbp25867	RLK	Other-RLK
Capsella bursa-pastoris	Cbp25868	RLK	Other-RLK
Capsella bursa-pastoris	Cbp25870	RLK	Other-RLK
Capsella bursa-pastoris	Cbp25871	RLK	Other-RLK
Capsella bursa-pastoris	Cbp25872	RLK	Other-RLK
Capsella bursa-pastoris	Cbp25873	RLK	Other-RLK
Capsella bursa-pastoris	Cbp25874	RLK	Other-RLK
Capsella bursa-pastoris	Cbp25903	RLK	Other-RLK
Capsella bursa-pastoris	Cbp26024	RLK	Other-RLK
Capsella bursa-pastoris	Cbp2604	RLK	Other-RLK
Capsella bursa-pastoris	Cbp26176	RLK	Other-RLK
Capsella bursa-pastoris	Cbp2618	RLK	LRR
Capsella bursa-pastoris	Cbp26245	RLK	LRR
Capsella bursa-pastoris	Cbp26280	RLK	LRR
Capsella bursa-pastoris	Cbp2629	RLK	Other-RLK
Capsella bursa-pastoris	Cbp26359	RLK	LRR
Capsella bursa-pastoris	Cbp26476	RLK	Other-RLK
Capsella bursa-pastoris	Cbp26486	RLK	LRR
Capsella bursa-pastoris	Cbp26681	RLK	Other-RLK
Capsella bursa-pastoris	Cbp26691	RLK	LRR
Capsella bursa-pastoris	Cbp26712	RLK	Other-RLK
Capsella bursa-pastoris	Cbp26788	RLK	LRR
Capsella bursa-pastoris	Cbp26790	RLK	LRR
Capsella bursa-pastoris	Cbp26791	RLK	LRR
Capsella bursa-pastoris	Cbp26792	RLK	LRR
Capsella bursa-pastoris	Cbp26824	RLK	LRR
Capsella bursa-pastoris	Cbp26862	RLK	LRR
Capsella bursa-pastoris	Cbp26940	RLK	LRR
Capsella bursa-pastoris	Cbp26943	RLK	LRR
Capsella bursa-pastoris	Cbp26944	RLK	Other-RLK
Capsella bursa-pastoris	Cbp26948	RLK	LRR
Capsella bursa-pastoris	Cbp26974	RLK	Other-RLK
Capsella bursa-pastoris	Cbp26999	RLK	LRR
Capsella bursa-pastoris	Cbp27087	RLK	Other-RLK
Capsella bursa-pastoris	Cbp27290	RLK	LRR
Capsella bursa-pastoris	Cbp27389	RLK	Other-RLK
Capsella bursa-pastoris	Cbp27404	RLK	Other-RLK
Capsella bursa-pastoris	Cbp27413	RLK	LRR
Capsella bursa-pastoris	Cbp27425	RLK	LRR
Capsella bursa-pastoris	Cbp275	RLK	Other-RLK
Capsella bursa-pastoris	Cbp27546	RLK	Other-RLK
Capsella bursa-pastoris	Cbp27639	RLK	Other-RLK
Capsella bursa-pastoris	Cbp27656	RLK	LRR
Capsella bursa-pastoris	Cbp277	RLK	Other-RLK
Capsella bursa-pastoris	Cbp2783	RLK	Other-RLK
Capsella bursa-pastoris	Cbp27892	RLK	Other-RLK
Capsella bursa-pastoris	Cbp27932	RLK	LRR
Capsella bursa-pastoris	Cbp27987	RLK	LRR
Capsella bursa-pastoris	Cbp27992	RLK	Other-RLK
Capsella bursa-pastoris	Cbp28023	RLK	Other-RLK
Capsella bursa-pastoris	Cbp28117	RLK	Other-RLK
Capsella bursa-pastoris	Cbp28227	RLK	Other-RLK
Capsella bursa-pastoris	Cbp28364	RLK	Other-RLK

Capsella bursa-pastoris	Cbp28366	RLK	Other-RLK
Capsella bursa-pastoris	Cbp2838	RLK	LRR
Capsella bursa-pastoris	Cbp28396	RLK	LRR
Capsella bursa-pastoris	Cbp28457	RLK	Other-RLK
Capsella bursa-pastoris	Cbp28469	RLK	Other-RLK
Capsella bursa-pastoris	Cbp2857	RLK	LRR
Capsella bursa-pastoris	Cbp28632	RLK	LRR
Capsella bursa-pastoris	Cbp28784	RLK	Other-RLK
Capsella bursa-pastoris	Cbp28817	RLK	LRR
Capsella bursa-pastoris	Cbp28826	RLK	LRR
Capsella bursa-pastoris	Cbp28880	RLK	LRR
Capsella bursa-pastoris	Cbp28881	RLK	LRR
Capsella bursa-pastoris	Cbp28892	RLK	Other-RLK
Capsella bursa-pastoris	Cbp28893	RLK	Other-RLK
Capsella bursa-pastoris	Cbp28898	RLK	Other-RLK
Capsella bursa-pastoris	Cbp28921	RLK	LRR
Capsella bursa-pastoris	Cbp29033	RLK	Other-RLK
Capsella bursa-pastoris	Cbp29079	RLK	LRR
Capsella bursa-pastoris	Cbp29104	RLK	Other-RLK
Capsella bursa-pastoris	Cbp29208	RLK	Other-RLK
Capsella bursa-pastoris	Cbp29211	RLK	LRR
Capsella bursa-pastoris	Cbp29213	RLK	Other-RLK
Capsella bursa-pastoris	Cbp29240	RLK	Other-RLK
Capsella bursa-pastoris	Cbp29489	RLK	Other-RLK
Capsella bursa-pastoris	Cbp29821	RLK	LRR
Capsella bursa-pastoris	Cbp29842	RLK	Other-RLK
Capsella bursa-pastoris	Cbp29918	RLK	LRR
Capsella bursa-pastoris	Cbp30042	RLK	Other-RLK
Capsella bursa-pastoris	Cbp30058	RLK	Other-RLK
Capsella bursa-pastoris	Cbp30110	RLK	LRR
Capsella bursa-pastoris	Cbp30117	RLK	Other-RLK
Capsella bursa-pastoris	Cbp30121	RLK	Other-RLK
Capsella bursa-pastoris	Cbp30122	RLK	Other-RLK
Capsella bursa-pastoris	Cbp30123	RLK	Other-RLK
Capsella bursa-pastoris	Cbp30130	RLK	Other-RLK
Capsella bursa-pastoris	Cbp30243	RLK	Other-RLK
Capsella bursa-pastoris	Cbp30247	RLK	LRR
Capsella bursa-pastoris	Cbp3027	RLK	Other-RLK
Capsella bursa-pastoris	Cbp30376	RLK	Other-RLK
Capsella bursa-pastoris	Cbp30377	RLK	Other-RLK
Capsella bursa-pastoris	Cbp30378	RLK	Other-RLK
Capsella bursa-pastoris	Cbp30379	RLK	Other-RLK
Capsella bursa-pastoris	Cbp30380	RLK	Other-RLK
Capsella bursa-pastoris	Cbp30413	RLK	LRR
Capsella bursa-pastoris	Cbp30477	RLK	LRR
Capsella bursa-pastoris	Cbp3052	RLK	Other-RLK
Capsella bursa-pastoris	Cbp30557	RLK	Other-RLK
Capsella bursa-pastoris	Cbp30617	RLK	LRR
Capsella bursa-pastoris	Cbp30662	RLK	Other-RLK
Capsella bursa-pastoris	Cbp30838	RLK	Lysm
Capsella bursa-pastoris	Cbp30984	RLK	LRR
Capsella bursa-pastoris	Cbp31061	RLK	LRR
Capsella bursa-pastoris	Cbp31114	RLK	LRR
Capsella bursa-pastoris	Cbp3114	RLK	LRR
Capsella bursa-pastoris	Cbp31202	RLK	LRR
Capsella bursa-pastoris	Cbp31214	RLK	LRR
Capsella bursa-pastoris	Cbp31244	RLK	LRR
Capsella bursa-pastoris	Cbp31255	RLK	Other-RLK
Capsella bursa-pastoris	Cbp31289	RLK	Other-RLK
Capsella bursa-pastoris	Cbp31291	RLK	LRR
Capsella bursa-pastoris	Cbp31306	RLK	LRR
Capsella bursa-pastoris	Cbp31324	RLK	Other-RLK

Capsella bursa-pastoris	Cbp3137	RLK	LRR
Capsella bursa-pastoris	Cbp31430	RLK	LRR
Capsella bursa-pastoris	Cbp31492	RLK	Other-RLK
Capsella bursa-pastoris	Cbp31583	RLK	Other-RLK
Capsella bursa-pastoris	Cbp31597	RLK	LRR
Capsella bursa-pastoris	Cbp31617	RLK	Other-RLK
Capsella bursa-pastoris	Cbp31758	RLK	Other-RLK
Capsella bursa-pastoris	Cbp31767	RLK	LRR
Capsella bursa-pastoris	Cbp31780	RLK	LRR
Capsella bursa-pastoris	Cbp31898	RLK	Other-RLK
Capsella bursa-pastoris	Cbp31994	RLK	LRR
Capsella bursa-pastoris	Cbp31996	RLK	LRR
Capsella bursa-pastoris	Cbp32039	RLK	Other-RLK
Capsella bursa-pastoris	Cbp32040	RLK	Other-RLK
Capsella bursa-pastoris	Cbp32041	RLK	Other-RLK
Capsella bursa-pastoris	Cbp32042	RLK	Other-RLK
Capsella bursa-pastoris	Cbp32045	RLK	Other-RLK
Capsella bursa-pastoris	Cbp32075	RLK	Other-RLK
Capsella bursa-pastoris	Cbp32272	RLK	LRR
Capsella bursa-pastoris	Cbp32344	RLK	Other-RLK
Capsella bursa-pastoris	Cbp32345	RLK	Other-RLK
Capsella bursa-pastoris	Cbp32346	RLK	Other-RLK
Capsella bursa-pastoris	Cbp32347	RLK	Other-RLK
Capsella bursa-pastoris	Cbp3235	RLK	LRR
Capsella bursa-pastoris	Cbp32396	RLK	LRR
Capsella bursa-pastoris	Cbp32414	RLK	LRR
Capsella bursa-pastoris	Cbp32422	RLK	LRR
Capsella bursa-pastoris	Cbp32514	RLK	Other-RLK
Capsella bursa-pastoris	Cbp32571	RLK	LRR
Capsella bursa-pastoris	Cbp32712	RLK	LRR
Capsella bursa-pastoris	Cbp32778	RLK	Other-RLK
Capsella bursa-pastoris	Cbp32988	RLK	Other-RLK
Capsella bursa-pastoris	Cbp33018	RLK	Lysm
Capsella bursa-pastoris	Cbp33023	RLK	LRR
Capsella bursa-pastoris	Cbp33024	RLK	LRR
Capsella bursa-pastoris	Cbp33025	RLK	LRR
Capsella bursa-pastoris	Cbp33026	RLK	LRR
Capsella bursa-pastoris	Cbp33028	RLK	LRR
Capsella bursa-pastoris	Cbp33029	RLK	LRR
Capsella bursa-pastoris	Cbp33118	RLK	Other-RLK
Capsella bursa-pastoris	Cbp33126	RLK	LRR
Capsella bursa-pastoris	Cbp33194	RLK	Other-RLK
Capsella bursa-pastoris	Cbp33234	RLK	Other-RLK
Capsella bursa-pastoris	Cbp3325	RLK	LRR
Capsella bursa-pastoris	Cbp33254	RLK	LRR
Capsella bursa-pastoris	Cbp33312	RLK	LRR
Capsella bursa-pastoris	Cbp3336	RLK	Other-RLK
Capsella bursa-pastoris	Cbp33387	RLK	LRR
Capsella bursa-pastoris	Cbp33537	RLK	LRR
Capsella bursa-pastoris	Cbp33546	RLK	LRR
Capsella bursa-pastoris	Cbp3356	RLK	Other-RLK
Capsella bursa-pastoris	Cbp3357	RLK	Other-RLK
Capsella bursa-pastoris	Cbp33648	RLK	LRR
Capsella bursa-pastoris	Cbp33663	RLK	LRR
Capsella bursa-pastoris	Cbp33678	RLK	Other-RLK
Capsella bursa-pastoris	Cbp33727	RLK	Other-RLK
Capsella bursa-pastoris	Cbp33732	RLK	Other-RLK
Capsella bursa-pastoris	Cbp33733	RLK	Other-RLK
Capsella bursa-pastoris	Cbp33738	RLK	Other-RLK
Capsella bursa-pastoris	Cbp33747	RLK	Other-RLK
Capsella bursa-pastoris	Cbp33874	RLK	LRR
Capsella bursa-pastoris	Cbp34	RLK	LRR

Capsella bursa-pastoris	Cbp34033	RLK	LRR
Capsella bursa-pastoris	Cbp34125	RLK	LRR
Capsella bursa-pastoris	Cbp34184	RLK	LRR
Capsella bursa-pastoris	Cbp34185	RLK	LRR
Capsella bursa-pastoris	Cbp34186	RLK	LRR
Capsella bursa-pastoris	Cbp34194	RLK	LRR
Capsella bursa-pastoris	Cbp34208	RLK	Other-RLK
Capsella bursa-pastoris	Cbp34264	RLK	LRR
Capsella bursa-pastoris	Cbp34295	RLK	Other-RLK
Capsella bursa-pastoris	Cbp34344	RLK	LRR
Capsella bursa-pastoris	Cbp34345	RLK	LRR
Capsella bursa-pastoris	Cbp34449	RLK	LRR
Capsella bursa-pastoris	Cbp34641	RLK	Other-RLK
Capsella bursa-pastoris	Cbp34697	RLK	Other-RLK
Capsella bursa-pastoris	Cbp34698	RLK	Other-RLK
Capsella bursa-pastoris	Cbp34699	RLK	Other-RLK
Capsella bursa-pastoris	Cbp34700	RLK	Other-RLK
Capsella bursa-pastoris	Cbp34742	RLK	Other-RLK
Capsella bursa-pastoris	Cbp34931	RLK	Other-RLK
Capsella bursa-pastoris	Cbp34971	RLK	Other-RLK
Capsella bursa-pastoris	Cbp34972	RLK	Other-RLK
Capsella bursa-pastoris	Cbp34974	RLK	Other-RLK
Capsella bursa-pastoris	Cbp35106	RLK	LRR
Capsella bursa-pastoris	Cbp35268	RLK	Other-RLK
Capsella bursa-pastoris	Cbp35304	RLK	Other-RLK
Capsella bursa-pastoris	Cbp35484	RLK	LRR
Capsella bursa-pastoris	Cbp35488	RLK	LRR
Capsella bursa-pastoris	Cbp35500	RLK	Other-RLK
Capsella bursa-pastoris	Cbp35538	RLK	Other-RLK
Capsella bursa-pastoris	Cbp35539	RLK	Other-RLK
Capsella bursa-pastoris	Cbp35596	RLK	LRR
Capsella bursa-pastoris	Cbp35615	RLK	LRR
Capsella bursa-pastoris	Cbp3562	RLK	Other-RLK
Capsella bursa-pastoris	Cbp3579	RLK	LRR
Capsella bursa-pastoris	Cbp35962	RLK	LRR
Capsella bursa-pastoris	Cbp35963	RLK	LRR
Capsella bursa-pastoris	Cbp36013	RLK	Other-RLK
Capsella bursa-pastoris	Cbp36084	RLK	LRR
Capsella bursa-pastoris	Cbp36143	RLK	Other-RLK
Capsella bursa-pastoris	Cbp36156	RLK	LRR
Capsella bursa-pastoris	Cbp36165	RLK	LRR
Capsella bursa-pastoris	Cbp36166	RLK	LRR
Capsella bursa-pastoris	Cbp36168	RLK	LRR
Capsella bursa-pastoris	Cbp3643	RLK	LRR
Capsella bursa-pastoris	Cbp36460	RLK	Other-RLK
Capsella bursa-pastoris	Cbp36484	RLK	LRR
Capsella bursa-pastoris	Cbp36530	RLK	LRR
Capsella bursa-pastoris	Cbp36544	RLK	LRR
Capsella bursa-pastoris	Cbp36578	RLK	Other-RLK
Capsella bursa-pastoris	Cbp3690	RLK	Other-RLK
Capsella bursa-pastoris	Cbp36966	RLK	LRR
Capsella bursa-pastoris	Cbp36977	RLK	Other-RLK
Capsella bursa-pastoris	Cbp36995	RLK	Other-RLK
Capsella bursa-pastoris	Cbp370	RLK	Other-RLK
Capsella bursa-pastoris	Cbp37036	RLK	LRR
Capsella bursa-pastoris	Cbp37052	RLK	LRR
Capsella bursa-pastoris	Cbp37053	RLK	LRR
Capsella bursa-pastoris	Cbp37057	RLK	LRR
Capsella bursa-pastoris	Cbp37181	RLK	Other-RLK
Capsella bursa-pastoris	Cbp37256	RLK	Other-RLK
Capsella bursa-pastoris	Cbp37257	RLK	Other-RLK
Capsella bursa-pastoris	Cbp37274	RLK	Other-RLK

Capsella bursa-pastoris	Cbp37376	RLK	LRR
Capsella bursa-pastoris	Cbp37377	RLK	LRR
Capsella bursa-pastoris	Cbp37403	RLK	LRR
Capsella bursa-pastoris	Cbp37480	RLK	LRR
Capsella bursa-pastoris	Cbp37482	RLK	LRR
Capsella bursa-pastoris	Cbp37497	RLK	LRR
Capsella bursa-pastoris	Cbp37550	RLK	LRR
Capsella bursa-pastoris	Cbp37630	RLK	LRR
Capsella bursa-pastoris	Cbp37713	RLK	LRR
Capsella bursa-pastoris	Cbp37729	RLK	LRR
Capsella bursa-pastoris	Cbp38	RLK	LRR
Capsella bursa-pastoris	Cbp38028	RLK	LRR
Capsella bursa-pastoris	Cbp38092	RLK	Other-RLK
Capsella bursa-pastoris	Cbp38193	RLK	Other-RLK
Capsella bursa-pastoris	Cbp38211	RLK	LRR
Capsella bursa-pastoris	Cbp38224	RLK	LRR
Capsella bursa-pastoris	Cbp38226	RLK	Other-RLK
Capsella bursa-pastoris	Cbp38257	RLK	Other-RLK
Capsella bursa-pastoris	Cbp38267	RLK	LRR
Capsella bursa-pastoris	Cbp38308	RLK	LRR
Capsella bursa-pastoris	Cbp38392	RLK	LRR
Capsella bursa-pastoris	Cbp38429	RLK	Other-RLK
Capsella bursa-pastoris	Cbp38442	RLK	Other-RLK
Capsella bursa-pastoris	Cbp38452	RLK	LRR
Capsella bursa-pastoris	Cbp38533	RLK	Other-RLK
Capsella bursa-pastoris	Cbp38541	RLK	Other-RLK
Capsella bursa-pastoris	Cbp38634	RLK	LRR
Capsella bursa-pastoris	Cbp38703	RLK	LRR
Capsella bursa-pastoris	Cbp38874	RLK	Other-RLK
Capsella bursa-pastoris	Cbp389	RLK	LRR
Capsella bursa-pastoris	Cbp39056	RLK	Other-RLK
Capsella bursa-pastoris	Cbp39099	RLK	Other-RLK
Capsella bursa-pastoris	Cbp39125	RLK	Other-RLK
Capsella bursa-pastoris	Cbp39349	RLK	Other-RLK
Capsella bursa-pastoris	Cbp39465	RLK	Other-RLK
Capsella bursa-pastoris	Cbp39488	RLK	Other-RLK
Capsella bursa-pastoris	Cbp39490	RLK	Other-RLK
Capsella bursa-pastoris	Cbp39492	RLK	Other-RLK
Capsella bursa-pastoris	Cbp39493	RLK	Other-RLK
Capsella bursa-pastoris	Cbp39494	RLK	Other-RLK
Capsella bursa-pastoris	Cbp39510	RLK	Other-RLK
Capsella bursa-pastoris	Cbp39529	RLK	Other-RLK
Capsella bursa-pastoris	Cbp39573	RLK	Other-RLK
Capsella bursa-pastoris	Cbp39577	RLK	LRR
Capsella bursa-pastoris	Cbp39578	RLK	LRR
Capsella bursa-pastoris	Cbp39579	RLK	LRR
Capsella bursa-pastoris	Cbp39580	RLK	LRR
Capsella bursa-pastoris	Cbp39637	RLK	LRR
Capsella bursa-pastoris	Cbp39669	RLK	LRR
Capsella bursa-pastoris	Cbp39671	RLK	LRR
Capsella bursa-pastoris	Cbp39672	RLK	LRR
Capsella bursa-pastoris	Cbp3974	RLK	Other-RLK
Capsella bursa-pastoris	Cbp39862	RLK	LRR
Capsella bursa-pastoris	Cbp39914	RLK	LRR
Capsella bursa-pastoris	Cbp39916	RLK	Other-RLK
Capsella bursa-pastoris	Cbp40054	RLK	Other-RLK
Capsella bursa-pastoris	Cbp40103	RLK	Other-RLK
Capsella bursa-pastoris	Cbp40123	RLK	Other-RLK
Capsella bursa-pastoris	Cbp40324	RLK	Other-RLK
Capsella bursa-pastoris	Cbp40348	RLK	LRR
Capsella bursa-pastoris	Cbp40382	RLK	LRR
Capsella bursa-pastoris	Cbp40474	RLK	LRR

Capsella bursa-pastoris	Cbp405	RLK	Other-RLK
Capsella bursa-pastoris	Cbp40689	RLK	LRR
Capsella bursa-pastoris	Cbp4077	RLK	Other-RLK
Capsella bursa-pastoris	Cbp40991	RLK	Other-RLK
Capsella bursa-pastoris	Cbp40997	RLK	Other-RLK
Capsella bursa-pastoris	Cbp40998	RLK	Other-RLK
Capsella bursa-pastoris	Cbp40999	RLK	Other-RLK
Capsella bursa-pastoris	Cbp41001	RLK	Other-RLK
Capsella bursa-pastoris	Cbp41023	RLK	Other-RLK
Capsella bursa-pastoris	Cbp41047	RLK	Other-RLK
Capsella bursa-pastoris	Cbp41079	RLK	Other-RLK
Capsella bursa-pastoris	Cbp41157	RLK	LRR
Capsella bursa-pastoris	Cbp41168	RLK	Other-RLK
Capsella bursa-pastoris	Cbp41170	RLK	Other-RLK
Capsella bursa-pastoris	Cbp41321	RLK	LRR
Capsella bursa-pastoris	Cbp41370	RLK	LRR
Capsella bursa-pastoris	Cbp41661	RLK	LRR
Capsella bursa-pastoris	Cbp41671	RLK	LRR
Capsella bursa-pastoris	Cbp41672	RLK	LRR
Capsella bursa-pastoris	Cbp41673	RLK	LRR
Capsella bursa-pastoris	Cbp41724	RLK	Other-RLK
Capsella bursa-pastoris	Cbp41731	RLK	Other-RLK
Capsella bursa-pastoris	Cbp41732	RLK	Other-RLK
Capsella bursa-pastoris	Cbp41733	RLK	Other-RLK
Capsella bursa-pastoris	Cbp41734	RLK	Other-RLK
Capsella bursa-pastoris	Cbp41778	RLK	Other-RLK
Capsella bursa-pastoris	Cbp41880	RLK	LRR
Capsella bursa-pastoris	Cbp41977	RLK	Other-RLK
Capsella bursa-pastoris	Cbp42093	RLK	LRR
Capsella bursa-pastoris	Cbp42151	RLK	Other-RLK
Capsella bursa-pastoris	Cbp42314	RLK	Other-RLK
Capsella bursa-pastoris	Cbp42426	RLK	Other-RLK
Capsella bursa-pastoris	Cbp4243	RLK	Other-RLK
Capsella bursa-pastoris	Cbp42447	RLK	LRR
Capsella bursa-pastoris	Cbp42529	RLK	LRR
Capsella bursa-pastoris	Cbp42540	RLK	Other-RLK
Capsella bursa-pastoris	Cbp42541	RLK	Other-RLK
Capsella bursa-pastoris	Cbp42561	RLK	Other-RLK
Capsella bursa-pastoris	Cbp42603	RLK	LRR
Capsella bursa-pastoris	Cbp42618	RLK	LRR
Capsella bursa-pastoris	Cbp42622	RLK	LRR
Capsella bursa-pastoris	Cbp42756	RLK	Other-RLK
Capsella bursa-pastoris	Cbp42823	RLK	Other-RLK
Capsella bursa-pastoris	Cbp42872	RLK	LRR
Capsella bursa-pastoris	Cbp42873	RLK	LRR
Capsella bursa-pastoris	Cbp42874	RLK	LRR
Capsella bursa-pastoris	Cbp42875	RLK	LRR
Capsella bursa-pastoris	Cbp42919	RLK	Other-RLK
Capsella bursa-pastoris	Cbp42965	RLK	LRR
Capsella bursa-pastoris	Cbp43004	RLK	Other-RLK
Capsella bursa-pastoris	Cbp43118	RLK	Other-RLK
Capsella bursa-pastoris	Cbp43237	RLK	Other-RLK
Capsella bursa-pastoris	Cbp43310	RLK	LRR
Capsella bursa-pastoris	Cbp43359	RLK	LRR
Capsella bursa-pastoris	Cbp43377	RLK	Other-RLK
Capsella bursa-pastoris	Cbp43438	RLK	LRR
Capsella bursa-pastoris	Cbp43466	RLK	LRR
Capsella bursa-pastoris	Cbp43530	RLK	Other-RLK
Capsella bursa-pastoris	Cbp43746	RLK	Other-RLK
Capsella bursa-pastoris	Cbp43762	RLK	Other-RLK
Capsella bursa-pastoris	Cbp43856	RLK	Other-RLK
Capsella bursa-pastoris	Cbp43946	RLK	Other-RLK

Capsella bursa-pastoris	Cbp43977	RLK	Other-RLK
Capsella bursa-pastoris	Cbp44	RLK	Other-RLK
Capsella bursa-pastoris	Cbp44010	RLK	Other-RLK
Capsella bursa-pastoris	Cbp44069	RLK	Other-RLK
Capsella bursa-pastoris	Cbp44104	RLK	LRR
Capsella bursa-pastoris	Cbp44204	RLK	LRR
Capsella bursa-pastoris	Cbp44331	RLK	LRR
Capsella bursa-pastoris	Cbp44371	RLK	LRR
Capsella bursa-pastoris	Cbp44396	RLK	LRR
Capsella bursa-pastoris	Cbp44412	RLK	LRR
Capsella bursa-pastoris	Cbp44456	RLK	LRR
Capsella bursa-pastoris	Cbp44518	RLK	Other-RLK
Capsella bursa-pastoris	Cbp44670	RLK	Other-RLK
Capsella bursa-pastoris	Cbp44671	RLK	Other-RLK
Capsella bursa-pastoris	Cbp44673	RLK	Other-RLK
Capsella bursa-pastoris	Cbp44674	RLK	Other-RLK
Capsella bursa-pastoris	Cbp44677	RLK	Other-RLK
Capsella bursa-pastoris	Cbp44875	RLK	Other-RLK
Capsella bursa-pastoris	Cbp44934	RLK	LRR
Capsella bursa-pastoris	Cbp45027	RLK	Other-RLK
Capsella bursa-pastoris	Cbp45073	RLK	LRR
Capsella bursa-pastoris	Cbp45425	RLK	LRR
Capsella bursa-pastoris	Cbp45428	RLK	Other-RLK
Capsella bursa-pastoris	Cbp45429	RLK	LRR
Capsella bursa-pastoris	Cbp45432	RLK	LRR
Capsella bursa-pastoris	Cbp45532	RLK	LRR
Capsella bursa-pastoris	Cbp45562	RLK	LRR
Capsella bursa-pastoris	Cbp46103	RLK	Other-RLK
Capsella bursa-pastoris	Cbp46167	RLK	Other-RLK
Capsella bursa-pastoris	Cbp46187	RLK	LRR
Capsella bursa-pastoris	Cbp46188	RLK	LRR
Capsella bursa-pastoris	Cbp46189	RLK	LRR
Capsella bursa-pastoris	Cbp46190	RLK	LRR
Capsella bursa-pastoris	Cbp46191	RLK	LRR
Capsella bursa-pastoris	Cbp46192	RLK	LRR
Capsella bursa-pastoris	Cbp46196	RLK	Lysm
Capsella bursa-pastoris	Cbp46234	RLK	Other-RLK
Capsella bursa-pastoris	Cbp46352	RLK	LRR
Capsella bursa-pastoris	Cbp46353	RLK	LRR
Capsella bursa-pastoris	Cbp46381	RLK	LRR
Capsella bursa-pastoris	Cbp4642	RLK	LRR
Capsella bursa-pastoris	Cbp46658	RLK	Other-RLK
Capsella bursa-pastoris	Cbp46804	RLK	Other-RLK
Capsella bursa-pastoris	Cbp46805	RLK	Other-RLK
Capsella bursa-pastoris	Cbp46885	RLK	Other-RLK
Capsella bursa-pastoris	Cbp46894	RLK	Other-RLK
Capsella bursa-pastoris	Cbp46901	RLK	Other-RLK
Capsella bursa-pastoris	Cbp46902	RLK	Other-RLK
Capsella bursa-pastoris	Cbp46903	RLK	Other-RLK
Capsella bursa-pastoris	Cbp46958	RLK	LRR
Capsella bursa-pastoris	Cbp46981	RLK	LRR
Capsella bursa-pastoris	Cbp47025	RLK	Other-RLK
Capsella bursa-pastoris	Cbp47030	RLK	LRR
Capsella bursa-pastoris	Cbp47067	RLK	Other-RLK
Capsella bursa-pastoris	Cbp4710	RLK	Other-RLK
Capsella bursa-pastoris	Cbp47117	RLK	LRR
Capsella bursa-pastoris	Cbp47165	RLK	LRR
Capsella bursa-pastoris	Cbp47177	RLK	LRR
Capsella bursa-pastoris	Cbp47192	RLK	LRR
Capsella bursa-pastoris	Cbp47224	RLK	LRR
Capsella bursa-pastoris	Cbp47329	RLK	Other-RLK
Capsella bursa-pastoris	Cbp47330	RLK	Other-RLK

Capsella bursa-pastoris	Cbp47344	RLK	Other-RLK
Capsella bursa-pastoris	Cbp47354	RLK	LRR
Capsella bursa-pastoris	Cbp47356	RLK	LRR
Capsella bursa-pastoris	Cbp47362	RLK	LRR
Capsella bursa-pastoris	Cbp47364	RLK	LRR
Capsella bursa-pastoris	Cbp47366	RLK	LRR
Capsella bursa-pastoris	Cbp47460	RLK	LRR
Capsella bursa-pastoris	Cbp47469	RLK	Other-RLK
Capsella bursa-pastoris	Cbp47493	RLK	LRR
Capsella bursa-pastoris	Cbp4758	RLK	Other-RLK
Capsella bursa-pastoris	Cbp4759	RLK	Other-RLK
Capsella bursa-pastoris	Cbp4767	RLK	Other-RLK
Capsella bursa-pastoris	Cbp47776	RLK	Other-RLK
Capsella bursa-pastoris	Cbp47782	RLK	Other-RLK
Capsella bursa-pastoris	Cbp47800	RLK	LRR
Capsella bursa-pastoris	Cbp47844	RLK	Other-RLK
Capsella bursa-pastoris	Cbp4797	RLK	Other-RLK
Capsella bursa-pastoris	Cbp4799	RLK	Other-RLK
Capsella bursa-pastoris	Cbp48025	RLK	Other-RLK
Capsella bursa-pastoris	Cbp48026	RLK	Other-RLK
Capsella bursa-pastoris	Cbp48206	RLK	Other-RLK
Capsella bursa-pastoris	Cbp48224	RLK	Other-RLK
Capsella bursa-pastoris	Cbp48372	RLK	Other-RLK
Capsella bursa-pastoris	Cbp4851	RLK	Other-RLK
Capsella bursa-pastoris	Cbp48535	RLK	LRR
Capsella bursa-pastoris	Cbp48577	RLK	LRR
Capsella bursa-pastoris	Cbp48710	RLK	LRR
Capsella bursa-pastoris	Cbp48717	RLK	Other-RLK
Capsella bursa-pastoris	Cbp48721	RLK	Other-RLK
Capsella bursa-pastoris	Cbp48722	RLK	Other-RLK
Capsella bursa-pastoris	Cbp48723	RLK	Other-RLK
Capsella bursa-pastoris	Cbp48724	RLK	Other-RLK
Capsella bursa-pastoris	Cbp48725	RLK	Other-RLK
Capsella bursa-pastoris	Cbp48729	RLK	Other-RLK
Capsella bursa-pastoris	Cbp48732	RLK	Other-RLK
Capsella bursa-pastoris	Cbp48734	RLK	Other-RLK
Capsella bursa-pastoris	Cbp48946	RLK	Other-RLK
Capsella bursa-pastoris	Cbp49001	RLK	Other-RLK
Capsella bursa-pastoris	Cbp49033	RLK	LRR
Capsella bursa-pastoris	Cbp49034	RLK	LRR
Capsella bursa-pastoris	Cbp49054	RLK	LRR
Capsella bursa-pastoris	Cbp49095	RLK	Other-RLK
Capsella bursa-pastoris	Cbp49097	RLK	Other-RLK
Capsella bursa-pastoris	Cbp49189	RLK	Other-RLK
Capsella bursa-pastoris	Cbp49191	RLK	Other-RLK
Capsella bursa-pastoris	Cbp49193	RLK	Other-RLK
Capsella bursa-pastoris	Cbp49216	RLK	Other-RLK
Capsella bursa-pastoris	Cbp49244	RLK	Other-RLK
Capsella bursa-pastoris	Cbp49312	RLK	LRR
Capsella bursa-pastoris	Cbp49370	RLK	Other-RLK
Capsella bursa-pastoris	Cbp49461	RLK	Other-RLK
Capsella bursa-pastoris	Cbp49609	RLK	LRR
Capsella bursa-pastoris	Cbp49735	RLK	Other-RLK
Capsella bursa-pastoris	Cbp49757	RLK	LRR
Capsella bursa-pastoris	Cbp49810	RLK	LRR
Capsella bursa-pastoris	Cbp50057	RLK	Other-RLK
Capsella bursa-pastoris	Cbp50096	RLK	LRR
Capsella bursa-pastoris	Cbp50149	RLK	LRR
Capsella bursa-pastoris	Cbp50197	RLK	LRR
Capsella bursa-pastoris	Cbp50222	RLK	Other-RLK
Capsella bursa-pastoris	Cbp50250	RLK	LRR
Capsella bursa-pastoris	Cbp50262	RLK	LRR

Capsella bursa-pastoris	Cbp50347	RLK	LRR
Capsella bursa-pastoris	Cbp50612	RLK	LRR
Capsella bursa-pastoris	Cbp50624	RLK	Other-RLK
Capsella bursa-pastoris	Cbp50730	RLK	LRR
Capsella bursa-pastoris	Cbp50750	RLK	LRR
Capsella bursa-pastoris	Cbp50757	RLK	Other-RLK
Capsella bursa-pastoris	Cbp50782	RLK	Other-RLK
Capsella bursa-pastoris	Cbp50922	RLK	Other-RLK
Capsella bursa-pastoris	Cbp50930	RLK	LRR
Capsella bursa-pastoris	Cbp50952	RLK	LRR
Capsella bursa-pastoris	Cbp51058	RLK	Other-RLK
Capsella bursa-pastoris	Cbp51117	RLK	LRR
Capsella bursa-pastoris	Cbp51156	RLK	Other-RLK
Capsella bursa-pastoris	Cbp51195	RLK	Other-RLK
Capsella bursa-pastoris	Cbp51196	RLK	Other-RLK
Capsella bursa-pastoris	Cbp51205	RLK	LRR
Capsella bursa-pastoris	Cbp51226	RLK	Other-RLK
Capsella bursa-pastoris	Cbp51233	RLK	Other-RLK
Capsella bursa-pastoris	Cbp51338	RLK	LRR
Capsella bursa-pastoris	Cbp51403	RLK	Other-RLK
Capsella bursa-pastoris	Cbp51434	RLK	Other-RLK
Capsella bursa-pastoris	Cbp51542	RLK	Other-RLK
Capsella bursa-pastoris	Cbp51556	RLK	Other-RLK
Capsella bursa-pastoris	Cbp51565	RLK	LRR
Capsella bursa-pastoris	Cbp51643	RLK	Other-RLK
Capsella bursa-pastoris	Cbp51650	RLK	Other-RLK
Capsella bursa-pastoris	Cbp51685	RLK	Other-RLK
Capsella bursa-pastoris	Cbp51879	RLK	Other-RLK
Capsella bursa-pastoris	Cbp51905	RLK	LRR
Capsella bursa-pastoris	Cbp52016	RLK	LRR
Capsella bursa-pastoris	Cbp52032	RLK	LRR
Capsella bursa-pastoris	Cbp5216	RLK	LRR
Capsella bursa-pastoris	Cbp52181	RLK	Other-RLK
Capsella bursa-pastoris	Cbp52311	RLK	Other-RLK
Capsella bursa-pastoris	Cbp52489	RLK	Other-RLK
Capsella bursa-pastoris	Cbp52680	RLK	Other-RLK
Capsella bursa-pastoris	Cbp52747	RLK	Other-RLK
Capsella bursa-pastoris	Cbp52887	RLK	Other-RLK
Capsella bursa-pastoris	Cbp5293	RLK	LRR
Capsella bursa-pastoris	Cbp52944	RLK	Other-RLK
Capsella bursa-pastoris	Cbp53010	RLK	Other-RLK
Capsella bursa-pastoris	Cbp53011	RLK	Other-RLK
Capsella bursa-pastoris	Cbp53012	RLK	Other-RLK
Capsella bursa-pastoris	Cbp53013	RLK	Other-RLK
Capsella bursa-pastoris	Cbp53019	RLK	Other-RLK
Capsella bursa-pastoris	Cbp53023	RLK	Other-RLK
Capsella bursa-pastoris	Cbp53105	RLK	Other-RLK
Capsella bursa-pastoris	Cbp53195	RLK	LRR
Capsella bursa-pastoris	Cbp53260	RLK	LRR
Capsella bursa-pastoris	Cbp53291	RLK	Other-RLK
Capsella bursa-pastoris	Cbp53343	RLK	LRR
Capsella bursa-pastoris	Cbp53378	RLK	LRR
Capsella bursa-pastoris	Cbp5357	RLK	LRR
Capsella bursa-pastoris	Cbp5394	RLK	LRR
Capsella bursa-pastoris	Cbp5469	RLK	Other-RLK
Capsella bursa-pastoris	Cbp5484	RLK	LRR
Capsella bursa-pastoris	Cbp5538	RLK	Other-RLK
Capsella bursa-pastoris	Cbp5539	RLK	Other-RLK
Capsella bursa-pastoris	Cbp5540	RLK	Other-RLK
Capsella bursa-pastoris	Cbp5542	RLK	Other-RLK
Capsella bursa-pastoris	Cbp5596	RLK	Other-RLK
Capsella bursa-pastoris	Cbp5621	RLK	Other-RLK

Capsella bursa-pastoris	Cbp5761	RLK	Other-RLK
Capsella bursa-pastoris	Cbp5831	RLK	LRR
Capsella bursa-pastoris	Cbp5939	RLK	Other-RLK
Capsella bursa-pastoris	Cbp6033	RLK	LRR
Capsella bursa-pastoris	Cbp6124	RLK	LRR
Capsella bursa-pastoris	Cbp6173	RLK	LRR
Capsella bursa-pastoris	Cbp6234	RLK	Other-RLK
Capsella bursa-pastoris	Cbp6523	RLK	Other-RLK
Capsella bursa-pastoris	Cbp6572	RLK	Lysm
Capsella bursa-pastoris	Cbp66	RLK	Other-RLK
Capsella bursa-pastoris	Cbp67	RLK	Other-RLK
Capsella bursa-pastoris	Cbp6725	RLK	LRR
Capsella bursa-pastoris	Cbp6747	RLK	LRR
Capsella bursa-pastoris	Cbp68	RLK	Other-RLK
Capsella bursa-pastoris	Cbp6810	RLK	LRR
Capsella bursa-pastoris	Cbp6821	RLK	Other-RLK
Capsella bursa-pastoris	Cbp6828	RLK	LRR
Capsella bursa-pastoris	Cbp6831	RLK	LRR
Capsella bursa-pastoris	Cbp6840	RLK	LRR
Capsella bursa-pastoris	Cbp685	RLK	Other-RLK
Capsella bursa-pastoris	Cbp6905	RLK	Other-RLK
Capsella bursa-pastoris	Cbp7008	RLK	Other-RLK
Capsella bursa-pastoris	Cbp7038	RLK	LRR
Capsella bursa-pastoris	Cbp7104	RLK	LRR
Capsella bursa-pastoris	Cbp7236	RLK	Other-RLK
Capsella bursa-pastoris	Cbp7274	RLK	LRR
Capsella bursa-pastoris	Cbp7300	RLK	Other-RLK
Capsella bursa-pastoris	Cbp7390	RLK	Other-RLK
Capsella bursa-pastoris	Cbp7413	RLK	Other-RLK
Capsella bursa-pastoris	Cbp7583	RLK	LRR
Capsella bursa-pastoris	Cbp7644	RLK	LRR
Capsella bursa-pastoris	Cbp7661	RLK	Other-RLK
Capsella bursa-pastoris	Cbp7793	RLK	Other-RLK
Capsella bursa-pastoris	Cbp7807	RLK	LRR
Capsella bursa-pastoris	Cbp7824	RLK	LRR
Capsella bursa-pastoris	Cbp7909	RLK	LRR
Capsella bursa-pastoris	Cbp7971	RLK	LRR
Capsella bursa-pastoris	Cbp7972	RLK	LRR
Capsella bursa-pastoris	Cbp7974	RLK	LRR
Capsella bursa-pastoris	Cbp7984	RLK	LRR
Capsella bursa-pastoris	Cbp8067	RLK	LRR
Capsella bursa-pastoris	Cbp8084	RLK	LRR
Capsella bursa-pastoris	Cbp8360	RLK	LRR
Capsella bursa-pastoris	Cbp8395	RLK	Other-RLK
Capsella bursa-pastoris	Cbp8580	RLK	Other-RLK
Capsella bursa-pastoris	Cbp8598	RLK	LRR
Capsella bursa-pastoris	Cbp8649	RLK	LRR
Capsella bursa-pastoris	Cbp8671	RLK	Other-RLK
Capsella bursa-pastoris	Cbp8696	RLK	Other-RLK
Capsella bursa-pastoris	Cbp8697	RLK	Other-RLK
Capsella bursa-pastoris	Cbp8698	RLK	Other-RLK
Capsella bursa-pastoris	Cbp8699	RLK	Other-RLK
Capsella bursa-pastoris	Cbp8701	RLK	Other-RLK
Capsella bursa-pastoris	Cbp8702	RLK	Other-RLK
Capsella bursa-pastoris	Cbp8703	RLK	Other-RLK
Capsella bursa-pastoris	Cbp8704	RLK	Other-RLK
Capsella bursa-pastoris	Cbp8706	RLK	Other-RLK
Capsella bursa-pastoris	Cbp8707	RLK	Other-RLK
Capsella bursa-pastoris	Cbp8708	RLK	Other-RLK
Capsella bursa-pastoris	Cbp8709	RLK	Other-RLK
Capsella bursa-pastoris	Cbp8711	RLK	Other-RLK
Capsella bursa-pastoris	Cbp8712	RLK	Other-RLK

Capsella bursa-pastoris	Cbp8713	RLK	Other-RLK
Capsella bursa-pastoris	Cbp8714	RLK	Other-RLK
Capsella bursa-pastoris	Cbp8715	RLK	Other-RLK
Capsella bursa-pastoris	Cbp8758	RLK	LRR
Capsella bursa-pastoris	Cbp8888	RLK	LRR
Capsella bursa-pastoris	Cbp8968	RLK	LRR
Capsella bursa-pastoris	Cbp9020	RLK	Other-RLK
Capsella bursa-pastoris	Cbp9044	RLK	Other-RLK
Capsella bursa-pastoris	Cbp9045	RLK	Other-RLK
Capsella bursa-pastoris	Cbp9046	RLK	Other-RLK
Capsella bursa-pastoris	Cbp9095	RLK	Other-RLK
Capsella bursa-pastoris	Cbp9217	RLK	LRR
Capsella bursa-pastoris	Cbp9297	RLK	LRR
Capsella bursa-pastoris	Cbp9344	RLK	Other-RLK
Capsella bursa-pastoris	Cbp9655	RLK	LRR
Capsella bursa-pastoris	Cbp989	RLK	Other-RLK
Capsella bursa-pastoris	Cbp991	RLK	LRR
Capsella bursa-pastoris	Cbp9930	RLK	Other-RLK
Capsella bursa-pastoris	Cbp996	RLK	Other-RLK
Capsella bursa-pastoris	Cbp9961	RLK	Other-RLK
Capsella bursa-pastoris	Cbp9966	RLK	LRR
Capsella bursa-pastoris	Cbp10091	RLP	LRR
Capsella bursa-pastoris	Cbp10094	RLP	LRR
Capsella bursa-pastoris	Cbp10097	RLP	LRR
Capsella bursa-pastoris	Cbp10098	RLP	LRR
Capsella bursa-pastoris	Cbp10100	RLP	LRR
Capsella bursa-pastoris	Cbp10101	RLP	LRR
Capsella bursa-pastoris	Cbp10313	RLP	LRR
Capsella bursa-pastoris	Cbp1042	RLP	LRR
Capsella bursa-pastoris	Cbp10857	RLP	LRR
Capsella bursa-pastoris	Cbp11544	RLP	LRR
Capsella bursa-pastoris	Cbp11711	RLP	LRR
Capsella bursa-pastoris	Cbp12014	RLP	LRR
Capsella bursa-pastoris	Cbp12346	RLP	LRR
Capsella bursa-pastoris	Cbp12350	RLP	LRR
Capsella bursa-pastoris	Cbp13087	RLP	Lysm
Capsella bursa-pastoris	Cbp1351	RLP	LRR
Capsella bursa-pastoris	Cbp13801	RLP	LRR
Capsella bursa-pastoris	Cbp14033	RLP	LRR
Capsella bursa-pastoris	Cbp14166	RLP	LRR
Capsella bursa-pastoris	Cbp14169	RLP	LRR
Capsella bursa-pastoris	Cbp14312	RLP	LRR
Capsella bursa-pastoris	Cbp14314	RLP	LRR
Capsella bursa-pastoris	Cbp14568	RLP	LRR
Capsella bursa-pastoris	Cbp14812	RLP	Lysm
Capsella bursa-pastoris	Cbp1501	RLP	LRR
Capsella bursa-pastoris	Cbp15125	RLP	LRR
Capsella bursa-pastoris	Cbp15744	RLP	LRR
Capsella bursa-pastoris	Cbp15925	RLP	LRR
Capsella bursa-pastoris	Cbp16010	RLP	LRR
Capsella bursa-pastoris	Cbp16977	RLP	LRR
Capsella bursa-pastoris	Cbp17498	RLP	LRR
Capsella bursa-pastoris	Cbp17520	RLP	LRR
Capsella bursa-pastoris	Cbp17717	RLP	LRR
Capsella bursa-pastoris	Cbp1801	RLP	LRR
Capsella bursa-pastoris	Cbp1830	RLP	LRR
Capsella bursa-pastoris	Cbp18477	RLP	LRR
Capsella bursa-pastoris	Cbp18916	RLP	LRR
Capsella bursa-pastoris	Cbp19186	RLP	LRR
Capsella bursa-pastoris	Cbp19486	RLP	LRR
Capsella bursa-pastoris	Cbp19607	RLP	LRR
Capsella bursa-pastoris	Cbp19763	RLP	LRR

Capsella bursa-pastoris	Cbp20084	RLP	LRR
Capsella bursa-pastoris	Cbp20261	RLP	LRR
Capsella bursa-pastoris	Cbp20439	RLP	LRR
Capsella bursa-pastoris	Cbp20445	RLP	LRR
Capsella bursa-pastoris	Cbp20661	RLP	LRR
Capsella bursa-pastoris	Cbp21168	RLP	LRR
Capsella bursa-pastoris	Cbp21211	RLP	LRR
Capsella bursa-pastoris	Cbp21504	RLP	LRR
Capsella bursa-pastoris	Cbp23118	RLP	LRR
Capsella bursa-pastoris	Cbp24785	RLP	LRR
Capsella bursa-pastoris	Cbp24792	RLP	LRR
Capsella bursa-pastoris	Cbp25274	RLP	LRR
Capsella bursa-pastoris	Cbp2531	RLP	LRR
Capsella bursa-pastoris	Cbp2534	RLP	LRR
Capsella bursa-pastoris	Cbp2538	RLP	LRR
Capsella bursa-pastoris	Cbp2540	RLP	LRR
Capsella bursa-pastoris	Cbp25545	RLP	LRR
Capsella bursa-pastoris	Cbp25706	RLP	LRR
Capsella bursa-pastoris	Cbp26867	RLP	LRR
Capsella bursa-pastoris	Cbp26915	RLP	LRR
Capsella bursa-pastoris	Cbp26937	RLP	LRR
Capsella bursa-pastoris	Cbp27048	RLP	LRR
Capsella bursa-pastoris	Cbp27226	RLP	LRR
Capsella bursa-pastoris	Cbp27804	RLP	Lysm
Capsella bursa-pastoris	Cbp28040	RLP	LRR
Capsella bursa-pastoris	Cbp28068	RLP	LRR
Capsella bursa-pastoris	Cbp28449	RLP	LRR
Capsella bursa-pastoris	Cbp28518	RLP	LRR
Capsella bursa-pastoris	Cbp28870	RLP	LRR
Capsella bursa-pastoris	Cbp29378	RLP	LRR
Capsella bursa-pastoris	Cbp30375	RLP	LRR
Capsella bursa-pastoris	Cbp30385	RLP	LRR
Capsella bursa-pastoris	Cbp30795	RLP	LRR
Capsella bursa-pastoris	Cbp31077	RLP	LRR
Capsella bursa-pastoris	Cbp31300	RLP	LRR
Capsella bursa-pastoris	Cbp31953	RLP	LRR
Capsella bursa-pastoris	Cbp31954	RLP	LRR
Capsella bursa-pastoris	Cbp31968	RLP	LRR
Capsella bursa-pastoris	Cbp32383	RLP	LRR
Capsella bursa-pastoris	Cbp32559	RLP	LRR
Capsella bursa-pastoris	Cbp32753	RLP	LRR
Capsella bursa-pastoris	Cbp32754	RLP	LRR
Capsella bursa-pastoris	Cbp32755	RLP	LRR
Capsella bursa-pastoris	Cbp33361	RLP	LRR
Capsella bursa-pastoris	Cbp33476	RLP	LRR
Capsella bursa-pastoris	Cbp34170	RLP	LRR
Capsella bursa-pastoris	Cbp34354	RLP	LRR
Capsella bursa-pastoris	Cbp35198	RLP	LRR
Capsella bursa-pastoris	Cbp35582	RLP	LRR
Capsella bursa-pastoris	Cbp36183	RLP	LRR
Capsella bursa-pastoris	Cbp3640	RLP	LRR
Capsella bursa-pastoris	Cbp36410	RLP	LRR
Capsella bursa-pastoris	Cbp36452	RLP	LRR
Capsella bursa-pastoris	Cbp36938	RLP	LRR
Capsella bursa-pastoris	Cbp36940	RLP	LRR
Capsella bursa-pastoris	Cbp36941	RLP	LRR
Capsella bursa-pastoris	Cbp36942	RLP	LRR
Capsella bursa-pastoris	Cbp36944	RLP	LRR
Capsella bursa-pastoris	Cbp37121	RLP	LRR
Capsella bursa-pastoris	Cbp37122	RLP	LRR
Capsella bursa-pastoris	Cbp37500	RLP	LRR
Capsella bursa-pastoris	Cbp3783	RLP	LRR

Capsella bursa-pastoris	Cbp37927	RLP	LRR
Capsella bursa-pastoris	Cbp38218	RLP	LRR
Capsella bursa-pastoris	Cbp3824	RLP	LRR
Capsella bursa-pastoris	Cbp38575	RLP	LRR
Capsella bursa-pastoris	Cbp39366	RLP	LRR
Capsella bursa-pastoris	Cbp39423	RLP	LRR
Capsella bursa-pastoris	Cbp39427	RLP	LRR
Capsella bursa-pastoris	Cbp3946	RLP	LRR
Capsella bursa-pastoris	Cbp39871	RLP	LRR
Capsella bursa-pastoris	Cbp39953	RLP	LRR
Capsella bursa-pastoris	Cbp40390	RLP	LRR
Capsella bursa-pastoris	Cbp40391	RLP	LRR
Capsella bursa-pastoris	Cbp4047	RLP	LRR
Capsella bursa-pastoris	Cbp4055	RLP	LRR
Capsella bursa-pastoris	Cbp4056	RLP	LRR
Capsella bursa-pastoris	Cbp4057	RLP	LRR
Capsella bursa-pastoris	Cbp40579	RLP	LRR
Capsella bursa-pastoris	Cbp4058	RLP	LRR
Capsella bursa-pastoris	Cbp4059	RLP	LRR
Capsella bursa-pastoris	Cbp40594	RLP	LRR
Capsella bursa-pastoris	Cbp4060	RLP	LRR
Capsella bursa-pastoris	Cbp40774	RLP	LRR
Capsella bursa-pastoris	Cbp41264	RLP	LRR
Capsella bursa-pastoris	Cbp41308	RLP	LRR
Capsella bursa-pastoris	Cbp41869	RLP	LRR
Capsella bursa-pastoris	Cbp42143	RLP	LRR
Capsella bursa-pastoris	Cbp42217	RLP	LRR
Capsella bursa-pastoris	Cbp42488	RLP	LRR
Capsella bursa-pastoris	Cbp42492	RLP	LRR
Capsella bursa-pastoris	Cbp42555	RLP	LRR
Capsella bursa-pastoris	Cbp42686	RLP	LRR
Capsella bursa-pastoris	Cbp4279	RLP	LRR
Capsella bursa-pastoris	Cbp42975	RLP	LRR
Capsella bursa-pastoris	Cbp4323	RLP	LRR
Capsella bursa-pastoris	Cbp43827	RLP	LRR
Capsella bursa-pastoris	Cbp44101	RLP	LRR
Capsella bursa-pastoris	Cbp44116	RLP	LRR
Capsella bursa-pastoris	Cbp44492	RLP	LRR
Capsella bursa-pastoris	Cbp45436	RLP	LRR
Capsella bursa-pastoris	Cbp45462	RLP	LRR
Capsella bursa-pastoris	Cbp45463	RLP	LRR
Capsella bursa-pastoris	Cbp45769	RLP	LRR
Capsella bursa-pastoris	Cbp45770	RLP	LRR
Capsella bursa-pastoris	Cbp45904	RLP	LRR
Capsella bursa-pastoris	Cbp46646	RLP	LRR
Capsella bursa-pastoris	Cbp46751	RLP	LRR
Capsella bursa-pastoris	Cbp46755	RLP	LRR
Capsella bursa-pastoris	Cbp47252	RLP	LRR
Capsella bursa-pastoris	Cbp47415	RLP	LRR
Capsella bursa-pastoris	Cbp47438	RLP	LRR
Capsella bursa-pastoris	Cbp47907	RLP	LRR
Capsella bursa-pastoris	Cbp48108	RLP	LRR
Capsella bursa-pastoris	Cbp48636	RLP	LRR
Capsella bursa-pastoris	Cbp48744	RLP	LRR
Capsella bursa-pastoris	Cbp48845	RLP	LRR
Capsella bursa-pastoris	Cbp49817	RLP	LRR
Capsella bursa-pastoris	Cbp5035	RLP	LRR
Capsella bursa-pastoris	Cbp5057	RLP	LRR
Capsella bursa-pastoris	Cbp5067	RLP	LRR
Capsella bursa-pastoris	Cbp50773	RLP	LRR
Capsella bursa-pastoris	Cbp50906	RLP	LRR
Capsella bursa-pastoris	Cbp51141	RLP	LRR

Capsella bursa-pastoris	Cbp5197	RLP	LRR
Capsella bursa-pastoris	Cbp52377	RLP	LRR
Capsella bursa-pastoris	Cbp52378	RLP	LRR
Capsella bursa-pastoris	Cbp52423	RLP	LRR
Capsella bursa-pastoris	Cbp52571	RLP	LRR
Capsella bursa-pastoris	Cbp52577	RLP	LRR
Capsella bursa-pastoris	Cbp52579	RLP	LRR
Capsella bursa-pastoris	Cbp52785	RLP	LRR
Capsella bursa-pastoris	Cbp52787	RLP	LRR
Capsella bursa-pastoris	Cbp52982	RLP	LRR
Capsella bursa-pastoris	Cbp52983	RLP	LRR
Capsella bursa-pastoris	Cbp53470	RLP	LRR
Capsella bursa-pastoris	Cbp53472	RLP	LRR
Capsella bursa-pastoris	Cbp5525	RLP	LRR
Capsella bursa-pastoris	Cbp5526	RLP	LRR
Capsella bursa-pastoris	Cbp6319	RLP	LRR
Capsella bursa-pastoris	Cbp6620	RLP	LRR
Capsella bursa-pastoris	Cbp6654	RLP	LRR
Capsella bursa-pastoris	Cbp6658	RLP	LRR
Capsella bursa-pastoris	Cbp7339	RLP	LRR
Capsella bursa-pastoris	Cbp7586	RLP	LRR
Capsella bursa-pastoris	Cbp7691	RLP	LRR
Capsella bursa-pastoris	Cbp7692	RLP	LRR
Capsella bursa-pastoris	Cbp8083	RLP	LRR
Capsella bursa-pastoris	Cbp8111	RLP	LRR
Capsella bursa-pastoris	Cbp8136	RLP	LRR
Capsella bursa-pastoris	Cbp8400	RLP	LRR
Capsella bursa-pastoris	Cbp8410	RLP	LRR
Capsella bursa-pastoris	Cbp9261	RLP	LRR
Capsella bursa-pastoris	Cbp9505	RLP	LRR
Capsella bursa-pastoris	Cbp9533	RLP	LRR
Brassica cretica	KAF3515794.1	NLR	CN
Brassica cretica	KAF3610902.1	NLR	CN
Brassica cretica	KAF3532707.1	NLR	CN
Brassica cretica	KAF3497108.1	NLR	CN
Brassica cretica	KAF3495114.1	NLR	CN
Brassica cretica	KAF3532451.1	NLR	CN
Brassica cretica	KAF3577281.1	NLR	CN
Brassica cretica	KAF3597557.1	NLR	CN
Brassica cretica	KAF3498007.1	NLR	CN
Brassica cretica	KAF3545115.1	NLR	CN
Brassica cretica	KAF3517781.1	NLR	CN
Brassica cretica	KAF3590472.1	NLR	CN
Brassica cretica	KAF3517049.1	NLR	CN
Brassica cretica	KAF3531071.1	NLR	CNL
Brassica cretica	KAF3531036.1	NLR	CNL
Brassica cretica	KAF3611428.1	NLR	CNL
Brassica cretica	KAF3516560.1	NLR	CNL
Brassica cretica	KAF3581220.1	NLR	CNL
Brassica cretica	KAF3497592.1	NLR	CNL
Brassica cretica	KAF3562989.1	NLR	CNL
Brassica cretica	KAF3596471.1	NLR	CNL
Brassica cretica	KAF3604874.1	NLR	CNL
Brassica cretica	KAF3531089.1	NLR	CNL
Brassica cretica	KAF3517760.1	NLR	CNL
Brassica cretica	KAF3516083.1	NLR	CNL
Brassica cretica	KAF3542362.1	NLR	CNL
Brassica cretica	KAF3521169.1	NLR	CNL
Brassica cretica	KAF3531129.1	NLR	CNL
Brassica cretica	KAF3596251.1	NLR	CNL
Brassica cretica	KAF3496834.1	NLR	CNL
Brassica cretica	KAF3550964.1	NLR	CNL

Brassica cretica	KAF3563011.1	NLR	CNL
Brassica cretica	KAF3564177.1	NLR	CNL
Brassica cretica	KAF3527492.1	NLR	CNL
Brassica cretica	KAF3566285.1	NLR	CNL
Brassica cretica	KAF3496263.1	NLR	CNL
Brassica cretica	KAF3576107.1	NLR	CNL
Brassica cretica	KAF3531911.1	NLR	CNL
Brassica cretica	KAF3567412.1	NLR	CNL
Brassica cretica	KAF3594269.1	NLR	CNL
Brassica cretica	KAF3597564.1	NLR	CNL
Brassica cretica	KAF3493463.1	NLR	CNL
Brassica cretica	KAF3579304.1	NLR	CNL
Brassica cretica	KAF3592429.1	NLR	CNL
Brassica cretica	KAF3564692.1	NLR	NBS
Brassica cretica	KAF3609115.1	NLR	NBS
Brassica cretica	KAF3497482.1	NLR	NBS
Brassica cretica	KAF3518230.1	NLR	NBS
Brassica cretica	KAF3566195.1	NLR	NBS
Brassica cretica	KAF3528543.1	NLR	NBS
Brassica cretica	KAF3519003.1	NLR	NBS
Brassica cretica	KAF3493296.1	NLR	NBS
Brassica cretica	KAF3517048.1	NLR	NBS
Brassica cretica	KAF3552568.1	NLR	NBS
Brassica cretica	KAF3609116.1	NLR	NBS
Brassica cretica	KAF3562520.1	NLR	NBS
Brassica cretica	KAF3592482.1	NLR	NBS
Brassica cretica	KAF3495510.1	NLR	NBS
Brassica cretica	KAF3530560.1	NLR	NBS
Brassica cretica	KAF3547007.1	NLR	NBS
Brassica cretica	KAF3580003.1	NLR	NBS
Brassica cretica	KAF3516960.1	NLR	NBS
Brassica cretica	KAF3552164.1	NLR	NBS
Brassica cretica	KAF3527232.1	NLR	NBS
Brassica cretica	KAF3565479.1	NLR	NBS
Brassica cretica	KAF3550114.1	NLR	NBS
Brassica cretica	KAF3517766.1	NLR	NBS
Brassica cretica	KAF3567523.1	NLR	NBS
Brassica cretica	KAF3561286.1	NLR	NBS
Brassica cretica	KAF3497488.1	NLR	NBS
Brassica cretica	KAF3591862.1	NLR	NBS
Brassica cretica	KAF3564888.1	NLR	NBS
Brassica cretica	KAF3565695.1	NLR	NL
Brassica cretica	KAF3496302.1	NLR	NL
Brassica cretica	KAF3596191.1	NLR	NL
Brassica cretica	KAF3564697.1	NLR	NL
Brassica cretica	KAF3543640.1	NLR	NL
Brassica cretica	KAF3495506.1	NLR	NL
Brassica cretica	KAF3596196.1	NLR	NL
Brassica cretica	KAF3590505.1	NLR	NL
Brassica cretica	KAF3546785.1	NLR	NL
Brassica cretica	KAF3565073.1	NLR	NL
Brassica cretica	KAF3607697.1	NLR	NL
Brassica cretica	KAF3568705.1	NLR	NL
Brassica cretica	KAF3544173.1	NLR	NL
Brassica cretica	KAF3578869.1	NLR	NL
Brassica cretica	KAF3543398.1	NLR	NL
Brassica cretica	KAF3568473.1	NLR	NL
Brassica cretica	KAF3494519.1	NLR	NL
Brassica cretica	KAF3582276.1	NLR	NL
Brassica cretica	KAF3527319.1	NLR	NL
Brassica cretica	KAF3498255.1	NLR	NL
Brassica cretica	KAF3546528.1	NLR	NL

Brassica cretica	KAF3527233.1	NLR	NL
Brassica cretica	KAF3567325.1	NLR	NL
Brassica cretica	KAF3532710.1	NLR	NL
Brassica cretica	KAF3566504.1	NLR	NL
Brassica cretica	KAF3530454.1	NLR	NL
Brassica cretica	KAF3528549.1	NLR	NL
Brassica cretica	KAF3520289.1	NLR	NL
Brassica cretica	KAF3560671.1	NLR	NL
Brassica cretica	KAF3568104.1	NLR	NL
Brassica cretica	KAF3546722.1	NLR	NL
Brassica cretica	KAF3543048.1	NLR	NL
Brassica cretica	KAF3548145.1	NLR	NL
Brassica cretica	KAF3529291.1	NLR	NL
Brassica cretica	KAF3546097.1	NLR	NL
Brassica cretica	KAF3527235.1	NLR	NL
Brassica cretica	KAF3575497.1	NLR	NL
Brassica cretica	KAF3549348.1	NLR	NL
Brassica cretica	KAF3594610.1	NLR	NL
Brassica cretica	KAF3521170.1	NLR	NL
Brassica cretica	KAF3548547.1	NLR	NL
Brassica cretica	KAF3567875.1	NLR	NL
Brassica cretica	KAF3563457.1	NLR	NL
Brassica cretica	KAF3592220.1	NLR	NL
Brassica cretica	KAF3519034.1	NLR	NL
Brassica cretica	KAF3606031.1	NLR	NL
Brassica cretica	KAF3564885.1	NLR	NL
Brassica cretica	KAF3547008.1	NLR	NL
Brassica cretica	KAF3593132.1	NLR	NL
Brassica cretica	KAF3610903.1	NLR	NL
Brassica cretica	KAF3564691.1	NLR	NL
Brassica cretica	KAF3531620.1	NLR	NL
Brassica cretica	KAF3567302.1	NLR	NL
Brassica cretica	KAF3498111.1	NLR	NL
Brassica cretica	KAF3591671.1	NLR	NL
Brassica cretica	KAF3546786.1	NLR	NL
Brassica cretica	KAF3569282.1	NLR	NL
Brassica cretica	KAF3606819.1	NLR	NL
Brassica cretica	KAF3569280.1	NLR	NL
Brassica cretica	KAF3564113.1	NLR	NL
Brassica cretica	KAF3596919.1	NLR	NL
Brassica cretica	KAF3568451.1	NLR	NL
Brassica cretica	KAF3560565.1	NLR	NL
Brassica cretica	KAF3533427.1	NLR	NL
Brassica cretica	KAF3493888.1	NLR	NL
Brassica cretica	KAF3567662.1	NLR	NL
Brassica cretica	KAF3496261.1	NLR	NL
Brassica cretica	KAF3549138.1	NLR	NL
Brassica cretica	KAF3579404.1	NLR	NL
Brassica cretica	KAF3567395.1	NLR	NL
Brassica cretica	KAF3542303.1	NLR	NL
Brassica cretica	KAF3496083.1	NLR	NL
Brassica cretica	KAF3591133.1	NLR	NL
Brassica cretica	KAF3534215.1	NLR	NL
Brassica cretica	KAF3611537.1	NLR	NL
Brassica cretica	KAF3561134.1	NLR	NL
Brassica cretica	KAF3532444.1	NLR	Other-NLR
Brassica cretica	KAF3595301.1	NLR	Other-NLR
Brassica cretica	KAF3521051.1	NLR	Other-NLR
Brassica cretica	KAF3496476.1	NLR	Other-NLR
Brassica cretica	KAF3498623.1	NLR	Other-NLR
Brassica cretica	KAF3547879.1	NLR	Other-NLR
Brassica cretica	KAF3519002.1	NLR	TN

Brassica cretica	KAF3543644.1	NLR	TN
Brassica cretica	KAF3580195.1	NLR	TN
Brassica cretica	KAF3611543.1	NLR	TN
Brassica cretica	KAF3547908.1	NLR	TN
Brassica cretica	KAF3567144.1	NLR	TN
Brassica cretica	KAF3542213.1	NLR	TN
Brassica cretica	KAF3517359.1	NLR	TN
Brassica cretica	KAF3519854.1	NLR	TNL
Brassica cretica	KAF3552163.1	NLR	TNL
Brassica cretica	KAF3607898.1	NLR	TNL
Brassica cretica	KAF3575962.1	NLR	TNL
Brassica cretica	KAF3591138.1	NLR	TNL
Brassica cretica	KAF3564886.1	NLR	TNL
Brassica cretica	KAF3594265.1	NLR	TNL
Brassica cretica	KAF3596494.1	NLR	TNL
Brassica cretica	KAF3580395.1	NLR	TNL
Brassica cretica	KAF3519814.1	NLR	TNL
Brassica cretica	KAF3528035.1	NLR	TNL
Brassica cretica	KAF3564720.1	NLR	TNL
Brassica cretica	KAF3549399.1	NLR	TNL
Brassica cretica	KAF3493997.1	NLR	TNL
Brassica cretica	KAF3564694.1	NLR	TNL
Brassica cretica	KAF3593075.1	NLR	TNL
Brassica cretica	KAF3517358.1	NLR	TNL
Brassica cretica	KAF3593074.1	NLR	TNL
Brassica cretica	KAF3578478.1	NLR	TNL
Brassica cretica	KAF3544379.1	NLR	TNL
Brassica cretica	KAF3593072.1	NLR	TNL
Brassica cretica	KAF3530939.1	NLR	TNL
Brassica cretica	KAF3564688.1	NLR	TNL
Brassica cretica	KAF3533397.1	NLR	TNL
Brassica cretica	KAF3493994.1	NLR	TNL
Brassica cretica	KAF3566292.1	NLR	TNL
Brassica cretica	KAF3496464.1	NLR	TNL
Brassica cretica	KAF3542239.1	NLR	TX
Brassica cretica	KAF3516321.1	NLR	TX
Brassica cretica	KAF3590309.1	NLR	TX
Brassica cretica	KAF3611548.1	NLR	TX
Brassica cretica	KAF3594266.1	NLR	TX
Brassica cretica	KAF3563462.1	NLR	TX
Brassica cretica	KAF3531070.1	NLR	TX
Brassica cretica	KAF3516344.1	NLR	TX
Brassica cretica	KAF3611063.1	NLR	TX
Brassica cretica	KAF3533451.1	NLR	TX
Brassica cretica	KAF3533400.1	NLR	TX
Brassica cretica	KAF3575596.1	NLR	TX
Brassica cretica	KAF3518309.1	NLR	TX
Brassica cretica	KAF3521234.1	NLR	TX
Brassica cretica	KAF3543681.1	NLR	TX
Brassica cretica	KAF3563585.1	NLR	TX
Brassica cretica	KAF3542835.1	NLR	TX
Brassica cretica	KAF3593112.1	NLR	TX
Brassica cretica	KAF3533653.1	NLR	TX
Brassica cretica	KAF3568665.1	NLR	TX
Brassica cretica	KAF3604833.1	NLR	TX
Brassica cretica	KAF3533283.1	NLR	TX
Brassica cretica	KAF3563333.1	NLR	TX
Brassica cretica	KAF3528674.1	NLR	TX
Brassica cretica	KAF3595295.1	NLR	TX
Brassica cretica	KAF3550962.1	NLR	TX
Brassica cretica	KAF3565187.1	NLR	TX
Brassica cretica	KAF3590504.1	NLR	TX

Brassica cretica	KAF3593080.1	NLR	TX
Brassica cretica	KAF3594653.1	NLR	TX
Brassica cretica	KAF3498668.1	NLR	TX
Brassica cretica	KAF3563329.1	NLR	TX
Brassica cretica	KAF3533281.1	NLR	TX
Brassica cretica	KAF3564882.1	NLR	TX
Brassica cretica	KAF3549351.1	NLR	TX
Brassica cretica	KAF3493744.1	NLR	TX
Brassica cretica	KAF3550957.1	NLR	TX
Brassica cretica	KAF3610231.1	NLR	TX
Brassica cretica	KAF3561285.1	NLR	TX
Brassica cretica	KAF3534383.1	NLR	TX
Brassica cretica	KAF3548279.1	NLR	TX
Brassica cretica	KAF3492592.1	RLK	Other-RLK
Brassica cretica	KAF3492596.1	RLK	Other-RLK
Brassica cretica	KAF3492731.1	RLK	Other-RLK
Brassica cretica	KAF3492733.1	RLK	Other-RLK
Brassica cretica	KAF3492735.1	RLK	Other-RLK
Brassica cretica	KAF3492745.1	RLK	Other-RLK
Brassica cretica	KAF3492749.1	RLK	LRR
Brassica cretica	KAF3492751.1	RLK	Other-RLK
Brassica cretica	KAF3492933.1	RLK	Other-RLK
Brassica cretica	KAF3492934.1	RLK	Other-RLK
Brassica cretica	KAF3492935.1	RLK	Other-RLK
Brassica cretica	KAF3492967.1	RLK	LRR
Brassica cretica	KAF3493063.1	RLK	LRR
Brassica cretica	KAF3493186.1	RLK	Other-RLK
Brassica cretica	KAF3493333.1	RLK	Other-RLK
Brassica cretica	KAF3493439.1	RLK	Other-RLK
Brassica cretica	KAF3493548.1	RLK	Other-RLK
Brassica cretica	KAF3493574.1	RLK	Other-RLK
Brassica cretica	KAF3493737.1	RLK	Other-RLK
Brassica cretica	KAF3493818.1	RLK	LRR
Brassica cretica	KAF3493832.1	RLK	LRR
Brassica cretica	KAF3493920.1	RLK	Other-RLK
Brassica cretica	KAF3493992.1	RLK	Other-RLK
Brassica cretica	KAF3494148.1	RLK	LRR
Brassica cretica	KAF3494215.1	RLK	Other-RLK
Brassica cretica	KAF3494425.1	RLK	Other-RLK
Brassica cretica	KAF3494816.1	RLK	Other-RLK
Brassica cretica	KAF3495290.1	RLK	Other-RLK
Brassica cretica	KAF3495444.1	RLK	Other-RLK
Brassica cretica	KAF3495473.1	RLK	LRR
Brassica cretica	KAF3495560.1	RLK	Other-RLK
Brassica cretica	KAF3495581.1	RLK	LRR
Brassica cretica	KAF3495654.1	RLK	Other-RLK
Brassica cretica	KAF3495756.1	RLK	Other-RLK
Brassica cretica	KAF3495821.1	RLK	Other-RLK
Brassica cretica	KAF3496003.1	RLK	LRR
Brassica cretica	KAF3496166.1	RLK	Other-RLK
Brassica cretica	KAF3496194.1	RLK	Other-RLK
Brassica cretica	KAF3496246.1	RLK	Other-RLK
Brassica cretica	KAF3496353.1	RLK	LRR
Brassica cretica	KAF3496397.1	RLK	Other-RLK
Brassica cretica	KAF3496550.1	RLK	Other-RLK
Brassica cretica	KAF3496579.1	RLK	Other-RLK
Brassica cretica	KAF3496870.1	RLK	Other-RLK
Brassica cretica	KAF3496930.1	RLK	Other-RLK
Brassica cretica	KAF3497023.1	RLK	LRR
Brassica cretica	KAF3497077.1	RLK	LRR
Brassica cretica	KAF3497083.1	RLK	LRR
Brassica cretica	KAF3497287.1	RLK	Other-RLK

Brassica cretica	KAF3497563.1	RLK	LRR
Brassica cretica	KAF3497701.1	RLK	LRR
Brassica cretica	KAF3497883.1	RLK	Other-RLK
Brassica cretica	KAF3498026.1	RLK	LRR
Brassica cretica	KAF3498202.1	RLK	Other-RLK
Brassica cretica	KAF3498249.1	RLK	Other-RLK
Brassica cretica	KAF3498264.1	RLK	Other-RLK
Brassica cretica	KAF3498289.1	RLK	Other-RLK
Brassica cretica	KAF3498418.1	RLK	Other-RLK
Brassica cretica	KAF3498621.1	RLK	Other-RLK
Brassica cretica	KAF3498651.1	RLK	Other-RLK
Brassica cretica	KAF3498652.1	RLK	Other-RLK
Brassica cretica	KAF3498693.1	RLK	Other-RLK
Brassica cretica	KAF3498694.1	RLK	Other-RLK
Brassica cretica	KAF3498700.1	RLK	Other-RLK
Brassica cretica	KAF3498799.1	RLK	Other-RLK
Brassica cretica	KAF3498892.1	RLK	Other-RLK
Brassica cretica	KAF3498899.1	RLK	Other-RLK
Brassica cretica	KAF3515676.1	RLK	LRR
Brassica cretica	KAF3515833.1	RLK	LRR
Brassica cretica	KAF3515929.1	RLK	LRR
Brassica cretica	KAF3516086.1	RLK	Other-RLK
Brassica cretica	KAF3516499.1	RLK	Other-RLK
Brassica cretica	KAF3516545.1	RLK	Other-RLK
Brassica cretica	KAF3516572.1	RLK	LRR
Brassica cretica	KAF3516952.1	RLK	LRR
Brassica cretica	KAF3516999.1	RLK	LRR
Brassica cretica	KAF3517164.1	RLK	LRR
Brassica cretica	KAF3517169.1	RLK	LRR
Brassica cretica	KAF3517170.1	RLK	LRR
Brassica cretica	KAF3517306.1	RLK	LRR
Brassica cretica	KAF3517595.1	RLK	LRR
Brassica cretica	KAF3517682.1	RLK	Other-RLK
Brassica cretica	KAF3517683.1	RLK	Other-RLK
Brassica cretica	KAF3517711.1	RLK	Other-RLK
Brassica cretica	KAF3517820.1	RLK	Other-RLK
Brassica cretica	KAF3518026.1	RLK	Other-RLK
Brassica cretica	KAF3518185.1	RLK	LRR
Brassica cretica	KAF3518237.1	RLK	LRR
Brassica cretica	KAF3518269.1	RLK	LRR
Brassica cretica	KAF3518354.1	RLK	Other-RLK
Brassica cretica	KAF3518437.1	RLK	LRR
Brassica cretica	KAF3518606.1	RLK	Other-RLK
Brassica cretica	KAF3518747.1	RLK	LRR
Brassica cretica	KAF3518923.1	RLK	Other-RLK
Brassica cretica	KAF3518960.1	RLK	Other-RLK
Brassica cretica	KAF3519129.1	RLK	Other-RLK
Brassica cretica	KAF3519277.1	RLK	Other-RLK
Brassica cretica	KAF3519392.1	RLK	Other-RLK
Brassica cretica	KAF3519407.1	RLK	Other-RLK
Brassica cretica	KAF3519770.1	RLK	Other-RLK
Brassica cretica	KAF3519793.1	RLK	Other-RLK
Brassica cretica	KAF3519811.1	RLK	LRR
Brassica cretica	KAF3519887.1	RLK	LRR
Brassica cretica	KAF3519919.1	RLK	Other-RLK
Brassica cretica	KAF3519941.1	RLK	LRR
Brassica cretica	KAF3520061.1	RLK	Other-RLK
Brassica cretica	KAF3520244.1	RLK	LRR
Brassica cretica	KAF3520406.1	RLK	LRR
Brassica cretica	KAF3520527.1	RLK	Other-RLK
Brassica cretica	KAF3520640.1	RLK	Lysm
Brassica cretica	KAF3520641.1	RLK	Other-RLK

Brassica cretica	KAF3520776.1	RLK	LRR
Brassica cretica	KAF3520930.1	RLK	Other-RLK
Brassica cretica	KAF3520948.1	RLK	Other-RLK
Brassica cretica	KAF3521057.1	RLK	Other-RLK
Brassica cretica	KAF3527320.1	RLK	Other-RLK
Brassica cretica	KAF3527321.1	RLK	Other-RLK
Brassica cretica	KAF3527324.1	RLK	Other-RLK
Brassica cretica	KAF3527376.1	RLK	Other-RLK
Brassica cretica	KAF3527487.1	RLK	LRR
Brassica cretica	KAF3527555.1	RLK	LRR
Brassica cretica	KAF3528003.1	RLK	Other-RLK
Brassica cretica	KAF3528024.1	RLK	Other-RLK
Brassica cretica	KAF3528097.1	RLK	Other-RLK
Brassica cretica	KAF3528149.1	RLK	Other-RLK
Brassica cretica	KAF3528741.1	RLK	LRR
Brassica cretica	KAF3528749.1	RLK	Other-RLK
Brassica cretica	KAF3528879.1	RLK	Other-RLK
Brassica cretica	KAF3528964.1	RLK	LRR
Brassica cretica	KAF3529063.1	RLK	LRR
Brassica cretica	KAF3529220.1	RLK	Other-RLK
Brassica cretica	KAF3529224.1	RLK	Other-RLK
Brassica cretica	KAF3529305.1	RLK	LRR
Brassica cretica	KAF3529346.1	RLK	LRR
Brassica cretica	KAF3529350.1	RLK	Other-RLK
Brassica cretica	KAF3529368.1	RLK	Other-RLK
Brassica cretica	KAF3529401.1	RLK	Other-RLK
Brassica cretica	KAF3529402.1	RLK	Other-RLK
Brassica cretica	KAF3529537.1	RLK	Other-RLK
Brassica cretica	KAF3529538.1	RLK	Other-RLK
Brassica cretica	KAF3529579.1	RLK	Other-RLK
Brassica cretica	KAF3529641.1	RLK	Other-RLK
Brassica cretica	KAF3529669.1	RLK	Other-RLK
Brassica cretica	KAF3529787.1	RLK	Other-RLK
Brassica cretica	KAF3530016.1	RLK	Other-RLK
Brassica cretica	KAF3530206.1	RLK	LRR
Brassica cretica	KAF3530240.1	RLK	LRR
Brassica cretica	KAF3530338.1	RLK	LRR
Brassica cretica	KAF3530401.1	RLK	Other-RLK
Brassica cretica	KAF3530412.1	RLK	LRR
Brassica cretica	KAF3530441.1	RLK	LRR
Brassica cretica	KAF3530536.1	RLK	Other-RLK
Brassica cretica	KAF3530598.1	RLK	Other-RLK
Brassica cretica	KAF3530599.1	RLK	Other-RLK
Brassica cretica	KAF3530670.1	RLK	LRR
Brassica cretica	KAF3530718.1	RLK	Other-RLK
Brassica cretica	KAF3530720.1	RLK	Other-RLK
Brassica cretica	KAF3531120.1	RLK	LRR
Brassica cretica	KAF3531197.1	RLK	Other-RLK
Brassica cretica	KAF3531306.1	RLK	LRR
Brassica cretica	KAF3531321.1	RLK	LRR
Brassica cretica	KAF3531340.1	RLK	LRR
Brassica cretica	KAF3531393.1	RLK	Other-RLK
Brassica cretica	KAF3531394.1	RLK	Other-RLK
Brassica cretica	KAF3531405.1	RLK	Other-RLK
Brassica cretica	KAF3531409.1	RLK	Other-RLK
Brassica cretica	KAF3531506.1	RLK	Other-RLK
Brassica cretica	KAF3531590.1	RLK	LRR
Brassica cretica	KAF3531635.1	RLK	LRR
Brassica cretica	KAF3531701.1	RLK	Other-RLK
Brassica cretica	KAF3531702.1	RLK	Other-RLK
Brassica cretica	KAF3531769.1	RLK	LRR
Brassica cretica	KAF3532193.1	RLK	Other-RLK

Brassica cretica	KAF3532197.1	RLK	Other-RLK
Brassica cretica	KAF3532250.1	RLK	Other-RLK
Brassica cretica	KAF3532566.1	RLK	Other-RLK
Brassica cretica	KAF3532682.1	RLK	LRR
Brassica cretica	KAF3532727.1	RLK	Other-RLK
Brassica cretica	KAF3532813.1	RLK	LRR
Brassica cretica	KAF3533106.1	RLK	LRR
Brassica cretica	KAF3533454.1	RLK	Other-RLK
Brassica cretica	KAF3533455.1	RLK	Other-RLK
Brassica cretica	KAF3533742.1	RLK	LRR
Brassica cretica	KAF3533744.1	RLK	Other-RLK
Brassica cretica	KAF3534109.1	RLK	Other-RLK
Brassica cretica	KAF3534120.1	RLK	Other-RLK
Brassica cretica	KAF3534161.1	RLK	Other-RLK
Brassica cretica	KAF3534207.1	RLK	LRR
Brassica cretica	KAF3534208.1	RLK	LRR
Brassica cretica	KAF3534255.1	RLK	Other-RLK
Brassica cretica	KAF3534307.1	RLK	Other-RLK
Brassica cretica	KAF3534397.1	RLK	LRR
Brassica cretica	KAF3534726.1	RLK	Other-RLK
Brassica cretica	KAF3534760.1	RLK	LRR
Brassica cretica	KAF3534780.1	RLK	Other-RLK
Brassica cretica	KAF3534896.1	RLK	Other-RLK
Brassica cretica	KAF3534991.1	RLK	LRR
Brassica cretica	KAF3542231.1	RLK	Other-RLK
Brassica cretica	KAF3542552.1	RLK	Other-RLK
Brassica cretica	KAF3542635.1	RLK	Other-RLK
Brassica cretica	KAF3542856.1	RLK	LRR
Brassica cretica	KAF3542884.1	RLK	Other-RLK
Brassica cretica	KAF3543004.1	RLK	Other-RLK
Brassica cretica	KAF3543304.1	RLK	Other-RLK
Brassica cretica	KAF3543395.1	RLK	LRR
Brassica cretica	KAF3543401.1	RLK	LRR
Brassica cretica	KAF3543503.1	RLK	Other-RLK
Brassica cretica	KAF3543538.1	RLK	Other-RLK
Brassica cretica	KAF3543659.1	RLK	Other-RLK
Brassica cretica	KAF3543661.1	RLK	Other-RLK
Brassica cretica	KAF3543819.1	RLK	Other-RLK
Brassica cretica	KAF3544014.1	RLK	Other-RLK
Brassica cretica	KAF3544176.1	RLK	LRR
Brassica cretica	KAF3544326.1	RLK	LRR
Brassica cretica	KAF3544482.1	RLK	Other-RLK
Brassica cretica	KAF3544600.1	RLK	LRR
Brassica cretica	KAF3544714.1	RLK	Other-RLK
Brassica cretica	KAF3544726.1	RLK	Other-RLK
Brassica cretica	KAF3544740.1	RLK	Other-RLK
Brassica cretica	KAF3544901.1	RLK	Other-RLK
Brassica cretica	KAF3544905.1	RLK	Other-RLK
Brassica cretica	KAF3544906.1	RLK	Other-RLK
Brassica cretica	KAF3544907.1	RLK	Other-RLK
Brassica cretica	KAF3545061.1	RLK	Other-RLK
Brassica cretica	KAF3545258.1	RLK	Other-RLK
Brassica cretica	KAF3545319.1	RLK	Other-RLK
Brassica cretica	KAF3545417.1	RLK	LRR
Brassica cretica	KAF3545426.1	RLK	Other-RLK
Brassica cretica	KAF3545513.1	RLK	Other-RLK
Brassica cretica	KAF3545599.1	RLK	LRR
Brassica cretica	KAF3545637.1	RLK	Other-RLK
Brassica cretica	KAF3545713.1	RLK	Other-RLK
Brassica cretica	KAF3545767.1	RLK	LRR
Brassica cretica	KAF3545858.1	RLK	Other-RLK
Brassica cretica	KAF3545892.1	RLK	LRR

Brassica cretica	KAF3546006.1	RLK	LRR
Brassica cretica	KAF3546043.1	RLK	LRR
Brassica cretica	KAF3546077.1	RLK	Other-RLK
Brassica cretica	KAF3546166.1	RLK	Other-RLK
Brassica cretica	KAF3546222.1	RLK	Other-RLK
Brassica cretica	KAF3546267.1	RLK	Other-RLK
Brassica cretica	KAF3546433.1	RLK	LRR
Brassica cretica	KAF3546640.1	RLK	Other-RLK
Brassica cretica	KAF3546646.1	RLK	Other-RLK
Brassica cretica	KAF3546697.1	RLK	Other-RLK
Brassica cretica	KAF3546747.1	RLK	Other-RLK
Brassica cretica	KAF3546825.1	RLK	LRR
Brassica cretica	KAF3546836.1	RLK	LRR
Brassica cretica	KAF3546841.1	RLK	LRR
Brassica cretica	KAF3546970.1	RLK	Other-RLK
Brassica cretica	KAF3547175.1	RLK	Other-RLK
Brassica cretica	KAF3547271.1	RLK	Other-RLK
Brassica cretica	KAF3547440.1	RLK	LRR
Brassica cretica	KAF3547513.1	RLK	Other-RLK
Brassica cretica	KAF3547525.1	RLK	LRR
Brassica cretica	KAF3547528.1	RLK	Other-RLK
Brassica cretica	KAF3547618.1	RLK	Other-RLK
Brassica cretica	KAF3547657.1	RLK	Other-RLK
Brassica cretica	KAF3547660.1	RLK	Other-RLK
Brassica cretica	KAF3547678.1	RLK	Other-RLK
Brassica cretica	KAF3547685.1	RLK	LRR
Brassica cretica	KAF3547842.1	RLK	Other-RLK
Brassica cretica	KAF3547917.1	RLK	LRR
Brassica cretica	KAF3548122.1	RLK	Other-RLK
Brassica cretica	KAF3548255.1	RLK	Other-RLK
Brassica cretica	KAF3548407.1	RLK	Other-RLK
Brassica cretica	KAF3548579.1	RLK	Other-RLK
Brassica cretica	KAF3548747.1	RLK	LRR
Brassica cretica	KAF3548895.1	RLK	Other-RLK
Brassica cretica	KAF3548896.1	RLK	Other-RLK
Brassica cretica	KAF3549092.1	RLK	Other-RLK
Brassica cretica	KAF3549183.1	RLK	Other-RLK
Brassica cretica	KAF3549227.1	RLK	Other-RLK
Brassica cretica	KAF3549228.1	RLK	Other-RLK
Brassica cretica	KAF3549230.1	RLK	Other-RLK
Brassica cretica	KAF3549445.1	RLK	Other-RLK
Brassica cretica	KAF3549501.1	RLK	Other-RLK
Brassica cretica	KAF3549517.1	RLK	Other-RLK
Brassica cretica	KAF3549718.1	RLK	LRR
Brassica cretica	KAF3549720.1	RLK	LRR
Brassica cretica	KAF3549721.1	RLK	LRR
Brassica cretica	KAF3549823.1	RLK	Other-RLK
Brassica cretica	KAF3549886.1	RLK	LRR
Brassica cretica	KAF3550204.1	RLK	Other-RLK
Brassica cretica	KAF3550205.1	RLK	Other-RLK
Brassica cretica	KAF3550207.1	RLK	Other-RLK
Brassica cretica	KAF3550237.1	RLK	Other-RLK
Brassica cretica	KAF3550258.1	RLK	LRR
Brassica cretica	KAF3550268.1	RLK	Other-RLK
Brassica cretica	KAF3550289.1	RLK	Other-RLK
Brassica cretica	KAF3550535.1	RLK	LRR
Brassica cretica	KAF3550654.1	RLK	LRR
Brassica cretica	KAF3550746.1	RLK	Other-RLK
Brassica cretica	KAF3550987.1	RLK	LRR
Brassica cretica	KAF3551134.1	RLK	LRR
Brassica cretica	KAF3551208.1	RLK	LRR
Brassica cretica	KAF3551215.1	RLK	LRR

Brassica cretica	KAF3551216.1	RLK	LRR
Brassica cretica	KAF3551520.1	RLK	LRR
Brassica cretica	KAF3551801.1	RLK	LRR
Brassica cretica	KAF3551804.1	RLK	LRR
Brassica cretica	KAF3551806.1	RLK	LRR
Brassica cretica	KAF3552088.1	RLK	LRR
Brassica cretica	KAF3552125.1	RLK	LRR
Brassica cretica	KAF3552152.1	RLK	LRR
Brassica cretica	KAF3552369.1	RLK	LRR
Brassica cretica	KAF3552472.1	RLK	Other-RLK
Brassica cretica	KAF3560512.1	RLK	Other-RLK
Brassica cretica	KAF3560562.1	RLK	LRR
Brassica cretica	KAF3560572.1	RLK	Other-RLK
Brassica cretica	KAF3560574.1	RLK	Other-RLK
Brassica cretica	KAF3560652.1	RLK	Other-RLK
Brassica cretica	KAF3560654.1	RLK	Other-RLK
Brassica cretica	KAF3561033.1	RLK	Other-RLK
Brassica cretica	KAF3561064.1	RLK	LRR
Brassica cretica	KAF3561149.1	RLK	Other-RLK
Brassica cretica	KAF3561395.1	RLK	Other-RLK
Brassica cretica	KAF3561444.1	RLK	Other-RLK
Brassica cretica	KAF3561469.1	RLK	Other-RLK
Brassica cretica	KAF3561519.1	RLK	Other-RLK
Brassica cretica	KAF3561557.1	RLK	Other-RLK
Brassica cretica	KAF3561578.1	RLK	LRR
Brassica cretica	KAF3561775.1	RLK	LRR
Brassica cretica	KAF3562030.1	RLK	Other-RLK
Brassica cretica	KAF3562133.1	RLK	LRR
Brassica cretica	KAF3562170.1	RLK	Other-RLK
Brassica cretica	KAF3562210.1	RLK	Other-RLK
Brassica cretica	KAF3562340.1	RLK	Other-RLK
Brassica cretica	KAF3562511.1	RLK	Other-RLK
Brassica cretica	KAF3562548.1	RLK	Other-RLK
Brassica cretica	KAF3562760.1	RLK	Other-RLK
Brassica cretica	KAF3562943.1	RLK	LRR
Brassica cretica	KAF3563235.1	RLK	Other-RLK
Brassica cretica	KAF3563391.1	RLK	LRR
Brassica cretica	KAF3563497.1	RLK	LRR
Brassica cretica	KAF3563499.1	RLK	Other-RLK
Brassica cretica	KAF3563679.1	RLK	Other-RLK
Brassica cretica	KAF3563680.1	RLK	Other-RLK
Brassica cretica	KAF3563681.1	RLK	Other-RLK
Brassica cretica	KAF3563715.1	RLK	LRR
Brassica cretica	KAF3563725.1	RLK	Other-RLK
Brassica cretica	KAF3563845.1	RLK	Other-RLK
Brassica cretica	KAF3564141.1	RLK	LRR
Brassica cretica	KAF3564399.1	RLK	LRR
Brassica cretica	KAF3564458.1	RLK	Other-RLK
Brassica cretica	KAF3564635.1	RLK	LRR
Brassica cretica	KAF3564661.1	RLK	Other-RLK
Brassica cretica	KAF3564872.1	RLK	LRR
Brassica cretica	KAF3564878.1	RLK	LRR
Brassica cretica	KAF3565048.1	RLK	Other-RLK
Brassica cretica	KAF3565049.1	RLK	LRR
Brassica cretica	KAF3565055.1	RLK	Other-RLK
Brassica cretica	KAF3565146.1	RLK	LRR
Brassica cretica	KAF3565177.1	RLK	Other-RLK
Brassica cretica	KAF3565181.1	RLK	Other-RLK
Brassica cretica	KAF3565184.1	RLK	Other-RLK
Brassica cretica	KAF3565185.1	RLK	Other-RLK
Brassica cretica	KAF3565405.1	RLK	Other-RLK
Brassica cretica	KAF3565410.1	RLK	Other-RLK

Brassica cretica	KAF3565537.1	RLK	Other-RLK
Brassica cretica	KAF3565641.1	RLK	Other-RLK
Brassica cretica	KAF3565656.1	RLK	Other-RLK
Brassica cretica	KAF3565805.1	RLK	Other-RLK
Brassica cretica	KAF3566013.1	RLK	Other-RLK
Brassica cretica	KAF3566190.1	RLK	LRR
Brassica cretica	KAF3566206.1	RLK	Other-RLK
Brassica cretica	KAF3566357.1	RLK	Other-RLK
Brassica cretica	KAF3566359.1	RLK	Other-RLK
Brassica cretica	KAF3566407.1	RLK	LRR
Brassica cretica	KAF3566420.1	RLK	LRR
Brassica cretica	KAF3566664.1	RLK	Other-RLK
Brassica cretica	KAF3566685.1	RLK	Other-RLK
Brassica cretica	KAF3566800.1	RLK	Other-RLK
Brassica cretica	KAF3566982.1	RLK	Other-RLK
Brassica cretica	KAF3567039.1	RLK	LRR
Brassica cretica	KAF3567156.1	RLK	Other-RLK
Brassica cretica	KAF3567231.1	RLK	Other-RLK
Brassica cretica	KAF3567250.1	RLK	LRR
Brassica cretica	KAF3567301.1	RLK	Other-RLK
Brassica cretica	KAF3567358.1	RLK	Other-RLK
Brassica cretica	KAF3567385.1	RLK	LRR
Brassica cretica	KAF3567444.1	RLK	LRR
Brassica cretica	KAF3567467.1	RLK	Other-RLK
Brassica cretica	KAF3567647.1	RLK	LRR
Brassica cretica	KAF3567648.1	RLK	LRR
Brassica cretica	KAF3567894.1	RLK	Other-RLK
Brassica cretica	KAF3568114.1	RLK	LRR
Brassica cretica	KAF3568267.1	RLK	LRR
Brassica cretica	KAF3568523.1	RLK	Other-RLK
Brassica cretica	KAF3568533.1	RLK	Other-RLK
Brassica cretica	KAF3568610.1	RLK	Other-RLK
Brassica cretica	KAF3568699.1	RLK	Other-RLK
Brassica cretica	KAF3568938.1	RLK	LRR
Brassica cretica	KAF3569083.1	RLK	LRR
Brassica cretica	KAF3569266.1	RLK	Other-RLK
Brassica cretica	KAF3569306.1	RLK	Other-RLK
Brassica cretica	KAF3569373.1	RLK	LRR
Brassica cretica	KAF3569515.1	RLK	Other-RLK
Brassica cretica	KAF3575639.1	RLK	Other-RLK
Brassica cretica	KAF3575689.1	RLK	Other-RLK
Brassica cretica	KAF3575692.1	RLK	Other-RLK
Brassica cretica	KAF3575772.1	RLK	Other-RLK
Brassica cretica	KAF3575849.1	RLK	LRR
Brassica cretica	KAF3576064.1	RLK	LRR
Brassica cretica	KAF3576067.1	RLK	LRR
Brassica cretica	KAF3576292.1	RLK	LRR
Brassica cretica	KAF3576511.1	RLK	Other-RLK
Brassica cretica	KAF3576630.1	RLK	Other-RLK
Brassica cretica	KAF3576748.1	RLK	Other-RLK
Brassica cretica	KAF3576827.1	RLK	Other-RLK
Brassica cretica	KAF3577217.1	RLK	Other-RLK
Brassica cretica	KAF3577276.1	RLK	LRR
Brassica cretica	KAF3577283.1	RLK	Other-RLK
Brassica cretica	KAF3577290.1	RLK	Other-RLK
Brassica cretica	KAF3577550.1	RLK	Other-RLK
Brassica cretica	KAF3577558.1	RLK	Lysm
Brassica cretica	KAF3577740.1	RLK	Other-RLK
Brassica cretica	KAF3577991.1	RLK	LRR
Brassica cretica	KAF3578012.1	RLK	Other-RLK
Brassica cretica	KAF3578022.1	RLK	Other-RLK
Brassica cretica	KAF3578117.1	RLK	Other-RLK

Brassica cretica	KAF3578206.1	RLK	Other-RLK
Brassica cretica	KAF3578558.1	RLK	Other-RLK
Brassica cretica	KAF3578564.1	RLK	Other-RLK
Brassica cretica	KAF3578575.1	RLK	LRR
Brassica cretica	KAF3578797.1	RLK	Other-RLK
Brassica cretica	KAF3578898.1	RLK	Other-RLK
Brassica cretica	KAF3578905.1	RLK	LRR
Brassica cretica	KAF3578912.1	RLK	LRR
Brassica cretica	KAF3579069.1	RLK	LRR
Brassica cretica	KAF3579186.1	RLK	Other-RLK
Brassica cretica	KAF3579196.1	RLK	LRR
Brassica cretica	KAF3579334.1	RLK	Other-RLK
Brassica cretica	KAF3579457.1	RLK	LRR
Brassica cretica	KAF3579469.1	RLK	Other-RLK
Brassica cretica	KAF3579504.1	RLK	Other-RLK
Brassica cretica	KAF3579525.1	RLK	LRR
Brassica cretica	KAF3579608.1	RLK	Other-RLK
Brassica cretica	KAF3579734.1	RLK	Other-RLK
Brassica cretica	KAF3579800.1	RLK	Other-RLK
Brassica cretica	KAF3579851.1	RLK	LRR
Brassica cretica	KAF3579852.1	RLK	Other-RLK
Brassica cretica	KAF3579889.1	RLK	Other-RLK
Brassica cretica	KAF3579909.1	RLK	Other-RLK
Brassica cretica	KAF3580065.1	RLK	Other-RLK
Brassica cretica	KAF3580282.1	RLK	Other-RLK
Brassica cretica	KAF3580383.1	RLK	LRR
Brassica cretica	KAF3580466.1	RLK	Other-RLK
Brassica cretica	KAF3580522.1	RLK	LRR
Brassica cretica	KAF3580643.1	RLK	LRR
Brassica cretica	KAF3580951.1	RLK	LRR
Brassica cretica	KAF3581175.1	RLK	Other-RLK
Brassica cretica	KAF3581323.1	RLK	Other-RLK
Brassica cretica	KAF3581377.1	RLK	LRR
Brassica cretica	KAF3581405.1	RLK	Other-RLK
Brassica cretica	KAF3581439.1	RLK	Other-RLK
Brassica cretica	KAF3581517.1	RLK	LRR
Brassica cretica	KAF3581547.1	RLK	Other-RLK
Brassica cretica	KAF3581568.1	RLK	LRR
Brassica cretica	KAF3581707.1	RLK	Other-RLK
Brassica cretica	KAF3581847.1	RLK	LRR
Brassica cretica	KAF3581968.1	RLK	Other-RLK
Brassica cretica	KAF3582157.1	RLK	LRR
Brassica cretica	KAF3582274.1	RLK	Other-RLK
Brassica cretica	KAF3582602.1	RLK	Other-RLK
Brassica cretica	KAF3582635.1	RLK	Other-RLK
Brassica cretica	KAF3582684.1	RLK	Other-RLK
Brassica cretica	KAF3582773.1	RLK	Other-RLK
Brassica cretica	KAF3582824.1	RLK	Other-RLK
Brassica cretica	KAF3582977.1	RLK	Other-RLK
Brassica cretica	KAF3583049.1	RLK	LRR
Brassica cretica	KAF3590220.1	RLK	Other-RLK
Brassica cretica	KAF3590225.1	RLK	LRR
Brassica cretica	KAF3590229.1	RLK	LRR
Brassica cretica	KAF3590590.1	RLK	Other-RLK
Brassica cretica	KAF3590620.1	RLK	Other-RLK
Brassica cretica	KAF3590644.1	RLK	Other-RLK
Brassica cretica	KAF3590807.1	RLK	Other-RLK
Brassica cretica	KAF3591051.1	RLK	Other-RLK
Brassica cretica	KAF3591219.1	RLK	LRR
Brassica cretica	KAF3591238.1	RLK	LRR
Brassica cretica	KAF3591262.1	RLK	LRR
Brassica cretica	KAF3591292.1	RLK	LRR

Brassica cretica	KAF3591324.1	RLK	Other-RLK
Brassica cretica	KAF3591325.1	RLK	Other-RLK
Brassica cretica	KAF3591329.1	RLK	LRR
Brassica cretica	KAF3591550.1	RLK	Other-RLK
Brassica cretica	KAF3591613.1	RLK	Other-RLK
Brassica cretica	KAF3591810.1	RLK	Other-RLK
Brassica cretica	KAF3591952.1	RLK	Other-RLK
Brassica cretica	KAF3591956.1	RLK	Other-RLK
Brassica cretica	KAF3591957.1	RLK	Other-RLK
Brassica cretica	KAF3591958.1	RLK	Other-RLK
Brassica cretica	KAF3591959.1	RLK	Other-RLK
Brassica cretica	KAF3591961.1	RLK	LRR
Brassica cretica	KAF3591997.1	RLK	LRR
Brassica cretica	KAF3592238.1	RLK	Other-RLK
Brassica cretica	KAF3592381.1	RLK	LRR
Brassica cretica	KAF3592477.1	RLK	Other-RLK
Brassica cretica	KAF3592750.1	RLK	Other-RLK
Brassica cretica	KAF3593012.1	RLK	LRR
Brassica cretica	KAF3593070.1	RLK	Other-RLK
Brassica cretica	KAF3593223.1	RLK	Other-RLK
Brassica cretica	KAF3593432.1	RLK	Other-RLK
Brassica cretica	KAF3593658.1	RLK	Other-RLK
Brassica cretica	KAF3593750.1	RLK	Other-RLK
Brassica cretica	KAF3593886.1	RLK	LRR
Brassica cretica	KAF3594039.1	RLK	Other-RLK
Brassica cretica	KAF3594063.1	RLK	LRR
Brassica cretica	KAF3594068.1	RLK	LRR
Brassica cretica	KAF3594134.1	RLK	LRR
Brassica cretica	KAF3594145.1	RLK	LRR
Brassica cretica	KAF3594381.1	RLK	Other-RLK
Brassica cretica	KAF3594382.1	RLK	LRR
Brassica cretica	KAF3594541.1	RLK	Other-RLK
Brassica cretica	KAF3594672.1	RLK	Other-RLK
Brassica cretica	KAF3594681.1	RLK	LRR
Brassica cretica	KAF3594687.1	RLK	LRR
Brassica cretica	KAF3594897.1	RLK	LRR
Brassica cretica	KAF3594900.1	RLK	Other-RLK
Brassica cretica	KAF3594942.1	RLK	LRR
Brassica cretica	KAF3594973.1	RLK	Other-RLK
Brassica cretica	KAF3594980.1	RLK	Other-RLK
Brassica cretica	KAF3595027.1	RLK	LRR
Brassica cretica	KAF3595050.1	RLK	Other-RLK
Brassica cretica	KAF3595485.1	RLK	LRR
Brassica cretica	KAF3595551.1	RLK	Other-RLK
Brassica cretica	KAF3595681.1	RLK	LRR
Brassica cretica	KAF3595709.1	RLK	LRR
Brassica cretica	KAF3595813.1	RLK	LRR
Brassica cretica	KAF3595854.1	RLK	Other-RLK
Brassica cretica	KAF3595859.1	RLK	Other-RLK
Brassica cretica	KAF3595966.1	RLK	LRR
Brassica cretica	KAF3596107.1	RLK	LRR
Brassica cretica	KAF3596246.1	RLK	Other-RLK
Brassica cretica	KAF3596346.1	RLK	LRR
Brassica cretica	KAF3596398.1	RLK	LRR
Brassica cretica	KAF3596599.1	RLK	Other-RLK
Brassica cretica	KAF3596614.1	RLK	Other-RLK
Brassica cretica	KAF3596617.1	RLK	Other-RLK
Brassica cretica	KAF3596659.1	RLK	Other-RLK
Brassica cretica	KAF3596668.1	RLK	Other-RLK
Brassica cretica	KAF3596767.1	RLK	LRR
Brassica cretica	KAF3596777.1	RLK	Other-RLK
Brassica cretica	KAF3596797.1	RLK	LRR

Brassica cretica	KAF3596816.1	RLK	Other-RLK
Brassica cretica	KAF3596851.1	RLK	Other-RLK
Brassica cretica	KAF3597060.1	RLK	LRR
Brassica cretica	KAF3597102.1	RLK	LRR
Brassica cretica	KAF3597152.1	RLK	LRR
Brassica cretica	KAF3597652.1	RLK	Other-RLK
Brassica cretica	KAF3597752.1	RLK	LRR
Brassica cretica	KAF3597836.1	RLK	Other-RLK
Brassica cretica	KAF3597898.1	RLK	LRR
Brassica cretica	KAF3598012.1	RLK	LRR
Brassica cretica	KAF3598086.1	RLK	Other-RLK
Brassica cretica	KAF3598156.1	RLK	LRR
Brassica cretica	KAF3604826.1	RLK	Other-RLK
Brassica cretica	KAF3604840.1	RLK	Other-RLK
Brassica cretica	KAF3604915.1	RLK	Other-RLK
Brassica cretica	KAF3605237.1	RLK	LRR
Brassica cretica	KAF3605528.1	RLK	Other-RLK
Brassica cretica	KAF3605539.1	RLK	Other-RLK
Brassica cretica	KAF3605730.1	RLK	Other-RLK
Brassica cretica	KAF3605757.1	RLK	LRR
Brassica cretica	KAF3605812.1	RLK	Other-RLK
Brassica cretica	KAF3605881.1	RLK	Other-RLK
Brassica cretica	KAF3605888.1	RLK	LRR
Brassica cretica	KAF3605995.1	RLK	LRR
Brassica cretica	KAF3606163.1	RLK	Other-RLK
Brassica cretica	KAF3606164.1	RLK	Other-RLK
Brassica cretica	KAF3606261.1	RLK	LRR
Brassica cretica	KAF3606329.1	RLK	Other-RLK
Brassica cretica	KAF3606718.1	RLK	LRR
Brassica cretica	KAF3606752.1	RLK	Other-RLK
Brassica cretica	KAF3606881.1	RLK	LRR
Brassica cretica	KAF3606939.1	RLK	Other-RLK
Brassica cretica	KAF3607186.1	RLK	LRR
Brassica cretica	KAF3607204.1	RLK	Other-RLK
Brassica cretica	KAF3607292.1	RLK	Other-RLK
Brassica cretica	KAF3607675.1	RLK	LRR
Brassica cretica	KAF3607908.1	RLK	LRR
Brassica cretica	KAF3607936.1	RLK	LRR
Brassica cretica	KAF3608273.1	RLK	LRR
Brassica cretica	KAF3608474.1	RLK	LRR
Brassica cretica	KAF3608549.1	RLK	Other-RLK
Brassica cretica	KAF3609052.1	RLK	LRR
Brassica cretica	KAF3609185.1	RLK	LRR
Brassica cretica	KAF3609405.1	RLK	LRR
Brassica cretica	KAF3609485.1	RLK	LRR
Brassica cretica	KAF3609513.1	RLK	Other-RLK
Brassica cretica	KAF3609522.1	RLK	Other-RLK
Brassica cretica	KAF3609537.1	RLK	Other-RLK
Brassica cretica	KAF3609633.1	RLK	Other-RLK
Brassica cretica	KAF3609786.1	RLK	Other-RLK
Brassica cretica	KAF3609906.1	RLK	Other-RLK
Brassica cretica	KAF3609935.1	RLK	Other-RLK
Brassica cretica	KAF3610095.1	RLK	Other-RLK
Brassica cretica	KAF3610674.1	RLK	LRR
Brassica cretica	KAF3610783.1	RLK	Other-RLK
Brassica cretica	KAF3610809.1	RLK	Other-RLK
Brassica cretica	KAF3610851.1	RLK	Lysm
Brassica cretica	KAF3610959.1	RLK	LRR
Brassica cretica	KAF3611012.1	RLK	Other-RLK
Brassica cretica	KAF3611101.1	RLK	Other-RLK
Brassica cretica	KAF3611546.1	RLK	LRR
Brassica cretica	KAF3611659.1	RLK	LRR

Brassica cretica	KAF3611776.1	RLK	Other-RLK
Brassica cretica	KAF3611825.1	RLK	LRR
Brassica cretica	KAF3611901.1	RLK	Other-RLK
Brassica cretica	KAF3611902.1	RLK	Other-RLK
Brassica cretica	KAF3611970.1	RLK	Other-RLK
Brassica cretica	KAF3611993.1	RLK	Other-RLK
Brassica cretica	KAF3612069.1	RLK	Other-RLK
Brassica cretica	KAF3492795.1	RLP	LRR
Brassica cretica	KAF3492846.1	RLP	LRR
Brassica cretica	KAF3493125.1	RLP	Lysm
Brassica cretica	KAF3495299.1	RLP	LRR
Brassica cretica	KAF3495711.1	RLP	LRR
Brassica cretica	KAF3495939.1	RLP	LRR
Brassica cretica	KAF3496031.1	RLP	LRR
Brassica cretica	KAF3496187.1	RLP	LRR
Brassica cretica	KAF3496193.1	RLP	LRR
Brassica cretica	KAF3496538.1	RLP	LRR
Brassica cretica	KAF3496742.1	RLP	LRR
Brassica cretica	KAF3496993.1	RLP	LRR
Brassica cretica	KAF3497409.1	RLP	LRR
Brassica cretica	KAF3497625.1	RLP	LRR
Brassica cretica	KAF3497647.1	RLP	LRR
Brassica cretica	KAF3497651.1	RLP	LRR
Brassica cretica	KAF3497769.1	RLP	LRR
Brassica cretica	KAF3497809.1	RLP	LRR
Brassica cretica	KAF3498935.1	RLP	LRR
Brassica cretica	KAF3515489.1	RLP	LRR
Brassica cretica	KAF3515583.1	RLP	LRR
Brassica cretica	KAF3515587.1	RLP	LRR
Brassica cretica	KAF3515909.1	RLP	LRR
Brassica cretica	KAF3516628.1	RLP	LRR
Brassica cretica	KAF3517247.1	RLP	LRR
Brassica cretica	KAF3517249.1	RLP	LRR
Brassica cretica	KAF3517872.1	RLP	LRR
Brassica cretica	KAF3518061.1	RLP	LRR
Brassica cretica	KAF3519282.1	RLP	LRR
Brassica cretica	KAF3519381.1	RLP	LRR
Brassica cretica	KAF3519475.1	RLP	LRR
Brassica cretica	KAF3519685.1	RLP	LRR
Brassica cretica	KAF3520182.1	RLP	LRR
Brassica cretica	KAF3520760.1	RLP	LRR
Brassica cretica	KAF3520873.1	RLP	LRR
Brassica cretica	KAF3521027.1	RLP	LRR
Brassica cretica	KAF3527819.1	RLP	LRR
Brassica cretica	KAF3528544.1	RLP	LRR
Brassica cretica	KAF3528757.1	RLP	LRR
Brassica cretica	KAF3529312.1	RLP	LRR
Brassica cretica	KAF3529323.1	RLP	LRR
Brassica cretica	KAF3529880.1	RLP	LRR
Brassica cretica	KAF3531136.1	RLP	LRR
Brassica cretica	KAF3532173.1	RLP	LRR
Brassica cretica	KAF3532176.1	RLP	LRR
Brassica cretica	KAF3532638.1	RLP	LRR
Brassica cretica	KAF3532641.1	RLP	LRR
Brassica cretica	KAF3532646.1	RLP	LRR
Brassica cretica	KAF3533009.1	RLP	LRR
Brassica cretica	KAF3533676.1	RLP	LRR
Brassica cretica	KAF3542247.1	RLP	LRR
Brassica cretica	KAF3542341.1	RLP	LRR
Brassica cretica	KAF3543027.1	RLP	LRR
Brassica cretica	KAF3543527.1	RLP	LRR
Brassica cretica	KAF3544388.1	RLP	LRR

Brassica cretica	KAF3544496.1	RLP	LRR
Brassica cretica	KAF3544613.1	RLP	LRR
Brassica cretica	KAF3544789.1	RLP	LRR
Brassica cretica	KAF3545126.1	RLP	LRR
Brassica cretica	KAF3545844.1	RLP	LRR
Brassica cretica	KAF3545923.1	RLP	LRR
Brassica cretica	KAF3546057.1	RLP	LRR
Brassica cretica	KAF3546153.1	RLP	LRR
Brassica cretica	KAF3546461.1	RLP	LRR
Brassica cretica	KAF3547794.1	RLP	LRR
Brassica cretica	KAF3548035.1	RLP	LRR
Brassica cretica	KAF3548123.1	RLP	LRR
Brassica cretica	KAF3548840.1	RLP	LRR
Brassica cretica	KAF3549191.1	RLP	LRR
Brassica cretica	KAF3549193.1	RLP	LRR
Brassica cretica	KAF3550014.1	RLP	LRR
Brassica cretica	KAF3550447.1	RLP	LRR
Brassica cretica	KAF3550923.1	RLP	LRR
Brassica cretica	KAF3551131.1	RLP	LRR
Brassica cretica	KAF3551280.1	RLP	LRR
Brassica cretica	KAF3551561.1	RLP	LRR
Brassica cretica	KAF3551685.1	RLP	LRR
Brassica cretica	KAF3551908.1	RLP	LRR
Brassica cretica	KAF3551957.1	RLP	LRR
Brassica cretica	KAF3561189.1	RLP	LRR
Brassica cretica	KAF3561312.1	RLP	LRR
Brassica cretica	KAF3561686.1	RLP	LRR
Brassica cretica	KAF3562500.1	RLP	LRR
Brassica cretica	KAF3563475.1	RLP	LRR
Brassica cretica	KAF3563630.1	RLP	LRR
Brassica cretica	KAF3564526.1	RLP	LRR
Brassica cretica	KAF3565783.1	RLP	LRR
Brassica cretica	KAF3566287.1	RLP	LRR
Brassica cretica	KAF3567650.1	RLP	LRR
Brassica cretica	KAF3568442.1	RLP	LRR
Brassica cretica	KAF3568567.1	RLP	LRR
Brassica cretica	KAF3568573.1	RLP	LRR
Brassica cretica	KAF3569290.1	RLP	LRR
Brassica cretica	KAF3575513.1	RLP	LRR
Brassica cretica	KAF3576309.1	RLP	LRR
Brassica cretica	KAF3577152.1	RLP	LRR
Brassica cretica	KAF3577333.1	RLP	LRR
Brassica cretica	KAF3577513.1	RLP	LRR
Brassica cretica	KAF3577793.1	RLP	LRR
Brassica cretica	KAF3578311.1	RLP	LRR
Brassica cretica	KAF3578456.1	RLP	LRR
Brassica cretica	KAF3578983.1	RLP	LRR
Brassica cretica	KAF3579584.1	RLP	LRR
Brassica cretica	KAF3579687.1	RLP	LRR
Brassica cretica	KAF3580220.1	RLP	LRR
Brassica cretica	KAF3580346.1	RLP	LRR
Brassica cretica	KAF3580412.1	RLP	LRR
Brassica cretica	KAF3580635.1	RLP	LRR
Brassica cretica	KAF3581021.1	RLP	LRR
Brassica cretica	KAF3581148.1	RLP	LRR
Brassica cretica	KAF3582061.1	RLP	LRR
Brassica cretica	KAF3582636.1	RLP	LRR
Brassica cretica	KAF3590227.1	RLP	LRR
Brassica cretica	KAF3590332.1	RLP	LRR
Brassica cretica	KAF3590666.1	RLP	LRR
Brassica cretica	KAF3590951.1	RLP	LRR
Brassica cretica	KAF3592061.1	RLP	LRR

Brassica cretica	KAF3592110.1	RLP	LRR
Brassica cretica	KAF3592667.1	RLP	LRR
Brassica cretica	KAF3593524.1	RLP	LRR
Brassica cretica	KAF3595765.1	RLP	LRR
Brassica cretica	KAF3595768.1	RLP	LRR
Brassica cretica	KAF3597435.1	RLP	LRR
Brassica cretica	KAF3597616.1	RLP	LRR
Brassica cretica	KAF3605208.1	RLP	LRR
Brassica cretica	KAF3605889.1	RLP	LRR
Brassica cretica	KAF3605935.1	RLP	LRR
Brassica cretica	KAF3606052.1	RLP	LRR
Brassica cretica	KAF3606447.1	RLP	LRR
Brassica cretica	KAF3606720.1	RLP	LRR
Brassica cretica	KAF3608524.1	RLP	LRR
Brassica cretica	KAF3611002.1	RLP	LRR
Brassica cretica	KAF3611148.1	RLP	LRR
Brassica cretica	KAF3611536.1	RLP	LRR
Brassica cretica	KAF3611574.1	RLP	LRR
Brassica cretica	KAF3611668.1	RLP	Lysm
Brassica cretica	KAF3611753.1	RLP	LRR
Brassica cretica	KAF3612085.1	RLP	LRR

*note: Main RGA classes, NLR= nucleotide-binding site (NBS) -leucine rich repeats (LRR), RLK= receptor-like protein kinases (RLKs), RLP= receptor-like proteins. NLRs subclasses, CN= coiled-coil (CC)-NBS, CNL, NBS, NL= NBS-LRR, TNL= Toll/Interleukin-1 receptor (TIR)-NBS-LRR, TN= TIR-NBS, TX= TIR with unknown domains, Other-NLR= NLR with other domains; RLK subclasses, LRR-RLK, Lysin motif (Lysm)-RLK, Other (other-receptor)-RLK; RLP subclasses, LRR-RLP and Lysm-RLP.

Table S2. List of resistance gene analogs (RGAs) homologous to cloned disease resistance genes (R genes) and the E-value and similarity basis.

Cloned R gene	Cloned R gene (RGA type)	Species	Gene/Homologs	Similarity (%)	Alignment length (amino acid)	Query start (bp)	Query end (bp)	E-value	RGA type	RGA subclass in comparison to the cloned gene
At_RLP1	LRR-RLP	Aal	Aa_G543910.h1.t1	72.297	148	880	1027	1.18E-52	LRR-RLP	same
At_RPS4	TNL	Aal	Aa_G122930.h1.t1	60.479	167	530	696	1.15E-55	Other-NLR	different
At_RLM1b	TNL	Aal	Aa_G65760.h1.t1	63.758	149	10	158	5.95E-56	TNL	same
At_WRR4a	TNL	Aal	Aa_G65760.h1.t1	66.883	154	6	159	2.39E-59	TNL	same
At_RLM1a	TNL	Aal	Aa_G65760.h1.t1	65.789	152	7	158	2.89E-60	TNL	same
Bju_WRR1	CNL	Aal	Aa_G446450.h1.t1	63.351	191	1	189	1.29E-61	CNL	same
At_WRR4b	TNL	Aal	Aa_G65760.h1.t1	71.812	149	11	159	2.76E-64	TNL	same
At_RPP8	CNL	Aal	Aa_G446450.h1.t1	65.979	194	1	194	8.31E-65	CNL	same
Bra_cRa/cRb	TNL	Aal	Aa_G206860.h1.t1	61.17	188	73	260	3.44E-65	TX	different
At_WRR12	TNL	Aal	Aa_G278430.h1.t1	63.529	170	1	170	1.68E-67	TX	different
At_RFO3	Other-RLK	Aal	Aa_G749180.h1.t1	62.011	179	515	693	5.14E-69	Other-RLK	same
At_RPP1	TNL	Aal	Aa_G47200.h1.t1	61.421	197	84	279	1.82E-70	TX	different
At_WRR9	NL	Aal	Aa_G165270.h1.t1	60.096	208	17	224	6.05E-72	TNL	different
At_WRR4a	TNL	Aal	Aa_G574390.h1.t1	61.818	220	1	218	1.84E-73	Other-NLR	different
Bol_FocBo1	TNL	Aal	Aa_G165270.h1.t1	60.09	223	9	230	2.38E-77	TNL	same
At_RLM1b	TNL	Aal	Aa_G563460.h1.t1	68.617	188	44	231	2.99E-79	TNL	same
At_WRR4b	TNL	Aal	Aa_G574390.h1.t1	63.426	216	7	220	1.54E-79	Other-NLR	different
At_RPP1	TNL	Aal	Aa_G206860.h1.t1	60.829	217	68	284	4.78E-80	TX	different
At_RLM1b	TNL	Aal	Aa_G165270.h1.t1	65.7	207	13	219	8.99E-81	TNL	same
At_RLM1a	TNL	Aal	Aa_G574390.h1.t1	62.963	216	6	219	5.05E-81	Other-NLR	different
At_RLM1b	TNL	Aal	Aa_G229750.h1.t1	64.186	215	5	219	3.45E-81	TNL	same
At_WRR4a	TNL	Aal	Aa_G165270.h1.t1	65.7	207	12	218	5.42E-82	TNL	same
At_WRR9	NL	Aal	Aa_G534280.h1.t1	61.638	232	453	683	1.58E-82	TX	different
At_RLM1b	TNL	Aal	Aa_G612790.h1.t1	61.572	229	5	233	7.47E-84	TNL	same
At_WRR4a	TNL	Aal	Aa_G612790.h1.t1	62.385	218	1	218	3.76E-84	TNL	same
At_WRR4a	TNL	Aal	Aa_G676850.h1.t1	62.212	217	11	227	3.64E-84	TX	different
At_RLM1b	TNL	Aal	Aa_G676850.h1.t1	64.516	217	12	228	9.99E-85	TX	different
At_WRR4a	TNL	Aal	Aa_G302440.h1.t1	61.792	212	12	223	5.96E-85	TX	different
At_WRR4a	TNL	Aal	Aa_G201150.h1.t1	63.134	217	3	219	1.61E-85	TX	different
At_RLM1a	TNL	Aal	Aa_G676850.h1.t1	63.38	213	12	224	6.88E-86	TX	different
At_RLM1a	TNL	Aal	Aa_G534280.h1.t1	60.262	229	449	676	5.86E-86	TX	different
At_WRR4b	TNL	Aal	Aa_G229750.h1.t1	64.651	215	6	220	5.16E-86	TNL	same
At_WRR4a	TNL	Aal	Aa_G225910.h1.t1	63.81	210	9	218	4.38E-86	TX	different
At_WRR4b	TNL	Aal	Aa_G165270.h1.t1	67.15	207	14	220	1.93E-86	TNL	same
At_RLM1a	TNL	Aal	Aa_G229750.h1.t1	65.116	215	5	219	5.07E-88	TNL	same
At_RLM1b	TNL	Aal	Aa_G534280.h1.t1	62.232	233	449	681	4.52E-88	TX	different
At_WRR4a	TNL	Aal	Aa_G276580.h1.t1	63.303	218	3	220	3.26E-88	TX	different
Bra_cRa/cRb	TNL	Aal	Aa_G563350.h1.t1	62.5	232	256	480	9.44E-89	NBS	different
At_RFO3	Other-RLK	Aal	Aa_G241950.h1.t1	82.659	173	16	178	1.97E-89	Other-RLK	same
At_WRR9	NL	Aal	Aa_G408900.h1.t1	63.229	223	5	224	1.38E-89	TX	different
At_RLM1a	TNL	Aal	Aa_G165270.h1.t1	69.565	207	13	219	1.30E-89	TNL	same
At_RPP1	TNL	Aal	Aa_G276580.h1.t1	62.857	210	97	306	6.62E-90	TX	different
At_RLM1b	TNL	Aal	Aa_G276580.h1.t1	66.063	221	1	221	1.31E-90	TX	different
At_WRR9	NL	Aal	Aa_G534280.h1.t1	62.946	224	15	238	9.15E-91	TX	different
At_WRR4b	TNL	Aal	Aa_G676850.h1.t1	66.82	217	13	229	6.73E-91	TX	different
Bra_Crr1a	TNL	Aal	Aa_G339760.h1.t1	67.453	212	65	276	5.66E-91	TX	different
At_RLM1b	TNL	Aal	Aa_G106970.h1.t1	61.638	232	10	230	5.30E-91	TX	different
At_WRR4b	TNL	Aal	Aa_G612790.h1.t1	65.455	220	1	220	3.12E-91	TNL	same
Bol_FocBo1	TNL	Aal	Aa_G792870.h1.t1	71.282	195	7	200	6.87E-92	TX	different
At_WRR4b	TNL	Aal	Aa_G201150.h1.t1	65.611	221	1	221	3.47E-92	TX	different
At_RLM1a	TNL	Aal	Aa_G616140.h1.t1	62.348	247	428	673	2.05E-92	TX	different
Bra_Crr1a	TNL	Aal	Aa_G563350.h1.t1	64.502	231	251	474	1.16E-92	NBS	different
At_RLM1a	TNL	Aal	Aa_G612790.h1.t1	66.667	219	1	219	9.58E-93	TNL	same
At_WRR9	NL	Aal	Aa_G616140.h1.t1	65.086	232	48	278	5.68E-93	TX	different
At_RPP2a	TNL	Aal	Aa_G415020.h1.t1	62.348	247	687	933	4.72E-93	NL	different
At_WRR9	NL	Aal	Aa_G106970.h1.t1	63.507	211	13	223	4.02E-93	TX	different
At_WRR4a	TNL	Aal	Aa_G534270.h1.t1	71.635	208	12	219	1.11E-93	TNL	same
At_RLM1b	TNL	Aal	Aa_G534270.h1.t1	72.596	208	13	220	3.51E-94	TNL	same
At_RLM1a	TNL	Aal	Aa_G201150.h1.t1	66.82	217	4	220	2.45E-94	TX	different
At_WRR9	NL	Aal	Aa_G65730.h1.t1	64.865	222	5	226	1.39E-94	TX	different
At_WRR4a	TNL	Aal	Aa_G106970.h1.t1	62.553	235	8	231	1.06E-94	TX	different
At_RLM1a	TNL	Aal	Aa_G302440.h1.t1	64.253	221	13	233	5.28E-95	TX	different
At_WRR4b	TNL	Aal	Aa_G276580.h1.t1	65.766	222	1	222	3.98E-95	TX	different
At_RLM1b	TNL	Aal	Aa_G616140.h1.t1	60.741	270	419	678	3.80E-95	TX	different
At_RPP1	TNL	Aal	Aa_G339760.h1.t1	67.925	212	93	304	2.85E-95	TX	different
At_RLM1b	TNL	Aal	Aa_G65730.h1.t1	68.018	222	1	221	6.76E-96	TX	different
At_WRR4a	TNL	Aal	Aa_G408900.h1.t1	67.568	222	1	218	6.69E-97	TX	different
At_RLM1a	TNL	Aal	Aa_G276580.h1.t1	67.873	221	1	221	4.09E-98	TX	different
At_RLM1b	TNL	Aal	Aa_G408900.h1.t1	69.507	223	1	219	8.53E-100	TX	different
At_WRR4a	TNL	Aal	Aa_G616140.h1.t1	67.965	231	43	273	7.05E-101	TX	different
At_WRR4a	TNL	Aal	Aa_G65730.h1.t1	71.622	222	1	220	2.23E-101	TX	different
At_RPP2a	TNL	Aal	Aa_G299630.h1.t1	61.433	293	1007	1299	1.33E-102	TX	different
At_RLM1a	TNL	Aal	Aa_G534270.h1.t1	75.962	208	13	220	7.30E-103	TNL	same
At_RPP2b	TNL	Aal	Aa_G503780.h1.t1	66.154	260	222	479	1.99E-103	NBS	different
At_RLM1a	TNL	Aal	Aa_G106970.h1.t1	67.965	231	10	229	7.26E-106	TX	different
At_RRS1	TNL	Aal	Aa_G488000.h1.t1	67.816	261	435	683	1.50E-106	TNL	same
At_WRR4b	TNL	Aal	Aa_G534270.h1.t1	78.365	208	14	221	3.63E-107	TNL	same
Bna_Rlm9/4/7	Other-RLK	Aal	Aa_G412470.h1.t1	60.678	295	506	794	6.20E-108	Other-RLK	same
At_WRR4b	TNL	Aal	Aa_G106970.h1.t1	69.828	232	11	231	8.29E-109	TX	different
At_RAC1	TNL	Aal	Aa_G710830.h1.t1	73.568	227	8	231	5.59E-109	TX	different

At_WRR4a	TNL	Aal	Aa_G534280.h1.t1	72.444	225	9	233	3.02E-109	TX	different
At_RRS1	TNL	Aal	Aa_G636220.h1.t1	69.173	266	425	678	2.72E-109	TNL	same
At_WRR8	TNL	Aal	Aa_G710830.h1.t1	73.128	227	19	241	1.41E-109	TX	different
At_WRR9	NL	Aal	Aa_G365200.h1.t1	62.153	288	301	587	1.23E-109	TN	different
At_RLM1b	TNL	Aal	Aa_G534280.h1.t1	73.661	224	10	233	7.80E-110	TX	different
At_RLM1a	TNL	Aal	Aa_G65730.h1.t1	75.225	222	1	221	6.49E-110	TX	different
At_WRR4b	TNL	Aal	Aa_G408900.h1.t1	74.107	224	1	220	4.26E-111	TX	different
At_RLM1a	TNL	Aal	Aa_G616140.h1.t1	72.727	231	44	274	2.32E-112	TX	different
At_WRR4b	TNL	Aal	Aa_G616140.h1.t1	73.799	229	45	273	1.17E-112	TX	different
At_RLM1b	TNL	Aal	Aa_G616140.h1.t1	75.325	231	44	274	1.95E-113	TX	different
At_WRR4b	TNL	Aal	Aa_G65730.h1.t1	78.281	221	3	222	8.31E-114	TX	different
At_RFO1	Other-RLK	Aal	Aa_G293710.h1.t1	63.636	286	277	561	1.81E-114	Other-RLK	same
At_RRS1	TNL	Aal	Aa_G636220.h1.t1	62.429	354	1	352	1.30E-115	TNL	same
At_RPP2a	TNL	Aal	Aa_G369060.h1.t1	63.158	304	115	414	9.48E-117	TNL	same
Bol_FocBo1	TNL	Aal	Aa_G24630.h1.t1	65.942	276	97	372	1.84E-117	TX	different
At_RLM1a	TNL	Aal	Aa_G534280.h1.t1	78.222	225	10	234	5.65E-121	TX	different
At_WRR4b	TNL	Aal	Aa_G534280.h1.t1	77.778	234	1	234	1.93E-121	TX	different
At_RRS1	TNL	Aal	Aa_G488000.h1.t1	60.623	353	1	352	1.47E-121	TNL	same
At_RPP5	TNL	Aal	Aa_G902620.h1.t1	61.243	338	1	330	2.30E-122	TN	different
At_RPP2a	TNL	Aal	Aa_G565780.h1.t1	61.585	328	93	414	1.59E-123	TNL	same
At_RFO1	Other-RLK	Aal	Aa_G265680.h1.t1	64.026	303	423	719	8.86E-127	Other-RLK	same
At_RLP30	LRR-RLP	Aal	Aa_G319570.h1.t1	62.342	316	470	777	3.10E-127	LRR-RLP	same
At_RPP4	TNL	Aal	Aa_G902620.h1.t1	61.791	335	1	328	5.52E-129	TN	different
At_RPP13	CNL	Aal	Aa_G634840.h1.t1	74.632	272	13	281	1.61E-129	CNL	same
At_RLM3	TN	Aal	Aa_G902620.h1.t1	60.55	327	3	322	6.44E-130	TN	same
At_WRR4b	TNL	Aal	Aa_G589470.h1.t1	64.465	318	95	410	1.14E-130	TN	different
Bol_FocBo1	TNL	Aal	Aa_G902620.h1.t1	63.889	324	7	324	5.65E-139	TN	different
At_RPP2a	TNL	Aal	Aa_G295940.h1.t1	60.396	404	11	414	1.17E-141	TNL	same
At_RPP1	TNL	Aal	Aa_G360810.h1.t1	60.582	378	59	431	4.27E-142	TNL	same
Bna_Rlm9/4/7	Other-RLK	Aal	Aa_G265680.h1.t1	69.325	326	454	779	5.86E-144	Other-RLK	same
At_RLM1a	TNL	Aal	Aa_G589470.h1.t1	67.712	319	93	410	1.20E-144	TN	different
At_RFO1	Other-RLK	Aal	Aa_G556850.h1.t1	65.964	332	426	751	3.23E-147	Other-RLK	same
Bna_Rlm9/4/7	Other-RLK	Aal	Aa_G293710.h1.t1	82.721	272	324	594	4.34E-148	Other-RLK	same
Bra_cRa/cRb	TNL	Aal	Aa_G146330.h1.t1	60.207	387	73	458	2.67E-148	TNL	same
At_RPP2a	TNL	Aal	Aa_G415020.h1.t1	79.522	293	1007	1299	5.92E-155	NL	different
Bra_cRa/cRb	TNL	Aal	Aa_G360810.h1.t1	62.47	413	1	411	4.30E-156	TNL	same
Bra_cRa/cRb	TNL	Aal	Aa_G84490.h1.t1	66.762	349	97	445	2.13E-157	TNL	same
At_RLP30	LRR-RLP	Aal	Aa_G245790.h1.t1	61.055	398	386	782	6.23E-159	LRR-RLP	same
At_RPP2a	TNL	Aal	Aa_G173310.h1.t1	61.098	419	1	414	6.58E-162	TNL	same
At_RPP2a	TNL	Aal	Aa_G628640.h1.t1	60.097	411	10	414	5.39E-162	TNL	same
At_RPP2a	TNL	Aal	Aa_G628630.h1.t1	63.119	404	11	414	4.65E-162	TNL	same
At_RPP1	TNL	Aal	Aa_G84490.h1.t1	67.435	347	121	467	3.72E-162	TNL	same
Bra_Crr1a	TNL	Aal	Aa_G447910.h1.t1	60.671	417	52	464	6.55E-163	TNL	same
At_RPP2a	TNL	Aal	Aa_G486460.h1.t1	60.75	400	2	396	1.99E-163	TN	different
Bra_Crr1a	TNL	Aal	Aa_G84490.h1.t1	69.54	348	92	439	1.86E-164	TNL	same
At_WRR4a	TNL	Aal	Aa_G65800.h1.t1	60.465	430	1	427	1.67E-165	TN	different
Bra_cRa/cRb	TNL	Aal	Aa_G332260.h1.t1	60.945	402	73	473	6.25E-166	TN	different
At_ADR1	NL	Aal	Aa_G811740.h1.t1	65.449	356	216	560	1.04E-167	NL	same
Bra_Crr1a	TNL	Aal	Aa_G146330.h1.t1	64.691	388	68	455	5.30E-169	TNL	same
At_WRR9	NL	Aal	Aa_G65800.h1.t1	61.893	412	5	414	1.41E-169	TN	different
At_RLM3	TN	Aal	Aa_G486460.h1.t1	60.05	403	1	394	3.17E-170	TN	same
At_RPP2a	TNL	Aal	Aa_G620000.h1.t1	63.133	415	8	414	1.32E-170	TNL	same
At_RPP1	TNL	Aal	Aa_G447910.h1.t1	61.449	428	69	492	2.01E-174	TNL	same
Bra_Crr1a	TNL	Aal	Aa_G360810.h1.t1	74.854	342	66	406	8.34E-175	TNL	same
At_RPP2a	TNL	Aal	Aa_G295950.h1.t1	66.019	412	3	414	2.49E-175	TNL	same
At_RLM1b	TNL	Aal	Aa_G65800.h1.t1	64.891	413	1	410	6.79E-177	TN	different
At_WRR4b	TNL	Aal	Aa_G65800.h1.t1	60.377	477	4	474	1.44E-177	TN	different
Bra_Crr1a	TNL	Aal	Aa_G332260.h1.t1	64.764	403	67	468	5.57E-178	TN	different
At_RPP5	TNL	Aal	Aa_G295910.h1.t1	63.193	451	1	443	2.48E-178	TNL	same
At_BAK1	LRR-RLK	Aal	Aa_G609980.h1.t1	94.797	615	1	615	0	LRR-RLK	same
At_RFO3	Other-RLK	Aal	Aa_G241950.h1.t1	85.169	472	389	850	0	Other-RLK	same
At_RPS2	NL	Aal	Aa_G43080.h1.t1	85	740	170	909	0	CNL	different
At_RLP1	LRR-RLP	Aal	Aa_G275730.h1.t1	83.221	888	190	1076	0	LRR-RLP	same
At_RFO1	Other-RLK	Aal	Aa_G265710.h1.t1	83.109	521	236	751	0	Other-RLK	same
At_SOBIR1	LRR-RLK	Aal	Aa_G154740.h1.t1	82.492	634	8	641	0	LRR-RLK	same
At_FLS2	LRR-RLK	Aal	Aa_G524900.h1.t1	80.703	1109	21	1125	0	LRR-RLK	same
At_NGR1a	RNL	Aal	Aa_G180800.h1.t1	80.025	816	1	809	0	NL	different
Bna_Rlm9/4/7	Other-RLK	Aal	Aa_G21490.h1.t1	79.802	505	297	794	0	Other-RLK	same
At_BAK1	LRR-RLK	Aal	Aa_G377020.h1.t1	78.352	619	9	615	0	LRR-RLK	same
At_BAK1	LRR-RLK	Aal	Aa_G245800.h1.t1	77.724	624	4	615	0	LRR-RLK	same
At_RPP2a	TNL	Aal	Aa_G299570.h1.t1	76.027	876	425	1299	0	TNL	same
At_RPM1	NL	Aal	Aa_G78900.h1.t1	75.512	927	1	926	0	NL	same
At_RPP2b	TNL	Aal	Aa_G503790.h1.t1	74.814	1207	3	1207	0	TNL	same
At_RPP8	CNL	Aal	Aa_G446440.h1.t1	70.975	913	1	907	0	NL	different
At_RPP2a	TNL	Aal	Aa_G299590.h1.t1	70.126	713	579	1288	0	TNL	same
At_RFO2	LRR-RLP	Aal	Aa_G347880.h1.t1	69.12	693	45	737	0	LRR-RLK	different
At_RPP1	TNL	Aal	Aa_G146330.h1.t1	68.912	386	96	481	0	TNL	same
Bna_Rlm9/4/7	Other-RLK	Aal	Aa_G30130.h1.t1	68.908	476	282	727	0	Other-RLK	same
Bol_FocBo1	TNL	Aal	Aa_G486460.h1.t1	68.702	393	9	396	0	TN	different
Bna_Rlm9/4/7	Other-RLK	Aal	Aa_G265690.h1.t1	67.213	488	113	595	0	Other-RLK	same
At_NGR1b	RNL	Aal	Aa_G180800.h1.t1	67.193	823	3	815	0	NL	different
At_RLM1a	TNL	Aal	Aa_G65800.h1.t1	67.073	410	3	410	0	TN	different
At_RLP23	LRR-RLP	Aal	Aa_G183780.h1.t1	66.996	709	183	890	0	LRR-RLP	same
Bna_Rlm9/4/7	Other-RLK	Aal	Aa_G323670.h1.t1	66.667	435	361	793	0	Other-RLK	same
At_RLP42	LRR-RLP	Aal	Aa_G183780.h1.t1	66.619	701	183	881	0	LRR-RLP	same
At_RPP13	CNL	Aal	Aa_G500650.h1.t1	66.435	864	1	830	0	CNL	same

Bol_FocBo1	TNL	Aal	Aa_G628630.h1.t1	66.202	645	10	653	0	TNL	same
At_RLP23	LRR-RLP	Aal	Aa_G208770.h1.t1	66.096	643	248	890	0	LRR-RLP	same
At_NGR1a	RNL	Aal	Aa_G180780.h1.t1	66.091	637	177	809	0	NL	different
At_RPS4	TNL	Aal	Aa_G488020.h1.t1	66.034	1160	13	1163	0	Other-NLR	different
At_RLP42	LRR-RLP	Aal	Aa_G208770.h1.t1	65.984	635	249	879	0	LRR-RLP	same
Bra_Crr1a	TNL	Aal	Aa_G578400.h1.t1	65.772	596	119	692	0	TNL	same
At_RPS5	TNL	Aal	Aa_G521440.h1.t1	65.692	889	1	883	0	CNL	different
At_RLP42	LRR-RLP	Aal	Aa_G201920.h1.t1	65.394	864	22	879	0	LRR-RLP	same
At_RLP23	LRR-RLP	Aal	Aa_G201920.h1.t1	65.233	860	33	890	0	LRR-RLP	same
Bna_Rlm9/4/7	Other-RLK	Aal	Aa_G557360.h1.t1	65.228	417	324	737	0	Other-RLK	same
Bol_FocBo1	TNL	Aal	Aa_G295940.h1.t1	65.227	1366	10	1349	0	TNL	same
At_RLP23	LRR-RLP	Aal	Aa_G183750.h1.t1	65.12	711	182	890	0	LRR-RLP	same
Bna_Rlm9/4/7	Other-RLK	Aal	Aa_G293690.h1.t1	65.108	556	250	793	0	Other-RLK	same
At_RFO1	Other-RLK	Aal	Aa_G30130.h1.t1	65.057	435	290	696	0	Other-RLK	same
At_RLP42	LRR-RLP	Aal	Aa_G183750.h1.t1	64.95	699	182	878	0	LRR-RLP	same
At_RPS5	TNL	Aal	Aa_G366140.h1.t1	64.926	881	1	880	0	CNL	different
At_ADR1	NL	Aal	Aa_G636220.h1.t1	64.89	544	245	787	0	TNL	different
At_RPS5	TNL	Aal	Aa_G124650.h1.t1	64.713	887	1	880	0	CNL	different
At_RPP1	TNL	Aal	Aa_G332260.h1.t1	64.516	403	95	496	0	TN	different
At_RLP23	LRR-RLP	Aal	Aa_G363510.h1.t1	64.106	716	175	888	0	LRR-RLP	same
At_RPP7	NL	Aal	Aa_G323330.h1.t1	64.097	869	3	866	0	NL	same
At_RLP42	LRR-RLP	Aal	Aa_G363510.h1.t1	63.781	693	182	873	0	LRR-RLP	same
Bol_FocBo1	TNL	Aal	Aa_G295950.h1.t1	63.695	1088	7	1094	0	TNL	same
At_RPP8	CNL	Aal	Aa_G446450.h1.t1	63.537	639	273	904	0	CNL	same
At_RPS5	TNL	Aal	Aa_G193720.h1.t1	63.39	885	1	883	0	CNL	different
At_RLP23	LRR-RLP	Aal	Aa_G183740.h1.t1	63.26	724	172	890	0	LRR-RLP	same
At_RFO1	Other-RLK	Aal	Aa_G323670.h1.t1	63.134	434	326	751	0	Other-RLK	same
At_NGR1a	RNL	Aal	Aa_G180770.h1.t1	63.086	810	10	804	0	NL	different
At_RPP2a	TNL	Aal	Aa_G299610.h1.t1	63.082	558	748	1299	0	NL	different
At_NGR1b	RNL	Aal	Aa_G180770.h1.t1	62.885	811	12	813	0	NL	different
At_WRR8	TNL	Aal	Aa_G208030.h1.t1	62.801	707	5	704	0	TNL	same
At_RLM1b	TNL	Aal	Aa_G398600.h1.t1	62.718	1030	10	1013	0	TNL	same
At_RLP30	LRR-RLP	Aal	Aa_G21020.h1.t1	62.636	736	46	774	0	LRR-RLP	same
Bra_cRa/cRb	TNL	Aal	Aa_G578400.h1.t1	61.864	590	124	695	0	TNL	same
At_RLP42	LRR-RLP	Aal	Aa_G183740.h1.t1	61.833	731	172	889	0	LRR-RLP	same
At_RAC1	TNL	Aal	Aa_G208030.h1.t1	61.744	711	3	703	0	TNL	same
At_RLM1b	TNL	Aal	Aa_G65950.h1.t1	61.715	1003	1	995	0	TNL	same
Bju_WRR1	CNL	Aal	Aa_G446440.h1.t1	61.63	920	1	900	0	NL	different
Bol_FocBo1	TNL	Aal	Aa_G173310.h1.t1	61.57	968	5	960	0	TNL	same
At_NGR1b	RNL	Aal	Aa_G180780.h1.t1	61.502	639	181	815	0	NL	different
At_RLP32	LRR-RLP	Aal	Aa_G333250.h1.t1	61.481	810	121	854	0	LRR-RLP	same
At_RFO1	Other-RLK	Aal	Aa_G21490.h1.t1	61.46	493	270	750	0	Other-RLK	same
At_RLM1a	TNL	Aal	Aa_G65950.h1.t1	61.419	902	1	897	0	TNL	same
At_RLP32	LRR-RLP	Aal	Aa_G216090.h1.t1	61.346	921	1	853	0	LRR-RLP	same
At_WRR8	TNL	Aal	Aa_G208060.h1.t1	61.011	554	31	576	0	TN	different
Bol_FocBo1	TNL	Aal	Aa_G620000.h1.t1	60.95	968	7	960	0	TNL	same
At_RPP1	TNL	Aal	Aa_G146320.h1.t1	60.943	594	95	687	0	TN	different
At_RFO1	Other-RLK	Aal	Aa_G557360.h1.t1	60.89	427	287	712	0	Other-RLK	same
At_RRS1	TNL	Aal	Aa_G519310.h1.t1	60.881	1181	1	1168	0	TNL	same
Bra_Crr1a	TNL	Aal	Aa_G753220.h1.t1	60.569	738	92	822	0	TNL	same
At_RFO1	Other-RLK	Aal	Aa_G148280.h1.t1	60.544	735	7	721	0	Other-RLK	same
At_RLP30	LRR-RLP	Aal	Aa_G245780.h1.t1	60.514	623	166	786	0	LRR-RLP	same
At_RPP5	TNL	Aal	Aa_G628630.h1.t1	60.443	632	12	632	0	TNL	same
At_RAC1	TNL	Aal	Aa_G208060.h1.t1	60.436	551	20	562	0	TN	different
At_RLM1a	TNL	Aal	Aa_G65780.h1.t1	60.349	918	3	898	0	TNL	same
Bra_Crr1a	TNL	Aal	Aa_G146320.h1.t1	60.345	638	29	661	0	TN	different
At_RAC1	TNL	Aal	Aa_G33030.h1.t1	60.244	737	9	736	0	TNL	same
At_ADR1	NL	Aal	Aa_G719500.h1.t1	60.241	581	6	560	0	NBS	different
At_RPS5	TNL	Aal	Aa_G193750.h1.t1	60.136	883	1	878	0	CNL	different
At_WRR8	TNL	Aal	Aa_G33030.h1.t1	60.081	744	20	755	0	TNL	same
At_RPP4	TNL	Chi	CARHR222470	61.667	180	687	858	2.02E-57	TNL	same
At_RPP7	NL	Chi	CARHR123290	67.066	167	573	739	1.10E-57	LRR-RLP	different
At_RFO3	Other-RLK	Chi	CARHR051100	63.855	166	512	677	6.42E-64	Other-RLK	same
At_RLP32	LRR-RLP	Chi	CARHR105480	60.104	193	663	853	2.11E-64	LRR-RLP	same
Bna_LepR3/Rlm2	LRR-RLP	Chi	CARHR105480	60.488	205	746	950	1.08E-68	LRR-RLP	same
At_RPS5	TNL	Chi	CARHR048930	62.573	171	168	338	7.77E-71	NBS	different
At_NGR1a	RNL	Chi	CARHR177820	65.5	200	153	352	4.84E-74	NBS	different
At_RLP30	LRR-RLP	Chi	CARHR291370	62.562	203	525	722	2.38E-75	LRR-RLP	same
At_RLP42	LRR-RLP	Chi	CARHR126950	64.078	206	686	886	8.58E-76	LRR-RLP	same
At_RFO3	Other-RLK	Chi	CARHR230490	63.483	178	515	692	1.57E-77	Other-RLK	same
At_RFO3	Other-RLK	Chi	CARHR230490	63.483	178	515	692	7.66E-78	Other-RLK	same
At_RPP7	NL	Chi	CARHR284010	70.95	179	415	591	4.38E-80	NL	same
At_RPS5	TNL	Chi	CARHR049610	61.353	207	302	508	6.34E-81	NBS	different
At_RLP23	LRR-RLP	Chi	CARHR126950	68.137	204	686	889	1.30E-81	LRR-RLP	same
At_RPP7	NL	Chi	CARHR284080	71.892	185	126	309	1.04E-82	NBS	different
At_RLP30	LRR-RLP	Chi	CARHR118040	67.241	232	549	780	5.27E-89	LRR-RLP	same
At_ADR1	NL	Chi	CARHR033020	78.313	166	323	488	7.95E-90	NBS	different
At_RFO1	Other-RLK	Chi	CARHR062770	65.686	204	350	552	8.34E-92	Other-RLK	same
At_NGR1b	RNL	Chi	CARHR292120	67.873	221	337	555	6.77E-96	CN	different
At_NGR1b	RNL	Chi	CARHR292120	67.873	221	337	555	2.55E-96	CN	different
At_NGR1a	RNL	Chi	CARHR292120	71.364	220	334	549	7.77E-101	CN	different
At_NGR1a	RNL	Chi	CARHR292120	71.233	219	335	549	6.51E-101	CN	different
At_RPP39	CNL	Chi	CARHR052120	63.469	271	24	293	4.28E-111	CN	different
At_RRS1	TNL	Chi	CARHR176990	67.279	272	902	1170	3.26E-111	TX	different
At_RPS5	TNL	Chi	CARHR049180	62.411	282	1	282	1.36E-114	CN	different
At_RAC1	TNL	Chi	CARHR174840	68.44	282	103	383	2.34E-115	TNL	same

At_RPP39	CNL	Chi	CARHR052120	61.905	294	1	293	1.06E-117	CN	different
Bna_Rlm9/4/7	Other-RLK	Chi	CARHR062770	85.366	205	381	585	2.22E-119	Other-RLK	same
At_RAC1	TNL	Chi	CARHR289490	66.441	295	4	294	3.26E-120	TX	different
At_RPP7	NL	Chi	CARHR284050	68.359	256	353	606	1.33E-120	NL	same
At_RPP2a	TNL	Chi	CARHR223500	65.931	317	98	414	1.67E-124	Other-NLR	different
At_RAC1	TNL	Chi	CARHR175240	64.725	309	218	523	3.09E-125	NBS	different
At_WRR8	TNL	Chi	CARHR289490	67.007	294	15	304	2.75E-126	TX	different
At_NGR1b	RNL	Chi	CARHR177840	65.517	319	135	450	5.03E-129	NBS	different
At_BAK1	LRR-RLK	Chi	CARHR176190	61.875	320	255	573	9.00E-134	LRR-RLK	same
At_RLM3	TN	Chi	CARHR222990	60.92	348	3	350	2.50E-135	TN	same
Bra_Crr1a	TNL	Chi	CARHR071840	65.046	329	68	395	1.08E-139	TN	different
At_NGR1a	RNL	Chi	CARHR177840	73.63	292	154	444	2.31E-140	NL	different
Bna_Rlm9/4/7	Other-RLK	Chi	CARHR016630	75	268	396	662	9.59E-141	Other-RLK	same
Bna_Rlm9/4/7	Other-RLK	Chi	CARHR016630	75	268	396	662	9.59E-141	Other-RLK	same
At_RPP4	TNL	Chi	CARHR223500	63.868	393	100	488	2.23E-144	TX	different
At_RLP42	LRR-RLP	Chi	CARHR127040	61.298	416	20	434	1.10E-146	LRR-RLP	same
At_RFO1	Other-RLK	Chi	CARHR016630	81.452	248	383	629	2.02E-148	Other-RLK	same
At_RFO1	Other-RLK	Chi	CARHR016630	81.452	248	383	629	2.02E-148	Other-RLK	same
At_RRS1	TNL	Chi	CARHR177150	67.705	353	8	357	2.38E-150	TN	different
At_WRR8	TNL	Chi	CARHR175240	72.581	310	228	535	1.53E-150	NBS	different
At_RLM1b	TNL	Chi	CARHR213880	64.266	361	52	411	3.97E-152	NBS	different
At_RAC1	TNL	Chi	CARHR174840	66.489	376	9	383	2.50E-153	TNL	same
At_RLP23	LRR-RLP	Chi	CARHR127040	60.952	420	17	434	4.76E-155	LRR-RLP	same
At_RPS5	TNL	Chi	CARHR220850	64.77	369	8	376	1.58E-155	CNL	different
At_RRS1	TNL	Chi	CARHR177010	73.214	336	9	338	9.53E-157	NBS	different
At_RLP1	LRR-RLP	Chi	CARHR185740	65.897	390	667	1053	2.88E-159	LRR-RLP	same
Bna_LepR3/Rlm2	LRR-RLP	Chi	CARHR105510	71.023	352	593	944	3.02E-164	LRR-RLP	same
At_NGR1b	RNL	Chi	CARHR289020	61.501	413	407	815	8.79E-165	LRR-RLP	different
At_RLP32	LRR-RLP	Chi	CARHR105510	69.553	358	510	867	3.38E-166	LRR-RLP	same
At_RPP13	CNL	Chi	CARHR154990	68.194	371	103	471	1.91E-166	NBS	different
At_WRR4b	TNL	Chi	CARHR047710	61.412	425	5	426	7.26E-168	TN	different
At_WRR9	NL	Chi	CARHR149280	62.899	407	271	673	9.40E-170	NL	same
At_RPP39	CNL	Chi	CARHR052140	64.815	432	447	870	1.28E-170	CNL	same
Bra_Crr1a	TNL	Chi	CARHR078370	61.663	433	40	468	3.30E-171	TN	different
At_RLM1b	TNL	Chi	CARHR047710	62.5	424	5	426	1.40E-175	TN	different
At_NGR1a	RNL	Chi	CARHR289020	64.634	410	405	809	1.31E-175	LRR-RLP	different
At_RLM1a	TNL	Chi	CARHR048030	61.572	458	152	595	6.86E-180	NBS	different
At_BAK1	LRR-RLK	Chi	CARHR241480	98.511	470	146	615	0	LRR-RLK	same
At_WRR12	TNL	Chi	CARHR018200	87.226	1049	1	1049	0	TNL	same
At_RLP1	LRR-RLP	Chi	CARHR007110	86.921	734	344	1077	0	LRR-RLP	same
At_SOBIR1	LRR-RLK	Chi	CARHR125590	85.959	641	1	641	0	LRR-RLK	same
At_SOBIR1	LRR-RLK	Chi	CARHR125590	85.959	641	1	641	0	LRR-RLK	same
At_RFO1	Other-RLK	Chi	CARHR073440	85.563	755	1	751	0	Other-RLK	same
At_RPS4	TNL	Chi	CARHR177110	84.907	1186	14	1199	0	TNL	same
At_RPS4	TNL	Chi	CARHR177110	84.907	1186	14	1199	0	TNL	same
At_RPS4	TNL	Chi	CARHR177110	84.907	1186	14	1199	0	TNL	same
At_RPS5	TNL	Chi	CARHR012520	83.875	893	1	887	0	CNL	different
At_RPS2	NL	Chi	CARHR233600	83.736	910	1	909	0	NL	same
At_BAK1	LRR-RLK	Chi	CARHR104720	83.191	470	146	615	0	LRR-RLK	same
At_BAK1	LRR-RLK	Chi	CARHR290920	80.952	483	146	615	0	LRR-RLK	same
At_FLS2	LRR-RLK	Chi	CARHR175480	80.459	1177	1	1173	0	LRR-RLK	same
At_RLP32	LRR-RLP	Chi	CARHR080100	80.374	856	1	853	0	LRR-RLP	same
At_BAK1	LRR-RLK	Chi	CARHR290920	79.669	605	24	615	0	LRR-RLK	same
At_RLP32	LRR-RLP	Chi	CARHR080100	79.517	869	1	866	0	LRR-RLP	same
Bna_Rlm9/4/7	Other-RLK	Chi	CARHR016600	76.613	372	378	748	0	Other-RLK	same
Bna_Rlm9/4/7	Other-RLK	Chi	CARHR016600	76.613	372	378	748	0	Other-RLK	same
At_RPP2b	TNL	Chi	CARHR226300	75.376	1198	1	1193	0	TNL	same
At_RPP2b	TNL	Chi	CARHR226300	75.376	1198	1	1193	0	TNL	same
At_RPP2b	TNL	Chi	CARHR226300	75.376	1198	1	1193	0	TNL	same
At_RPP2a	TNL	Chi	CARHR226300	75.356	913	410	1299	0	TNL	same
At_RFO3	Other-RLK	Chi	CARHR091190	75.348	791	73	850	0	Other-RLK	same
At_RFO3	Other-RLK	Chi	CARHR091190	75.285	789	73	848	0	Other-RLK	same
At_RPP1	TNL	Chi	CARHR151520	73.675	547	69	613	0	Other-NLR	different
At_RPP8	CNL	Chi	CARHR183400	73.326	911	1	907	0	CNL	same
At_RPP2b	TNL	Chi	CARHR288050	73.163	980	1	978	0	TNL	same
At_RPS4	TNL	Chi	CARHR177020	72.215	763	1	755	0	Other-NLR	different
At_RPP2a	TNL	Chi	CARHR288060	72.102	785	516	1297	0	TNL	same
At_RPP2b	TNL	Chi	CARHR288050	72.05	1195	1	1193	0	TNL	same
At_RPP2b	TNL	Chi	CARHR288050	72.05	1195	1	1193	0	TNL	same
At_RPP8	CNL	Chi	CARHR010420	72.009	468	1	462	0	CN	different
At_RPP8	CNL	Chi	CARHR010420	72.009	468	1	462	0	CN	different
At_RPP39	CNL	Chi	CARHR052080	72.009	443	1	442	0	CN	different
At_RFO1	Other-RLK	Chi	CARHR016610	71.882	441	282	719	0	Other-RLK	same
At_RLM1b	TNL	Chi	CARHR149280	71.253	407	267	672	0	NL	different
At_RLM1b	TNL	Chi	CARHR047100	70.851	470	1	470	0	TN	different
At_RLP23	LRR-RLP	Chi	CARHR126570	70.729	878	17	888	0	LRR-RLP	same
At_RFO2	LRR-RLP	Chi	CARHR261670	70.278	720	30	748	0	LRR-RLP	same
At_NGR1a	RNL	Chi	CARHR177840	70.213	658	154	806	0	NL	different
At_RLM1b	TNL	Chi	CARHR047300	69.951	1025	1	1009	0	TNL	same
At_RFO1	Other-RLK	Chi	CARHR016600	69.577	378	343	717	0	Other-RLK	same
At_RFO1	Other-RLK	Chi	CARHR016600	69.577	378	343	717	0	Other-RLK	same
At_RLM1b	TNL	Chi	CARHR047090	69.515	1030	1	1013	0	TNL	same
At_BAK1	LRR-RLK	Chi	CARHR033720	68.938	631	23	615	0	LRR-RLK	same
At_RPP2a	TNL	Chi	CARHR222470	68.932	412	3	414	0	TNL	same
At_RPP39	CNL	Chi	CARHR052100	68.898	463	160	616	0	NL	different
At_RLP42	LRR-RLP	Chi	CARHR126570	68.879	874	18	880	0	LRR-RLP	same

At_RPP39	CNL	Chi	CARHR052140	68.734	387	1	386	0	CNL	same
At_RFO2	LRR-RLP	Chi	CARHR017820	68.646	724	20	742	0	LRR-RLP	same
At_RLM1b	TNL	Chi	CARHR047140	68.6	1000	1	989	0	TNL	same
At_RLM1b	TNL	Chi	CARHR047120	68.416	1010	1	996	0	TNL	same
Bna_Rlm9/4/7	Other-RLK	Chi	CARHR062740	68.344	477	125	594	0	Other-RLK	same
At_RPP8	CNL	Chi	CARHR183370	68.287	432	349	775	0	NL	different
At_RPP8	CNL	Chi	CARHR011140	68.202	912	1	908	0	CNL	same
At_RLP1	LRR-RLP	Chi	CARHR007120	68.093	514	564	1071	0	LRR-RLP	same
At_RLP1	LRR-RLP	Chi	CARHR007120	68.093	514	564	1071	0	LRR-RLP	same
At_RLP1	LRR-RLP	Chi	CARHR007120	68.093	514	564	1071	0	LRR-RLP	same
At_RLM1b	TNL	Chi	CARHR047210	67.943	836	164	990	0	NL	different
At_RLP32	LRR-RLP	Chi	CARHR085660	67.791	860	1	853	0	LRR-RLP	same
Bna_Rlm9/4/7	Other-RLK	Chi	CARHR073450	67.522	779	23	794	0	Other-RLK	same
At_RLM1b	TNL	Chi	CARHR047170	67.368	570	210	775	0	NL	different
At_RLP32	LRR-RLP	Chi	CARHR105520	67.339	744	113	854	0	LRR-RLP	same
Bju_WRR1	CNL	Chi	CARHR010420	67.171	463	1	456	0	CN	different
Bju_WRR1	CNL	Chi	CARHR010420	67.171	463	1	456	0	CN	different
At_RLM1a	TNL	Chi	CARHR047100	67.164	469	1	469	0	TN	different
At_RPP39	CNL	Chi	CARHR051470	67.122	879	1	868	0	CNL	same
At_RLP23	LRR-RLP	Chi	CARHR126990	67.082	723	183	890	0	LRR-RLP	same
At_RPP7	NL	Chi	CARHR123300	67.023	561	1	560	0	NBS	different
At_RPP13	CNL	Chi	CARHR154980	67.014	864	1	830	0	CNL	same
At_RPP8	CNL	Chi	CARHR290720	66.923	910	1	904	0	CNL	same
At_RPP8	CNL	Chi	CARHR290720	66.923	910	1	904	0	CNL	same
At_RLM1a	TNL	Chi	CARHR149280	66.914	405	267	667	0	NL	different
At_RFO2	LRR-RLP	Chi	CARHR065190	66.392	729	13	737	0	LRR-RLK	different
At_RFO2	LRR-RLP	Chi	CARHR065190	66.392	729	13	737	0	LRR-RLK	different
At_RFO1	Other-RLK	Chi	CARHR016640	66.234	616	25	626	0	Other-RLK	same
At_RFO1	Other-RLK	Chi	CARHR016640	66.234	616	25	626	0	Other-RLK	same
At_NGR1b	RNL	Chi	CARHR177840	66.174	677	142	812	0	NL	different
At_RPP7	NL	Chi	CARHR283900	66.161	461	416	866	0	NL	same
At_RPP39	CNL	Chi	CARHR051470	66.098	879	1	868	0	CNL	same
At_RPP39	CNL	Chi	CARHR052110	65.984	732	146	867	0	CNL	same
At_RPP39	CNL	Chi	CARHR052110	65.984	732	146	867	0	CNL	same
At_RPP39	CNL	Chi	CARHR052110	65.984	732	146	867	0	CNL	same
At_RPP7	NL	Chi	CARHR283900	65.939	458	419	866	0	NL	same
At_RRS1	TNL	Chi	CARHR177100	65.901	1176	8	1169	0	TNL	same
At_RPS4	TNL	Chi	CARHR177200	65.673	973	158	1126	0	NL	different
At_RFO1	Other-RLK	Chi	CARHR016610	65.522	728	10	719	0	Other-RLK	same
At_RPS5	TNL	Chi	CARHR013070	65.484	733	1	729	0	CNL	different
At_RPS5	TNL	Chi	CARHR013070	65.484	733	1	729	0	CNL	different
At_RLM1b	TNL	Chi	CARHR047080	65.43	1024	1	1015	0	TNL	same
At_RPP7	NL	Chi	CARHR284070	65.399	789	5	786	0	NL	same
At_RFO1	Other-RLK	Chi	CARHR016620	65.385	754	6	750	0	Other-RLK	same
At_RPP7	NL	Chi	CARHR283760	65.32	718	151	866	0	NL	same
At_RPP7	NL	Chi	CARHR283760	65.32	718	151	866	0	NL	same
At_RLP42	LRR-RLP	Chi	CARHR126990	65.223	716	183	880	0	LRR-RLP	same
Bol_FocBo1	TNL	Chi	CARHR222470	65.217	713	1	711	0	TNL	same
Bna_Rlm9/4/7	Other-RLK	Chi	CARHR016610	65.207	434	325	751	0	Other-RLK	same
At_WRR4b	TNL	Chi	CARHR047510	65.116	430	5	432	0	TN	different
At_RFO1	Other-RLK	Chi	CARHR016640	65.079	756	6	747	0	Other-RLK	same
At_RPS5	TNL	Chi	CARHR012590	65.04	881	1	880	0	CNL	different
At_RLM1a	TNL	Chi	CARHR047710	64.941	425	4	426	0	TN	different
At_RLP23	LRR-RLP	Chi	CARHR126970	64.854	717	175	889	0	LRR-RLP	same
Bna_LepR3/Rlm2	LRR-RLP	Chi	CARHR105520	64.845	970	1	948	0	LRR-RLP	same
At_WRR8	TNL	Chi	CARHR175840	64.835	546	354	894	0	NL	different
At_RLP23	LRR-RLP	Chi	CARHR127300	64.723	703	182	877	0	LRR-RLP	same
At_WRR8	TNL	Chi	CARHR174840	64.568	1112	20	1100	0	TNL	same
At_RLM1a	TNL	Chi	CARHR047510	64.419	430	4	432	0	TN	different
At_RPP7	NL	Chi	CARHR283910	64.401	868	4	866	0	CNL	different
At_RPP7	NL	Chi	CARHR283910	64.401	868	4	866	0	CNL	different
At_RAC1	TNL	Chi	CARHR175840	64.234	548	344	888	0	NL	different
Bna_Rlm9/4/7	Other-RLK	Chi	CARHR057010	64.122	786	24	794	0	Other-RLK	same
Bra_Crr1a	TNL	Chi	CARHR294530	64.12	432	66	495	0	TN	different
Bra_Crr1a	TNL	Chi	CARHR294530	64.12	432	66	495	0	TN	different
Bra_Crr1a	TNL	Chi	CARHR294530	64.12	432	66	495	0	TN	different
At_WRR4b	TNL	Chi	CARHR055030	63.957	1107	4	1071	0	TNL	same
At_RAC1	TNL	Chi	CARHR116350	63.953	430	9	434	0	TN	different
At_RAC1	TNL	Chi	CARHR116350	63.953	430	9	434	0	TN	different
At_RLM1a	TNL	Chi	CARHR047430	63.844	614	11	622	0	TNL	same
At_RFO1	Other-RLK	Chi	CARHR016610	63.839	719	10	719	0	Other-RLK	same
At_WRR12	TNL	Chi	CARHR262010	63.681	1027	11	1028	0	TNL	same
At_RLM1b	TNL	Chi	CARHR047510	63.636	429	4	431	0	TN	different
At_RLM1b	TNL	Chi	CARHR047270	63.561	848	164	1003	0	NL	different
At_RRS1	TNL	Chi	CARHR177640	63.546	705	473	1169	0	TX	different
At_RRS1	TNL	Chi	CARHR177640	63.546	705	473	1169	0	TX	different
At_RRS1	TNL	Chi	CARHR177030	63.35	1176	8	1169	0	TNL	same
At_RLM1b	TNL	Chi	CARHR051270	63.243	993	1	981	0	TNL	same
At_RPP7	NL	Chi	CARHR284020	63.185	785	89	869	0	NL	same
At_RPP7	NL	Chi	CARHR284130	63.116	873	4	866	0	CNL	different
At_RPP7	NL	Chi	CARHR284130	63.116	873	4	866	0	CNL	different
At_RPP7	NL	Chi	CARHR284130	63.116	873	4	866	0	CNL	different
At_RLP42	LRR-RLP	Chi	CARHR126970	63.077	715	182	889	0	LRR-RLP	same
At_RPS4	TNL	Chi	CARHR177050	63.046	985	159	1132	0	NL	different
At_RPS4	TNL	Chi	CARHR177620	62.957	1150	8	1139	0	TNL	same
At_RPS4	TNL	Chi	CARHR177620	62.957	1150	8	1139	0	TNL	same

At_WRR4b	TNL	Chi	CARHR047430	62.945	618	12	626	0	TNL	same
At_WRR9	NL	Chi	CARHR047100	62.924	472	4	474	0	TN	different
At_RPP7	NL	Chi	CARHR284130	62.919	836	42	866	0	CNL	different
At_RPP7	NL	Chi	CARHR058170	62.874	870	1	866	0	NL	same
At_RLP42	LRR-RLP	Chi	CARHR127300	62.787	696	182	871	0	LRR-RLP	same
At_RLM1b	TNL	Chi	CARHR050870	62.586	1021	1	999	0	TNL	same
At_RLP1	LRR-RLP	Chi	CARHR006900	62.574	847	223	1067	0	LRR-RLP	same
At_RLM1a	TNL	Chi	CARHR047060	62.537	1009	5	995	0	NBS	different
At_RLM1a	TNL	Chi	CARHR047060	62.537	1009	5	995	0	TX	different
At_RLM1a	TNL	Chi	CARHR050870	62.486	909	1	896	0	TNL	same
At_ADR1	NL	Chi	CARHR208570	62.454	815	6	787	0	NL	same
At_RPP7	NL	Chi	CARHR284060	62.33	884	4	866	0	CNL	different
At_RAC1	TNL	Chi	CARHR174840	62.299	1122	9	1109	0	TNL	same
At_WRR4a	TNL	Chi	CARHR055030	62.268	1023	1	999	0	TNL	same
At_RPP8	CNL	Chi	CARHR028380	62.25	551	363	908	0	CNL	same
At_RPP8	CNL	Chi	CARHR028380	62.25	551	363	908	0	NL	different
At_WRR8	TNL	Chi	CARHR116350	62.15	428	20	442	0	TN	different
At_WRR8	TNL	Chi	CARHR116350	62.15	428	20	442	0	TN	different
At_WRR9	NL	Chi	CARHR047510	62.005	429	9	436	0	TN	different
Bra_Crr1a	TNL	Chi	CARHR151520	61.95	523	68	589	0	Other-NLR	different
At_RPP5	TNL	Chi	CARHR222470	61.898	727	4	715	0	TNL	same
Bna_LepR3/Rlm2	LRR-RLP	Chi	CARHR085660	61.835	752	201	950	0	LRR-RLP	same
At_RPP8	CNL	Chi	CARHR028380	61.774	913	1	908	0	NL	different
At_RPP8	CNL	Chi	CARHR028380	61.594	828	86	908	0	NL	different
At_WRR9	NL	Chi	CARHR047140	61.361	999	4	995	0	TNL	different
At_RLM1b	TNL	Chi	CARHR048030	61.039	462	152	600	0	NBS	different
At_RPS5	TNL	Chi	CARHR012600	60.963	748	139	883	0	CNL	different
At_RPS5	TNL	Chi	CARHR012600	60.963	748	139	883	0	NL	different
At_RLM1b	TNL	Chi	CARHR047430	60.778	617	12	626	0	TNL	same
At_RLP30	LRR-RLP	Chi	CARHR118040	60.633	790	1	781	0	LRR-RLP	same
At_RLP30	LRR-RLP	Chi	CARHR118040	60.633	790	1	781	0	LRR-RLP	same
At_WRR8	TNL	Chi	CARHR116330	60.598	736	20	745	0	TNL	same
At_WRR8	TNL	Chi	CARHR175060	60.488	901	12	891	0	TNL	same
At_RLM1a	TNL	Chi	CARHR047170	60.461	564	210	772	0	NL	different
At_RLM1b	TNL	Chi	CARHR047060	60.405	1038	5	1010	0	TX	different
At_RLM1b	TNL	Chi	CARHR047060	60.405	1038	5	1010	0	TX	different
At_RLM1a	TNL	Chi	CARHR048060	60.398	553	160	709	0	NL	different
At_WRR4b	TNL	Chi	CARHR047100	60.341	469	1	469	0	TN	different
At_RPP4	TNL	Chi	CARHR222470	60.249	722	5	722	0	TNL	same
Bra_Crr1a	TNL	Chi	CARHR202050	60.244	737	68	796	0	NL	different
Bju_WRR1	CNL	Chi	CARHR011140	60.154	911	1	900	0	CNL	same
At_RPS4	TNL	Chi	CARHR177620	60.087	1150	8	1139	0	TNL	same
At_RAC1	TNL	Csa	Csa11g065490	68.354	158	160	317	1.57E-53	NL	different
Bna_Rlm9/4/7	Other-RLK	Csa	Csa14g020270	65.882	170	588	757	1.52E-54	Other-RLK	same
At_WRR8	TNL	Csa	Csa11g065490	68.987	158	170	327	8.53E-56	NL	different
At_RFO2	LRR-RLP	Csa	Csa16g034990	64.815	162	576	737	3.22E-56	LRR-RLK	different
At_RPS5	TNL	Csa	Csa03g016040	70.667	150	140	289	3.89E-58	CNL	different
At_ADR1	NL	Csa	Csa05g083250	61.236	178	214	391	2.14E-63	NBS	different
At_RPP7	NL	Csa	Csa05g052490	61.878	181	126	306	1.26E-64	NL	same
At_RPS5	TNL	Csa	Csa04404s010	64.571	175	434	605	1.55E-66	NL	different
At_RFO1	Other-RLK	Csa	Csa14g020270	72.892	166	555	720	1.19E-68	Other-RLK	same
Bna_Rlm9/4/7	Other-RLK	Csa	Csa07g058810	68.627	204	594	793	5.66E-70	Other-RLK	same
At_RFO1	Other-RLK	Csa	Csa09g093940	66.667	183	519	701	3.12E-77	Other-RLK	same
At_ADR1	NL	Csa	Csa16g035790	60.952	210	214	423	3.08E-79	NBS	different
At_RPS5	TNL	Csa	Csa14g015220	60.569	246	387	623	4.08E-80	CNL	different
At_RLP23	LRR-RLP	Csa	Csa15g052710	73.333	195	693	887	5.29E-82	LRR-RLP	same
At_RLP23	LRR-RLP	Csa	Csa15g052710	73.333	195	693	887	5.29E-82	LRR-RLP	same
At_RPP2b	TNL	Csa	Csa12g042110	77.439	164	465	628	2.04E-86	NL	different
At_NGR1a	RNL	Csa	Csa03087s010	71.845	206	269	467	8.77E-90	NBS	different
At_RFO3	Other-RLK	Csa	Csa11g022890	64.815	216	515	730	2.26E-91	Other-RLK	same
At_RPP2a	TNL	Csa	Csa10g028540	61.569	255	161	414	5.04E-93	NL	different
At_RPP2b	TNL	Csa	Csa03866s010	73.659	205	179	382	4.53E-93	NBS	different
At_RLP42	LRR-RLP	Csa	Csa15g052710	79.275	193	693	880	2.59E-94	LRR-RLP	same
At_RLP42	LRR-RLP	Csa	Csa15g052710	79.275	193	693	880	2.59E-94	LRR-RLP	same
Bju_WRR1	CNL	Csa	Csa11g050760	60.547	256	1	256	4.10E-101	CN	different
Bna_LepR3/Rlm2	LRR-RLP	Csa	Csa19g015120	63.88	299	502	794	1.45E-102	LRR-RLP	same
Bna_LepR3/Rlm2	LRR-RLP	Csa	Csa19g015120	63.88	299	502	794	1.45E-102	LRR-RLP	same
At_NGR1b	RNL	Csa	Csa03087s010	81.193	218	259	472	2.36E-105	NBS	different
Bna_Rlm9/4/7	Other-RLK	Csa	Csa09g093940	76.543	243	552	794	2.02E-105	Other-RLK	same
At_RPM1	NL	Csa	Csa01g008050	60.703	313	615	926	1.86E-105	NL	same
At_RPP39	CNL	Csa	Csa16g055550	73.568	227	403	629	1.04E-105	CNL	same
At_RPP8	CNL	Csa	Csa11g050760	65.385	260	1	260	2.32E-110	CN	different
At_RPP39	CNL	Csa	Csa16g055550	76.569	239	380	618	1.58E-118	CNL	same
At_RAC1	TNL	Csa	Csa07g030100	63.522	318	160	474	8.12E-121	NL	different
At_RPP39	CNL	Csa	Csa17g016520	60.355	338	236	568	9.97E-125	CNL	same
At_RPS5	TNL	Csa	Csa17g016100	65.449	301	1	299	2.11E-126	NBS	different
At_RLM1a	TNL	Csa	Csa11g038780	62.046	303	319	621	1.53E-126	NL	different
At_RPP8	CNL	Csa	Csa11g074780	64.198	324	585	908	1.04E-127	CNL	same
At_BAK1	LRR-RLK	Csa	Csa10g008830	99.471	189	427	615	2.75E-128	LRR-RLK	same
At_RFO1	Other-RLK	Csa	Csa14g022240	65.854	287	438	723	1.05E-128	Other-RLK	same
Bna_Rlm9/4/7	Other-RLK	Csa	Csa14g022240	72.593	270	469	737	8.68E-129	Other-RLK	same
At_RLP32	LRR-RLP	Csa	Csa19g015120	72.575	299	415	712	4.32E-129	LRR-RLP	same
At_RLP32	LRR-RLP	Csa	Csa19g015120	72.575	299	415	712	4.32E-129	LRR-RLP	same
At_RPP8	CNL	Csa	Csa20g041580	65.616	349	562	906	3.53E-137	CNL	same
Bju_WRR1	CNL	Csa	Csa10g029780	61.862	333	1	333	8.34E-140	CNL	same
At_RFO1	Other-RLK	Csa	Csa09g093940	87.649	251	50	296	3.36E-144	Other-RLK	same

Bju_WRR1	CNL	Csa	Csa03g013720	63.636	341	1	336	6.39E-146	CNL	same
At_NGR1b	RNL	Csa	Csa11g104020	62.716	405	415	815	6.62E-154	CNL	different
At_RPP8	CNL	Csa	Csa10g029780	64.723	343	1	343	5.31E-155	CNL	same
At_WRR12	TNL	Csa	Csa03g021400	84.588	279	393	652	1.51E-156	LRR-RLP	different
At_WRR12	TNL	Csa	Csa03g021400	84.588	279	393	652	1.51E-156	LRR-RLP	different
At_NGR1a	RNL	Csa	Csa11g104020	63.184	402	413	809	1.03E-156	CNL	different
At_RPP1	TNL	Csa	Csa09g050290	60.879	478	684	1152	2.32E-159	CNL	different
At_RPP8	CNL	Csa	Csa03g013720	68.915	341	1	341	3.99E-165	CNL	same
At_RFO1	Other-RLK	Csa	Csa09g093940	62.879	396	275	667	2.74E-165	Other-RLK	same
At_RPS5	TNL	Csa	Csa14g015130	60.584	411	1	411	7.41E-168	CN	different
Bju_WRR1	CNL	Csa	Csa20g041580	67.196	378	1	372	1.44E-169	CNL	same
At_RPP13	CNL	Csa	Csa06g020520	72.022	361	103	460	5.44E-170	NBS	different
At_RLP42	LRR-RLP	Csa	Csa05g023540	65.116	387	392	777	3.86E-171	LRR-RLP	same
At_RLP42	LRR-RLP	Csa	Csa05g023540	65.116	387	392	777	3.86E-171	LRR-RLP	same
At_RFO1	Other-RLK	Csa	Csa14g034030	61.809	398	360	751	1.14E-171	Other-RLK	same
At_RLP23	LRR-RLP	Csa	Csa05g023540	65.633	387	392	777	1.11E-174	LRR-RLP	same
At_RLP23	LRR-RLP	Csa	Csa05g023540	65.633	387	392	777	1.11E-174	LRR-RLP	same
At_RPS5	TNL	Csa	Csa03g016040	67.176	393	1	393	7.10E-176	CNL	different
At_BAK1	LRR-RLK	Csa	Csa03g038390	72.178	381	9	377	3.03E-176	LRR-RLK	same
At_BAK1	LRR-RLK	Csa	Csa12g011400	98.699	615	1	615	0	LRR-RLK	same
At_BAK1	LRR-RLK	Csa	Csa11g009710	98.699	615	1	615	0	LRR-RLK	same
At_BAK1	LRR-RLK	Csa	Csa11g009710	97.276	624	1	615	0	LRR-RLK	same
At_RFO1	Other-RLK	Csa	Csa09g093940	89.452	730	29	751	0	Other-RLK	same
At_RFO1	Other-RLK	Csa	Csa16g049330	89.373	734	24	751	0	Other-RLK	same
At_FLS2	LRR-RLK	Csa	Csa18g003700	89.325	1171	1	1168	0	LRR-RLK	same
At_FLS2	LRR-RLK	Csa	Csa11g065650	88.682	804	367	1167	0	LRR-RLK	same
At_RFO2	LRR-RLP	Csa	Csa17g022930	88.228	756	1	755	0	LRR-RLP	same
At_RFO2	LRR-RLP	Csa	Csa17g022930	88.228	756	1	755	0	LRR-RLP	same
At_ADR1	NL	Csa	Csa17g056910	88.184	457	1	455	0	NBS	different
At_RPS2	NL	Csa	Csa10g016820	87.671	657	253	909	0	CNL	different
At_SOBIR1	LRR-RLK	Csa	Csa07g010930	87.072	642	1	641	0	LRR-RLK	same
At_BAK1	LRR-RLK	Csa	Csa12g011400	85.547	685	1	615	0	LRR-RLK	same
At_RLP32	LRR-RLP	Csa	Csa01g006540	85.434	865	1	860	0	LRR-RLP	same
At_RLP32	LRR-RLP	Csa	Csa01g006540	85.434	865	1	860	0	LRR-RLP	same
At_ADR1	NL	Csa	Csa14g043540	85.316	790	1	787	0	NL	same
At_RLP32	LRR-RLP	Csa	Csa19g008130	85.269	835	1	830	0	LRR-RLP	same
At_RLP32	LRR-RLP	Csa	Csa19g008130	85.269	835	1	830	0	LRR-RLP	same
At_RLP32	LRR-RLP	Csa	Csa15g007010	85.047	856	1	852	0	LRR-RLP	same
At_RLP32	LRR-RLP	Csa	Csa15g007010	85.047	856	1	852	0	LRR-RLP	same
At_BAK1	LRR-RLK	Csa	Csa10g008830	83.56	663	1	612	0	LRR-RLK	same
At_FLS2	LRR-RLK	Csa	Csa20g079470	83.543	1112	73	1167	0	LRR-RLK	same
At_RPS2	NL	Csa	Csa11g018310	82.513	955	1	909	0	CNL	different
At_RPS2	NL	Csa	Csa10g016820	81.893	486	1	480	0	CNL	different
At_BAK1	LRR-RLK	Csa	Csa15g064420	81.728	602	17	615	0	LRR-RLK	same
At_RPP8	CNL	Csa	Csa20g041580	81.627	381	1	380	0	CNL	same
At_RLP30	LRR-RLP	Csa	Csa19g007700	81.599	788	1	786	0	LRR-RLP	same
At_RLP30	LRR-RLP	Csa	Csa19g007700	81.599	788	1	786	0	LRR-RLP	same
At_RLP30	LRR-RLP	Csa	Csa15g006630	79.716	774	16	786	0	LRR-RLP	same
At_RLP30	LRR-RLP	Csa	Csa15g006630	79.716	774	16	786	0	LRR-RLP	same
At_RPP13	CNL	Csa	Csa09g042600	79.636	604	1	592	0	NBS	different
At_NGR1b	RNL	Csa	Csa18g040610	79.63	756	5	753	0	NL	different
At_BAK1	LRR-RLK	Csa	Csa07g038730	79.504	605	24	615	0	LRR-RLK	same
At_BAK1	LRR-RLK	Csa	Csa09g075150	79.504	605	24	615	0	LRR-RLK	same
At_BAK1	LRR-RLK	Csa	Csa16g034450	79.504	605	24	615	0	LRR-RLK	same
At_RLP42	LRR-RLP	Csa	Csa01g031010	79.274	854	17	869	0	LRR-RLP	same
At_RLP42	LRR-RLP	Csa	Csa01g031010	79.274	854	17	869	0	LRR-RLP	same
At_RLP42	LRR-RLP	Csa	Csa19g036840	78.463	859	25	880	0	LRR-RLP	same
At_RLP42	LRR-RLP	Csa	Csa19g036840	78.463	859	25	880	0	LRR-RLP	same
At_WRR12	TNL	Csa	Csa03g021400	78.365	587	463	1048	0	LRR-RLP	different
At_WRR12	TNL	Csa	Csa03g021400	78.365	587	463	1048	0	LRR-RLP	different
At_RFO1	Other-RLK	Csa	Csa07g058810	78.31	710	50	751	0	Other-RLK	same
At_WRR12	TNL	Csa	Csa14g021890	78.174	701	355	1035	0	NL	different
At_RPP39	CNL	Csa	Csa09g095740	77.914	326	1	326	0	CN	different
Bna_Rlm9/4/7	Other-RLK	Csa	Csa09g093940	77.859	411	286	693	0	Other-RLK	same
At_RPP8	CNL	Csa	Csa18g014750	77.79	914	1	906	0	CNL	same
At_NGR1b	RNL	Csa	Csa11g104020	77.119	826	5	790	0	CNL	different
At_BAK1	LRR-RLK	Csa	Csa17g059530	76.414	619	9	615	0	LRR-RLK	same
At_RLP30	LRR-RLP	Csa	Csa01g006210	74.968	783	1	782	0	LRR-RLP	same
At_RLP30	LRR-RLP	Csa	Csa01g006210	74.968	783	1	782	0	LRR-RLP	same
At_RPP8	CNL	Csa	Csa11g074780	74.73	463	1	462	0	CNL	same
At_RPP39	CNL	Csa	Csa16g053080	74.706	340	1	340	0	CN	different
At_WRR4b	TNL	Csa	Csa07g026440	74.641	765	315	1073	0	NL	different
At_RFO1	Other-RLK	Csa	Csa07g058680	74.006	704	55	751	0	Other-RLK	same
At_RFO3	Other-RLK	Csa	Csa19g022860	73.767	892	1	850	0	Other-RLK	same
At_RFO3	Other-RLK	Csa	Csa15g020770	73.359	777	19	773	0	Other-RLK	same
At_RPP39	CNL	Csa	Csa07g062500	73.273	550	1	548	0	CNL	same
At_RPP39	CNL	Csa	Csa07g062500	73.273	550	1	548	0	CNL	same
At_WRR4b	TNL	Csa	Csa17g098890	73.038	586	494	1072	0	LRR-RLP	different
At_WRR4b	TNL	Csa	Csa17g098890	73.038	586	494	1072	0	LRR-RLP	different
At_RFO3	Other-RLK	Csa	Csa05g081570	72.306	863	1	850	0	Other-RLK	same
At_RFO2	LRR-RLP	Csa	Csa07g039390	72.131	549	189	737	0	LRR-RLK	different
At_RFO2	LRR-RLP	Csa	Csa14g021470	72.043	744	6	749	0	LRR-RLP	same
At_RFO2	LRR-RLP	Csa	Csa14g021470	72.043	744	6	749	0	LRR-RLP	same
At_RFO2	LRR-RLP	Csa	Csa16g034990	72.029	690	48	737	0	LRR-RLK	different
Bna_Rlm9/4/7	Other-RLK	Csa	Csa01g034720	71.681	791	22	794	0	Other-RLK	same
At_RPP13	CNL	Csa	Csa06g020300	71.678	858	1	834	0	NL	different

Bna_Rlm9/4/7	Other-RLK	Csa	Csa19g050880	71.648	783	22	794	0	Other-RLK	same
At_RPP39	CNL	Csa	Csa09g095770	71.361	845	42	871	0	CNL	same
At_RPP39	CNL	Csa	Csa07g062500	71.268	891	1	871	0	CNL	same
At_BAK1	LRR-RLK	Csa	Csa14g044100	71.254	574	97	615	0	LRR-RLK	same
At_RPP39	CNL	Csa	Csa07g062500	71.124	890	1	871	0	CNL	same
At_RPP39	CNL	Csa	Csa07g062500	71.124	890	1	871	0	CNL	same
At_RFO3	Other-RLK	Csa	Csa16g027360	71.101	872	1	850	0	Other-RLK	same
At_RLP32	LRR-RLP	Csa	Csa19g013610	70.952	630	228	854	0	LRR-RLP	same
At_RLP32	LRR-RLP	Csa	Csa19g013610	70.952	630	228	854	0	LRR-RLP	same
At_RPP8	CNL	Csa	Csa14g013640	70.46	914	1	908	0	CNL	same
At_RPP8	CNL	Csa	Csa17g093690	70.273	915	1	906	0	CNL	same
At_RPP39	CNL	Csa	Csa07g062360	70.056	885	1	870	0	CNL	same
At_NGR1a	RNL	Csa	Csa18g040610	70.039	761	1	747	0	NL	different
At_RPP39	CNL	Csa	Csa09g095730	70.034	881	1	871	0	CNL	same
At_RPP8	CNL	Csa	Csa12g074470	69.934	908	1	905	0	CNL	same
At_RFO2	LRR-RLP	Csa	Csa09g075890	69.783	738	1	737	0	LRR-RLK	different
Bna_Rlm9/4/7	Other-RLK	Csa	Csa07g036470	69.466	786	22	794	0	Other-RLK	same
At_RFO2	LRR-RLP	Csa	Csa03g021020	69.231	741	1	740	0	LRR-RLP	same
At_RFO2	LRR-RLP	Csa	Csa03g021020	69.231	741	1	740	0	LRR-RLP	same
At_RPP39	CNL	Csa	Csa07g062520	69.221	809	81	877	0	NL	different
At_RLP23	LRR-RLP	Csa	Csa05g024050	68.899	881	17	890	0	LRR-RLP	same
At_RLP23	LRR-RLP	Csa	Csa05g024050	68.899	881	17	890	0	LRR-RLP	same
At_RPP39	CNL	Csa	Csa09g095890	68.787	849	42	877	0	CNL	same
At_RLM3	TN	Csa	Csa12g049800	68.736	435	155	548	0	NBS	different
At_RPP13	CNL	Csa	Csa09g043970	68.458	856	1	834	0	NL	different
At_NGR1a	RNL	Csa	Csa11g104020	68.231	831	1	784	0	CNL	different
At_RPP39	CNL	Csa	Csa07g062370	68.182	462	424	871	0	LRR-RLP	different
At_RPP39	CNL	Csa	Csa07g062370	68.182	462	424	871	0	LRR-RLP	different
Bna_Rlm9/4/7	Other-RLK	Csa	Csa16g031260	68.164	757	50	794	0	Other-RLK	same
At_RFO2	LRR-RLP	Csa	Csa17g022950	68.151	741	1	740	0	LRR-RLP	same
At_RFO2	LRR-RLP	Csa	Csa17g022950	68.151	741	1	740	0	LRR-RLP	same
Bna_LepR3/Rlm2	LRR-RLP	Csa	Csa19g046270	67.886	629	323	947	0	LRR-RLP	same
Bna_LepR3/Rlm2	LRR-RLP	Csa	Csa19g046270	67.886	629	323	947	0	LRR-RLP	same
At_RLP23	LRR-RLP	Csa	Csa01g031010	67.87	859	18	875	0	LRR-RLP	same
At_RLP23	LRR-RLP	Csa	Csa01g031010	67.87	859	18	875	0	LRR-RLP	same
At_RPP7	NL	Csa	Csa05g052240	67.727	880	1	866	0	NL	same
At_RLP23	LRR-RLP	Csa	Csa05g024010	67.698	808	84	890	0	LRR-RLP	same
At_RLP23	LRR-RLP	Csa	Csa05g024010	67.698	808	84	890	0	LRR-RLP	same
Bna_Rlm9/4/7	Other-RLK	Csa	Csa07g058810	67.643	683	24	699	0	Other-RLK	same
At_RLP23	LRR-RLP	Csa	Csa19g036840	67.552	866	25	887	0	LRR-RLP	same
At_RLP23	LRR-RLP	Csa	Csa19g036840	67.552	866	25	887	0	LRR-RLP	same
At_RLP32	LRR-RLP	Csa	Csa19g046270	67.528	619	234	852	0	LRR-RLP	same
At_RLP32	LRR-RLP	Csa	Csa19g046270	67.528	619	234	852	0	LRR-RLP	same
At_RLP42	LRR-RLP	Csa	Csa05g024050	67.506	874	18	880	0	LRR-RLP	same
At_RLP42	LRR-RLP	Csa	Csa05g024050	67.506	874	18	880	0	LRR-RLP	same
At_RPP8	CNL	Csa	Csa03g060600	66.914	674	1	668	0	CNL	same
At_RPP39	CNL	Csa	Csa16g053190	66.894	879	1	869	0	CNL	same
At_RLP32	LRR-RLP	Csa	Csa19g008130	66.822	859	29	860	0	LRR-RLP	same
At_RLP32	LRR-RLP	Csa	Csa19g008130	66.822	859	29	860	0	LRR-RLP	same
At_RFO1	Other-RLK	Csa	Csa03g019890	66.809	705	5	699	0	Other-RLK	same
At_RPS5	TNL	Csa	Csa14g015220	66.757	737	1	736	0	CNL	different
At_BAK1	LRR-RLK	Csa	Csa19g043470	66.729	538	17	549	0	LRR-RLK	same
At_BAK1	LRR-RLK	Csa	Csa19g043470	66.729	538	17	549	0	LRR-RLK	same
At_RLP32	LRR-RLP	Csa	Csa15g007010	66.525	708	66	748	0	LRR-RLP	same
At_RLP32	LRR-RLP	Csa	Csa15g007010	66.525	708	66	748	0	LRR-RLP	same
Bna_Rlm9/4/7	Other-RLK	Csa	Csa14g034030	66.514	436	359	793	0	Other-RLK	same
At_RFO1	Other-RLK	Csa	Csa17g021700	66.243	708	13	704	0	Other-RLK	same
At_RPP8	CNL	Csa	Csa14g063890	65.939	916	1	906	0	CNL	same
At_RPS5	TNL	Csa	Csa17g016500	65.876	885	1	884	0	CNL	different
At_RFO1	Other-RLK	Csa	Csa17g021720	65.869	627	133	747	0	Other-RLK	same
At_RPP39	CNL	Csa	Csa09g095760	65.847	691	1	683	0	CNL	same
At_RPP39	CNL	Csa	Csa09g095750	65.798	883	1	873	0	CNL	same
At_RLP32	LRR-RLP	Csa	Csa15g014680	65.783	868	1	854	0	LRR-RLP	same
At_RLP32	LRR-RLP	Csa	Csa15g014680	65.783	868	1	854	0	LRR-RLP	same
At_RPP39	CNL	Csa	Csa16g055540	65.684	848	1	821	0	CNL	same
At_RLP32	LRR-RLP	Csa	Csa19g014830	65.471	892	1	854	0	LRR-RLP	same
At_RLP32	LRR-RLP	Csa	Csa19g014830	65.471	892	1	854	0	LRR-RLP	same
At_RPS5	TNL	Csa	Csa10g031910	65.471	892	1	883	0	NL	different
At_RFO1	Other-RLK	Csa	Csa03g019890	65.444	709	10	699	0	Other-RLK	same
At_RLP23	LRR-RLP	Csa	Csa07g008160	65.438	868	22	883	0	LRR-RLP	same
At_RLP23	LRR-RLP	Csa	Csa07g008160	65.438	868	22	883	0	LRR-RLP	same
At_RLP32	LRR-RLP	Csa	Csa15g014440	65.389	835	38	830	0	LRR-RLP	same
At_RLP32	LRR-RLP	Csa	Csa15g014440	65.389	835	38	830	0	LRR-RLP	same
At_RLP32	LRR-RLP	Csa	Csa19g015110	65.264	832	80	854	0	LRR-RLP	same
At_RLP32	LRR-RLP	Csa	Csa19g015110	65.264	832	80	854	0	LRR-RLP	same
At_FLS2	LRR-RLK	Csa	Csa20g079470	65.256	780	24	764	0	LRR-RLK	same
At_RFO1	Other-RLK	Csa	Csa17g021700	65.158	729	10	718	0	Other-RLK	same
At_RFO1	Other-RLK	Csa	Csa14g020260	65.154	749	18	751	0	Other-RLK	same
Bna_Rlm9/4/7	Other-RLK	Csa	Csa05g087560	65.071	773	41	794	0	Other-RLK	same
At_RLP23	LRR-RLP	Csa	Csa16g008260	64.854	717	175	889	0	LRR-RLP	same
At_RLP23	LRR-RLP	Csa	Csa16g008260	64.854	717	175	889	0	LRR-RLP	same
At_RPP8	CNL	Csa	Csa03g013720	64.73	482	431	907	0	CNL	same
At_RLP23	LRR-RLP	Csa	Csa16g008000	64.673	719	175	888	0	LRR-RLP	same
At_RLP23	LRR-RLP	Csa	Csa16g008000	64.673	719	175	888	0	LRR-RLP	same
At_RPP13	CNL	Csa	Csa04g029590	64.596	853	1	834	0	NL	different
At_RLP32	LRR-RLP	Csa	Csa15g014660	64.567	889	1	854	0	LRR-RLP	same

At_RLP32	LRR-RLP	Csa	Csa15g014660	64.567	889	1	854	0	LRR-RLP	same
At_RPP8	CNL	Csa	Csa12g053350	64.466	909	1	904	0	CNL	same
Bna_LepR3/Rlm2	LRR-RLP	Csa	Csa19g013610	64.275	641	317	947	0	LRR-RLP	same
Bna_LepR3/Rlm2	LRR-RLP	Csa	Csa19g013610	64.275	641	317	947	0	LRR-RLP	same
At_RLP32	LRR-RLP	Csa	Csa19g015030	64.27	890	1	854	0	LRR-RLP	same
At_RLP32	LRR-RLP	Csa	Csa19g015030	64.27	890	1	854	0	LRR-RLP	same
At_RLP32	LRR-RLP	Csa	Csa19g014850	64.267	778	114	853	0	LRR-RLP	same
At_RLP32	LRR-RLP	Csa	Csa19g014850	64.267	778	114	853	0	LRR-RLP	same
At_RLP32	LRR-RLP	Csa	Csa01g012020	64.123	878	29	846	0	LRR-RLP	same
At_RLP32	LRR-RLP	Csa	Csa01g012020	64.123	878	29	846	0	LRR-RLP	same
At_RLP23	LRR-RLP	Csa	Csa05g023150	64.078	721	175	888	0	LRR-RLP	same
At_RLP23	LRR-RLP	Csa	Csa05g023150	64.078	721	175	888	0	LRR-RLP	same
At_RLP23	LRR-RLP	Csa	Csa05g023150	64.078	721	175	888	0	LRR-RLP	same
At_RLP23	LRR-RLP	Csa	Csa05g023150	64.078	721	175	888	0	LRR-RLP	same
At_RFO1	Other-RLK	Csa	Csa03g019890	64.058	754	10	751	0	Other-RLK	same
Bna_MPK9	Other-RLK	Csa	Csa09g002650	63.91	532	100	564	0	Other-RLK	same
At_RFO1	Other-RLK	Csa	Csa14g020250	63.821	738	5	721	0	Other-RLK	same
At_RFO1	Other-RLK	Csa	Csa17g021720	63.525	732	12	720	0	Other-RLK	same
At_RLP32	LRR-RLP	Csa	Csa15g014440	63.474	783	55	832	0	LRR-RLP	same
At_RLP32	LRR-RLP	Csa	Csa15g014440	63.474	783	55	832	0	LRR-RLP	same
At_RPP7	NL	Csa	Csa16g018240	63.314	875	1	869	0	NL	same
At_RPP8	CNL	Csa	Csa03g014560	63.293	741	27	761	0	CNL	same
At_RRS1	TNL	Csa	Csa11g068440	63.246	1189	6	1158	0	NL	different
Bna_LepR3/Rlm2	LRR-RLP	Csa	Csa15g007010	63.194	739	204	937	0	LRR-RLP	same
Bna_LepR3/Rlm2	LRR-RLP	Csa	Csa15g007010	63.194	739	204	937	0	LRR-RLP	same
At_RFO1	Other-RLK	Csa	Csa14g020240	63.172	763	1	741	0	Other-RLK	same
At_RLP42	LRR-RLP	Csa	Csa16g008000	63.083	707	182	880	0	LRR-RLP	same
At_RLP42	LRR-RLP	Csa	Csa16g008000	63.083	707	182	880	0	LRR-RLP	same
Bna_LepR3/Rlm2	LRR-RLP	Csa	Csa01g006540	63.017	749	204	942	0	LRR-RLP	same
Bna_LepR3/Rlm2	LRR-RLP	Csa	Csa01g006540	63.017	749	204	942	0	LRR-RLP	same
At_RLP42	LRR-RLP	Csa	Csa07g008160	62.993	862	22	876	0	LRR-RLP	same
At_RLP42	LRR-RLP	Csa	Csa07g008160	62.993	862	22	876	0	LRR-RLP	same
At_RPP7	NL	Csa	Csa05g052410	62.916	782	1	776	0	NL	same
At_RPP8	CNL	Csa	Csa03g060600	62.867	579	335	908	0	CNL	same
Bna_LepR3/Rlm2	LRR-RLP	Csa	Csa15g007010	62.84	662	176	831	0	LRR-RLP	same
Bna_LepR3/Rlm2	LRR-RLP	Csa	Csa15g007010	62.84	662	176	831	0	LRR-RLP	same
At_ADR1	NL	Csa	Csa20g005920	62.822	815	6	787	0	CNL	different
At_RLP42	LRR-RLP	Csa	Csa05g023150	62.764	709	182	880	0	LRR-RLP	same
At_RLP42	LRR-RLP	Csa	Csa05g023150	62.764	709	182	880	0	LRR-RLP	same
At_RLP42	LRR-RLP	Csa	Csa05g023150	62.764	709	182	880	0	LRR-RLP	same
At_RLP42	LRR-RLP	Csa	Csa05g023150	62.764	709	182	880	0	LRR-RLP	same
Bna_LepR3/Rlm2	LRR-RLP	Csa	Csa19g008130	62.743	773	176	942	0	LRR-RLP	same
Bna_LepR3/Rlm2	LRR-RLP	Csa	Csa19g008130	62.743	773	176	942	0	LRR-RLP	same
At_WRR12	TNL	Csa	Csa12g079810	62.736	636	347	981	0	NL	different
At_RLP42	LRR-RLP	Csa	Csa16g008260	62.674	718	182	889	0	LRR-RLP	same
At_RLP42	LRR-RLP	Csa	Csa16g008260	62.674	718	182	889	0	LRR-RLP	same
At_WRR12	TNL	Csa	Csa14g021930	62.637	637	374	1010	0	NL	different
Bju_WRR1	CNL	Csa	Csa17g093690	62.623	915	1	900	0	CNL	same
At_RLP32	LRR-RLP	Csa	Csa01g012100	62.613	888	1	846	0	LRR-RLP	same
At_RLP32	LRR-RLP	Csa	Csa01g012100	62.613	888	1	846	0	LRR-RLP	same
At_ADR1	NL	Csa	Csa08g059780	62.546	817	6	787	0	CNL	different
Bna_LepR3/Rlm2	LRR-RLP	Csa	Csa19g008130	62.327	722	204	915	0	LRR-RLP	same
Bna_LepR3/Rlm2	LRR-RLP	Csa	Csa19g008130	62.327	722	204	915	0	LRR-RLP	same
At_RPP13	CNL	Csa	Csa09g043950	62.284	867	1	830	0	CNL	same
At_RPS5	TNL	Csa	Csa17g016520	62.116	879	1	874	0	CNL	different
At_RLP32	LRR-RLP	Csa	Csa19g014810	62.099	934	1	868	0	LRR-RLP	same
At_RLP32	LRR-RLP	Csa	Csa19g014810	62.099	934	1	868	0	LRR-RLP	same
At_RLP32	LRR-RLP	Csa	Csa19g014880	62.09	823	1	765	0	LRR-RLP	same
At_RLP32	LRR-RLP	Csa	Csa19g014880	62.09	823	1	765	0	LRR-RLP	same
At_RLM1b	TNL	Csa	Csa07g063320	62.061	883	161	998	0	NL	different
At_RPS5	TNL	Csa	Csa17g016520	62.027	582	308	883	0	CNL	different
At_ADR1	NL	Csa	Csa13g006680	61.74	724	6	695	0	CNL	different
Bna_LepR3/Rlm2	LRR-RLP	Csa	Csa15g014440	61.73	763	176	921	0	LRR-RLP	same
Bna_LepR3/Rlm2	LRR-RLP	Csa	Csa15g014440	61.73	763	176	921	0	LRR-RLP	same
Bju_WRR1	CNL	Csa	Csa12g074470	61.648	910	1	900	0	CNL	same
Bna_LepR3/Rlm2	LRR-RLP	Csa	Csa15g014680	61.598	776	187	950	0	LRR-RLP	same
Bna_LepR3/Rlm2	LRR-RLP	Csa	Csa15g014680	61.598	776	187	950	0	LRR-RLP	same
Bju_WRR1	CNL	Csa	Csa03g060600	61.573	674	1	667	0	CNL	same
At_RLP32	LRR-RLP	Csa	Csa19g015120	61.555	926	34	853	0	LRR-RLP	same
At_RLP32	LRR-RLP	Csa	Csa19g015120	61.555	926	34	853	0	LRR-RLP	same
At_RFO1	Other-RLK	Csa	Csa03g019890	61.528	707	35	725	0	Other-RLK	same
Bju_WRR1	CNL	Csa	Csa18g014750	61.379	914	1	900	0	CNL	same
At_RLP32	LRR-RLP	Csa	Csa19g015010	61.148	610	252	852	0	LRR-RLP	same
At_RLP32	LRR-RLP	Csa	Csa19g015010	61.148	610	252	852	0	LRR-RLP	same
Bna_Rlm9/4/7	Other-RLK	Csa	Csa03g019890	60.676	740	22	748	0	Other-RLK	same
Bju_WRR1	CNL	Csa	Csa14g013640	60.593	911	1	900	0	CNL	same
At_RPS5	TNL	Csa	Csa17g016520	60.541	887	1	881	0	CNL	different
At_RLP32	LRR-RLP	Csa	Csa19g014580	60.535	935	1	854	0	LRR-RLP	same
At_RLP32	LRR-RLP	Csa	Csa19g014580	60.535	935	1	854	0	LRR-RLP	same
Bna_LepR3/Rlm2	LRR-RLP	Csa	Csa19g015120	60.515	932	26	938	0	LRR-RLP	same
Bna_LepR3/Rlm2	LRR-RLP	Csa	Csa19g015120	60.515	932	26	938	0	LRR-RLP	same
At_RLM1a	TNL	Csa	Csa07g023960	60.342	643	367	997	0	NL	different
At_RLP23	LRR-RLP	Csa	Csa05g023480	60.332	784	175	889	0	LRR-RLP	same
At_RLP23	LRR-RLP	Csa	Csa05g023480	60.332	784	175	889	0	LRR-RLP	same
At_RPS5	TNL	Csa	Csa17g016520	60.311	514	27	538	0	CNL	different
At_RPP13	CNL	Csa	Csa04g029570	60.23	870	1	830	0	CNL	same

At_RLP32	LRR-RLP	Csa	Csa19g014800	60.225	890	1	854	0	LRR-RLP	same
At_RLP32	LRR-RLP	Csa	Csa19g014800	60.225	890	1	854	0	LRR-RLP	same
At_RLP23	LRR-RLP	Csa	Csa05g024200	60.141	710	175	880	0	LRR-RLP	same
At_RLP23	LRR-RLP	Csa	Csa05g024200	60.141	710	175	880	0	LRR-RLP	same
At_RPP7	NL	Csa	Csa16g018250	60.114	875	1	866	0	NL	same
At_RPP1	TNL	Aha	Araha.4751s0001.1	60.403	149	94	242	1.07E-52	TN	different
At_RPP5	TNL	Aha	Araha.25586s0003.1	61.074	149	11	159	1.75E-55	TX	different
Bol_FocBo1	TNL	Aha	Araha.25586s0003.1	60.135	148	7	154	1.06E-55	TX	different
At_RPP5	TNL	Aha	Araha.66955s0001.1	60.784	153	3	155	7.73E-56	TX	different
At_WRR4a	TNL	Aha	Araha.66955s0001.1	61.184	152	1	152	1.97E-56	TX	different
At_RLM1a	TNL	Aha	Araha.66955s0001.1	60.135	148	7	154	9.82E-59	TX	different
Bra_cRa/cRb	TNL	Aha	Araha.66955s0001.1	62.838	148	68	215	2.86E-59	TX	different
At_RPP5	TNL	Aha	Araha.1430s0001.1	61.745	149	11	159	2.34E-59	TX	different
Bol_FocBo1	TNL	Aha	Araha.66955s0001.1	63.758	149	5	153	1.36E-59	TX	different
Bra_cRa/cRb	TNL	Aha	Araha.4751s0001.1	70.47	149	71	219	2.12E-60	TN	different
At_WRR4b	TNL	Aha	Araha.66827s0001.1	62.162	148	11	158	5.94E-61	TX	different
At_WRR4b	TNL	Aha	Araha.66955s0001.1	62.658	158	1	158	4.31E-61	TX	different
Bol_FocBo1	TNL	Aha	Araha.59926s0001.1	60.377	159	9	167	6.50E-62	TX	different
At_WRR9	NL	Aha	Araha.59926s0001.1	61.635	159	17	175	2.04E-63	TX	different
Bra_Crr1a	TNL	Aha	Araha.59926s0001.1	62.025	158	70	227	1.31E-64	TX	different
At_RPP1	TNL	Aha	Araha.5406s0003.1	66.86	172	64	235	5.47E-68	TX	different
At_RPP1	TNL	Aha	Araha.59926s0001.1	64.78	159	97	255	7.26E-69	TX	different
At_WRR4a	TNL	Aha	Araha.59926s0001.1	64.706	170	1	170	1.85E-69	TX	different
Bra_Crr1a	TNL	Aha	Araha.4751s0001.1	77.181	149	66	214	1.04E-69	TN	different
At_WRR9	NL	Aha	Araha.38723s0001.1	61.5	200	5	204	5.69E-70	TX	different
At_RLM1b	TNL	Aha	Araha.25586s0003.1	70.47	149	10	158	8.58E-71	TX	different
At_RLM1b	TNL	Aha	Araha.59926s0001.1	66.082	171	1	171	4.53E-71	TX	different
At_RPP1	TNL	Aha	Araha.38723s0001.1	61.809	199	87	285	4.93E-74	TX	different
At_WRR4b	TNL	Aha	Araha.59926s0001.1	66.279	172	1	172	2.72E-74	TX	different
At_RFO3	Other-RLK	Aha	Araha.3018s0001.1	63.687	179	515	693	1.83E-74	Other-RLK	same
At_WRR4a	TNL	Aha	Araha.38723s0001.1	63.317	199	1	199	1.83E-74	TX	different
At_WRR4a	TNL	Aha	Araha.25586s0003.1	74.497	149	9	157	1.51E-74	TX	different
At_RPP4	TNL	Aha	Araha.18217s0002.1	69.588	194	1	190	6.67E-75	TN	different
At_RLM1a	TNL	Aha	Araha.59926s0001.1	67.456	169	3	171	3.41E-75	TX	different
At_RFO3	Other-RLK	Aha	Araha.3018s0001.3	63.687	179	515	693	3.16E-76	Other-RLK	same
At_RPP5	TNL	Aha	Araha.18217s0002.1	82.781	151	10	160	1.68E-76	TN	different
At_RPP1	TNL	Aha	Araha.66827s0001.1	65.341	176	69	241	1.23E-76	TX	different
At_RPP2a	TNL	Aha	Araha.18217s0002.1	64.249	193	231	423	9.10E-77	TN	different
At_RFO3	Other-RLK	Aha	Araha.3018s0001.2	63.687	179	515	693	7.30E-77	Other-RLK	same
At_RLM1a	TNL	Aha	Araha.25586s0003.1	77.852	149	10	158	2.84E-79	TX	different
At_RLM1b	TNL	Aha	Araha.38723s0001.1	66.5	200	1	200	1.97E-80	TX	different
At_WRR4b	TNL	Aha	Araha.25586s0003.1	81.132	159	1	159	2.23E-81	TX	different
At_WRR4b	TNL	Aha	Araha.38723s0001.1	63.981	211	1	206	2.07E-81	TX	different
At_RLM1a	TNL	Aha	Araha.38723s0001.1	66.162	198	3	200	1.41E-81	TX	different
At_RPP8	CNL	Aha	Araha.31961s0001.1	79.769	173	1	173	2.95E-84	CNL	same
Bol_FocBo1	TNL	Aha	Araha.18217s0002.1	60.086	233	229	454	4.53E-93	TN	different
At_RPP4	TNL	Aha	Araha.18217s0002.1	74.672	229	232	453	7.89E-109	TN	different
At_RPP5	TNL	Aha	Araha.18217s0002.1	75.904	249	236	477	4.58E-116	TN	different
At_RLP42	LRR-RLP	Aha	Araha.5796s0001.1	67.774	301	588	880	1.19E-128	LRR-RLP	same
Bra_Crr1a	TNL	Aha	Araha.4751s0001.1	61.19	353	69	421	6.29E-131	TN	different
At_RPP7	NL	Aha	Araha.10133s0001.1	61.818	330	1	329	2.72E-132	NBS	different
At_RLM1b	TNL	Aha	Araha.30867s0001.1	61.69	355	1	355	7.00E-153	TN	different
At_RLP23	LRR-RLP	Aha	Araha.5796s0001.1	76.898	303	588	890	9.42E-155	LRR-RLP	same
Bju_WRR1	CNL	Aha	Araha.20058s0003.1	64.402	368	66	427	3.52E-156	NBS	different
At_NGR1b	RNL	Aha	Araha.11408s0002.1	62.35	417	406	815	3.05E-157	NL	different
At_NGR1a	RNL	Aha	Araha.11408s0002.1	65.942	414	404	809	1.86E-170	NL	different
At_RLM1a	TNL	Aha	Araha.30867s0001.1	67.787	357	1	357	2.65E-172	TN	different
At_SOBIR1	LRR-RLK	Aha	Araha.3596s0002.1	95.327	642	1	641	0	LRR-RLK	same
At_RPS5	TNL	Aha	Araha.0650s0020.1	94.831	890	1	889	0	CNL	different
At_RPS4	TNL	Aha	Araha.1947s0003.1	94.084	1217	1	1217	0	TNL	same
At_RLP30	LRR-RLP	Aha	Araha.61250s0003.1	92.875	786	1	786	0	LRR-RLP	same
At_WRR12	TNL	Aha	Araha.15627s0017.1	92.718	1030	1	1030	0	TNL	same
At_RPS2	NL	Aha	Araha.4285s0004.1	92.426	911	1	909	0	NL	same
At_RPM1	NL	Aha	Araha.32978s0005.1	91.733	883	44	926	0	NL	same
At_RLP32	LRR-RLP	Aha	Araha.5514s0006.1	91.654	683	183	865	0	LRR-RLP	same
At_ADR1	NL	Aha	Araha.3012s0003.1	91.624	788	1	787	0	NL	same
At_FLS2	LRR-RLK	Aha	Araha.10139s0013.1	91.489	1175	1	1173	0	LRR-RLK	same
At_RPS5	TNL	Aha	Araha.0650s0020.3	91.011	890	1	889	0	CNL	different
At_RPS5	TNL	Aha	Araha.0650s0020.2	91.011	890	1	889	0	CNL	different
At_RLP1	LRR-RLP	Aha	Araha.23444s0004.1	90.278	1080	1	1077	0	LRR-RLP	same
At_RFO1	Other-RLK	Aha	Araha.29413s0005.1	89.687	766	1	751	0	Other-RLK	same
At_NGR1a	RNL	Aha	Araha.11408s0003.1	89.312	814	1	809	0	CNL	different
At_RRS1	TNL	Aha	Araha.1947s0004.1	88.444	1298	1	1286	0	TNL	same
At_BAK1	LRR-RLK	Aha	Araha.20697s0001.1	83.752	597	22	615	0	LRR-RLK	same
At_RLP42	LRR-RLP	Aha	Araha.7647s0001.1	83.418	591	291	880	0	LRR-RLP	same
At_RPP8	CNL	Aha	Araha.31961s0001.1	80.37	433	340	772	0	CNL	same
At_RFO3	Other-RLK	Aha	Araha.18229s0005.1	79.813	857	14	850	0	Other-RLK	same
At_RPP13	CNL	Aha	Araha.6729s0012.1	79.103	847	1	834	0	CNL	same
At_BAK1	LRR-RLK	Aha	Araha.24929s0001.1	78.986	414	23	376	0	LRR-RLK	same
At_RPP2b	TNL	Aha	Araha.8600s0002.2	78.821	831	381	1207	0	NL	different
At_RLP32	LRR-RLP	Aha	Araha.24867s0001.1	77.324	441	415	854	0	LRR-RLP	same
At_RLM1a	TNL	Aha	Araha.3236s0003.1	77.247	712	4	715	0	TNL	same
At_RPP2a	TNL	Aha	Araha.8600s0003.1	76.752	856	409	1257	0	TNL	same
At_WRR4a	TNL	Aha	Araha.30867s0001.1	76.554	354	1	353	0	TN	different
At_NGR1b	RNL	Aha	Araha.11408s0003.1	76.415	636	5	633	0	CNL	different
At_BAK1	LRR-RLK	Aha	Araha.5645s0001.1	75.929	619	9	615	0	LRR-RLK	same

At_RPP2b	TNL	Aha	Araha.8600s0002.1	75.495	1212	1	1207	0	TNL	same
At_RLM3	TN	Aha	Araha.16675s0001.1	74.327	483	159	605	0	NBS	different
At_WRR4b	TNL	Aha	Araha.30867s0001.1	74.221	353	3	355	0	TN	different
At_RFO2	LRR-RLP	Aha	Araha.2722s0007.1	72.849	744	6	749	0	LRR-RLP	same
At_RFO3	Other-RLK	Aha	Araha.11090s0001.1	72	725	134	850	0	Other-RLK	same
At_RPP8	CNL	Aha	Araha.11242s0013.2	71.944	720	193	908	0	NL	different
At_RLP23	LRR-RLP	Aha	Araha.7647s0001.1	71.215	601	290	890	0	LRR-RLP	same
At_RPP8	CNL	Aha	Araha.11242s0013.1	71.131	911	1	907	0	CNL	same
At_NGR1b	RNL	Aha	Araha.11408s0003.1	71.011	821	5	815	0	CNL	different
Bna_Rlm9/4/7	Other-RLK	Aha	Araha.15691s0004.1	70.274	804	1	794	0	Other-RLK	same
Bna_LepR3/Rlm2	LRR-RLP	Aha	Araha.24867s0001.1	69.248	452	502	948	0	LRR-RLP	same
At_RLM1b	TNL	Aha	Araha.15145s0001.1	69.194	844	66	901	0	TNL	same
Bna_Rlm9/4/7	Other-RLK	Aha	Araha.29413s0004.1	68.863	774	24	794	0	Other-RLK	same
At_RFO1	Other-RLK	Aha	Araha.1482s0001.1	68.62	529	210	733	0	Other-RLK	same
At_RPS4	TNL	Aha	Araha.2395s0003.2	68.189	635	198	821	0	NL	different
At_RLM1b	TNL	Aha	Araha.3236s0003.1	68.061	717	5	721	0	TNL	same
At_RLP1	LRR-RLP	Aha	Araha.23444s0005.1	66.954	1044	44	1077	0	LRR-RLP	same
At_RPP8	CNL	Aha	Araha.3152s0005.2	66.557	909	1	904	0	CNL	same
At_RPP8	CNL	Aha	Araha.3152s0005.1	66.557	909	1	904	0	CNL	same
At_NGR1a	RNL	Aha	Araha.11408s0003.1	66.553	589	1	573	0	CNL	different
Bna_Rlm9/4/7	Other-RLK	Aha	Araha.29413s0003.1	66.294	537	263	793	0	Other-RLK	same
At_RLP1	LRR-RLP	Aha	Araha.10536s0003.1	66.262	824	249	1065	0	LRR-RLP	same
At_RPS5	TNL	Aha	Araha.1122s0007.1	66.142	889	1	883	0	NL	different
At_RPP8	CNL	Aha	Araha.3450s0004.1	66.083	914	1	908	0	NL	different
At_RPP8	CNL	Aha	Araha.3450s0004.2	66.083	914	1	908	0	NL	different
At_RPS5	TNL	Aha	Araha.0650s0013.1	65.607	881	1	880	0	CNL	different
Bna_LepR3/Rlm2	LRR-RLP	Aha	Araha.34729s0003.1	65.586	956	1	949	0	LRR-RLP	same
At_RFO1	Other-RLK	Aha	Araha.53748s0001.1	65.546	476	232	704	0	Other-RLK	same
At_WRR4b	TNL	Aha	Araha.3236s0003.1	65.229	719	1	718	0	TNL	same
At_RPP7	NL	Aha	Araha.25006s0001.1	65.152	726	150	866	0	NL	same
At_RLP23	LRR-RLP	Aha	Araha.20926s0004.1	64.993	717	175	889	0	LRR-RLP	same
At_RLP32	LRR-RLP	Aha	Araha.12216s0001.1	64.892	883	29	850	0	LRR-RLP	same
At_RPS4	TNL	Aha	Araha.2395s0003.1	64.601	839	4	821	0	TNL	same
At_RFO1	Other-RLK	Aha	Araha.1482s0002.1	64.481	732	10	718	0	Other-RLK	same
At_RLP23	LRR-RLP	Aha	Araha.4280s0013.1	64.426	714	175	886	0	LRR-RLP	same
At_RPP7	NL	Aha	Araha.13235s0001.1	64.133	750	126	869	0	NL	same
At_RLM1a	TNL	Aha	Araha.12875s0001.1	64.036	1001	1	988	0	TNL	same
At_WRR9	NL	Aha	Araha.3236s0003.1	64.017	717	5	720	0	TNL	different
At_RLM1a	TNL	Aha	Araha.15145s0001.1	64.005	839	66	898	0	TNL	same
Bna_LepR3/Rlm2	LRR-RLP	Aha	Araha.5514s0006.1	63.942	685	275	950	0	LRR-RLP	same
At_RPP13	CNL	Aha	Araha.21372s0011.1	63.94	868	1	830	0	CNL	same
At_RPS5	TNL	Aha	Araha.0650s0021.1	63.636	891	1	885	0	CNL	different
At_RLP42	LRR-RLP	Aha	Araha.4280s0013.1	63.61	698	182	875	0	LRR-RLP	same
At_WRR8	TNL	Aha	Araha.28366s0002.1	63.425	1203	20	1137	0	TNL	same
At_WRR9	NL	Aha	Araha.0550s0005.1	63.352	1086	16	1095	0	TNL	different
At_RLM1b	TNL	Aha	Araha.0550s0005.1	63.284	1005	10	997	0	TNL	same
At_RPP8	CNL	Aha	Araha.20058s0003.1	63.274	452	66	516	0	NBS	different
At_RLM1a	TNL	Aha	Araha.34654s0002.1	62.791	817	187	997	0	NL	different
At_RLP30	LRR-RLP	Aha	Araha.5378s0001.1	62.768	513	276	786	0	LRR-RLP	same
At_WRR8	TNL	Aha	Araha.8200s0005.1	62.737	738	17	745	0	TNL	same
At_RAC1	TNL	Aha	Araha.28366s0002.1	62.727	1210	9	1156	0	TNL	same
Bna_Rlm9/4/7	Other-RLK	Aha	Araha.2578s0001.1	62.516	779	22	793	0	Other-RLK	same
At_RLP42	LRR-RLP	Aha	Araha.20926s0004.1	62.256	718	182	889	0	LRR-RLP	same
At_WRR12	TNL	Aha	Araha.16807s0001.1	61.858	1012	18	1021	0	TNL	same
At_RLP30	LRR-RLP	Aha	Araha.12235s0001.1	61.779	798	1	779	0	LRR-RLP	same
At_WRR8	TNL	Aha	Araha.5427s0001.1	61.722	755	12	757	0	TNL	same
At_RAC1	TNL	Aha	Araha.8200s0005.1	61.673	741	6	735	0	TNL	same
At_RLP32	LRR-RLP	Aha	Araha.34729s0003.1	61.255	924	1	854	0	LRR-RLP	same
At_RAC1	TNL	Aha	Araha.5427s0001.1	61.141	754	1	747	0	TNL	same
At_RLM1a	TNL	Aha	Araha.15917s0002.1	60.965	912	5	914	0	TNL	same
At_RLM1b	TNL	Aha	Araha.12875s0001.1	60.905	995	1	979	0	TNL	same
At_RLP30	LRR-RLP	Aha	Araha.28473s0001.1	60.653	521	255	774	0	LRR-RLP	same
At_WRR9	NL	Aha	Araha.15145s0001.1	60.373	858	71	920	0	TNL	different
Bju_WRR1	CNL	Aha	Araha.11242s0013.2	60.362	719	188	900	0	NL	different
Bju_WRR1	CNL	Aha	Araha.11242s0013.1	60.044	911	1	900	0	CNL	same
At_RPP7	NL	Aha	Araha.44858s0001.1	60.035	568	112	677	0	NL	same
At_RPP1	TNL	Aly	AL2G28690.t1	62.581	155	98	252	2.31E-58	TX	different
Bra_Crr1a	TNL	Aly	AL2G28690.t1	73.718	156	69	224	6.49E-71	TX	different
At_RPP1	TNL	Aly	AL4G22240.t1	61.751	217	89	305	1.16E-79	TNL	same
At_RLM1b	TNL	Aly	AL4G22240.t1	64.423	208	13	220	1.55E-80	TNL	same
At_WRR9	NL	Aly	AL1G66180.t1	60.776	232	168	398	7.05E-81	NL	same
At_RLM1b	TNL	Aly	AL2G11220.t1	73.404	188	1	188	3.01E-83	TNL	same
At_RPP1	TNL	Aly	AL786U10010.t1	63.959	197	312	508	2.03E-83	NBS	different
At_WRR4a	TNL	Aly	AL2G11220.t1	73.404	188	9	196	3.96E-87	TNL	same
At_RLM1b	TNL	Aly	AL7G41850.t1	63.137	255	76	329	5.15E-88	Other-NLR	different
At_WRR4b	TNL	Aly	AL7G41850.t1	64.706	255	77	329	1.11E-89	Other-NLR	different
At_RLM1b	TNL	Aly	AL1G66180.t1	60.643	249	147	395	1.15E-90	NL	different
At_RLM1a	TNL	Aly	AL7G41850.t1	65.098	255	76	329	7.78E-91	Other-NLR	different
At_RLM1a	TNL	Aly	AL1G66180.t1	63.948	233	162	394	5.96E-91	NL	different
At_RLP30	LRR-RLP	Aly	AL4G14280.t1	70.936	203	585	786	2.68E-92	LRR-RLP	same
At_WRR4b	TNL	Aly	AL2G11220.t1	83.146	178	11	188	3.78E-95	TNL	same
At_WRR4a	TNL	Aly	AL1G66180.t1	62.595	262	150	408	3.18E-97	NL	different
At_RLM1a	TNL	Aly	AL2G11220.t1	84.574	188	1	188	2.71E-97	TNL	same
At_RRS1	TNL	Aly	AL8G13710.t1	64	275	896	1169	2.88E-103	TX	different
At_RPP8	CNL	Aly	AL625U10020.t1	73.894	226	1	226	4.82E-107	CN	different
At_WRR4b	TNL	Aly	AL1G66180.t1	71.315	251	163	410	1.06E-109	NL	different

At_RPP1	TNL	Aly	AL20U10050.t1	65.568	273	96	368	5.36E-116	TNL	same
At_RAC1	TNL	Aly	AL8G18480.t1	65.033	306	12	314	1.99E-116	TN	different
At_RPS4	TNL	Aly	AL8G13590.t1	94.527	201	1	201	1.36E-117	Other-NLR	different
At_WRR8	TNL	Aly	AL8G18480.t1	64.262	305	23	324	6.97E-120	TN	different
At_RFO3	Other-RLK	Aly	AL3G28680.t1	83.478	230	19	238	1.51E-123	Other-RLK	same
Bra_cRa/cRb	TNL	Aly	AL20U10050.t1	61.095	347	1	345	1.03E-124	TNL	same
Bra_Crr1a	TNL	Aly	AL20U10050.t1	72.161	273	68	340	9.86E-128	TNL	same
At_WRR4a	TNL	Aly	AL2G23700.t1	60.302	398	43	436	5.52E-147	TN	different
At_RAC1	TNL	Aly	AL8G11900.t1	61.253	431	3	430	1.09E-147	Other-NLR	different
At_RLM1b	TNL	Aly	AL2G23700.t1	64.722	360	44	401	2.61E-148	TN	different
At_WRR8	TNL	Aly	AL8G11900.t1	60.93	430	14	438	2.14E-151	Other-NLR	different
At_RPP7	NL	Aly	AL1G65650.t1	65.698	344	1	343	9.08E-153	NBS	different
At_RLM1a	TNL	Aly	AL1G65640.t1	62.67	442	1	440	8.09E-163	TNL	same
Bra_Crr1a	TNL	Aly	AL5G16480.t1	61.675	394	67	460	3.66E-166	TN	different
At_RPP2a	TNL	Aly	AL7G37760.t1	64.286	406	9	414	1.31E-168	Other-NLR	different
At_NGR1b	RNL	Aly	AL8G44510.t1	63.549	417	406	815	4.32E-170	NL	different
Bra_cRa/cRb	TNL	Aly	AL7G14900.t1	63.326	439	65	503	1.26E-174	TNL	same
At_WRR4b	TNL	Aly	AL2G23700.t1	67.254	397	45	439	5.35E-175	TN	different
At_BAK1	LRR-RLK	Aly	AL7G18140.t1	99.35	615	1	615	0	LRR-RLK	same
At_RPS5	TNL	Aly	AL1G23500.t1	95.52	692	122	812	0	NL	different
At_SOBIR1	LRR-RLK	Aly	AL4G27170.t1	94.548	642	1	641	0	LRR-RLK	same
At_RPS2	NL	Aly	AL7G26890.t1	92.865	911	1	909	0	NL	same
At_RLP30	LRR-RLP	Aly	AL3G15860.t1	92.494	786	1	786	0	LRR-RLP	same
At_ADR1	NL	Aly	AL1G47950.t1	92.259	788	1	787	0	NL	same
At_RLP1	LRR-RLP	Aly	AL1G17550.t1	91.736	1077	1	1077	0	LRR-RLP	same
At_RPM1	NL	Aly	AL3G18260.t1	91.037	926	1	926	0	NL	same
At_NGR1a	RNL	Aly	AL8G44500.t1	89.312	814	1	809	0	CNL	different
At_RRS1	TNL	Aly	AL8G13580.t1	87.318	1238	1	1228	0	TNL	same
At_RPS4	TNL	Aly	AL8G13590.t1	86.268	619	284	902	0	Other-NLR	different
At_FLS2	LRR-RLK	Aly	AL8G12090.t1	85.532	1175	1	1173	0	LRR-RLK	same
At_WRR4b	TNL	Aly	AL1G66350.t2	85.271	774	11	764	0	TNL	same
At_RPP8	CNL	Aly	AL293U10010.t1	85.201	473	1	473	0	CN	different
At_WRR12	TNL	Aly	AL1G29920.t1	85.181	992	1	992	0	TNL	same
At_RPP8	CNL	Aly	AL43U10010.t1	84.052	696	1	696	0	CNL	same
At_RLP42	LRR-RLP	Aly	AL3G40650.t1	83.565	864	18	880	0	LRR-RLP	same
At_RFO1	Other-RLK	Aly	AL2G40360.t1	83.438	477	230	705	0	Other-RLK	same
At_WRR4b	TNL	Aly	AL1G66350.t1	82.558	774	11	764	0	TNL	same
At_BAK1	LRR-RLK	Aly	AL3G45570.t1	82.392	602	17	615	0	LRR-RLK	same
At_RLP32	LRR-RLP	Aly	AL3G16300.t1	79.299	628	1	625	0	LRR-RLP	same
At_RPP8	CNL	Aly	AL8G21640.t1	78.611	907	1	907	0	CNL	same
At_RLP42	LRR-RLP	Aly	AL3G40730.t1	78.523	880	1	879	0	LRR-RLP	same
At_WRR8	TNL	Aly	AL8G12190.t1	78.343	1159	12	1139	0	TNL	same
At_BAK1	LRR-RLK	Aly	AL2G31410.t1	77.424	629	1	615	0	LRR-RLK	same
At_BAK1	LRR-RLK	Aly	AL3G45640.t1	77.393	606	10	615	0	LRR-RLK	same
At_RPP2b	TNL	Aly	AL7G34610.t1	76.073	1212	1	1207	0	TNL	same
At_RPP4	TNL	Aly	AL7G37760.t1	75.896	1116	1	1115	0	Other-NLR	different
At_BAK1	LRR-RLK	Aly	AL1G48710.t1	75.654	612	16	615	0	LRR-RLK	same
At_RFO3	Other-RLK	Aly	AL3G28680.t1	74.806	516	339	850	0	Other-RLK	same
At_RPP5	TNL	Aly	AL7G37760.t1	74.673	1070	10	1075	0	Other-NLR	different
At_RAC1	TNL	Aly	AL8G12190.t1	74.144	1168	1	1156	0	TNL	same
At_RPP2a	TNL	Aly	AL2G17260.t1	73.732	769	533	1300	0	TNL	same
Bna_Rlm9/4/7	Other-RLK	Aly	AL3G52440.t1	72.587	777	24	793	0	Other-RLK	same
Bna_Rlm9/4/7	Other-RLK	Aly	AL3G52410.t1	72.587	777	24	793	0	Other-RLK	same
At_WRR8	TNL	Aly	AL8G11920.t1	72.333	1153	12	1136	0	TNL	same
At_RPP2a	TNL	Aly	AL7G34620.t1	71.899	911	409	1299	0	TNL	same
At_RLP23	LRR-RLP	Aly	AL4G16860.t1	71.689	876	17	888	0	LRR-RLP	same
At_NGR1b	RNL	Aly	AL8G44500.t1	71.62	821	5	815	0	CNL	different
At_RRS1	TNL	Aly	AL8G13740.t1	71.246	1019	1	1005	0	TNL	same
At_RPP8	CNL	Aly	AL1G61840.t2	71.116	914	1	908	0	CNL	same
At_RPS4	TNL	Aly	AL8G13750.t1	71.085	1207	1	1192	0	TNL	same
At_RPP7	NL	Aly	AL2G17650.t1	71.034	870	1	866	0	NL	same
At_RFO2	LRR-RLP	Aly	AL1G29490.t1	70.963	737	4	740	0	LRR-RLP	same
At_RPP13	CNL	Aly	AL5G25700.t1	70.651	845	1	830	0	NL	different
At_RLM1a	TNL	Aly	AL3G49580.t1	70.523	994	4	991	0	TNL	same
At_RPP39	CNL	Aly	AL2G15160.t1	70.472	911	1	878	0	CNL	same
At_RLP42	LRR-RLP	Aly	AL4G16860.t1	70.208	866	18	876	0	LRR-RLP	same
At_RPP8	CNL	Aly	AL1G21900.t1	69.737	912	1	908	0	CNL	same
At_RAC1	TNL	Aly	AL8G11920.t1	69.633	1225	1	1160	0	TNL	same
At_RFO1	Other-RLK	Aly	AL1G28270.t1	69.558	588	115	694	0	Other-RLK	same
At_RLP23	LRR-RLP	Aly	AL3G40650.t1	69.143	875	17	890	0	LRR-RLP	same
At_RFO2	LRR-RLP	Aly	AL2G31980.t1	69.083	731	20	750	0	LRR-RLK	different
At_RRS1	TNL	Aly	AL8G13770.t1	68.985	532	8	536	0	NBS	different
At_NGR1b	RNL	Aly	AL8G44490.t1	68.675	747	64	806	0	NL	different
At_RPP1	TNL	Aly	AL1G67230.t1	68.41	459	94	551	0	TN	different
At_RPP8	CNL	Aly	AL1G61840.t1	68.162	914	1	908	0	CNL	same
At_RFO3	Other-RLK	Aly	AL2G26120.t1	68.054	817	23	823	0	Other-RLK	same
At_RLP32	LRR-RLP	Aly	AL3G22620.t2	67.807	497	360	854	0	LRR-RLP	same
At_WRR8	TNL	Aly	AL8G11950.t1	67.681	526	16	535	0	TN	different
At_RLM1b	TNL	Aly	AL2G11260.t1	67.659	1008	1	997	0	Other-NLR	different
Bna_Rlm9/4/7	Other-RLK	Aly	AL2G40380.t1	67.253	455	263	709	0	Other-RLK	same
At_RAC1	TNL	Aly	AL8G11950.t1	67.17	530	1	523	0	TN	different
At_RPP13	CNL	Aly	AL5G25710.t1	67.012	773	1	743	0	CNL	same
At_RPS4	TNL	Aly	AL8G13700.t1	66.838	1164	7	1165	0	Other-NLR	different
At_NGR1a	RNL	Aly	AL8G44510.t1	66.667	414	404	809	0	NL	different
At_WRR4a	TNL	Aly	AL1G66350.t2	66.372	791	1	770	0	TNL	same
At_RLP1	LRR-RLP	Aly	AL1G17540.t2	66.208	1089	1	1077	0	LRR-RLP	same

At_RFO1	Other-RLK	Aly	AL1G28270.t1	66.141	697	27	704	0	Other-RLK	same
At_RPS5	TNL	Aly	AL1G23480.t1	66.105	891	1	885	0	CNL	different
At_RLP1	LRR-RLP	Aly	AL1G17310.t1	66.019	824	249	1065	0	LRR-RLP	same
At_RPS4	TNL	Aly	AL8G14360.t1	65.998	1147	11	1133	0	TNL	same
At_RLP1	LRR-RLP	Aly	AL1G17540.t1	65.932	1089	1	1077	0	LRR-RLP	same
At_RLP23	LRR-RLP	Aly	AL4G28610.t1	65.915	710	184	888	0	LRR-RLP	same
Bna_Rlm9/4/7	Other-RLK	Aly	AL2G28900.t1	65.863	788	28	794	0	Other-RLK	same
Bju_WRR1	CNL	Aly	AL293U10010.t1	65.828	477	1	469	0	CN	different
Bra_Crr1a	TNL	Aly	AL1G67230.t1	65.795	459	66	524	0	TN	different
Bra_Crr1a	TNL	Aly	AL7G31750.t1	65.714	525	66	590	0	TN	different
At_RLP23	LRR-RLP	Aly	AL3G40730.t1	65.657	891	1	890	0	LRR-RLP	same
At_RRS1	TNL	Aly	AL8G14320.t1	65.559	752	6	704	0	TNL	same
At_RLM1b	TNL	Aly	AL2G11240.t1	65.554	1019	1	1004	0	TNL	same
At_RPP8	CNL	Aly	AL7G39090.t1	65.265	904	1	899	0	CNL	same
At_RPS4	TNL	Aly	AL8G14240.t1	65.259	1160	4	1139	0	TNL	same
At_RFO1	Other-RLK	Aly	AL1G28280.t1	65.132	760	6	751	0	Other-RLK	same
At_RLP23	LRR-RLP	Aly	AL4G28600.t1	64.854	717	175	889	0	LRR-RLP	same
At_RFO1	Other-RLK	Aly	AL1G28290.t1	64.674	736	19	738	0	Other-RLK	same
At_RPP7	NL	Aly	AL2G17830.t1	64.612	876	1	866	0	NL	same
At_WRR4a	TNL	Aly	AL1G66350.t1	64.475	791	1	770	0	TNL	same
At_WRR4b	TNL	Aly	AL1G66260.t1	64.158	1063	1	1058	0	TNL	same
At_RLP32	LRR-RLP	Aly	AL3G16310.t1	64.129	867	28	868	0	LRR-RLP	same
Bra_Crr1a	TNL	Aly	AL8G17970.t1	64.085	749	68	807	0	TNL	same
At_RLM1a	TNL	Aly	AL2G14410.t1	63.964	999	5	997	0	TNL	same
Bna_Rlm9/4/7	Other-RLK	Aly	AL2G40360.t1	63.915	424	325	737	0	Other-RLK	same
At_RPS4	TNL	Aly	AL8G13790.t1	63.879	1160	7	1151	0	TNL	same
At_RLP42	LRR-RLP	Aly	AL4G28610.t1	63.585	714	184	889	0	LRR-RLP	same
At_RPP1	TNL	Aly	AL4G22780.t1	63.333	450	93	542	0	TN	different
At_RRS1	TNL	Aly	AL8G13620.t1	63.286	1272	11	1169	0	NL	different
Bju_WRR1	CNL	Aly	AL1G61840.t2	63.256	909	1	900	0	CNL	same
At_RPS5	TNL	Aly	AL7G41510.t1	63.105	889	1	883	0	CNL	different
At_RLP32	LRR-RLP	Aly	AL3G46870.t1	62.899	752	110	845	0	LRR-RLP	same
Bra_Crr1a	TNL	Aly	AL7G14900.t1	62.869	474	57	530	0	TNL	same
Bra_Crr1a	TNL	Aly	AL4G22780.t1	62.857	455	64	518	0	TN	different
Bju_WRR1	CNL	Aly	AL43U10010.t1	62.837	705	1	695	0	CNL	same
At_WRR4a	TNL	Aly	AL1G66260.t1	62.563	999	1	998	0	TNL	same
At_RPS5	TNL	Aly	AL1G23590.t1	62.543	881	1	880	0	CNL	different
At_RLP42	LRR-RLP	Aly	AL4G28600.t1	62.535	718	182	889	0	LRR-RLP	same
At_ADR1	NL	Aly	AL6G14380.t1	62.408	814	6	787	0	NL	same
At_RPP7	NL	Aly	AL2G17810.t1	62.372	877	1	869	0	NL	same
At_RLP42	LRR-RLP	Aly	AL7G37050.t1	62.212	868	18	876	0	LRR-RLP	same
Bna_LepR3/Rlm2	LRR-RLP	Aly	AL3G16310.t1	61.783	785	176	950	0	LRR-RLP	same
At_RLM1a	TNL	Aly	AL1G66330.t1	61.705	833	10	839	0	TNL	same
At_RLM1a	TNL	Aly	AL1G66350.t2	61.32	773	10	759	0	TNL	same
At_RLM1a	TNL	Aly	AL2G11240.t1	61.184	997	3	986	0	TNL	same
Bna_LepR3/Rlm2	LRR-RLP	Aly	AL3G22620.t2	61.1	509	442	948	0	LRR-RLP	same
Bju_WRR1	CNL	Aly	AL1G61840.t1	60.946	909	1	900	0	CNL	same
At_NGR1a	RNL	Aly	AL8G44490.t1	60.858	746	68	800	0	NL	different
At_RRS1	TNL	Aly	AL8G14230.t1	60.766	1305	8	1288	0	NL	different
At_RLM1b	TNL	Aly	AL3G49580.t1	60.685	992	5	985	0	TNL	same
Bra_cRa/cRb	TNL	Aly	AL1G67230.t1	60.667	450	73	522	0	TN	different
At_RPP7	NL	Aly	AL1G65670.t1	60.64	531	349	866	0	NL	same
At_RLP23	LRR-RLP	Aly	AL7G37050.t1	60.523	879	10	887	0	LRR-RLP	same
At_WRR12	TNL	Aly	AL7G49910.t1	60.419	907	12	915	0	TNL	same
At_WRR8	TNL	Aly	AL2G19370.t1	60.218	734	19	745	0	TNL	same
At_RLM1a	TNL	Aly	AL2G11260.t1	60.18	1002	1	997	0	Other-NLR	different
Bna_Rlm9/4/7	Other-RLK	Bvu	maker-Contig382-snap-gene-0.50-mRNA-1	70.886	158	567	724	6.86E-59	Other-RLK	same
At_RFO1	Other-RLK	Bvu	maker-Contig382-snap-gene-0.50-mRNA-1	73.856	153	534	686	8.02E-64	Other-RLK	same
At_WRR8	TNL	Bvu	maker-Contig2985-snap-gene-0.2-mRNA-1	63.333	210	275	482	4.54E-66	Other-NLR	different
At_RLP1	LRR-RLP	Bvu	snap_masked-Contig7566-processed-gene-0.0-mRNA	68.263	167	898	1064	3.05E-69	LRR-RLP	same
At_WRR9	NL	Bvu	maker-Contig97-snap-gene-0.50-mRNA-1	61.058	208	17	224	2.70E-73	TNL	different
At_RFO3	Other-RLK	Bvu	maker-Contig412-snap-gene-0.33-mRNA-1	63.243	185	515	699	3.77E-77	Other-RLK	same
At_RPP1	TNL	Bvu	maker-Contig97-snap-gene-0.50-mRNA-1	61.538	208	97	304	2.04E-77	TNL	same
At_RLM1b	TNL	Bvu	maker-Contig97-snap-gene-0.50-mRNA-1	65.7	207	13	219	1.18E-79	TNL	same
At_WRR4a	TNL	Bvu	maker-Contig97-snap-gene-0.50-mRNA-1	63.084	214	12	225	6.74E-80	TNL	same
At_RPP8	CNL	Bvu	snap_masked-Contig685-processed-gene-0.6-mRNA-	67.308	208	71	278	8.61E-86	NBS	different
At_WRR4b	TNL	Bvu	maker-Contig97-snap-gene-0.50-mRNA-1	64.706	221	14	234	9.48E-87	TNL	same
At_RLM1a	TNL	Bvu	maker-Contig97-snap-gene-0.50-mRNA-1	61.506	239	13	251	5.95E-88	TNL	same
Bra_Crr1a	TNL	Bvu	maker-Contig907-snap-gene-0.24-mRNA-1	64.655	232	40	271	4.09E-88	TX	different
At_RLP1	LRR-RLP	Bvu	maker-Contig188-snap-gene-0.8-mRNA-1	61.798	267	809	1071	4.07E-93	LRR-RLP	same
At_RPP1	TNL	Bvu	maker-Contig907-snap-gene-0.24-mRNA-1	69.369	222	78	299	8.71E-97	TX	different
Bju_WRR1	CNL	Bvu	snap_masked-Contig3090-processed-gene-0.4-mRNA	65.587	247	12	253	1.01E-101	CN	different
At_RPP8	CNL	Bvu	maker-Contig6075-snap-gene-0.2-mRNA-1	72.065	247	662	908	4.41E-102	CNL	same
At_RLM3	TN	Bvu	maker-Contig989-snap-gene-0.30-mRNA-1	65.351	228	9	235	2.02E-102	TX	different
At_WRR4a	TNL	Bvu	maker-Contig1030-snap-gene-0.15-mRNA-1	64.662	266	1	266	6.76E-103	TX	different
At_RPP4	TNL	Bvu	maker-Contig989-snap-gene-0.30-mRNA-1	69.697	231	11	240	1.12E-107	TX	different
At_RPP2a	TNL	Bvu	maker-Contig989-snap-gene-0.30-mRNA-1	70	230	9	237	1.04E-109	TX	different
At_RPP5	TNL	Bvu	maker-Contig989-snap-gene-0.30-mRNA-1	71.368	234	10	242	6.44E-110	TX	different
At_RLM1b	TNL	Bvu	maker-Contig1030-snap-gene-0.15-mRNA-1	67.857	252	16	267	6.30E-110	TX	different
At_RLM1a	TNL	Bvu	maker-Contig1030-snap-gene-0.15-mRNA-1	68.539	267	1	267	4.20E-115	TX	different
At_WRR4b	TNL	Bvu	maker-Contig1030-snap-gene-0.15-mRNA-1	68.657	268	1	268	4.07E-116	TX	different
At_RLP1	LRR-RLP	Bvu	maker-Contig173-snap-gene-0.36-mRNA-1	67.681	263	809	1071	4.64E-118	LRR-RLP	same
Bra_Crr1a	TNL	Bvu	maker-Contig6921-snap-gene-0.4-mRNA-1	64.516	279	93	371	5.50E-121	TN	different
Bol_FocBo1	TNL	Bvu	maker-Contig989-snap-gene-0.30-mRNA-1	74.236	229	9	237	7.55E-122	TX	different
At_RPP8	CNL	Bvu	snap_masked-Contig3090-processed-gene-0.4-mRNA	77.778	252	12	259	9.86E-131	CN	different
At_RPP4	TNL	Bvu	maker-Contig989-snap-gene-0.34-mRNA-1	60.959	438	676	1106	2.31E-142	NL	different

At_RPP4	TNL	Bvu	maker-Contig989-snap-gene-0.32-mRNA-1	61.017	413	688	1092	4.11E-144	TNL	same
At_RPP1	TNL	Bvu	maker-Contig6921-snap-gene-0.4-mRNA-1	74.912	283	118	400	1.90E-145	TN	different
At_WRR8	TNL	Bvu	maker-Contig6657-snap-gene-0.5-mRNA-1	62.57	358	14	354	3.25E-149	TX	different
At_RAC1	TNL	Bvu	maker-Contig6657-snap-gene-0.5-mRNA-1	63.966	358	3	344	3.44E-150	TX	different
At_RPP1	TNL	Bvu	maker-Contig5799-snap-gene-0.3-mRNA-1	61.787	403	96	490	9.28E-157	TN	different
At_RLM1a	TNL	Bvu	maker-Contig178-snap-gene-0.36-mRNA-1	62.085	422	14	433	5.58E-158	Other-NLR	different
At_RAC1	TNL	Bvu	maker-Contig1769-snap-gene-0.9-mRNA-1	61.353	414	330	735	8.49E-159	NL	different
Bra_Crr1a	TNL	Bvu	snap_masked-Contig414-processed-gene-0.17-mRNA	60.396	404	67	460	8.75E-160	TN	different
At_NGR1b	RNL	Bvu	maker-Contig374-snap-gene-0.22-mRNA-1	61.259	413	406	812	5.63E-161	CNL	different
At_RLM3	TN	Bvu	maker-Contig989-snap-gene-0.32-mRNA-1	61.374	422	9	422	7.97E-163	TNL	different
Bna_Rlm9/4/7	Other-RLK	Bvu	maker-Contig85-snap-gene-1.57-mRNA-1	69.274	358	381	737	4.21E-165	Other-RLK	same
At_RFO1	Other-RLK	Bvu	maker-Contig85-snap-gene-1.57-mRNA-1	64.416	385	348	731	7.31E-167	Other-RLK	same
At_RPP1	TNL	Bvu	maker-Contig900-snap-gene-0.15-mRNA-1	64.097	454	715	1163	1.67E-168	NL	different
At_RPP2a	TNL	Bvu	maker-Contig715-snap-gene-0.36-mRNA-1	66.422	408	7	414	2.21E-171	TNL	same
At_RPP7	NL	Bvu	snap_masked-Contig2505-processed-gene-0.2-mRNA	60.68	412	1	409	1.69E-171	NBS	different
At_WRR8	TNL	Bvu	maker-Contig1769-snap-gene-0.9-mRNA-1	63.835	412	340	745	7.42E-172	NL	different
At_NGR1a	RNL	Bvu	maker-Contig374-snap-gene-0.22-mRNA-1	64.563	412	404	808	3.24E-173	CNL	different
At_RPP1	TNL	Bvu	maker-Contig900-snap-gene-0.13-mRNA-1	64.086	465	701	1163	3.16E-173	TNL	same
Bra_Crr1a	TNL	Bvu	snap_masked-Contig196-processed-gene-0.21-mRNA	60.9	422	67	483	2.59E-173	TN	different
At_RFO1	Other-RLK	Bvu	maker-Contig264-snap-gene-0.42-mRNA-1	62.927	410	350	751	2.29E-174	Other-RLK	same
At_BAK1	LRR-RLK	Bvu	maker-Contig1644-snap-gene-0.13-mRNA-1	78.896	308	244	548	1.52E-175	Other-RLK	different
At_RPP2a	TNL	Bvu	maker-Contig989-snap-gene-0.32-mRNA-1	68.796	407	8	414	2.23E-176	TNL	same
At_RPP1	TNL	Bvu	snap_masked-Contig196-processed-gene-0.21-mRNA	60.706	425	94	513	2.38E-177	TN	different
At_SOBIR1	LRR-RLK	Bvu	snap_masked-Contig942-processed-gene-0.20-mRNA	88.456	641	1	641	0	LRR-RLK	same
At_RPM1	NL	Bvu	snap_masked-Contig376-processed-gene-0.14-mRNA	85.006	887	1	881	0	NL	same
At_FLS2	LRR-RLK	Bvu	maker-Contig505-snap-gene-0.21-mRNA-1	83.029	1149	1	1145	0	LRR-RLK	same
At_RLP32	LRR-RLP	Bvu	maker-Contig428-snap-gene-1.46-mRNA-1	82.73	857	1	854	0	LRR-RLP	same
At_RFO1	Other-RLK	Bvu	maker-Contig110-snap-gene-1.49-mRNA-1	82.205	753	3	751	0	Other-RLK	same
Bna_Rlm9/4/7	Other-RLK	Bvu	maker-Contig2524-snap-gene-0.16-mRNA-1	79.526	464	344	794	0	Other-RLK	same
At_BAK1	LRR-RLK	Bvu	maker-Contig303-snap-gene-0.56-mRNA-1	79.208	606	24	615	0	LRR-RLK	same
At_ADR1	NL	Bvu	maker-Contig1214-snap-gene-0.17-mRNA-1	78.544	783	3	778	0	NL	same
At_BAK1	LRR-RLK	Bvu	maker-Contig1522-snap-gene-0.10-mRNA-1	77.521	605	23	615	0	LRR-RLK	same
At_RPP8	CNL	Bvu	maker-Contig4596-snap-gene-0.1-mRNA-1	76.54	422	1	420	0	CNL	same
At_RPP8	CNL	Bvu	maker-Contig5728-snap-gene-0.2-mRNA-1	76.371	474	1	470	0	CNL	same
At_RPS4	TNL	Bvu	maker-Contig58-snap-gene-0.53-mRNA-1	76.261	1209	1	1193	0	TNL	same
At_NGR1a	RNL	Bvu	maker-Contig374-snap-gene-0.21-mRNA-1	75.137	547	1	540	0	CNL	different
At_RPP2b	TNL	Bvu	maker-Contig287-snap-gene-0.45-mRNA-1	74.75	1200	1	1193	0	TNL	same
Bna_MPK9	Other-RLK	Bvu	maker-Contig903-snap-gene-0.16-mRNA-1	74.359	546	63	591	0	Other-RLK	same
At_RFO3	Other-RLK	Bvu	maker-Contig110-snap-gene-1.40-mRNA-1	74.327	892	1	849	0	Other-RLK	same
At_WRR4b	TNL	Bvu	maker-Contig544-snap-gene-0.19-mRNA-1	74.17	542	3	534	0	TN	different
At_RPP8	CNL	Bvu	maker-Contig183-snap-gene-1.35-mRNA-1	73.937	541	1	537	0	CNL	same
At_WRR8	TNL	Bvu	maker-Contig751-snap-gene-0.13-mRNA-1	73.714	525	14	534	0	TN	different
At_RFO3	Other-RLK	Bvu	maker-Contig3131-snap-gene-0.3-mRNA-1	73.624	872	1	850	0	Other-RLK	same
At_RPP8	CNL	Bvu	maker-Contig2028-snap-gene-0.12-mRNA-1	73.319	476	94	564	0	NBS	different
At_RPP8	CNL	Bvu	maker-Contig6075-snap-gene-0.2-mRNA-1	73.124	893	1	888	0	CNL	same
At_RPP8	CNL	Bvu	maker-Contig892-snap-gene-0.8-mRNA-1	72.846	917	1	905	0	CNL	same
At_RPP8	CNL	Bvu	maker-Contig5728-snap-gene-0.3-mRNA-1	72.005	818	1	812	0	CNL	same
At_RPP8	CNL	Bvu	maker-Contig3090-snap-gene-0.6-mRNA-1	71.927	659	250	908	0	NL	different
At_RFO2	LRR-RLP	Bvu	snap_masked-Contig635-processed-gene-0.0-mRNA-	71.111	720	19	737	0	LRR-RLK	different
Bna_MPK9	Other-RLK	Bvu	maker-Contig60-snap-gene-0.75-mRNA-1	70.954	482	100	564	0	Other-RLK	same
At_RAC1	TNL	Bvu	maker-Contig751-snap-gene-0.13-mRNA-1	70.51	529	3	526	0	TN	different
At_RLP23	LRR-RLP	Bvu	snap_masked-Contig1356-processed-gene-0.11-mRN	70.159	878	17	890	0	LRR-RLP	same
At_WRR4b	TNL	Bvu	maker-Contig2444-snap-gene-0.5-mRNA-1	70.095	943	165	1091	0	NL	different
At_RPP13	CNL	Bvu	snap_masked-Contig408-processed-gene-0.13-mRNA	70.035	861	1	830	0	CNL	same
At_WRR8	TNL	Bvu	maker-Contig753-snap-gene-0.34-mRNA-1	69.874	1112	11	1102	0	TNL	same
At_RLP32	LRR-RLP	Bvu	maker-Contig1906-snap-gene-0.12-mRNA-1	69.757	453	397	849	0	LRR-RLP	same
At_RPP39	CNL	Bvu	snap_masked-Contig5814-processed-gene-0.1-mRNA	69.618	576	305	866	0	NL	different
At_RPP39	CNL	Bvu	maker-Contig4589-snap-gene-0.4-mRNA-1	69.371	875	1	866	0	CNL	same
At_RPP8	CNL	Bvu	maker-Contig1104-snap-gene-0.53-mRNA-1	69.358	904	1	899	0	CNL	same
Bra_Crr1a	TNL	Bvu	maker-Contig848-snap-gene-0.13-mRNA-1	69.231	442	64	503	0	TN	different
At_WRR4a	TNL	Bvu	maker-Contig544-snap-gene-0.19-mRNA-1	68.877	543	1	531	0	TN	different
At_RLP32	LRR-RLP	Bvu	snap_masked-Contig2365-processed-gene-0.6-mRNA	68.63	883	1	854	0	LRR-RLP	same
At_RPP39	CNL	Bvu	maker-Contig2932-snap-gene-0.20-mRNA-1	68.514	848	1	840	0	CNL	same
Bna_Rlm9/4/7	Other-RLK	Bvu	maker-Contig110-snap-gene-1.49-mRNA-1	68.123	778	23	794	0	Other-RLK	same
Bna_LepR3/Rlm2	LRR-RLP	Bvu	maker-Contig1906-snap-gene-0.12-mRNA-1	68.067	476	474	944	0	LRR-RLP	same
At_WRR8	TNL	Bvu	maker-Contig57-snap-gene-0.46-mRNA-1	67.985	531	19	543	0	TN	different
At_RPP7	NL	Bvu	maker-Contig883-snap-gene-0.14-mRNA-1	67.643	683	190	866	0	NL	same
At_RLP32	LRR-RLP	Bvu	maker-Contig4970-snap-gene-0.6-mRNA-1	67.595	790	66	853	0	LRR-RLP	same
At_NGR1a	RNL	Bvu	maker-Contig374-snap-gene-0.21-mRNA-1	67.233	824	1	807	0	CNL	different
At_RRS1	TNL	Bvu	maker-Contig2674-snap-gene-0.6-mRNA-1	67.208	677	8	681	0	NL	different
At_RLP42	LRR-RLP	Bvu	snap_masked-Contig1356-processed-gene-0.11-mRN	66.935	871	15	880	0	LRR-RLP	same
At_RAC1	TNL	Bvu	maker-Contig57-snap-gene-0.46-mRNA-1	66.792	533	9	534	0	TN	different
At_WRR8	TNL	Bvu	maker-Contig4851-snap-gene-0.8-mRNA-1	66.753	1155	12	1130	0	TNL	same
At_RPP7	NL	Bvu	snap_masked-Contig505-processed-gene-0.8-mRNA-	66.744	866	6	866	0	NL	same
Bra_Crr1a	TNL	Bvu	maker-Contig934-snap-gene-0.4-mRNA-1	66.731	517	68	584	0	TN	different
At_RAC1	TNL	Bvu	maker-Contig753-snap-gene-0.34-mRNA-1	66.696	1135	1	1121	0	TNL	same
At_RPS4	TNL	Bvu	maker-Contig58-snap-gene-0.55-mRNA-1	66.695	1165	7	1165	0	TNL	same
At_RPS4	TNL	Bvu	maker-Contig2674-snap-gene-0.3-mRNA-1	66.608	1141	4	1137	0	TNL	same
Bju_WRR1	CNL	Bvu	maker-Contig183-snap-gene-1.35-mRNA-1	66.605	542	1	534	0	CNL	same
At_RPP39	CNL	Bvu	maker-Contig859-snap-gene-0.10-mRNA-1	66.364	880	1	870	0	CNL	same
At_RPS5	TNL	Bvu	snap_masked-Contig5921-processed-gene-0.3-mRNA	66.1	882	1	880	0	CNL	different
At_NGR1b	RNL	Bvu	maker-Contig374-snap-gene-0.21-mRNA-1	66.093	814	9	812	0	CNL	different
At_RAC1	TNL	Bvu	maker-Contig6855-snap-gene-0.3-mRNA-1	66.003	753	9	735	0	TNL	same
At_RLP1	LRR-RLP	Bvu	maker-Contig1473-snap-gene-0.20-mRNA-1	65.893	862	216	1071	0	LRR-RLP	same
At_RLM1b	TNL	Bvu	maker-Contig1716-snap-gene-0.17-mRNA-1	65.882	1020	1	992	0	TNL	same
At_WRR8	TNL	Bvu	maker-Contig6855-snap-gene-0.3-mRNA-1	65.796	766	20	755	0	TNL	same

At_RPP7	NL	Bvu	maker-Contig5064-snap-gene-0.1-mRNA-1	65.711	872	4	866	0	NL	same
At_RLM1b	TNL	Bvu	snap_masked-Contig3802-processed-gene-0.1-mRNA	65.396	971	33	972	0	TNL	same
At_RPS5	TNL	Bvu	maker-Contig5921-snap-gene-0.8-mRNA-1	65.389	887	1	881	0	CNL	different
At_RRS1	TNL	Bvu	maker-Contig58-snap-gene-0.67-mRNA-1	65.336	1177	8	1169	0	TNL	same
At_NGR1b	RNL	Bvu	maker-Contig374-snap-gene-0.21-mRNA-1	65.28	553	5	546	0	CNL	different
Bju_WRR1	CNL	Bvu	maker-Contig4596-snap-gene-0.1-mRNA-1	65.248	423	1	413	0	CNL	same
Bol_FocBo1	TNL	Bvu	maker-Contig715-snap-gene-0.36-mRNA-1	65.156	706	8	711	0	TNL	same
Bna_LepR3/Rlm2	LRR-RLP	Bvu	maker-Contig4970-snap-gene-0.6-mRNA-1	64.896	772	187	950	0	LRR-RLP	same
Bna_Rlm9/4/7	Other-RLK	Bvu	maker-Contig264-snap-gene-0.42-mRNA-1	64.414	444	359	793	0	Other-RLK	same
Bju_WRR1	CNL	Bvu	maker-Contig5728-snap-gene-0.2-mRNA-1	64.346	474	1	466	0	CNL	same
At_RAC1	TNL	Bvu	maker-Contig4851-snap-gene-0.8-mRNA-1	64.212	1168	1	1148	0	TNL	same
Bna_LepR3/Rlm2	LRR-RLP	Bvu	snap_masked-Contig2399-processed-gene-0.6-mRNA	64.062	960	1	948	0	LRR-RLP	same
Bra_Crr1a	TNL	Bvu	maker-Contig1103-snap-gene-0.17-mRNA-1	63.724	521	66	586	0	TN	different
Bol_FocBo1	TNL	Bvu	maker-Contig989-snap-gene-0.32-mRNA-1	63.425	905	6	907	0	TNL	same
At_RPS5	TNL	Bvu	maker-Contig3871-snap-gene-0.8-mRNA-1	63.33	889	1	883	0	NL	different
At_RPP4	TNL	Bvu	maker-Contig715-snap-gene-0.36-mRNA-1	63.22	677	12	682	0	TNL	same
Bra_Crr1a	TNL	Bvu	maker-Contig3153-snap-gene-0.3-mRNA-1	63.123	602	68	665	0	TNL	same
At_RPP5	TNL	Bvu	maker-Contig715-snap-gene-0.36-mRNA-1	62.857	700	8	695	0	TNL	same
At_RFO1	Other-RLK	Bvu	maker-Contig382-snap-gene-0.50-mRNA-1	62.781	755	7	751	0	Other-RLK	same
At_RLM1a	TNL	Bvu	maker-Contig544-snap-gene-0.19-mRNA-1	62.731	542	1	532	0	TN	different
At_RPS5	TNL	Bvu	snap_masked-Contig4004-processed-gene-0.1-mRNA	62.606	706	184	883	0	NL	different
At_WRR8	TNL	Bvu	maker-Contig180-snap-gene-0.47-mRNA-1	62.534	734	20	745	0	TNL	same
At_RLM1b	TNL	Bvu	maker-Contig1442-snap-gene-0.28-mRNA-1	62.515	851	159	1000	0	NL	different
At_RPP1	TNL	Bvu	maker-Contig900-snap-gene-0.13-mRNA-1	62.38	832	187	1001	0	TNL	same
At_RPP8	CNL	Bvu	maker-Contig6075-snap-gene-0.2-mRNA-1	62.375	598	1	583	0	CNL	same
At_PBS1	Other-RLK	Bvu	snap_masked-Contig159-processed-gene-0.39-mRNA	62.282	517	2	455	0	Other-RLK	same
Bju_WRR1	CNL	Bvu	maker-Contig1104-snap-gene-0.53-mRNA-1	62.266	909	1	900	0	CNL	same
At_RLP1	LRR-RLP	Bvu	maker-Contig3997-snap-gene-0.4-mRNA-1	62.172	838	249	1071	0	LRR-RLP	same
At_RLM1a	TNL	Bvu	maker-Contig605-snap-gene-0.26-mRNA-1	62.105	475	164	636	0	NL	different
At_RLM1a	TNL	Bvu	maker-Contig1716-snap-gene-0.17-mRNA-1	62.048	996	16	996	0	TNL	same
At_WRR4a	TNL	Bvu	maker-Contig2444-snap-gene-0.5-mRNA-1	61.91	848	163	1004	0	NL	different
At_WRR8	TNL	Bvu	maker-Contig761-snap-gene-0.17-mRNA-1	61.864	1159	12	1138	0	TNL	same
Bna_LepR3/Rlm2	LRR-RLP	Bvu	snap_masked-Contig2365-processed-gene-0.6-mRNA	61.673	801	159	950	0	LRR-RLP	same
At_WRR9	NL	Bvu	maker-Contig605-snap-gene-0.26-mRNA-1	61.555	463	168	627	0	NL	same
At_ADR1	NL	Bvu	maker-Contig522-snap-gene-0.41-mRNA-1	61.425	814	6	787	0	NL	same
Bna_Rlm9/4/7	Other-RLK	Bvu	maker-Contig1460-snap-gene-0.33-mRNA-1	61.423	731	22	738	0	Other-RLK	same
At_WRR8	TNL	Bvu	maker-Contig355-snap-gene-0.7-mRNA-1	61.392	790	337	1107	0	TNL	same
Bju_WRR1	CNL	Bvu	maker-Contig2028-snap-gene-0.12-mRNA-1	61.325	468	95	554	0	NBS	different
At_RAC1	TNL	Bvu	maker-Contig180-snap-gene-0.47-mRNA-1	61.277	736	9	735	0	TNL	same
At_WRR8	TNL	Bvu	maker-Contig6634-snap-gene-0.2-mRNA-1	61.234	859	19	864	0	TNL	same
At_RFO1	Other-RLK	Bvu	maker-Contig1460-snap-gene-0.33-mRNA-1	61.096	712	34	732	0	Other-RLK	same
At_RPP1	TNL	Bvu	maker-Contig900-snap-gene-0.15-mRNA-1	61.004	777	244	983	0	NL	different
Bju_WRR1	CNL	Bvu	maker-Contig6075-snap-gene-0.2-mRNA-1	60.987	892	1	885	0	CNL	same
At_RLM1b	TNL	Bvu	maker-Contig1718-snap-gene-0.2-mRNA-1	60.978	1043	1	1001	0	TNL	same
At_RAC1	TNL	Bvu	maker-Contig355-snap-gene-0.7-mRNA-1	60.924	801	327	1114	0	TNL	same
At_RLP30	LRR-RLP	Bvu	snap_masked-Contig1346-processed-gene-0.6-mRNA	60.914	788	2	786	0	LRR-RLP	same
At_RPP1	TNL	Bvu	maker-Contig934-snap-gene-0.4-mRNA-1	60.886	519	94	611	0	TN	different
At_RLM1b	TNL	Bvu	maker-Contig544-snap-gene-0.19-mRNA-1	60.878	547	1	537	0	TN	different
Bju_WRR1	CNL	Bvu	maker-Contig892-snap-gene-0.8-mRNA-1	60.765	915	1	898	0	CNL	same
At_RPP39	CNL	Bvu	snap_masked-Contig862-processed-gene-0.0-mRNA-	60.701	542	305	837	0	NL	different
At_RLM1b	TNL	Bvu	maker-Contig605-snap-gene-0.26-mRNA-1	60.465	473	164	634	0	NL	different
At_WRR8	TNL	Bvu	maker-Contig7543-snap-gene-0.1-mRNA-1	60.36	666	225	885	0	NL	different
At_RLP32	LRR-RLP	Bvu	snap_masked-Contig2399-processed-gene-0.6-mRNA	60.216	925	1	854	0	LRR-RLP	same
At_RLP42	LRR-RLP	Bvu	snap_masked-Contig490-processed-gene-0.28-mRNA	60.085	704	182	880	0	LRR-RLP	same
Bna_LepR3/Rlm2	LRR-RLP	Bvu	maker-Contig2251-snap-gene-0.3-mRNA-1	60.062	974	1	944	0	LRR-RLP	same
At_RPP5	TNL	Bst	Bostr.9638s0056.1.p	66.443	149	10	158	1.13E-52	TNL	same
At_RPP4	TNL	Bst	Bostr.5342s0004.1.p	60.452	177	13	183	2.75E-55	Other-NLR	different
At_RPP5	TNL	Bst	Bostr.5342s0004.1.p	61.667	180	12	185	4.42E-56	Other-NLR	different
Bol_FocBo1	TNL	Bst	Bostr.5342s0004.1.p	61.798	178	10	181	2.83E-56	Other-NLR	different
At_RPP7	NL	Bst	Bostr.10040s0304.1.p	65.497	171	1	170	1.09E-61	NL	same
At_RPS5	TNL	Bst	Bostr.22157s0081.1.p	65.244	164	362	523	3.68E-64	NBS	different
At_RLP30	LRR-RLP	Bst	Bostr.15697s0391.1.p	62.371	194	570	760	8.02E-69	LRR-RLP	same
At_RPP7	NL	Bst	Bostr.10040s0329.1.p	61.856	194	90	280	4.64E-73	NBS	different
At_RPP7	NL	Bst	Bostr.10040s0305.1.p	64.629	229	1	228	3.17E-82	NL	same
Bju_WRR1	CNL	Bst	Bostr.14419s0022.1.p	60.079	253	1	251	3.28E-88	NL	different
At_RPP2b	TNL	Bst	Bostr.2983s0177.1.p	65.432	243	1	240	1.79E-89	TNL	same
Bra_Crr1a	TNL	Bst	Bostr.2983s0062.1.p	70.183	218	62	279	5.53E-92	TX	different
At_RLM1b	TNL	Bst	Bostr.29223s0118.1.p	73.214	224	1	224	5.12E-102	TX	different
At_RPP8	CNL	Bst	Bostr.14419s0022.1.p	69.141	256	1	256	1.55E-105	NL	different
At_RPP2a	TNL	Bst	Bostr.29223s0087.1.p	61.745	298	117	414	1.70E-113	TNL	same
At_WRR9	NL	Bst	Bostr.29223s0118.1.p	80.889	225	5	229	1.27E-114	TX	different
At_RLP30	LRR-RLP	Bst	Bostr.15697s0393.1.p	61.159	345	427	769	1.01E-119	LRR-RLP	same
At_RPS4	TNL	Bst	Bostr.18351s0043.1.p	60.59	373	211	572	1.28E-131	NBS	different
At_RPP2a	TNL	Bst	Bostr.2983s0178.1.p	68.868	318	533	849	4.02E-132	Other-NLR	different
At_WRR4a	TNL	Bst	Bostr.8169s0020.1.p	70.227	309	1	309	2.43E-138	TX	different
At_RPP1	TNL	Bst	Bostr.29514s0003.1.p	64.921	382	248	613	2.14E-150	Other-NLR	different
At_RPP2a	TNL	Bst	Bostr.29223s0077.1.p	63.636	352	10	361	2.18E-151	TN	different
At_RPP1	TNL	Bst	Bostr.2983s0062.1.p	75.316	316	1	305	9.25E-152	TX	different
At_RLM3	TN	Bst	Bostr.29223s0077.1.p	62.393	351	9	359	6.18E-154	TN	same
At_RPP2a	TNL	Bst	Bostr.29223s0076.1.p	65.228	417	1	414	3.66E-161	TNL	same
At_RPP2a	TNL	Bst	Bostr.29223s0089.1.p	63.547	406	10	414	5.48E-162	TNL	same
At_NGR1b	RNL	Bst	Bostr.0568s0113.1.p	62.56	414	406	815	3.79E-164	NL	different
At_RPP1	TNL	Bst	Bostr.7200s0011.1.p	60.816	490	693	1156	6.66E-166	Other-NLR	different
At_PBS1	Other-RLK	Bst	Bostr.25219s0162.1.p	68.966	319	71	381	1.71E-166	Other-RLK	same
At_RPP2a	TNL	Bst	Bostr.5342s0004.1.p	63.07	417	1	413	7.27E-167	Other-NLR	different
At_RPP2a	TNL	Bst	Bostr.29223s0095.1.p	65.025	406	10	414	1.70E-167	TNL	same

At_WRR4b	TNL	Bst	Bostr.8169s0020.1.p	81.029	311	4	314	1.01E-167	TX	different
At_RPP2a	TNL	Bst	Bostr.3148s0224.1.p	64.029	417	1	413	8.16E-168	Other-NLR	different
At_RPP2a	TNL	Bst	Bostr.29223s0069.1.p	65.459	414	1	414	1.69E-168	Other-NLR	different
At_RPP2a	TNL	Bst	Bostr.29223s0074.1.p	62.319	414	1	414	1.62E-169	TN	different
At_RPP2a	TNL	Bst	Bostr.29223s0096.1.p	65.111	407	10	414	1.22E-170	TNL	same
At_RPP8	CNL	Bst	Bostr.13671s0460.1.p	64.16	399	47	445	2.16E-172	NBS	different
At_RLM3	TN	Bst	Bostr.29223s0072.1.p	60.185	432	9	432	1.03E-172	Other-NLR	different
At_RPP2a	TNL	Bst	Bostr.29223s0100.1.p	64.976	414	1	414	7.00E-173	TNL	same
At_RPP2a	TNL	Bst	Bostr.29223s0080.1.p	65.217	414	1	414	6.82E-177	TNL	same
At_NGR1a	RNL	Bst	Bostr.0568s0113.1.p	66.423	411	404	809	1.39E-177	NL	different
At_RPP2a	TNL	Bst	Bostr.29223s0072.1.p	66.425	414	1	414	2.60E-178	Other-NLR	different
At_BAK1	LRR-RLK	Bst	Bostr.7867s1189.1.p	99.187	615	1	615	0	LRR-RLK	same
At_FLS2	LRR-RLK	Bst	Bostr.8819s0170.1.p	89.505	1172	1	1170	0	LRR-RLK	same
At_SOBIR1	LRR-RLK	Bst	Bostr.24513s0040.1.p	89.236	641	1	641	0	LRR-RLK	same
At_RPS2	NL	Bst	Bostr.7867s0427.1.p	87.239	909	1	909	0	CNL	different
At_RPS4	TNL	Bst	Bostr.25599s0001.2.p	85.528	919	34	952	0	TNL	same
At_RPS4	TNL	Bst	Bostr.25599s0001.1.p	84.643	1120	34	1144	0	TNL	same
At_BAK1	LRR-RLK	Bst	Bostr.13083s0025.1.p	82.391	619	1	615	0	LRR-RLK	same
At_RPP2b	TNL	Bst	Bostr.2983s0177.1.p	80.858	606	589	1193	0	TNL	same
At_BAK1	LRR-RLK	Bst	Bostr.10273s0172.1.p	79.504	605	24	615	0	LRR-RLK	same
At_WRR4b	TNL	Bst	Bostr.8169s0027.2.p	79.266	1090	1	1076	0	TNL	same
At_WRR4b	TNL	Bst	Bostr.8169s0027.1.p	79.266	1090	1	1076	0	TNL	same
At_WRR8	TNL	Bst	Bostr.28243s0020.1.p	78.933	356	12	367	0	TNL	same
At_RPP2a	TNL	Bst	Bostr.30275s0429.1.p	78.813	859	409	1257	0	TNL	same
At_RFO3	Other-RLK	Bst	Bostr.28625s0088.1.p	77.586	870	1	850	0	Other-RLK	same
At_BAK1	LRR-RLK	Bst	Bostr.1040s0048.1.p	76.414	619	9	615	0	LRR-RLK	same
At_RPP1	TNL	Bst	Bostr.29514s0003.1.p	75.41	427	67	493	0	Other-NLR	different
At_WRR9	NL	Bst	Bostr.29223s0183.2.p	75.16	624	5	627	0	TNL	different
At_RLM1b	TNL	Bst	Bostr.29223s0183.2.p	74.96	627	1	626	0	TNL	same
At_WRR4b	TNL	Bst	Bostr.30057s0091.3.p	74.648	710	5	713	0	TNL	same
At_WRR4b	TNL	Bst	Bostr.30057s0091.2.p	74.648	710	5	713	0	TNL	same
At_RLM1b	TNL	Bst	Bostr.29223s0183.1.p	74.623	729	1	728	0	TNL	same
At_WRR9	NL	Bst	Bostr.29223s0183.1.p	74.242	726	5	729	0	TNL	different
At_WRR8	TNL	Bst	Bostr.4485s0004.1.p	74.232	586	12	593	0	TN	different
At_RAC1	TNL	Bst	Bostr.4485s0004.1.p	74.106	587	1	582	0	TN	different
At_WRR4a	TNL	Bst	Bostr.8169s0019.1.p	74.087	849	43	890	0	TNL	same
At_WRR4a	TNL	Bst	Bostr.30057s0091.3.p	74.085	710	3	711	0	TNL	same
At_WRR4a	TNL	Bst	Bostr.30057s0091.2.p	74.085	710	3	711	0	TNL	same
At_RPP39	CNL	Bst	Bostr.10040s0071.2.p	73.542	703	1	698	0	CNL	same
At_WRR4a	TNL	Bst	Bostr.30057s0091.1.p	73.453	889	3	890	0	TNL	same
At_RPP1	TNL	Bst	Bostr.26675s0373.1.p	73.188	414	96	508	0	Other-NLR	different
At_WRR9	NL	Bst	Bostr.29223s0198.2.p	73.161	734	5	733	0	TNL	different
At_WRR9	NL	Bst	Bostr.29223s0198.1.p	73.161	734	5	733	0	TNL	different
At_WRR4b	TNL	Bst	Bostr.8169s0028.1.p	72.748	433	1	433	0	TN	different
At_RAC1	TNL	Bst	Bostr.8819s0184.1.p	72.598	1197	1	1160	0	TNL	same
At_RPP8	CNL	Bst	Bostr.0556s0548.1.p	72.581	744	150	889	0	NL	different
At_RPP39	CNL	Bst	Bostr.10040s0071.1.p	72.562	882	1	870	0	CNL	same
At_WRR4b	TNL	Bst	Bostr.30057s0091.1.p	72.542	885	5	888	0	TNL	same
At_RPP39	CNL	Bst	Bostr.10040s0072.1.p	72.531	881	1	870	0	CNL	same
At_WRR4b	TNL	Bst	Bostr.8169s0018.1.p	72.41	946	159	1096	0	NL	different
At_RAC1	TNL	Bst	Bostr.4485s0002.1.p	72.339	1193	1	1156	0	TNL	same
At_RPP39	CNL	Bst	Bostr.10040s0075.1.p	72.336	882	1	870	0	CNL	same
At_RLM1b	TNL	Bst	Bostr.29223s0155.1.p	72.111	796	102	876	0	TNL	same
At_RPP8	CNL	Bst	Bostr.0124s0128.1.p	72.101	914	1	908	0	CNL	same
At_RPP39	CNL	Bst	Bostr.10040s0099.1.p	72.022	890	1	869	0	CNL	same
At_WRR4b	TNL	Bst	Bostr.8169s0019.1.p	71.986	846	45	888	0	TNL	same
At_RPP39	CNL	Bst	Bostr.10040s0079.1.p	71.919	844	42	874	0	CNL	same
At_RPM1	NL	Bst	Bostr.22252s0330.1.p	71.875	928	1	926	0	NL	same
At_WRR9	NL	Bst	Bostr.29223s0031.1.p	71.701	629	12	627	0	TNL	different
At_RPP39	CNL	Bst	Bostr.10040s0136.1.p	71.397	895	1	883	0	CNL	same
At_RFO2	LRR-RLP	Bst	Bostr.10273s0122.1.p	71.349	719	19	737	0	LRR-RLK	different
At_RAC1	TNL	Bst	Bostr.8819s0189.2.p	71.309	1192	1	1156	0	TNL	same
At_RAC1	TNL	Bst	Bostr.8819s0189.1.p	71.249	1193	1	1157	0	TNL	same
At_BAK1	LRR-RLK	Bst	Bostr.19424s1005.1.p	71.218	476	141	615	0	LRR-RLK	same
At_RLP1	LRR-RLP	Bst	Bostr.4704s0001.1.p	71.209	910	165	1071	0	LRR-RLP	same
At_RLM1b	TNL	Bst	Bostr.8169s0059.1.p	71.109	938	3	934	0	TNL	same
At_RPP39	CNL	Bst	Bostr.10040s0026.1.p	71.036	618	260	870	0	NL	different
At_RPP39	CNL	Bst	Bostr.10040s0027.1.p	70.828	737	145	870	0	NL	different
At_RAC1	TNL	Bst	Bostr.28243s0042.1.p	70.593	1197	1	1160	0	TNL	same
At_RLM1a	TNL	Bst	Bostr.29223s0208.1.p	70.588	425	11	433	0	TN	different
At_RPP39	CNL	Bst	Bostr.10040s0078.1.p	70.522	882	1	870	0	CNL	same
At_WRR8	TNL	Bst	Bostr.28243s0042.1.p	70.364	1181	12	1138	0	TNL	same
At_RPP7	NL	Bst	Bostr.10040s0328.1.p	70.308	714	1	708	0	NL	same
At_RLM1b	TNL	Bst	Bostr.29223s0162.1.p	70.215	1024	1	1012	0	TNL	same
At_RAC1	TNL	Bst	Bostr.13083s0042.1.p	70.139	1152	1	1139	0	TNL	same
At_WRR8	TNL	Bst	Bostr.4485s0017.1.p	70.079	1133	12	1131	0	TNL	same
At_WRR8	TNL	Bst	Bostr.8819s0189.2.p	70.059	1179	12	1138	0	TNL	same
At_WRR8	TNL	Bst	Bostr.8819s0189.1.p	70.059	1179	12	1138	0	TNL	same
At_WRR8	TNL	Bst	Bostr.13083s0042.1.p	69.991	1133	12	1122	0	TNL	same
At_RPP8	CNL	Bst	Bostr.0124s0128.2.p	69.869	687	227	908	0	NL	different
At_RLM1b	TNL	Bst	Bostr.29223s0114.1.p	69.804	1020	10	1013	0	TNL	same
At_RLM1b	TNL	Bst	Bostr.29223s0184.1.p	69.737	988	1	977	0	TNL	same
At_WRR8	TNL	Bst	Bostr.8819s0184.1.p	69.613	1188	12	1138	0	TNL	same
At_RAC1	TNL	Bst	Bostr.28243s0018.2.p	69.456	1195	1	1149	0	TNL	same
At_RAC1	TNL	Bst	Bostr.28243s0018.1.p	69.456	1195	1	1149	0	TNL	same
At_RAC1	TNL	Bst	Bostr.4485s0017.1.p	68.992	1161	1	1149	0	TNL	same

At_WRR4a	TNL	Bst	Bostr.8169s0021.1.p	68.835	1030	1	1004	0	TNL	same
Bna_Rlm9/4/7	Other-RLK	Bst	Bostr.26959s0385.1.p	68.822	696	100	794	0	Other-RLK	same
At_RLM1b	TNL	Bst	Bostr.29223s0151.1.p	68.574	1031	1	1014	0	TNL	same
At_WRR8	TNL	Bst	Bostr.28243s0018.2.p	68.52	1169	12	1131	0	TNL	same
At_WRR8	TNL	Bst	Bostr.28243s0018.1.p	68.52	1169	12	1131	0	TNL	same
At_RPP1	TNL	Bst	Bostr.0556s0745.1.p	68.421	1254	1	1163	0	Other-NLR	different
At_RAC1	TNL	Bst	Bostr.28243s0020.1.p	68.408	1168	1	1149	0	TNL	same
At_RLM1b	TNL	Bst	Bostr.29223s0029.1.p	68.379	1012	11	1009	0	TNL	same
At_NGR1a	RNL	Bst	Bostr.27102s0001.1.p	68.283	495	12	501	0	CN	different
At_RAC1	TNL	Bst	Bostr.28243s0043.2.p	68.209	1167	9	1156	0	TNL	same
At_RAC1	TNL	Bst	Bostr.28243s0043.1.p	68.209	1167	9	1156	0	TNL	same
Bna_Rlm9/4/7	Other-RLK	Bst	Bostr.10273s0393.1.p	68.204	802	22	794	0	Other-RLK	same
At_WRR8	TNL	Bst	Bostr.28243s0020.1.p	68.163	735	420	1131	0	TNL	same
At_WRR4a	TNL	Bst	Bostr.12938s0002.1.p	68.116	897	102	996	0	TNL	same
At_RAC1	TNL	Bst	Bostr.28243s0018.3.p	68.095	1050	146	1149	0	NL	different
At_RAC1	TNL	Bst	Bostr.28243s0018.4.p	68.095	1050	146	1149	0	NL	different
At_WRR4a	TNL	Bst	Bostr.8169s0030.1.p	67.925	424	325	747	0	TNL	same
At_RRS1	TNL	Bst	Bostr.29514s0001.2.p	67.869	1220	1	1190	0	TNL	same
At_RLM1b	TNL	Bst	Bostr.29223s0160.1.p	67.682	1018	1	1009	0	TNL	same
At_WRR8	TNL	Bst	Bostr.28243s0043.2.p	67.509	1148	19	1138	0	TNL	same
At_WRR8	TNL	Bst	Bostr.28243s0043.1.p	67.509	1148	19	1138	0	TNL	same
At_RLM1a	TNL	Bst	Bostr.29223s0183.2.p	67.468	624	1	622	0	TNL	same
At_RLM1b	TNL	Bst	Bostr.29223s0031.1.p	67.353	631	11	626	0	TNL	same
At_WRR8	TNL	Bst	Bostr.4485s0002.1.p	67.311	1190	12	1138	0	TNL	same
At_RPS5	TNL	Bst	Bostr.13671s0231.1.p	67.31	881	1	880	0	CNL	different
At_RPP1	TNL	Bst	Bostr.7200s0014.1.p	67.145	1184	58	1163	0	Other-NLR	different
At_WRR9	NL	Bst	Bostr.29223s0147.1.p	67.136	995	5	987	0	TNL	different
At_RRS1	TNL	Bst	Bostr.29514s0001.1.p	67.095	1246	1	1216	0	TNL	same
At_RAC1	TNL	Bst	Bostr.28243s0017.1.p	67.048	1223	9	1156	0	TNL	same
At_RLM1b	TNL	Bst	Bostr.29223s0181.1.p	66.935	995	1	980	0	TNL	same
At_WRR9	NL	Bst	Bostr.29223s0151.1.p	66.863	1020	5	1009	0	TNL	different
At_RPP39	CNL	Bst	Bostr.22157s0136.1.p	66.793	527	355	870	0	CNL	same
At_WRR4b	TNL	Bst	Bostr.1040s0006.1.p	66.607	563	104	624	0	TNL	same
At_RLM1b	TNL	Bst	Bostr.10040s0141.1.p	66.576	739	164	901	0	NL	different
At_RPP1	TNL	Bst	Bostr.18473s0376.1.p	66.571	1041	1	915	0	Other-NLR	different
At_RAC1	TNL	Bst	Bostr.8819s0190.1.p	66.558	1229	1	1149	0	TNL	same
At_WRR8	TNL	Bst	Bostr.28243s0018.3.p	66.537	1025	156	1131	0	NL	different
At_WRR8	TNL	Bst	Bostr.28243s0018.4.p	66.537	1025	156	1131	0	NL	different
At_WRR4b	TNL	Bst	Bostr.8169s0020.1.p	66.425	691	396	1076	0	TX	different
At_WRR9	NL	Bst	Bostr.29223s0029.1.p	66.269	1005	12	1008	0	TNL	different
At_RRS1	TNL	Bst	Bostr.8819s0021.1.p	66.263	1322	1	1288	0	NL	different
At_WRR9	NL	Bst	Bostr.29223s0181.1.p	66.232	998	5	989	0	TNL	different
At_RLM1b	TNL	Bst	Bostr.29223s0121.1.p	66.223	752	278	1012	0	NL	different
At_WRR9	NL	Bst	Bostr.29223s0172.1.p	66.203	1006	12	1002	0	TNL	different
At_RLP23	LRR-RLP	Bst	Bostr.23794s0796.1.p	66.109	717	175	889	0	LRR-RLP	same
At_WRR8	TNL	Bst	Bostr.28243s0017.1.p	66.007	1212	19	1138	0	TNL	same
At_RLM1b	TNL	Bst	Bostr.29223s0147.1.p	65.941	1010	1	990	0	TNL	same
At_RPP8	CNL	Bst	Bostr.30275s0049.1.p	65.714	910	1	904	0	CNL	same
At_WRR4a	TNL	Bst	Bostr.8169s0027.2.p	65.709	1009	9	1004	0	TNL	same
At_WRR4a	TNL	Bst	Bostr.8169s0027.1.p	65.709	1009	9	1004	0	TNL	same
At_RLM1a	TNL	Bst	Bostr.29223s0198.2.p	65.698	723	1	719	0	TNL	same
At_RLM1a	TNL	Bst	Bostr.29223s0198.1.p	65.698	723	1	719	0	TNL	same
At_RPP8	CNL	Bst	Bostr.30275s0049.2.p	65.652	821	1	815	0	CNL	same
At_RPP1	TNL	Bst	Bostr.19424s0067.1.p	65.65	492	94	584	0	TN	different
At_RLP32	LRR-RLP	Bst	Bostr.2199s0003.1.p	65.638	745	114	854	0	LRR-RLP	same
At_RPP1	TNL	Bst	Bostr.18473s0374.1.p	65.611	1294	1	1163	0	Other-NLR	different
At_WRR9	NL	Bst	Bostr.29223s0024.1.p	65.572	1005	5	995	0	TNL	different
At_RLP23	LRR-RLP	Bst	Bostr.23794s0775.1.p	65.551	717	175	888	0	LRR-RLP	same
At_WRR8	TNL	Bst	Bostr.8819s0190.1.p	65.455	1210	12	1131	0	TNL	same
At_RPP1	TNL	Bst	Bostr.26326s0044.1.p	65.414	1064	128	1161	0	Other-NLR	different
At_RPS4	TNL	Bst	Bostr.5342s0008.1.p	65.405	1136	9	1134	0	TNL	same
At_WRR8	TNL	Bst	Bostr.3148s0208.1.p	65.4	737	45	772	0	TNL	same
At_RPP1	TNL	Bst	Bostr.18351s0417.1.p	65.333	1275	1	1163	0	Other-NLR	different
At_RPP1	TNL	Bst	Bostr.0556s0496.1.p	65.175	1117	108	1160	0	Other-NLR	different
At_WRR4b	TNL	Bst	Bostr.1040s0084.1.p	65.068	1022	11	998	0	TNL	same
At_WRR9	NL	Bst	Bostr.29223s0033.1.p	65.024	1035	12	1026	0	TNL	different
At_RRS1	TNL	Bst	Bostr.5342s0006.1.p	64.948	1261	8	1238	0	TNL	same
At_RFO1	Other-RLK	Bst	Bostr.7128s0227.1.p	64.915	761	6	751	0	Other-RLK	same
At_RPP1	TNL	Bst	Bostr.18473s0352.1.p	64.848	825	176	983	0	Other-NLR	different
Bol_FocBo1	TNL	Bst	Bostr.29223s0074.1.p	64.803	483	8	490	0	TN	different
At_WRR9	NL	Bst	Bostr.29223s0114.1.p	64.802	983	13	987	0	TNL	different
At_RAC1	TNL	Bst	Bostr.8819s0175.1.p	64.777	741	1	735	0	TNL	same
At_WRR8	TNL	Bst	Bostr.8819s0175.1.p	64.682	739	12	745	0	TNL	same
At_WRR4b	TNL	Bst	Bostr.12938s0002.1.p	64.452	903	104	994	0	TNL	same
At_RPS5	TNL	Bst	Bostr.26675s0378.1.p	64.27	890	1	883	0	CNL	different
At_RLP42	LRR-RLP	Bst	Bostr.23794s0796.1.p	64.256	719	182	889	0	LRR-RLP	same
At_RPP5	TNL	Bst	Bostr.29223s0074.1.p	64.241	481	11	491	0	TN	different
At_RLP32	LRR-RLP	Bst	Bostr.19424s1033.1.p	64.224	928	1	853	0	LRR-RLP	same
At_WRR12	TNL	Bst	Bostr.3751s0023.1.p	64.133	1026	12	1019	0	TNL	same
At_RLM1a	TNL	Bst	Bostr.29223s0183.1.p	64.065	743	1	741	0	TNL	same
Bra_Crr1a	TNL	Bst	Bostr.25463s0391.2.p	64.041	1079	67	1044	0	TNL	same
At_RFO1	Other-RLK	Bst	Bostr.7128s0228.1.p	64.022	731	7	717	0	Other-RLK	same
At_RPP7	NL	Bst	Bostr.10040s0323.1.p	63.936	879	1	865	0	NL	same
Bju_WRR1	CNL	Bst	Bostr.0124s0128.1.p	63.916	909	1	900	0	CNL	same
At_RPP4	TNL	Bst	Bostr.29223s0074.1.p	63.808	478	12	489	0	TN	different
At_RPP7	NL	Bst	Bostr.10040s0305.1.p	63.787	544	243	775	0	NL	same

At_WRR4b	TNL	Bst	Bostr.2983s0178.1.p	63.778	900	160	1053	0	Other-NLR	different
At_WRR4a	TNL	Bst	Bostr.2983s0178.1.p	63.754	847	159	1000	0	Other-NLR	different
At_RAC1	TNL	Bst	Bostr.3148s0208.1.p	63.735	739	34	765	0	TNL	same
Bna_LepR3/Rlm2	LRR-RLP	Bst	Bostr.5776s0003.1.p	63.674	969	1	947	0	LRR-RLP	same
At_RLM1b	TNL	Bst	Bostr.29223s0172.1.p	63.672	1024	11	1013	0	TNL	same
At_RLM1b	TNL	Bst	Bostr.29223s0033.1.p	63.645	1026	11	1008	0	TNL	same
At_WRR12	TNL	Bst	Bostr.3751s0040.1.p	63.614	797	218	1010	0	NL	different
At_WRR12	TNL	Bst	Bostr.3751s0028.1.p	63.559	944	85	1021	0	TNL	same
At_WRR12	TNL	Bst	Bostr.3751s0032.1.p	63.434	1031	12	1019	0	TNL	same
At_RLM1a	TNL	Bst	Bostr.29223s0155.1.p	63.43	793	102	873	0	TNL	same
At_RAC1	TNL	Bst	Bostr.4485s0016.2.p	63.4	653	105	735	0	TNL	same
At_RAC1	TNL	Bst	Bostr.4485s0016.1.p	63.4	653	105	735	0	TNL	same
At_RLM1a	TNL	Bst	Bostr.30057s0091.3.p	63.38	710	4	712	0	TNL	same
At_RLM1a	TNL	Bst	Bostr.30057s0091.2.p	63.38	710	4	712	0	TNL	same
At_WRR12	TNL	Bst	Bostr.3751s0035.1.p	63.365	1040	12	1044	0	TNL	same
At_RPP7	NL	Bst	Bostr.25375s0006.2.p	63.303	545	254	793	0	NL	same
At_RPS5	TNL	Bst	Bostr.13671s0231.3.p	63.199	769	112	880	0	NL	different
At_RPS5	TNL	Bst	Bostr.13671s0231.2.p	63.199	769	112	880	0	NL	different
At_WRR4a	TNL	Bst	Bostr.0124s0150.1.p	62.985	1005	1	1004	0	TNL	same
At_RPP7	NL	Bst	Bostr.10040s0017.1.p	62.971	875	1	866	0	NL	same
At_WRR9	NL	Bst	Bostr.29223s0184.1.p	62.842	1098	5	1077	0	TNL	different
At_RLM1b	TNL	Bst	Bostr.29223s0118.1.p	62.828	495	373	865	0	TX	different
At_RLM1b	TNL	Bst	Bostr.8169s0058.1.p	62.745	1020	1	1009	0	TNL	same
At_RLP42	LRR-RLP	Bst	Bostr.23794s0775.1.p	62.712	708	182	883	0	LRR-RLP	same
At_WRR8	TNL	Bst	Bostr.4485s0016.2.p	62.673	651	116	745	0	TNL	same
At_WRR8	TNL	Bst	Bostr.4485s0016.1.p	62.673	651	116	745	0	TNL	same
At_WRR4b	TNL	Bst	Bostr.8169s0021.1.p	62.579	1101	4	1024	0	TNL	same
At_RPP1	TNL	Bst	Bostr.0556s0798.1.p	62.376	1111	66	1163	0	Other-NLR	different
Bna_LepR3/Rlm2	LRR-RLP	Bst	Bostr.2199s0003.1.p	62.37	962	1	947	0	LRR-RLP	same
Bra_Crr1a	TNL	Bst	Bostr.25463s0391.1.p	62.351	1259	67	1215	0	TNL	same
At_RPP1	TNL	Bst	Bostr.18473s0359.1.p	62.316	1019	58	1013	0	Other-NLR	different
At_RLP42	LRR-RLP	Bst	Bostr.23794s0774.1.p	62.302	695	182	876	0	LRR-RLP	same
At_WRR4a	TNL	Bst	Bostr.8169s0026.1.p	62.28	851	163	1004	0	NL	different
At_RLP23	LRR-RLP	Bst	Bostr.23794s0774.1.p	62.256	718	175	890	0	LRR-RLP	same
At_RLM1b	TNL	Bst	Bostr.10040s0058.1.p	62.241	964	33	981	0	Other-NLR	different
At_WRR4a	TNL	Bst	Bostr.8169s0018.1.p	62.134	853	157	1004	0	NL	different
At_RLM1b	TNL	Bst	Bostr.10040s0057.1.p	62.13	1014	1	997	0	TNL	same
At_RLP32	LRR-RLP	Bst	Bostr.19424s1114.1.p	62.125	866	6	853	0	LRR-RLP	same
At_RPP1	TNL	Bst	Bostr.2983s0062.1.p	62.109	768	476	1163	0	TX	different
At_RPS5	TNL	Bst	Bostr.13671s0240.1.p	62.091	794	1	790	0	CNL	different
At_WRR4b	TNL	Bst	Bostr.0124s0150.1.p	62.011	1074	6	1075	0	TNL	same
At_RFO2	LRR-RLP	Bst	Bostr.7128s0335.1.p	61.98	697	45	740	0	LRR-RLP	same
At_RPP7	NL	Bst	Bostr.10040s0324.1.p	61.786	963	1	956	0	NL	same
At_RLM1a	TNL	Bst	Bostr.10040s0141.1.p	61.737	737	164	898	0	NL	different
At_RLM1a	TNL	Bst	Bostr.29223s0162.1.p	61.709	995	1	986	0	TNL	same
At_RLM1a	TNL	Bst	Bostr.29223s0031.1.p	61.661	626	12	622	0	TNL	same
At_WRR9	NL	Bst	Bostr.29223s0155.1.p	61.606	797	106	881	0	TNL	different
Bju_WRR1	CNL	Bst	Bostr.0124s0128.2.p	61.606	685	222	900	0	NL	different
At_RPP7	NL	Bst	Bostr.10040s0313.1.p	61.597	526	1	525	0	NBS	different
At_RPP1	TNL	Bst	Bostr.18473s0352.1.p	61.583	518	659	1163	0	Other-NLR	different
At_RLM1b	TNL	Bst	Bostr.29223s0024.1.p	61.508	1008	1	992	0	TNL	same
At_WRR4b	TNL	Bst	Bostr.29223s0183.2.p	61.502	626	1	624	0	TNL	same
At_WRR8	TNL	Bst	Bostr.4485s0003.1.p	61.477	745	27	745	0	TNL	same
At_RPP7	NL	Bst	Bostr.25375s0006.1.p	61.452	799	1	793	0	NL	same
At_RLM1a	TNL	Bst	Bostr.10040s0227.1.p	61.438	918	1	915	0	TNL	same
At_WRR9	NL	Bst	Bostr.29223s0160.1.p	61.35	1022	5	1020	0	TNL	different
At_WRR9	NL	Bst	Bostr.10040s0141.1.p	61.325	755	168	920	0	NL	same
At_RLP32	LRR-RLP	Bst	Bostr.10199s0122.1.p	61.245	707	175	866	0	LRR-RLP	same
At_RPP1	TNL	Bst	Bostr.7200s0011.1.p	61.086	884	177	1001	0	Other-NLR	different
At_NGR1b	RNL	Bst	Bostr.27102s0001.1.p	61.044	498	14	503	0	CN	different
At_RLM1a	TNL	Bst	Bostr.30057s0091.1.p	61.036	888	4	889	0	TNL	same
At_RPP7	NL	Bst	Bostr.10040s0300.1.p	61.027	993	1	977	0	NL	same
At_RPP7	NL	Bst	Bostr.10040s0300.2.p	61.027	993	1	977	0	NL	same
At_WRR8	TNL	Bst	Bostr.4485s0018.1.p	61.02	549	282	751	0	NL	different
At_ADR1	NL	Bst	Bostr.14419s0021.1.p	61.017	590	179	765	0	NL	same
At_RPP7	NL	Bst	Bostr.7200s0186.1.p	61.002	1018	1	977	0	CNL	different
At_RFO1	Other-RLK	Bst	Bostr.7128s0225.1.p	60.957	648	119	725	0	Other-RLK	same
At_RPS5	TNL	Bst	Bostr.26675s0377.1.p	60.904	885	1	883	0	CNL	different
At_RLM1a	TNL	Bst	Bostr.8169s0019.1.p	60.895	849	44	889	0	TNL	same
At_WRR9	NL	Bst	Bostr.29223s0162.1.p	60.861	999	5	989	0	TNL	different
At_RLP32	LRR-RLP	Bst	Bostr.19424s1036.1.p	60.833	840	6	766	0	LRR-RLP	same
Bra_Crr1a	TNL	Bst	Bostr.18473s0376.1.p	60.823	924	70	975	0	Other-NLR	different
Bra_Crr1a	TNL	Bst	Bostr.30057s0025.3.p	60.806	620	70	688	0	TNL	same
At_RPP1	TNL	Bst	Bostr.30057s0025.3.p	60.778	617	98	711	0	TNL	same
Bra_cRa/cRb	TNL	Bst	Bostr.25463s0391.2.p	60.742	1159	1	1152	0	TNL	same
At_RPP39	CNL	Bst	Bostr.10040s0074.1.p	60.727	825	1	814	0	CNL	same
At_RPP7	NL	Bst	Bostr.10040s0277.1.p	60.593	1147	1	1136	0	CNL	different
At_RLM1a	TNL	Bst	Bostr.10040s0058.1.p	60.581	964	33	988	0	Other-NLR	different
At_RPP1	TNL	Bst	Bostr.30057s0025.1.p	60.567	705	98	799	0	TNL	same
At_RLM1b	TNL	Bst	Bostr.29223s0198.2.p	60.485	1030	1	1013	0	TNL	same
At_RLM1b	TNL	Bst	Bostr.29223s0198.1.p	60.485	1030	1	1013	0	TNL	same
Bra_Crr1a	TNL	Bst	Bostr.2570s0295.1.p	60.482	706	92	795	0	TNL	same
At_RPS5	TNL	Bst	Bostr.13671s0230.1.p	60.473	888	1	883	0	CNL	different
At_RLM1a	TNL	Bst	Bostr.8169s0059.1.p	60.453	928	3	925	0	TNL	same
At_RPS5	TNL	Bst	Bostr.15774s0296.1.p	60.384	886	1	881	0	CNL	different
Bra_Crr1a	TNL	Bst	Bostr.30057s0025.1.p	60.347	691	70	759	0	TNL	same

At_WRR4b	TNL	Bst	Bostr.29223s0183.1.p	60.331	726	1	724	0	TNL	same
At_WRR9	NL	Bst	Bostr.29223s0121.1.p	60.328	731	282	1001	0	NL	same
Bra_Crr1a	TNL	Bst	Bostr.2570s0335.1.p	60.302	728	92	815	0	TNL	same
Bna_LepR3/Rlm2	LRR-RLP	Bst	Bostr.18351s0001.1.p	60.171	934	25	947	0	LRR-RLP	same
Bju_WRR1	CNL	Bst	Bostr.0556s0548.1.p	60.16	748	142	886	0	NL	different
At_ADR1	NL	Bst	Bostr.7867s1179.1.p	60.073	819	3	787	0	NL	same
At_RLM1a	TNL	Bst	Bostr.10040s0057.1.p	60.04	996	1	988	0	TNL	same
At_RLM3	TN	Cgr	Cagra.1618s0019.1	60.39	154	3	152	3.10E-55	TX	different
At_RLM3	TN	Cgr	Cagra.1618s0019.1	60.39	154	3	152	3.10E-55	TX	different
At_WRR9	NL	Cgr	Cagra.1531s0001.1	61.333	150	8	157	1.91E-60	TX	different
At_WRR9	NL	Cgr	Cagra.1531s0001.1	61.333	150	8	157	1.91E-60	TX	different
At_RPP2a	TNL	Cgr	Cagra.1618s0019.1	64.557	158	1	154	2.57E-62	TX	different
At_RPP2a	TNL	Cgr	Cagra.1618s0019.1	64.557	158	1	154	2.57E-62	TX	different
At_RLM1b	TNL	Cgr	Cagra.1531s0001.1	66.667	153	4	156	3.48E-66	TX	different
At_RLM1b	TNL	Cgr	Cagra.1531s0001.1	66.667	153	4	156	3.48E-66	TX	different
Bol_FocBo1	TNL	Cgr	Cagra.1618s0019.1	67.901	162	6	163	8.06E-68	TX	different
Bol_FocBo1	TNL	Cgr	Cagra.1618s0019.1	67.901	162	6	163	8.06E-68	TX	different
At_RPP4	TNL	Cgr	Cagra.1618s0019.1	75	152	10	157	8.09E-70	TX	different
At_RPP4	TNL	Cgr	Cagra.1618s0019.1	75	152	10	157	8.09E-70	TX	different
At_RPP1	TNL	Cgr	Cagra.1761s0003.1	67.63	173	69	241	3.89E-73	TX	different
At_RPP1	TNL	Cgr	Cagra.1761s0003.1	67.63	173	69	241	3.89E-73	TX	different
At_WRR4a	TNL	Cgr	Cagra.1531s0001.1	73.377	154	3	156	3.33E-75	TX	different
At_WRR4a	TNL	Cgr	Cagra.1531s0001.1	73.377	154	3	156	3.33E-75	TX	different
At_WRR4b	TNL	Cgr	Cagra.1531s0001.1	78.571	154	5	158	2.18E-80	TX	different
At_WRR4b	TNL	Cgr	Cagra.1531s0001.1	78.571	154	5	158	2.18E-80	TX	different
At_RLM1a	TNL	Cgr	Cagra.1531s0001.1	77.922	154	4	157	6.15E-81	TX	different
At_RLM1a	TNL	Cgr	Cagra.1531s0001.1	77.922	154	4	157	6.15E-81	TX	different
At_RPP5	TNL	Cgr	Cagra.1618s0019.1	84	150	11	160	4.73E-82	TX	different
At_RPP5	TNL	Cgr	Cagra.1618s0019.1	84	150	11	160	4.73E-82	TX	different
At_RAC1	TNL	Cgr	Cagra.0569s0002.1	72.085	283	553	832	7.98E-117	TNL	same
At_RAC1	TNL	Cgr	Cagra.0569s0002.1	72.085	283	553	832	7.98E-117	TNL	same
Bna_LepR3/Rlm2	LRR-RLP	Cgr	Cagra.1655s0067.1	65.266	357	599	948	5.97E-153	LRR-RLP	same
Bra_Crr1a	TNL	Cgr	Cagra.4961s0011.1	60.253	395	67	460	1.01E-161	TN	different
Bra_Crr1a	TNL	Cgr	Cagra.4961s0011.1	60.253	395	67	460	1.01E-161	TN	different
At_NGR1b	RNL	Cgr	Cagra.2007s0012.1	62.319	414	406	815	6.37E-162	NL	different
At_NGR1b	RNL	Cgr	Cagra.2007s0012.1	62.319	414	406	815	6.37E-162	NL	different
At_RLP32	LRR-RLP	Cgr	Cagra.1655s0067.1	71.307	352	512	853	3.83E-168	LRR-RLP	same
At_NGR1a	RNL	Cgr	Cagra.2007s0012.1	65.937	411	404	809	1.83E-172	NL	different
At_NGR1a	RNL	Cgr	Cagra.2007s0012.1	65.937	411	404	809	1.83E-172	NL	different
At_WRR12	TNL	Cgr	Cagra.0824s0126.1	86.807	1046	3	1048	0	TNL	same
At_WRR12	TNL	Cgr	Cagra.0824s0126.1	86.807	1046	3	1048	0	TNL	same
At_RPS2	NL	Cgr	Cagra.3467s0049.1	85.809	909	1	909	0	CNL	different
At_RPS2	NL	Cgr	Cagra.3467s0049.1	85.809	909	1	909	0	CNL	different
At_RLP32	LRR-RLP	Cgr	Cagra.2108s0001.1	85.698	853	1	852	0	LRR-RLP	same
At_RLP1	LRR-RLP	Cgr	Cagra.1671s0360.1	85	1080	1	1077	0	LRR-RLP	same
At_ADR1	NL	Cgr	Cagra.6133s0007.1	83.692	791	1	787	0	NL	same
At_ADR1	NL	Cgr	Cagra.6133s0007.1	83.692	791	1	787	0	NL	same
At_RLP30	LRR-RLP	Cgr	Cagra.2179s0052.1	83.641	758	30	786	0	LRR-RLP	same
At_NGR1a	RNL	Cgr	Cagra.2007s0011.1	82.432	814	1	809	0	CNL	different
At_NGR1a	RNL	Cgr	Cagra.2007s0011.1	82.432	814	1	809	0	CNL	different
At_RLM1a	TNL	Cgr	Cagra.0062s0032.1	77.854	1025	4	997	0	TNL	same
At_RLM1a	TNL	Cgr	Cagra.0062s0032.1	77.854	1025	4	997	0	TNL	same
At_RLP42	LRR-RLP	Cgr	Cagra.16515s0001.1	76.633	689	11	698	0	LRR-RLP	same
At_WRR4b	TNL	Cgr	Cagra.12399s0002.1	75.549	1092	11	1072	0	TNL	same
At_WRR4b	TNL	Cgr	Cagra.12399s0002.1	75.549	1092	11	1072	0	TNL	same
At_RPP7	NL	Cgr	Cagra.0550s0003.2	72.184	870	1	866	0	NL	same
At_RPP7	NL	Cgr	Cagra.0550s0003.1	72.184	870	1	866	0	NL	same
At_RPP7	NL	Cgr	Cagra.0550s0003.2	72.184	870	1	866	0	NL	same
At_RPP7	NL	Cgr	Cagra.0550s0003.1	72.184	870	1	866	0	NL	same
At_RLP23	LRR-RLP	Cgr	Cagra.0301s0115.1	70.857	875	17	890	0	LRR-RLP	same
At_RPP8	CNL	Cgr	Cagra.2383s0008.1	70.241	914	1	908	0	CNL	same
At_RPP8	CNL	Cgr	Cagra.2383s0008.1	70.241	914	1	908	0	CNL	same
At_RPP39	CNL	Cgr	Cagra.0982s0007.3	70.149	670	214	870	0	NL	different
At_RPP39	CNL	Cgr	Cagra.0982s0007.1	70.149	670	214	870	0	NL	different
At_RPP39	CNL	Cgr	Cagra.0982s0007.2	70.149	670	214	870	0	NL	different
At_RPP39	CNL	Cgr	Cagra.0982s0007.3	70.149	670	214	870	0	NL	different
At_RPP39	CNL	Cgr	Cagra.0982s0007.1	70.149	670	214	870	0	NL	different
At_RPP39	CNL	Cgr	Cagra.0982s0007.2	70.149	670	214	870	0	NL	different
At_NGR1b	RNL	Cgr	Cagra.2007s0011.1	69.315	818	5	812	0	CNL	different
At_NGR1b	RNL	Cgr	Cagra.2007s0011.1	69.315	818	5	812	0	CNL	different
Bna_LepR3/Rlm2	LRR-RLP	Cgr	Cagra.3457s0001.1	68.037	657	289	945	0	LRR-RLP	same
At_RLP42	LRR-RLP	Cgr	Cagra.0301s0115.1	66.897	867	19	880	0	LRR-RLP	same
At_RPP1	TNL	Cgr	Cagra.26171s0001.1	65.804	927	90	938	0	Other-NLR	different
At_RPP1	TNL	Cgr	Cagra.26171s0001.1	65.804	927	90	938	0	Other-NLR	different
At_RPP8	CNL	Cgr	Cagra.11315s0008.1	65.755	914	1	908	0	CNL	same
At_RPP8	CNL	Cgr	Cagra.11315s0008.1	65.755	914	1	908	0	CNL	same
At_RLP23	LRR-RLP	Cgr	Cagra.16515s0001.1	65.449	657	42	697	0	LRR-RLP	same
At_RPP1	TNL	Cgr	Cagra.6251s0001.2	64.942	1198	36	1163	0	TNL	same
At_RPP1	TNL	Cgr	Cagra.6251s0001.2	64.942	1198	36	1163	0	TNL	same
At_RPP1	TNL	Cgr	Cagra.6251s0001.1	64.804	1199	36	1163	0	TNL	same
At_RPP1	TNL	Cgr	Cagra.6251s0001.1	64.804	1199	36	1163	0	TNL	same
At_RLP32	LRR-RLP	Cgr	Cagra.3457s0001.1	64.733	655	200	854	0	LRR-RLP	same
At_RPS4	TNL	Cgr	Cagra.1562s0043.1	64.429	1147	5	1139	0	TNL	same
At_RPS4	TNL	Cgr	Cagra.1562s0043.1	64.429	1147	5	1139	0	TNL	same
At_RLM1b	TNL	Cgr	Cagra.3285s0010.1	64.411	857	162	1006	0	NL	different
At_RLM1b	TNL	Cgr	Cagra.3285s0010.1	64.411	857	162	1006	0	NL	different

At_RLM1b	TNL	Cgr	Cagra.7993s0004.1	64.377	1019	10	1009	0	TNL	same
At_RLM1b	TNL	Cgr	Cagra.7993s0004.1	64.377	1019	10	1009	0	TNL	same
At_RPS5	TNL	Cgr	Cagra.0568s0138.1	64.334	886	1	884	0	CNL	different
At_RPS5	TNL	Cgr	Cagra.0568s0138.1	64.334	886	1	884	0	CNL	different
At_WRR12	TNL	Cgr	Cagra.4715s0008.1	64.167	1027	1	1021	0	TNL	same
At_WRR12	TNL	Cgr	Cagra.4715s0008.1	64.167	1027	1	1021	0	TNL	same
At_WRR12	TNL	Cgr	Cagra.4715s0010.1	63.938	1026	1	1021	0	TNL	same
At_WRR12	TNL	Cgr	Cagra.4715s0010.1	63.938	1026	1	1021	0	TNL	same
At_RLM1b	TNL	Cgr	Cagra.7993s0001.1	63.84	802	210	1002	0	NL	different
At_RLM1b	TNL	Cgr	Cagra.7993s0001.1	63.84	802	210	1002	0	NL	different
At_WRR8	TNL	Cgr	Cagra.0569s0023.1	63.655	498	14	506	0	TN	different
At_WRR8	TNL	Cgr	Cagra.0569s0023.1	63.655	498	14	506	0	TN	different
Bra_Crr1a	TNL	Cgr	Cagra.3601s0001.1	63.579	475	148	618	0	TN	different
Bra_Crr1a	TNL	Cgr	Cagra.3601s0001.1	63.579	475	148	618	0	TN	different
At_WRR8	TNL	Cgr	Cagra.0569s0025.1	63.32	747	12	739	0	TNL	same
At_WRR8	TNL	Cgr	Cagra.0569s0025.1	63.32	747	12	739	0	TNL	same
At_WRR8	TNL	Cgr	Cagra.0569s0002.1	63.249	1151	20	1139	0	TNL	same
At_WRR8	TNL	Cgr	Cagra.0569s0002.1	63.249	1151	20	1139	0	TNL	same
At_RPP7	NL	Cgr	Cagra.6704s0008.1	63.2	750	126	869	0	NL	same
At_RPP7	NL	Cgr	Cagra.6704s0008.1	63.2	750	126	869	0	NL	same
At_RLP23	LRR-RLP	Cgr	Cagra.0301s0076.1	63.194	720	175	889	0	LRR-RLP	same
Bna_LepR3/Rlm2	LRR-RLP	Cgr	Cagra.2108s0001.1	63.151	749	204	943	0	LRR-RLP	same
At_RRS1	TNL	Cgr	Cagra.1562s0042.1	62.941	1333	8	1277	0	TNL	same
At_RRS1	TNL	Cgr	Cagra.1562s0042.1	62.941	1333	8	1277	0	TNL	same
At_RRS1	TNL	Cgr	Cagra.1562s0042.2	62.913	1332	8	1277	0	TNL	same
At_RRS1	TNL	Cgr	Cagra.1562s0042.2	62.913	1332	8	1277	0	TNL	same
Bju_WRR1	CNL	Cgr	Cagra.2383s0008.1	62.816	909	1	900	0	CNL	same
Bju_WRR1	CNL	Cgr	Cagra.2383s0008.1	62.816	909	1	900	0	CNL	same
At_RLP32	LRR-RLP	Cgr	Cagra.8062s0003.1	62.784	747	110	852	0	LRR-RLP	same
At_RPP8	CNL	Cgr	Cagra.0053s0020.1	62.772	873	1	867	0	NL	different
At_RPP8	CNL	Cgr	Cagra.0053s0020.1	62.772	873	1	867	0	NL	different
At_RLP23	LRR-RLP	Cgr	Cagra.0301s0053.1	62.762	717	175	890	0	LRR-RLP	same
At_RAC1	TNL	Cgr	Cagra.0569s0023.1	62.675	501	3	497	0	TN	different
At_RAC1	TNL	Cgr	Cagra.0569s0023.1	62.675	501	3	497	0	TN	different
At_ADR1	NL	Cgr	Cagra.5249s0010.1	62.562	812	6	787	0	NL	same
At_ADR1	NL	Cgr	Cagra.5249s0010.1	62.562	812	6	787	0	NL	same
At_RPP8	CNL	Cgr	Cagra.0053s0020.2	62.543	873	1	867	0	NL	different
At_RPP8	CNL	Cgr	Cagra.0053s0020.2	62.543	873	1	867	0	NL	different
At_RLP42	LRR-RLP	Cgr	Cagra.0301s0053.1	62.482	701	182	878	0	LRR-RLP	same
At_RLP32	LRR-RLP	Cgr	Cagra.1655s0060.1	62.225	908	1	850	0	LRR-RLP	same
At_RLP42	LRR-RLP	Cgr	Cagra.0301s0076.1	61.986	705	182	878	0	LRR-RLP	same
At_RLM1b	TNL	Cgr	Cagra.7993s0003.1	61.919	1011	1	998	0	TNL	same
At_RLM1b	TNL	Cgr	Cagra.7993s0003.1	61.919	1011	1	998	0	TNL	same
At_WRR8	TNL	Cgr	Cagra.3954s0001.1	61.84	739	17	745	0	TNL	same
At_WRR8	TNL	Cgr	Cagra.3954s0001.2	61.84	739	17	745	0	TNL	same
At_WRR8	TNL	Cgr	Cagra.3954s0001.1	61.84	739	17	745	0	TNL	same
At_WRR8	TNL	Cgr	Cagra.3954s0001.2	61.84	739	17	745	0	TNL	same
At_RLP23	LRR-RLP	Cgr	Cagra.0301s0053.2	61.714	525	175	698	0	LRR-RLP	same
At_RAC1	TNL	Cgr	Cagra.3954s0001.1	61.456	742	6	735	0	TNL	same
At_RAC1	TNL	Cgr	Cagra.3954s0001.1	61.456	742	6	735	0	TNL	same
At_WRR4b	TNL	Cgr	Cagra.12399s0001.1	61.41	1078	3	1064	0	TNL	same
At_WRR4b	TNL	Cgr	Cagra.12399s0001.1	61.41	1078	3	1064	0	TNL	same
At_WRR4a	TNL	Cgr	Cagra.12399s0001.1	61.377	1002	1	998	0	TNL	same
At_WRR4a	TNL	Cgr	Cagra.12399s0001.1	61.377	1002	1	998	0	TNL	same
At_RLP42	LRR-RLP	Cgr	Cagra.0301s0053.2	61.079	519	182	696	0	LRR-RLP	same
Bna_LepR3/Rlm2	LRR-RLP	Cgr	Cagra.8062s0003.1	60.963	789	156	937	0	LRR-RLP	same
At_RAC1	TNL	Cgr	Cagra.0569s0025.1	60.705	766	1	741	0	TNL	same
At_RAC1	TNL	Cgr	Cagra.0569s0025.1	60.705	766	1	741	0	TNL	same
At_WRR4a	TNL	Cgr	Cagra.12399s0002.1	60.624	1026	9	1004	0	TNL	same
At_WRR4a	TNL	Cgr	Cagra.12399s0002.1	60.624	1026	9	1004	0	TNL	same
Bra_Crr1a	TNL	Cgr	Cagra.1183s0005.1	60.482	706	85	782	0	TNL	same
Bra_Crr1a	TNL	Cgr	Cagra.1183s0005.1	60.482	706	85	782	0	TNL	same
At_RPP39	CNL	Bcr	KAF3519034.1	60.265	151	385	529	1.75E-50	NL	different
At_WRR9	NL	Bcr	KAF3575596.1	63.19	163	106	265	2.94E-58	TX	different
At_RPP2a	TNL	Bcr	KAF3531620.1	63.128	179	206	383	1.25E-58	NL	different
At_WRR8	TNL	Bcr	KAF3567523.1	61.875	160	354	511	4.35E-59	NBS	different
At_RPP5	TNL	Bcr	KAF3531620.1	61.957	184	211	394	2.76E-59	NL	different
At_RPM1	NL	Bcr	KAF3528544.1	60.104	193	468	656	4.16E-62	LRR-RLP	different
At_WRR4b	TNL	Bcr	KAF3575596.1	66.258	163	103	262	4.87E-64	TX	different
At_WRR4a	TNL	Bcr	KAF3564882.1	68.354	158	1	157	9.28E-66	TX	different
At_RLM1a	TNL	Bcr	KAF3575596.1	66.258	163	102	261	2.31E-67	TX	different
At_RLP42	LRR-RLP	Bcr	KAF3578456.1	77.632	152	725	876	1.36E-67	LRR-RLP	same
At_RLP23	LRR-RLP	Bcr	KAF3578456.1	73.494	166	725	890	4.34E-68	LRR-RLP	same
Bol_FocBo1	TNL	Bcr	KAF3531620.1	66.484	182	206	386	2.23E-68	NL	different
At_RLM1b	TNL	Bcr	KAF3575596.1	69.136	162	102	260	1.21E-68	TX	different
At_RLM1a	TNL	Bcr	KAF3564882.1	68.354	158	1	158	5.59E-69	TX	different
At_WRR9	NL	Bcr	KAF3564882.1	69.565	161	2	162	3.42E-69	TX	different
At_RPP13	CNL	Bcr	KAF3527235.1	75.316	158	148	304	3.25E-70	NL	different
At_RRS1	TNL	Bcr	KAF3564691.1	69.892	186	149	331	9.01E-72	NL	different
At_WRR4b	TNL	Bcr	KAF3564882.1	72.956	159	1	159	5.90E-73	TX	different
At_RPS5	TNL	Bcr	KAF3517781.1	64.767	193	1	193	7.11E-75	CN	different
At_RLM1b	TNL	Bcr	KAF3564882.1	77.848	158	1	158	1.12E-77	TX	different
At_RLP30	LRR-RLP	Bcr	KAF3517872.1	66.667	198	580	777	2.59E-80	LRR-RLP	same
At_RLP1	LRR-RLP	Bcr	KAF3544388.1	68.182	198	880	1077	1.90E-81	LRR-RLP	same
At_RPP2b	TNL	Bcr	KAF3532444.1	64.167	240	121	360	2.51E-84	Other-NLR	different
Bra_Crr1a	TNL	Bcr	KAF3550114.1	60.311	257	215	471	2.37E-85	NBS	different

Bna_LepR3/Rlm2	LRR-RLP	Bcr	KAF3548035.1	69.898	196	738	932	7.65E-89	LRR-RLP	same
At_RAC1	TNL	Bcr	KAF3567662.1	65.401	237	160	396	1.43E-89	NL	different
At_WRR8	TNL	Bcr	KAF3567662.1	66.087	230	170	399	1.14E-91	NL	different
At_RFO3	Other-RLK	Bcr	KAF3534109.1	60.345	232	515	745	9.57E-92	Other-RLK	same
Bna_LepR3/Rlm2	LRR-RLP	Bcr	KAF3551131.1	67.265	223	731	950	1.45E-96	LRR-RLP	same
At_RLP32	LRR-RLP	Bcr	KAF3548035.1	75.243	206	649	852	1.96E-98	LRR-RLP	same
At_RPP2a	TNL	Bcr	KAF3596196.1	65.504	258	157	414	1.52E-98	NL	different
At_RPM1	NL	Bcr	KAF3528543.1	83.06	183	132	313	1.15E-98	NBS	different
At_RLP32	LRR-RLP	Bcr	KAF3551131.1	70.852	223	648	868	1.51E-103	LRR-RLP	same
Bna_Rlm9/4/7	Other-RLK	Bcr	KAF3518354.1	67.537	268	97	361	3.66E-106	Other-RLK	same
At_RPP2a	TNL	Bcr	KAF3578478.1	78.39	236	1065	1299	2.12E-106	TNL	same
At_RPP2a	TNL	Bcr	KAF3596191.1	65.799	269	147	414	6.88E-111	NL	different
At_RLP42	LRR-RLP	Bcr	KAF3551685.1	66.066	333	544	875	2.40E-134	LRR-RLP	same
At_RFO1	Other-RLK	Bcr	KAF3518354.1	67.879	330	433	750	1.66E-138	Other-RLK	same
At_RLP23	LRR-RLP	Bcr	KAF3551685.1	67.976	331	544	874	2.26E-141	LRR-RLP	same
At_NGR1b	RNL	Bcr	KAF3562989.1	60.766	418	406	815	2.58E-149	CNL	different
At_RLP1	LRR-RLP	Bcr	KAF3497647.1	63.728	397	567	959	1.44E-154	LRR-RLP	same
At_RFO1	Other-RLK	Bcr	KAF3611901.1	64.602	339	326	662	3.46E-155	Other-RLK	same
At_PBS1	Other-RLK	Bcr	KAF3550268.1	63.235	340	71	408	4.37E-156	Other-RLK	same
Bra_Crr1a	TNL	Bcr	KAF3611543.1	64.456	377	67	441	3.64E-159	TN	different
At_NGR1a	RNL	Bcr	KAF3562989.1	62.884	423	396	809	1.79E-159	CNL	different
At_RPP1	TNL	Bcr	KAF3611543.1	61.905	378	94	469	3.11E-161	TN	different
At_NGR1b	RNL	Bcr	KAF3597564.1	63.814	409	423	800	9.24E-162	CNL	different
At_NGR1a	RNL	Bcr	KAF3597564.1	64.461	408	421	794	3.56E-162	CNL	different
Bna_Rlm9/4/7	Other-RLK	Bcr	KAF3611901.1	69.806	361	344	695	1.82E-169	Other-RLK	same
At_RFO1	Other-RLK	Bcr	KAF3518354.1	64.198	405	355	747	7.15E-170	Other-RLK	same
Bra_cRa/cRb	TNL	Bcr	KAF3611543.1	60.465	430	74	501	2.18E-170	TN	different
At_NGR1b	RNL	Bcr	KAF3562989.1	67.133	429	192	593	3.91E-174	CNL	different
Bra_Crr1a	TNL	Bcr	KAF3611543.1	64.12	432	67	496	8.17E-178	TN	different
Bol_FocBo1	TNL	Bcr	KAF3596191.1	98.897	816	146	961	0	NL	different
Bna_Rlm9/4/7	Other-RLK	Bcr	KAF3518354.1	96.979	331	464	794	0	Other-RLK	same
At_BAK1	LRR-RLK	Bcr	KAF3593886.1	94.976	617	1	615	0	LRR-RLK	same
At_BAK1	LRR-RLK	Bcr	KAF3542856.1	94.595	518	98	615	0	LRR-RLK	same
Bna_Rlm9/4/7	Other-RLK	Bcr	KAF3518354.1	89.731	409	384	792	0	Other-RLK	same
Bna_LepR3/Rlm2	LRR-RLP	Bcr	KAF3565783.1	87.762	572	379	950	0	LRR-RLP	same
At_RPS2	NL	Bcr	KAF3531089.1	83.956	910	1	909	0	CNL	different
Bju_WRR1	CNL	Bcr	KAF3578869.1	82.834	501	406	904	0	NL	different
At_SOBIR1	LRR-RLK	Bcr	KAF3549886.1	80.989	647	1	641	0	LRR-RLK	same
At_SOBIR1	LRR-RLK	Bcr	KAF3576067.1	80.871	643	1	641	0	LRR-RLK	same
At_RFO1	Other-RLK	Bcr	KAF3516545.1	80.531	452	27	470	0	Other-RLK	same
At_FLS2	LRR-RLK	Bcr	KAF3565146.1	80.102	1176	1	1173	0	LRR-RLK	same
At_RFO2	LRR-RLP	Bcr	KAF3495711.1	79.383	519	222	740	0	LRR-RLP	same
At_BAK1	LRR-RLK	Bcr	KAF3516952.1	79.333	600	30	615	0	LRR-RLK	same
At_RPM1	NL	Bcr	KAF3528549.1	79.211	938	1	926	0	NL	same
At_BAK1	LRR-RLK	Bcr	KAF3520244.1	79	600	30	615	0	LRR-RLK	same
Bna_Rlm9/4/7	Other-RLK	Bcr	KAF3518354.1	78.995	657	1	655	0	Other-RLK	same
At_BAK1	LRR-RLK	Bcr	KAF3597752.1	78.824	595	22	615	0	LRR-RLK	same
At_RPP2b	TNL	Bcr	KAF3532444.1	78.493	730	465	1193	0	Other-NLR	different
At_RPS5	TNL	Bcr	KAF3496083.1	78.356	730	1	730	0	NL	different
At_BAK1	LRR-RLK	Bcr	KAF3543401.1	77.796	608	10	615	0	LRR-RLK	same
At_WRR12	TNL	Bcr	KAF3498111.1	77.732	723	340	1049	0	NL	different
At_NGR1a	RNL	Bcr	KAF3562989.1	76.765	779	1	771	0	CNL	different
At_SOBIR1	LRR-RLK	Bcr	KAF3576064.1	76.361	643	1	641	0	LRR-RLK	same
At_RPS4	TNL	Bcr	KAF3564697.1	76.096	753	441	1192	0	NL	different
At_NGR1a	RNL	Bcr	KAF3562989.1	75.878	427	190	587	0	CNL	different
At_RFO3	Other-RLK	Bcr	KAF3609633.1	75.846	857	18	850	0	Other-RLK	same
At_RPP2a	TNL	Bcr	KAF3532444.1	72.937	824	479	1300	0	Other-NLR	different
At_RPP2b	TNL	Bcr	KAF3593075.1	71.784	1166	13	1175	0	TNL	same
At_RLP23	LRR-RLP	Bcr	KAF3575513.1	68.694	888	1	882	0	LRR-RLP	same
Bra_Crr1a	TNL	Bcr	KAF3544173.1	68.598	656	220	874	0	NL	different
At_RLP23	LRR-RLP	Bcr	KAF3580346.1	68.127	822	1	820	0	LRR-RLP	same
At_RLP42	LRR-RLP	Bcr	KAF3580346.1	68	825	1	822	0	LRR-RLP	same
At_RLP42	LRR-RLP	Bcr	KAF3575513.1	67.95	883	1	876	0	LRR-RLP	same
Bna_MPK9	Other-RLK	Bcr	KAF3609513.1	67.925	583	55	591	0	Other-RLK	same
At_RFO2	LRR-RLP	Bcr	KAF3605889.1	67.806	702	41	740	0	LRR-RLP	same
At_RFO2	LRR-RLP	Bcr	KAF3518269.1	67.733	719	20	737	0	LRR-RLK	different
Bna_Rlm9/4/7	Other-RLK	Bcr	KAF3516545.1	67.651	779	25	794	0	Other-RLK	same
At_RFO2	LRR-RLP	Bcr	KAF3497769.1	67.651	711	29	737	0	LRR-RLP	same
At_RPP2a	TNL	Bcr	KAF3528035.1	67.545	835	472	1300	0	TNL	same
At_RLM1b	TNL	Bcr	KAF3606819.1	66.197	852	164	1005	0	NL	different
At_RFO1	Other-RLK	Bcr	KAF3498694.1	65.931	725	7	719	0	Other-RLK	same
At_RLP23	LRR-RLP	Bcr	KAF3576309.1	65.576	703	182	881	0	LRR-RLP	same
Bju_WRR1	CNL	Bcr	KAF3596471.1	65.533	647	285	901	0	CNL	same
At_NGR1b	RNL	Bcr	KAF3562989.1	65.184	787	3	777	0	CNL	different
At_RLP32	LRR-RLP	Bcr	KAF3532641.1	64.56	728	114	840	0	LRR-RLP	same
At_RPP2b	TNL	Bcr	KAF3578478.1	64.531	1108	134	1193	0	TNL	same
At_RLP32	LRR-RLP	Bcr	KAF3532638.1	64.459	740	114	852	0	LRR-RLP	same
At_RLP32	LRR-RLP	Bcr	KAF3565783.1	64.374	567	292	853	0	LRR-RLP	same
At_RLP1	LRR-RLP	Bcr	KAF3497651.1	64.326	527	559	1076	0	LRR-RLP	same
At_RPS5	TNL	Bcr	KAF3611428.1	64.129	591	1	590	0	CNL	different
At_RFO1	Other-RLK	Bcr	KAF3498700.1	64.095	713	1	696	0	Other-RLK	same
Bna_Rlm9/4/7	Other-RLK	Bcr	KAF3549501.1	63.359	786	21	793	0	Other-RLK	same
At_RFO3	Other-RLK	Bcr	KAF3544714.1	63.14	879	74	850	0	Other-RLK	same
At_RLP42	LRR-RLP	Bcr	KAF3576309.1	62.817	710	182	888	0	LRR-RLP	same
At_RLP30	LRR-RLP	Bcr	KAF3546057.1	62.252	453	323	772	0	LRR-RLP	same
At_RPP13	CNL	Bcr	KAF3560565.1	61.687	569	205	755	0	NL	different

At_RFO1	Other-RLK	Bcr	KAF3498693.1	61.518	764	1	751	0	Other-RLK	same
Bra_Crr1a	TNL	Bcr	KAF3542303.1	61.139	790	220	975	0	NL	different
At_RLM1b	TNL	Bcr	KAF3564885.1	61.136	669	347	999	0	NL	different
Bju_WRR1	CNL	Bcr	KAF3596471.1	60.976	492	1	471	0	CNL	same
Bna_LepR3/Rlm2	LRR-RLP	Bcr	KAF3532638.1	60.761	762	176	937	0	LRR-RLP	same
Bna_LepR3/Rlm2	LRR-RLP	Bcr	KAF3532641.1	60.618	518	201	718	0	LRR-RLP	same
Bna_LepR3/Rlm2	LRR-RLP	Bcr	KAF3532641.1	60.54	778	152	927	0	LRR-RLP	same
At_RPS5	TNL	Bcr	KAF3498255.1	60.36	888	1	883	0	NL	different
At_RPS5	TNL	Bcr	KAF3497592.1	60.289	554	1	552	0	CNL	different
At_RPP7	NL	Cbp	Cbp19077	62.5	152	575	723	3.63E-48	NL	same
Bol_FocBo1	TNL	Cbp	Cbp34462	62.162	148	7	154	2.79E-50	TNL	same
At_RAC1	TNL	Cbp	Cbp34462	66.443	149	9	154	8.85E-54	TNL	same
At_WRR8	TNL	Cbp	Cbp34462	67.114	149	19	164	8.41E-57	TNL	same
At_RPP13	CNL	Cbp	Cbp11721	60.406	197	646	834	1.03E-60	NL	different
At_RPP7	NL	Cbp	Cbp13283	65.294	170	1	169	4.68E-62	NBS	different
At_RFO3	Other-RLK	Cbp	Cbp14207	60.326	184	510	693	1.24E-70	Other-RLK	same
At_RLP30	LRR-RLP	Cbp	Cbp2531	66.279	172	604	775	1.69E-74	LRR-RLP	same
Bol_FocBo1	TNL	Cbp	Cbp34461	60.256	234	5	238	4.66E-80	TNL	same
At_RAC1	TNL	Cbp	Cbp34461	68.201	239	3	238	4.93E-92	TNL	same
At_RFO3	Other-RLK	Cbp	Cbp22874	60.606	231	515	745	2.03E-93	Other-RLK	same
At_WRR8	TNL	Cbp	Cbp34461	67.47	249	9	254	1.74E-97	TNL	same
At_RPP1	TNL	Cbp	Cbp13366	63.306	248	94	341	4.55E-106	TN	different
At_WRR4b	TNL	Cbp	Cbp19430	63.424	257	14	269	7.98E-109	TN	different
At_RLM1b	TNL	Cbp	Cbp19430	64.591	257	13	268	3.48E-109	TN	different
At_NGR1b	RNL	Cbp	Cbp33510	64.982	277	539	815	3.17E-109	NL	different
At_RPP2a	TNL	Cbp	Cbp10076	60.692	318	98	414	5.46E-111	TNL	same
At_RLM1a	TNL	Cbp	Cbp19430	64.202	257	13	268	1.78E-111	TN	different
At_NGR1a	RNL	Cbp	Cbp33510	66.787	277	533	809	2.95E-112	NL	different
At_RPP2a	TNL	Cbp	Cbp2517	61.635	318	98	414	6.88E-114	TNL	same
At_NGR1b	RNL	Cbp	Cbp28794	66.426	277	539	815	1.48E-118	NL	different
At_NGR1a	RNL	Cbp	Cbp28794	67.509	277	533	809	1.01E-119	NL	different
Bju_WRR1	CNL	Cbp	Cbp47817	61.728	324	1	312	1.23E-127	CN	different
Bju_WRR1	CNL	Cbp	Cbp47816	61.728	324	1	312	1.23E-127	CN	different
At_RLP30	LRR-RLP	Cbp	Cbp5067	78.626	262	15	273	1.22E-130	LRR-RLP	same
At_WRR9	NL	Cbp	Cbp13417	60.247	405	13	416	5.36E-147	Other-NLR	different
At_RLM3	TN	Cbp	Cbp49279	60.448	402	58	450	6.31E-155	TNL	different
At_RLM3	TN	Cbp	Cbp45527	60.448	402	58	450	6.31E-155	TNL	different
At_RPP8	CNL	Cbp	Cbp47817	75.817	306	1	306	1.07E-155	CN	different
At_RPP8	CNL	Cbp	Cbp47816	75.817	306	1	306	1.07E-155	CN	different
At_RPP2a	TNL	Cbp	Cbp49279	68.245	359	56	414	4.87E-159	TNL	same
At_RPP2a	TNL	Cbp	Cbp45527	68.245	359	56	414	4.87E-159	TNL	same
Bna_Rlm9/4/7	Other-RLK	Cbp	Cbp23249	69.591	342	395	735	6.89E-165	Other-RLK	same
At_RLM1b	TNL	Cbp	Cbp13417	62.651	415	10	424	1.40E-166	Other-NLR	different
Bna_Rlm9/4/7	Other-RLK	Cbp	Cbp41731	69.477	344	395	737	2.06E-167	Other-RLK	same
At_RLM1a	TNL	Cbp	Cbp13417	63.438	413	12	424	3.49E-168	Other-NLR	different
At_RPP13	CNL	Cbp	Cbp11721	70.492	366	227	574	1.88E-171	NL	different
At_WRR4b	TNL	Cbp	Cbp13132	61.268	426	63	485	6.06E-173	TNL	same
At_WRR4a	TNL	Cbp	Cbp13417	65.7	414	8	421	7.03E-178	Other-NLR	different
At_BAK1	LRR-RLK	Cbp	Cbp38634	98.537	615	1	615	0	LRR-RLK	same
At_FLS2	LRR-RLK	Cbp	Cbp34449	89.316	1170	1	1167	0	LRR-RLK	same
At_SOBIR1	LRR-RLK	Cbp	Cbp6725	88.509	644	1	641	0	LRR-RLK	same
At_FLS2	LRR-RLK	Cbp	Cbp26999	88.13	1171	1	1167	0	LRR-RLK	same
At_RFO1	Other-RLK	Cbp	Cbp27992	87.203	758	1	751	0	Other-RLK	same
At_RFO1	Other-RLK	Cbp	Cbp9961	86.508	756	1	751	0	Other-RLK	same
At_RLP1	LRR-RLP	Cbp	Cbp34170	86.124	1081	1	1077	0	LRR-RLP	same
At_RLP1	LRR-RLP	Cbp	Cbp36183	85.939	1081	1	1077	0	LRR-RLP	same
At_RLP1	LRR-RLP	Cbp	Cbp28518	85.939	1081	1	1077	0	LRR-RLP	same
At_RPS2	NL	Cbp	Cbp37986	85.919	909	1	909	0	NL	same
At_RPS2	NL	Cbp	Cbp5868	85.699	909	1	909	0	NL	same
At_RLP32	LRR-RLP	Cbp	Cbp9533	84.517	859	1	852	0	LRR-RLP	same
At_RLP32	LRR-RLP	Cbp	Cbp7339	84.517	859	1	852	0	LRR-RLP	same
At_RPM1	NL	Cbp	Cbp31703	84.499	929	1	926	0	NL	same
At_RLP32	LRR-RLP	Cbp	Cbp5035	84.465	869	1	867	0	LRR-RLP	same
At_RLP30	LRR-RLP	Cbp	Cbp9505	84.037	758	30	786	0	LRR-RLP	same
At_ADR1	NL	Cbp	Cbp47963	83.312	791	1	787	0	NL	same
At_ADR1	NL	Cbp	Cbp14253	83.312	791	1	787	0	NL	same
At_ADR1	NL	Cbp	Cbp47529	83.059	791	1	787	0	NL	same
At_NGR1a	RNL	Cbp	Cbp28795	82.432	814	1	809	0	CNL	different
At_NGR1a	RNL	Cbp	Cbp33511	81.818	814	1	809	0	CNL	different
At_BAK1	LRR-RLK	Cbp	Cbp20587	81.489	470	146	615	0	LRR-RLK	same
At_BAK1	LRR-RLK	Cbp	Cbp116	81.489	470	146	615	0	LRR-RLK	same
Bna_MPK9	Other-RLK	Cbp	Cbp28457	80.723	332	73	402	0	Other-RLK	same
At_BAK1	LRR-RLK	Cbp	Cbp40474	80.693	606	13	615	0	LRR-RLK	same
At_RPP8	CNL	Cbp	Cbp8106	80.176	908	1	907	0	CNL	same
At_BAK1	LRR-RLK	Cbp	Cbp14007	79.865	591	30	607	0	LRR-RLK	same
At_BAK1	LRR-RLK	Cbp	Cbp14006	79.865	591	30	607	0	LRR-RLK	same
At_BAK1	LRR-RLK	Cbp	Cbp47224	79.504	605	24	615	0	LRR-RLK	same
At_RLP42	LRR-RLP	Cbp	Cbp45770	79.064	855	24	877	0	LRR-RLP	same
At_RFO3	Other-RLK	Cbp	Cbp19547	78.356	864	1	850	0	Other-RLK	same
At_RLP42	LRR-RLP	Cbp	Cbp36944	78.207	881	1	880	0	LRR-RLP	same
At_RLP42	LRR-RLP	Cbp	Cbp36941	78.19	862	17	877	0	LRR-RLP	same
At_RLP42	LRR-RLP	Cbp	Cbp36938	77.929	879	1	877	0	LRR-RLP	same
At_RLP42	LRR-RLP	Cbp	Cbp45769	77.929	879	1	877	0	LRR-RLP	same
At_RLP42	LRR-RLP	Cbp	Cbp36940	77.378	862	17	877	0	LRR-RLP	same
At_WRR4b	TNL	Cbp	Cbp19202	76.562	1088	11	1091	0	TNL	same
At_RLP23	LRR-RLP	Cbp	Cbp1801	75.754	796	95	890	0	LRR-RLP	same

At_WRR4b	TNL	Cbp	Cbp13410	75.678	1069	11	1072	0	TNL	same
At_RFO1	Other-RLK	Cbp	Cbp41731	73.913	345	364	707	0	Other-RLK	same
At_WRR9	NL	Cbp	Cbp14210	73.433	1005	4	1002	0	TNL	different
At_RFO1	Other-RLK	Cbp	Cbp23249	73.333	345	364	707	0	Other-RLK	same
At_RPP39	CNL	Cbp	Cbp33720	72.547	856	22	870	0	CNL	same
At_RPP39	CNL	Cbp	Cbp16103	71.971	842	42	870	0	CNL	same
At_RLM1a	TNL	Cbp	Cbp9286	71.945	1023	4	997	0	TNL	same
At_RPP39	CNL	Cbp	Cbp33711	71.833	884	1	870	0	CNL	same
At_RPP39	CNL	Cbp	Cbp46917	71.38	884	1	870	0	CNL	same
At_RPP7	NL	Cbp	Cbp19065	71.038	877	1	866	0	NL	same
At_RPP13	CNL	Cbp	Cbp39590	70.839	799	40	826	0	NL	different
At_RFO2	LRR-RLP	Cbp	Cbp50262	70.596	721	19	737	0	LRR-RLK	different
Bna_Rlm9/4/7	Other-RLK	Cbp	Cbp19342	70.565	744	24	756	0	Other-RLK	same
At_RPP8	CNL	Cbp	Cbp51186	70.556	917	1	907	0	CNL	same
At_RPP8	CNL	Cbp	Cbp51187	70.556	917	1	907	0	CNL	same
At_RLP23	LRR-RLP	Cbp	Cbp6654	70.514	875	17	890	0	LRR-RLP	same
At_RFO2	LRR-RLP	Cbp	Cbp47177	70.411	730	13	737	0	LRR-RLK	different
At_RLM1b	TNL	Cbp	Cbp42255	70.397	831	1	827	0	TNL	same
At_WRR4b	TNL	Cbp	Cbp13417	70.388	412	13	424	0	Other-NLR	different
At_RPP13	CNL	Cbp	Cbp39607	70.035	861	1	830	0	NL	different
At_RPP39	CNL	Cbp	Cbp33710	69.839	746	146	877	0	NL	different
At_WRR9	NL	Cbp	Cbp9219	69.544	1008	4	1002	0	TNL	different
Bna_Rlm9/4/7	Other-RLK	Cbp	Cbp3356	69.4	683	1	680	0	Other-RLK	same
At_NGR1b	RNL	Cbp	Cbp28795	69.315	818	5	812	0	CNL	different
At_NGR1b	RNL	Cbp	Cbp33511	68.949	818	5	812	0	CNL	different
Bna_Rlm9/4/7	Other-RLK	Cbp	Cbp13171	68.455	783	24	794	0	Other-RLK	same
Bna_Rlm9/4/7	Other-RLK	Cbp	Cbp3357	68.356	809	4	794	0	Other-RLK	same
At_RLM1b	TNL	Cbp	Cbp9286	68.288	473	5	469	0	TNL	same
At_RPP8	CNL	Cbp	Cbp46346	68.271	914	1	908	0	CNL	same
At_RPP39	CNL	Cbp	Cbp46906	68.212	604	121	719	0	NL	different
Bna_Rlm9/4/7	Other-RLK	Cbp	Cbp25638	68.129	775	29	794	0	Other-RLK	same
At_RLP23	LRR-RLP	Cbp	Cbp45770	67.757	856	25	879	0	LRR-RLP	same
At_RPP8	CNL	Cbp	Cbp53094	67.724	914	1	908	0	CNL	same
Bol_FocBo1	TNL	Cbp	Cbp49279	67.208	924	9	928	0	TNL	same
Bol_FocBo1	TNL	Cbp	Cbp45527	67.208	924	9	928	0	TNL	same
At_RLP23	LRR-RLP	Cbp	Cbp36940	66.744	863	18	879	0	LRR-RLP	same
At_RLP42	LRR-RLP	Cbp	Cbp6654	66.667	867	19	880	0	LRR-RLP	same
Bra_Crr1a	TNL	Cbp	Cbp3036	66.667	522	68	585	0	TN	different
At_RLP23	LRR-RLP	Cbp	Cbp45769	66.514	872	18	887	0	LRR-RLP	same
At_RLP23	LRR-RLP	Cbp	Cbp36944	66.441	888	1	887	0	LRR-RLP	same
At_RLM1b	TNL	Cbp	Cbp9263	66.37	1011	1	998	0	TNL	same
At_RPP1	TNL	Cbp	Cbp39404	65.97	1196	1	1163	0	TNL	same
At_RPP39	CNL	Cbp	Cbp33712	65.863	539	359	870	0	NL	different
At_RLM1a	TNL	Cbp	Cbp24904	65.857	659	44	701	0	TNL	same
At_RPP39	CNL	Cbp	Cbp46916	65.677	539	359	870	0	NL	different
At_RPP1	TNL	Cbp	Cbp39399	65.665	932	248	1170	0	NL	different
At_RPP8	CNL	Cbp	Cbp19189	65.646	914	1	908	0	CNL	same
At_RLP42	LRR-RLP	Cbp	Cbp1801	65.57	790	95	880	0	LRR-RLP	same
At_RPP7	NL	Cbp	Cbp19079	65.448	547	1	545	0	NBS	different
At_RPS4	TNL	Cbp	Cbp29527	65.381	1141	11	1139	0	TNL	same
At_RPS4	TNL	Cbp	Cbp26861	65.381	1141	11	1139	0	TNL	same
At_RLP23	LRR-RLP	Cbp	Cbp36938	65.354	889	1	887	0	LRR-RLP	same
At_RPP7	NL	Cbp	Cbp13280	65.265	547	1	545	0	NBS	different
At_RFO1	Other-RLK	Cbp	Cbp41733	64.993	737	1	718	0	Other-RLK	same
At_WRR12	TNL	Cbp	Cbp48776	64.819	992	1	989	0	TNL	same
At_WRR12	TNL	Cbp	Cbp52775	64.819	992	1	989	0	TNL	same
At_RPS4	TNL	Cbp	Cbp45533	64.778	1147	5	1139	0	TNL	same
At_WRR12	TNL	Cbp	Cbp48777	64.563	1030	1	1021	0	TNL	same
At_WRR12	TNL	Cbp	Cbp52774	64.563	1030	1	1021	0	TNL	same
At_RLP23	LRR-RLP	Cbp	Cbp36941	64.524	871	18	887	0	LRR-RLP	same
At_RPP1	TNL	Cbp	Cbp11528	64.502	1124	70	1163	0	TNL	same
At_WRR12	TNL	Cbp	Cbp47916	64.039	1015	12	1021	0	TNL	same
At_WRR12	TNL	Cbp	Cbp47917	64.016	1031	1	1021	0	TNL	same
At_RPS5	TNL	Cbp	Cbp51318	63.995	886	1	884	0	CNL	different
At_RPP1	TNL	Cbp	Cbp39406	63.949	1104	125	1163	0	TNL	same
At_RFO1	Other-RLK	Cbp	Cbp23247	63.946	735	1	718	0	Other-RLK	same
At_RLP23	LRR-RLP	Cbp	Cbp1830	63.75	720	175	889	0	LRR-RLP	same
At_RPP1	TNL	Cbp	Cbp11527	63.71	992	94	1033	0	TNL	same
Bna_LepR3/Rlm2	LRR-RLP	Cbp	Cbp5035	63.709	755	204	949	0	LRR-RLP	same
At_RLM1b	TNL	Cbp	Cbp24904	63.554	664	44	706	0	TNL	same
At_RPS5	TNL	Cbp	Cbp47695	63.431	886	1	884	0	CNL	different
Bna_LepR3/Rlm2	LRR-RLP	Cbp	Cbp40390	63.283	926	27	948	0	LRR-RLP	same
At_WRR4b	TNL	Cbp	Cbp9286	63.265	490	5	484	0	TNL	same
At_RLP23	LRR-RLP	Cbp	Cbp6620	63.194	720	175	889	0	LRR-RLP	same
At_ADR1	NL	Cbp	Cbp16664	63.054	812	6	787	0	NL	same
At_RRS1	TNL	Cbp	Cbp45534	63.016	1333	8	1277	0	NL	different
At_RRS1	TNL	Cbp	Cbp26860	62.941	1333	8	1277	0	TNL	same
At_RFO1	Other-RLK	Cbp	Cbp23248	62.905	771	14	751	0	Other-RLK	same
At_WRR4b	TNL	Cbp	Cbp9266	62.709	1019	1	1004	0	Other-NLR	different
At_WRR9	NL	Cbp	Cbp42255	62.696	831	4	832	0	TNL	different
At_ADR1	NL	Cbp	Cbp17170	62.685	812	6	787	0	NL	same
At_RPP1	TNL	Cbp	Cbp11526	62.585	1176	24	1163	0	Other-NLR	different
At_RLM1a	TNL	Cbp	Cbp25968	62.574	847	164	997	0	NL	different
At_RLP32	LRR-RLP	Cbp	Cbp20439	62.225	908	1	850	0	LRR-RLP	same
At_RFO1	Other-RLK	Cbp	Cbp41732	62.194	775	10	751	0	Other-RLK	same
At_RLP32	LRR-RLP	Cbp	Cbp20445	62.115	908	1	853	0	LRR-RLP	same

At_WRR4a	TNL	Cbp	Cbp19202	62.076	1002	9	1004	0	TNL	same
At_WRR4b	TNL	Cbp	Cbp13411	62.075	1060	3	1058	0	TNL	same
Bna_LepR3/Rlm2	LRR-RLP	Cbp	Cbp9533	62.037	756	204	943	0	LRR-RLP	same
Bna_LepR3/Rlm2	LRR-RLP	Cbp	Cbp7339	62.037	756	204	943	0	LRR-RLP	same
At_RLP32	LRR-RLP	Cbp	Cbp15925	61.894	908	1	850	0	LRR-RLP	same
Bju_WRR1	CNL	Cbp	Cbp8106	61.623	912	1	900	0	CNL	same
At_RLP42	LRR-RLP	Cbp	Cbp1830	61.581	721	182	889	0	LRR-RLP	same
At_RLP42	LRR-RLP	Cbp	Cbp6620	61.581	721	182	889	0	LRR-RLP	same
At_WRR4b	TNL	Cbp	Cbp24904	61.573	661	45	703	0	TNL	same
At_WRR4a	TNL	Cbp	Cbp19203	61.531	993	9	1000	0	TNL	same
At_WRR4b	TNL	Cbp	Cbp19203	61.438	1071	11	1058	0	TNL	same
At_RLP42	LRR-RLP	Cbp	Cbp36942	61.31	641	60	644	0	LRR-RLP	same
At_RLM1b	TNL	Cbp	Cbp14210	61.22	1016	1	997	0	TNL	same
At_RPP7	NL	Cbp	Cbp52173	61.151	556	1	554	0	CN	different
At_RLM1a	TNL	Cbp	Cbp9251	61.072	858	44	896	0	TNL	same
At_WRR4a	TNL	Cbp	Cbp13411	61.022	998	2	998	0	TNL	same
At_RLM1a	TNL	Cbp	Cbp51486	60.99	1010	1	996	0	TNL	same
At_WRR9	NL	Cbp	Cbp24904	60.968	661	48	707	0	TNL	different
At_RAC1	TNL	Cbp	Cbp26994	60.953	1196	8	1128	0	TNL	same
At_RPP8	CNL	Cbp	Cbp45210	60.914	875	1	867	0	NL	different
At_WRR4a	TNL	Cbp	Cbp19206	60.863	626	9	631	0	TNL	same
At_RLP30	LRR-RLP	Cbp	Cbp10100	60.708	537	255	786	0	LRR-RLP	same
Bju_WRR1	CNL	Cbp	Cbp46346	60.706	906	1	900	0	CNL	same
At_WRR4b	TNL	Cbp	Cbp19206	60.685	613	11	621	0	TNL	same
At_RLM1a	TNL	Cbp	Cbp42255	60.621	838	1	837	0	TNL	same
Bju_WRR1	CNL	Cbp	Cbp53094	60.486	906	1	900	0	CNL	same
At_WRR4a	TNL	Cbp	Cbp13410	60.379	1002	9	1004	0	TNL	same
At_WRR8	TNL	Cbp	Cbp34462	60.361	1163	15	1135	0	TNL	same
At_WRR4b	TNL	Cbp	Cbp13253	60.227	968	1	956	0	TNL	same
At_RLM1a	TNL	Cbp	Cbp9263	60.199	1005	1	995	0	TNL	same
At_RPP8	CNL	Cbp	Cbp520	60.137	873	1	867	0	NL	different
At_RPP8	CNL	Cbp	Cbp6345	60.137	873	1	867	0	NL	different
At_RPP8	CNL	Cbp	Cbp519	60.137	873	1	867	0	NL	different
At_WRR9	NL	Cbp	Cbp9263	60.039	1021	4	1010	0	TNL	different
Bol_FocBo1	TNL	Cru	Carubv10028353m	60.135	148	7	154	1.24E-50	TX	different
At_RLM1b	TNL	Cru	Carubv10006532m	60.759	158	1	157	6.84E-55	TX	different
At_RPP1	TNL	Cru	Carubv10025905m	63.226	155	275	429	6.44E-58	NL	different
At_RLM1a	TNL	Cru	Carubv10006532m	63.291	158	1	157	4.89E-58	TX	different
Bra_Crr1a	TNL	Cru	Carubv10019460m	64	150	65	214	3.91E-58	Other-NLR	different
Bra_Crr1a	TNL	Cru	Carubv10025905m	67.097	155	247	401	7.86E-62	NL	different
At_RPP1	TNL	Cru	Carubv10005833m	61.272	173	97	269	3.72E-68	TX	different
At_WRR9	NL	Cru	Carubv10005833m	62.791	172	17	188	4.53E-69	TX	different
At_RLM1b	TNL	Cru	Carubv10021492m	65.823	158	1	158	2.67E-69	TX	different
At_RLM1b	TNL	Cru	Carubv10005833m	66.279	172	13	184	3.29E-73	TX	different
At_RLM1a	TNL	Cru	Carubv10021492m	68.354	158	1	158	2.81E-73	TX	different
At_WRR4a	TNL	Cru	Carubv10005833m	63.934	183	1	183	2.09E-73	TX	different
At_WRR4a	TNL	Cru	Carubv10021492m	73.418	158	1	157	1.37E-76	TX	different
At_RPP1	TNL	Cru	Carubv10019460m	80.795	151	92	242	2.81E-77	Other-NLR	different
At_WRR4b	TNL	Cru	Carubv10005833m	66.286	175	11	185	1.58E-77	TX	different
At_RLM1a	TNL	Cru	Carubv10005833m	65.714	175	10	184	1.35E-77	TX	different
At_WRR4b	TNL	Cru	Carubv10021492m	75.159	157	3	159	9.92E-82	TX	different
At_NGR1b	RNL	Cru	Carubv10028036m	66.426	277	539	815	8.09E-119	NL	different
At_NGR1a	RNL	Cru	Carubv10028036m	67.509	277	533	809	5.53E-120	NL	different
At_RPP39	CNL	Cru	Carubv10021673m	67.268	388	323	705	1.92E-153	NL	different
At_RLM3	TN	Cru	Carubv10025753m	60.448	402	58	450	3.24E-155	TNL	different
At_RPP2a	TNL	Cru	Carubv10025432m	63.325	409	7	414	3.63E-156	TNL	same
At_RPP2a	TNL	Cru	Carubv10025753m	68.245	359	56	414	2.92E-159	TNL	same
At_RPP2a	TNL	Cru	Carubv10004008m	63.659	410	7	414	8.32E-165	TNL	same
Bna_Rlm9/4/7	Other-RLK	Cru	Carubv10009505m	69.591	342	395	735	3.63E-165	Other-RLK	same
At_RPP4	TNL	Cru	Carubv10004008m	64.352	432	676	1105	3.94E-169	TNL	same
At_BAK1	LRR-RLK	Cru	Carubv10006936m	98.537	615	1	615	0	LRR-RLK	same
At_PBS1	Other-RLK	Cru	Carubv10000618m	98.026	456	1	456	0	Other-RLK	same
At_FLS2	LRR-RLK	Cru	Carubv10025764m	90.235	1147	23	1167	0	LRR-RLK	same
At_SOBIR1	LRR-RLK	Cru	Carubv10025406m	88.043	644	1	641	0	LRR-RLK	same
At_RPS4	TNL	Cru	Carubv10025757m	85.115	1216	1	1199	0	TNL	same
At_ADR1	NL	Cru	Carubv10008348m	83.439	791	1	787	0	NL	same
At_NGR1a	RNL	Cru	Carubv10027991m	82.432	814	1	809	0	CNL	different
At_BAK1	LRR-RLK	Cru	Carubv10013676m	81.798	456	160	615	0	LRR-RLK	same
At_RRS1	TNL	Cru	Carubv10025742m	81.081	1295	1	1277	0	TNL	same
At_BAK1	LRR-RLK	Cru	Carubv10013244m	79.935	618	1	615	0	LRR-RLK	same
At_RPP8	CNL	Cru	Carubv10025854m	79.736	908	1	907	0	CNL	same
At_BAK1	LRR-RLK	Cru	Carubv10019969m	79.504	605	24	615	0	LRR-RLK	same
At_RFO3	Other-RLK	Cru	Carubv10015398m	76.539	861	1	850	0	Other-RLK	same
At_BAK1	LRR-RLK	Cru	Carubv10011846m	76.214	618	10	615	0	LRR-RLK	same
At_RPP2b	TNL	Cru	Carubv10006430m	76.155	1212	1	1207	0	TNL	same
At_RPP2a	TNL	Cru	Carubv10007493m	74.466	889	409	1275	0	TNL	same
At_RPP39	CNL	Cru	Carubv10020008m	74.322	479	146	624	0	NL	different
At_RFO1	Other-RLK	Cru	Carubv10009505m	73.333	345	364	707	0	Other-RLK	same
At_RPP8	CNL	Cru	Carubv10027796m	72.827	909	1	907	0	CNL	same
At_RPP8	CNL	Cru	Carubv10004109m	72.527	910	1	907	0	CNL	same
At_RPP7	NL	Cru	Carubv10019742m	72.299	870	1	866	0	NL	same
At_RPP7	NL	Cru	Carubv10019741m	72.299	870	1	866	0	NL	same
At_RPP39	CNL	Cru	Carubv10019775m	72.241	879	1	870	0	CNL	same
At_WRR8	TNL	Cru	Carubv10027634m	71.959	1159	20	1139	0	TNL	same
At_RPP39	CNL	Cru	Carubv10019842m	71.68	738	146	870	0	NL	different
At_RPP39	CNL	Cru	Carubv10019789m	71.395	846	42	874	0	CNL	same

Bna_Rlm9/4/7	Other-RLK	Cru	Carubv10013039m	71.35	726	22	737	0	Other-RLK	same
At_RFO1	Other-RLK	Cru	Carubv10008263m	71.076	446	308	751	0	Other-RLK	same
At_RPP8	CNL	Cru	Carubv10028494m	70.82	915	1	908	0	CNL	same
At_RPP39	CNL	Cru	Carubv10019851m	70.777	746	146	877	0	NL	different
At_RPP39	CNL	Cru	Carubv10019773m	70.535	879	1	870	0	CNL	same
At_RPP13	CNL	Cru	Carubv10018962m	70.525	838	1	826	0	NL	different
At_RPP8	CNL	Cru	Carubv10008215m	70.229	917	1	907	0	CNL	same
At_RAC1	TNL	Cru	Carubv10027634m	69.509	1161	9	1156	0	TNL	same
At_RFO2	LRR-RLP	Cru	Carubv10022303m	69.501	741	13	748	0	LRR-RLK	different
At_NGR1b	RNL	Cru	Carubv10027991m	69.315	818	5	812	0	CNL	different
Bna_Rlm9/4/7	Other-RLK	Cru	Carubv10021418m	68.992	645	29	669	0	Other-RLK	same
At_RPP8	CNL	Cru	Carubv10008267m	68.531	912	1	906	0	CNL	same
At_RPP39	CNL	Cru	Carubv10019908m	68.175	707	176	874	0	NL	different
Bna_Rlm9/4/7	Other-RLK	Cru	Carubv10019823m	67.98	812	1	794	0	Other-RLK	same
Bol_FocBo1	TNL	Cru	Carubv10025753m	67.208	924	9	928	0	TNL	same
At_RPP7	NL	Cru	Carubv10022028m	66.858	872	1	866	0	NL	same
At_RPP8	CNL	Cru	Carubv10019753m	65.864	914	1	908	0	CNL	same
At_WRR12	TNL	Cru	Carubv10004054m	64.819	992	1	989	0	TNL	same
At_RPS4	TNL	Cru	Carubv10025767m	64.778	1147	5	1139	0	TNL	same
At_RPS4	TNL	Cru	Carubv10025765m	64.778	1147	5	1139	0	TNL	same
At_RPS4	TNL	Cru	Carubv10025766m	64.778	1147	5	1139	0	TNL	same
At_WRR12	TNL	Cru	Carubv10004053m	64.563	1030	1	1021	0	TNL	same
At_RPS4	TNL	Cru	Carubv10025769m	64.516	1147	5	1139	0	TNL	same
Bna_Rlm9/4/7	Other-RLK	Cru	Carubv10008562m	64.172	441	359	793	0	Other-RLK	same
At_RPS5	TNL	Cru	Carubv10012293m	63.995	886	1	884	0	CNL	different
At_RFO1	Other-RLK	Cru	Carubv10008430m	63.946	735	1	718	0	Other-RLK	same
At_RPP5	TNL	Cru	Carubv10004008m	63.844	874	6	847	0	TNL	same
At_RPP7	NL	Cru	Carubv10021896m	63.543	875	1	869	0	NL	same
At_RRS1	TNL	Cru	Carubv10025744m	63.213	1332	8	1277	0	TNL	same
At_ADR1	NL	Cru	Carubv10000246m	62.685	812	6	787	0	NL	same
At_RPS5	TNL	Cru	Carubv10008409m	61.757	740	149	883	0	NL	different
Bna_Rlm9/4/7	Other-RLK	Cru	Carubv10008263m	61.674	454	344	793	0	Other-RLK	same
At_RPP8	CNL	Cru	Carubv10022213m	61.588	781	1	774	0	CNL	same
Bju_WRR1	CNL	Cru	Carubv10025854m	61.294	912	1	900	0	CNL	same
At_RPS5	TNL	Cru	Carubv10008383m	61.227	766	123	883	0	NL	different
Bju_WRR1	CNL	Cru	Carubv10008267m	61.038	906	1	900	0	CNL	same
At_RPP7	NL	Cru	Carubv10019978m	60.548	621	1	615	0	CNL	different
At_RPP7	NL	Cru	Carubv10021618m	60.426	892	4	866	0	CNL	different
At_RPP8	CNL	Cru	Carubv10007066m	60.367	873	1	867	0	NL	different
At_RPP1	TNL	Cru	Carubv10007991m	60.248	888	363	1163	0	NL	different
Bol_FocBo1	TNL	Cru	Carubv10004008m	60.177	904	7	902	0	TNL	same
At_RPS5	TNL	Cru	Carubv10020008m	60.166	482	148	629	0	NL	different
At_RLM1b	TNL	Esa	Thhalv10027706m	61.486	148	532	679	3.84E-51	TNL	same
At_WRR9	NL	Esa	Thhalv10027706m	62.838	148	533	680	8.76E-52	TNL	different
At_RLM1b	TNL	Esa	Thhalv10028273m	60.135	148	1	148	4.73E-54	TX	different
At_RPP5	TNL	Esa	Thhalv10027748m	61.818	165	14	178	3.75E-57	Other-NLR	different
At_RPP5	TNL	Esa	Thhalv10027751m	61.818	165	14	178	3.66E-57	TX	different
At_RPP5	TNL	Esa	Thhalv10027752m	62.424	165	14	178	1.61E-57	TX	different
At_RPP5	TNL	Esa	Thhalv10027754m	62.424	165	14	178	1.57E-57	TX	different
At_RPP5	TNL	Esa	Thhalv10027802m	61.818	165	14	178	1.41E-58	TX	different
At_RPP4	TNL	Esa	Thhalv10026484m	60.927	151	93	243	6.36E-59	TN	different
At_RPP2a	TNL	Esa	Thhalv10026484m	60.667	150	91	240	1.01E-59	TN	different
At_RLM1b	TNL	Esa	Thhalv10027748m	61.35	163	14	176	7.44E-60	Other-NLR	different
At_RLM1b	TNL	Esa	Thhalv10027751m	61.35	163	14	176	6.41E-60	TX	different
Bol_FocBo1	TNL	Esa	Thhalv10027748m	60.87	161	12	172	4.13E-60	Other-NLR	different
Bol_FocBo1	TNL	Esa	Thhalv10027751m	60.87	161	12	172	3.73E-60	TX	different
At_RLM1b	TNL	Esa	Thhalv10027802m	61.35	163	14	176	1.30E-60	TX	different
At_WRR4a	TNL	Esa	Thhalv10027748m	60.119	168	14	181	9.04E-61	Other-NLR	different
At_WRR4a	TNL	Esa	Thhalv10027751m	60.119	168	14	181	9.00E-61	TX	different
Bol_FocBo1	TNL	Esa	Thhalv10027802m	60.87	161	12	172	7.66E-61	TX	different
At_RPP1	TNL	Esa	Thhalv10027752m	60.87	161	100	260	6.69E-61	TX	different
At_RPP1	TNL	Esa	Thhalv10027748m	61.491	161	100	260	6.51E-61	Other-NLR	different
At_RPP1	TNL	Esa	Thhalv10027751m	61.491	161	100	260	6.29E-61	TX	different
At_RPP1	TNL	Esa	Thhalv10027754m	60.87	161	100	260	5.83E-61	TX	different
Bra_Crr1a	TNL	Esa	Thhalv10027748m	61.491	161	72	232	3.77E-61	Other-NLR	different
Bra_Crr1a	TNL	Esa	Thhalv10027751m	61.491	161	72	232	3.44E-61	TX	different
At_RPP5	TNL	Esa	Thhalv10026484m	63.636	154	92	245	1.98E-61	TN	different
At_WRR4a	TNL	Esa	Thhalv10027802m	60.119	168	14	181	1.80E-61	TX	different
At_RPP1	TNL	Esa	Thhalv10027802m	61.491	161	100	260	1.65E-61	TX	different
Bra_Crr1a	TNL	Esa	Thhalv10027802m	61.491	161	72	232	1.04E-61	TX	different
At_RLM1a	TNL	Esa	Thhalv10027748m	60.479	167	16	182	2.93E-62	Other-NLR	different
At_RLM1a	TNL	Esa	Thhalv10027751m	60.479	167	16	182	2.89E-62	TX	different
Bra_Crr1a	TNL	Esa	Thhalv10027752m	62.112	161	72	232	8.19E-63	TX	different
Bra_Crr1a	TNL	Esa	Thhalv10027754m	62.112	161	72	232	8.06E-63	TX	different
At_RLM1a	TNL	Esa	Thhalv10027752m	60.355	169	16	184	6.69E-63	TX	different
At_RLM3	TN	Esa	Thhalv10026484m	62.667	150	89	238	6.34E-63	TN	same
At_RLM1a	TNL	Esa	Thhalv10027754m	60.355	169	16	184	6.09E-63	TX	different
At_RLM1a	TNL	Esa	Thhalv10027802m	60.479	167	16	182	4.85E-63	TX	different
At_WRR4a	TNL	Esa	Thhalv10027752m	61.176	170	14	183	1.97E-63	TX	different
At_WRR4a	TNL	Esa	Thhalv10027754m	61.176	170	14	183	1.92E-63	TX	different
At_WRR4b	TNL	Esa	Thhalv10027748m	63.58	162	16	177	4.91E-65	Other-NLR	different
At_WRR4b	TNL	Esa	Thhalv10027751m	63.58	162	16	177	4.74E-65	TX	different
At_RPP2a	TNL	Esa	Thhalv10024445m	69.091	165	250	414	1.77E-65	NL	different
At_RLM3	TN	Esa	Thhalv10024445m	60.106	188	248	427	1.34E-65	NL	different
At_WRR4b	TNL	Esa	Thhalv10027802m	63.58	162	16	177	1.29E-65	TX	different
At_WRR4b	TNL	Esa	Thhalv10027752m	62.353	170	16	185	8.69E-66	TX	different

At_WRR4b	TNL	Esa	Thhalv10027754m	62.353	170	16	185	8.32E-66	TX	different
Bra_cRa/cRb	TNL	Esa	Thhalv10011769m	61.765	204	16	219	1.01E-66	TX	different
Bol_FocBo1	TNL	Esa	Thhalv10026484m	76.667	150	91	240	4.82E-76	TN	different
At_RLP32	LRR-RLP	Esa	Thhalv10003118m	60.417	240	614	853	3.08E-77	LRR-RLP	same
At_RAC1	TNL	Esa	Thhalv10028756m	62.866	307	146	451	2.96E-112	NBS	different
At_WRR8	TNL	Esa	Thhalv10028756m	60.991	323	156	477	5.46E-119	NBS	different
At_RPP2a	TNL	Esa	Thhalv10019465m	60.399	351	66	414	1.27E-132	TNL	same
At_RLP32	LRR-RLP	Esa	Thhalv10000552m	60.513	390	478	854	1.64E-137	LRR-RLP	same
At_RPP39	CNL	Esa	Thhalv10023842m	61.347	401	479	870	6.34E-143	LRR-RLP	different
At_RPP2a	TNL	Esa	Thhalv10026940m	60.145	414	1	414	4.18E-151	TN	different
At_WRR4b	TNL	Esa	Thhalv10000509m	64.467	394	11	402	1.04E-168	TN	different
At_NGR1b	RNL	Esa	Thhalv10005517m	61.717	431	391	815	1.78E-170	NL	different
At_RLM1a	TNL	Esa	Thhalv10000509m	64.573	398	10	406	2.30E-174	TN	different
At_WRR9	NL	Esa	Thhalv10000509m	63.747	411	4	407	1.97E-174	TN	different
At_BAK1	LRR-RLK	Esa	Thhalv10024694m	95.935	615	1	615	0	LRR-RLK	same
At_RFO1	Other-RLK	Esa	Thhalv10018175m	87.484	759	1	751	0	Other-RLK	same
At_RPS2	NL	Esa	Thhalv10024353m	86.374	910	1	909	0	CNL	different
At_RPM1	NL	Esa	Thhalv10020008m	83.746	929	1	926	0	NL	same
At_FLS2	LRR-RLK	Esa	Thhalv10000746m	82.165	1155	23	1173	0	LRR-RLK	same
At_SOBIR1	LRR-RLK	Esa	Thhalv10016374m	81.182	643	1	641	0	LRR-RLK	same
At_BAK1	LRR-RLK	Esa	Thhalv10019435m	79.833	600	29	615	0	LRR-RLK	same
At_BAK1	LRR-RLK	Esa	Thhalv10007069m	78.477	604	24	615	0	LRR-RLK	same
At_NGR1a	RNL	Esa	Thhalv10003662m	78.022	819	1	809	0	CNL	different
At_RLP42	LRR-RLP	Esa	Thhalv10010006m	73.576	878	1	876	0	LRR-RLP	same
At_RPP2a	TNL	Esa	Thhalv10027276m	73.54	582	720	1299	0	TNL	same
At_WRR4b	TNL	Esa	Thhalv10000023m	73.084	1070	1	1059	0	TNL	same
At_RPS4	TNL	Esa	Thhalv10000750m	73.045	1202	1	1196	0	TNL	same
At_RLM1b	TNL	Esa	Thhalv10000647m	72.771	628	1	626	0	TNL	same
At_RFO2	LRR-RLP	Esa	Thhalv10006928m	72.57	751	1	748	0	LRR-RLP	same
At_RFO2	LRR-RLP	Esa	Thhalv10018044m	71.55	703	46	748	0	LRR-RLK	different
At_RLM1b	TNL	Esa	Thhalv10000695m	71.383	622	7	626	0	TNL	same
At_RLM1b	TNL	Esa	Thhalv10000509m	70.968	403	6	407	0	TN	different
At_RPP8	CNL	Esa	Thhalv10002442m	70.172	580	1	573	0	NBS	different
At_WRR4b	TNL	Esa	Thhalv10027634m	70.131	914	5	916	0	TNL	same
At_RPP39	CNL	Esa	Thhalv10023260m	69.811	848	42	870	0	CNL	same
At_RPP39	CNL	Esa	Thhalv10023248m	69.629	889	1	870	0	CNL	same
At_RPP39	CNL	Esa	Thhalv10023258m	69.58	881	1	868	0	CNL	same
At_RLP32	LRR-RLP	Esa	Thhalv10002360m	69.347	659	197	854	0	LRR-RLP	same
At_RPP39	CNL	Esa	Thhalv10023286m	68.828	802	1	792	0	CNL	same
At_RPP39	CNL	Esa	Thhalv10023257m	68.806	888	1	875	0	CNL	same
At_NGR1a	RNL	Esa	Thhalv10020099m	68.666	817	1	809	0	CNL	different
At_NGR1b	RNL	Esa	Thhalv10020099m	68.187	811	9	815	0	CNL	different
At_RPP39	CNL	Esa	Thhalv10023266m	68.146	879	1	870	0	CNL	same
At_NGR1b	RNL	Esa	Thhalv10003662m	68.039	826	3	815	0	CNL	different
At_WRR4a	TNL	Esa	Thhalv10027634m	67.634	896	3	897	0	TNL	same
At_RPP2b	TNL	Esa	Thhalv10024441m	67.6	821	1	818	0	TNL	same
At_RLP32	LRR-RLP	Esa	Thhalv10000034m	67.6	750	128	854	0	LRR-RLP	same
Bju_WRR1	CNL	Esa	Thhalv10002442m	67.526	582	1	571	0	NBS	different
At_RLM1b	TNL	Esa	Thhalv10023230m	67.512	1025	1	1013	0	TNL	same
At_RLP23	LRR-RLP	Esa	Thhalv10010006m	67.269	886	1	885	0	LRR-RLP	same
At_RLM1a	TNL	Esa	Thhalv10000647m	67.095	623	3	622	0	TNL	same
At_WRR9	NL	Esa	Thhalv10000647m	66.773	626	5	627	0	TNL	different
At_RLM1a	TNL	Esa	Thhalv10000695m	66.504	615	10	621	0	TNL	same
At_NGR1a	RNL	Esa	Thhalv10005443m	66.299	816	1	806	0	CNL	different
At_RRS1	TNL	Esa	Thhalv10001165m	66.127	803	1	799	0	TNL	same
At_NGR1a	RNL	Esa	Thhalv10005517m	66.033	421	396	809	0	NL	different
At_RLP32	LRR-RLP	Esa	Thhalv10000044m	65.826	872	9	854	0	LRR-RLP	same
At_RLP23	LRR-RLP	Esa	Thhalv10016266m	65.69	717	175	889	0	LRR-RLP	same
At_RLP42	LRR-RLP	Esa	Thhalv10016266m	65.429	700	182	876	0	LRR-RLP	same
At_WRR9	NL	Esa	Thhalv10000695m	65.428	619	12	627	0	TNL	different
Bna_LepR3/Rlm2	LRR-RLP	Esa	Thhalv10002360m	65.263	665	286	948	0	LRR-RLP	same
At_RLP23	LRR-RLP	Esa	Thhalv10016267m	64.903	718	175	889	0	LRR-RLP	same
At_NGR1b	RNL	Esa	Thhalv10005443m	64.233	808	11	813	0	CNL	different
At_RLP30	LRR-RLP	Esa	Thhalv10024467m	64.194	782	1	779	0	LRR-RLP	same
At_WRR4a	TNL	Esa	Thhalv10000023m	63.836	1001	9	1002	0	TNL	same
At_RLP32	LRR-RLP	Esa	Thhalv10000035m	63.745	924	9	854	0	LRR-RLP	same
Bol_FocBo1	TNL	Esa	Thhalv10026940m	63.498	526	5	513	0	TN	different
At_RLP42	LRR-RLP	Esa	Thhalv10016267m	63.421	719	182	889	0	LRR-RLP	same
At_RLP30	LRR-RLP	Esa	Thhalv10018341m	62.937	572	203	769	0	LRR-RLP	same
At_RFO1	Other-RLK	Esa	Thhalv10006943m	62.807	734	2	718	0	Other-RLK	same
At_ADR1	NL	Esa	Thhalv10012720m	62.779	806	6	787	0	NL	same
Bol_FocBo1	TNL	Esa	Thhalv10024445m	62.537	670	250	907	0	NL	different
At_RFO1	Other-RLK	Esa	Thhalv10009310m	62.5	768	10	751	0	Other-RLK	same
At_WRR4b	TNL	Esa	Thhalv10000647m	62.179	624	4	624	0	TNL	same
Bna_LepR3/Rlm2	LRR-RLP	Esa	Thhalv10000044m	62.152	790	163	948	0	LRR-RLP	same
At_RPS5	TNL	Esa	Thhalv10024140m	61.959	531	1	530	0	CN	different
At_RLP30	LRR-RLP	Esa	Thhalv10024473m	61.911	806	1	774	0	LRR-RLP	same
At_RLP32	LRR-RLP	Esa	Thhalv10000632m	61.846	899	17	854	0	LRR-RLP	same
Bol_FocBo1	TNL	Esa	Thhalv10019465m	61.528	720	65	778	0	TNL	same
At_RLM1a	TNL	Esa	Thhalv10018052m	61.393	1005	1	997	0	TNL	same
At_RPP5	TNL	Esa	Thhalv10026940m	61.308	535	3	518	0	TN	different
At_WRR4b	TNL	Esa	Thhalv10000695m	61.264	617	11	623	0	TNL	same
Bna_Rlm9/4/7	Other-RLK	Esa	Thhalv10001848m	60.833	720	100	794	0	Other-RLK	same
At_WRR8	TNL	Esa	Thhalv10003136m	60.794	1158	12	1139	0	TNL	same
At_RPP7	NL	Esa	Thhalv10023235m	60.585	992	1	977	0	NL	same
At_ADR1	NL	Esa	Thhalv10024421m	60.488	820	3	787	0	NL	same

At_RLP32	LRR-RLP	Esa	Thhalv10002367m	60.211	950	9	854	0	LRR-RLP	same
Bra_Crr1a	TNL	Lal	LA_scaffold1274_1	60.494	162	808	968	9.27E-49	TNL	same
At_RPP4	TNL	Lal	LA_C64493_1	60.479	167	715	880	8.24E-53	TNL	same
At_WRR4a	TNL	Lal	LA_scaffold3244_19	62.416	149	9	157	3.04E-54	TX	different
At_WRR4b	TNL	Lal	LA_scaffold3244_19	61.074	149	11	159	8.33E-56	TX	different
At_RLM1a	TNL	Lal	LA_scaffold3244_19	61.074	149	10	158	2.71E-56	TX	different
At_WRR9	NL	Lal	LA_scaffold345_4	63.087	149	13	161	8.45E-57	TNL	different
At_RPP1	TNL	Lal	LA_scaffold707_4	63.636	154	248	401	7.39E-57	NL	different
Bra_Crr1a	TNL	Lal	LA_scaffold3244_19	63.514	148	67	214	4.74E-60	TX	different
At_RPP4	TNL	Lal	LA_scaffold3465_1	64.118	170	10	179	1.08E-61	TNL	same
Bol_FocBo1	TNL	Lal	LA_scaffold512_7	63.095	168	97	264	5.06E-62	TNL	same
At_RPP1	TNL	Lal	LA_scaffold3244_19	64.238	151	92	242	4.16E-63	TX	different
At_RPP5	TNL	Lal	LA_scaffold3465_1	66.667	168	12	179	7.99E-64	TNL	same
At_RLM3	TN	Lal	LA_scaffold3465_1	61.143	175	6	180	4.99E-65	TNL	different
At_WRR9	NL	Lal	LA_scaffold292_4	60.795	176	307	482	1.03E-66	TN	different
At_RLM1b	TNL	Lal	LA_scaffold2656_3	62.651	166	16	181	3.97E-67	TX	different
At_WRR4a	TNL	Lal	LA_scaffold2656_3	62.424	165	15	179	3.96E-67	TX	different
Bol_FocBo1	TNL	Lal	LA_scaffold3465_1	66.279	172	6	177	6.04E-69	TNL	same
At_RLM1a	TNL	Lal	LA_scaffold2656_3	66.061	165	16	180	1.19E-72	TX	different
At_WRR4b	TNL	Lal	LA_scaffold2656_3	67.47	166	17	182	3.31E-75	TX	different
At_WRR4b	TNL	Lal	LA_scaffold345_4	84.076	157	1	157	6.84E-77	TNL	same
At_RLM1b	TNL	Lal	LA_scaffold292_4	72.571	175	303	477	3.20E-81	TN	different
At_RPP2a	TNL	Lal	LA_scaffold512_7	64.557	237	178	414	4.68E-95	TNL	same
At_RPP1	TNL	Lal	LA_scaffold1274_1	62.4	250	93	335	1.57E-96	TNL	same
At_RPP1	TNL	Lal	LA_scaffold3231_2	66.531	245	136	380	8.46E-106	TNL	same
At_RLP30	LRR-RLP	Lal	LA_scaffold1391_3	60.714	364	427	786	3.11E-135	LRR-RLP	same
At_RPP1	TNL	Lal	LA_C59137_1	64.307	339	66	402	3.45E-139	TN	different
At_PBS1	Other-RLK	Lal	LA_scaffold3521_10	66.447	304	61	364	1.31E-140	Other-RLK	same
At_RPP2a	TNL	Lal	LA_scaffold1873_18	61.916	407	8	414	7.39E-156	TNL	same
At_WRR4b	TNL	Lal	LA_C58557_1	61.013	395	6	398	2.37E-160	TN	different
At_RLM1b	TNL	Lal	LA_C58557_1	60.56	393	5	396	7.93E-164	TN	different
At_PBS1	Other-RLK	Lal	LA_scaffold729_7	73.09	301	65	364	1.45E-165	Other-RLK	same
At_RLM1a	TNL	Lal	LA_C58557_1	61.869	396	5	399	4.98E-170	TN	different
At_RPP2a	TNL	Lal	LA_C60571_1	62.85	428	10	414	4.91E-173	Other-NLR	different
At_RPP2a	TNL	Lal	LA_scaffold512_4	63.3	406	9	414	1.41E-173	TNL	same
At_RPP2a	TNL	Lal	LA_C64493_1	65.926	405	10	414	1.27E-178	TNL	same
At_BAK1	LRR-RLK	Lal	LA_scaffold1550_50	89.431	615	1	615	0	LRR-RLK	same
At_PBS1	Other-RLK	Lal	LA_scaffold3472_4	86.653	487	1	456	0	Other-RLK	same
At_RFO1	Other-RLK	Lal	LA_scaffold1187_3	83.134	753	2	751	0	Other-RLK	same
At_RPM1	NL	Lal	LA_scaffold698_109	83.047	932	1	926	0	NL	same
At_SOBIR1	LRR-RLK	Lal	LA_scaffold151_27	82.893	643	1	641	0	LRR-RLK	same
At_RPS2	NL	Lal	LA_scaffold2413_6	82.34	889	21	909	0	CNL	different
At_FLS2	LRR-RLK	Lal	LA_scaffold1430_19	80.645	1178	1	1173	0	LRR-RLK	same
At_RLP1	LRR-RLP	Lal	LA_scaffold863_6	80.223	1077	1	1076	0	LRR-RLP	same
At_BAK1	LRR-RLK	Lal	LA_scaffold3434_1	78.025	628	1	615	0	LRR-RLK	same
At_RLP32	LRR-RLP	Lal	LA_scaffold611_46	76.896	857	1	854	0	LRR-RLP	same
At_BAK1	LRR-RLK	Lal	LA_scaffold1533_74	73.333	615	23	615	0	LRR-RLK	same
At_RFO3	Other-RLK	Lal	LA_scaffold1835_9	70.366	874	4	850	0	Other-RLK	same
At_WRR4b	TNL	Lal	LA_scaffold1320_11	70.091	1100	1	1096	0	TNL	same
At_RFO3	Other-RLK	Lal	LA_scaffold1710_47	69.586	628	234	850	0	Other-RLK	same
At_NGR1a	RNL	Lal	LA_scaffold1324_6	69.25	813	1	807	0	NL	different
At_RFO2	LRR-RLP	Lal	LA_scaffold869_19	68.716	732	17	748	0	LRR-RLK	different
At_RLP32	LRR-RLP	Lal	LA_scaffold1263_3	68.39	851	9	854	0	LRR-RLP	same
At_RPP13	CNL	Lal	LA_scaffold1596_7	67.596	861	1	830	0	CNL	same
At_RFO2	LRR-RLP	Lal	LA_scaffold402_9	67.475	701	20	718	0	LRR-RLP	same
At_RPP13	CNL	Lal	LA_scaffold1511_29	67.015	861	1	830	0	NL	different
At_RPP39	CNL	Lal	LA_scaffold903_5	66.712	736	145	871	0	NL	different
At_RLM1b	TNL	Lal	LA_C66795_2	65.774	1008	1	999	0	TNL	same
At_RLP23	LRR-RLP	Lal	LA_scaffold49_15	65.49	878	1	874	0	LRR-RLP	same
At_RFO1	Other-RLK	Lal	LA_scaffold2496_3	65.442	735	1	718	0	Other-RLK	same
At_NGR1a	RNL	Lal	LA_scaffold653_3	64.691	810	12	809	0	NL	different
At_RAC1	TNL	Lal	LA_scaffold49_1	64.638	608	160	762	0	NL	different
At_WRR8	TNL	Lal	LA_scaffold49_1	64.521	606	170	769	0	NL	different
At_RLP42	LRR-RLP	Lal	LA_scaffold49_15	64.376	873	1	868	0	LRR-RLP	same
At_WRR4a	TNL	Lal	LA_scaffold1320_11	64.087	1008	1	1000	0	TNL	same
At_WRR8	TNL	Lal	LA_scaffold968_13	64.061	1113	12	1103	0	TNL	same
At_NGR1b	RNL	Lal	LA_scaffold1324_6	63.98	819	5	812	0	NL	different
At_RLP23	LRR-RLP	Lal	LA_scaffold659_19	63.877	717	175	889	0	LRR-RLP	same
At_NGR1b	RNL	Lal	LA_scaffold1143_11	63.625	822	3	815	0	NL	different
At_RLM1a	TNL	Lal	LA_scaffold2290_11	63.177	831	16	845	0	TNL	same
At_RLP23	LRR-RLP	Lal	LA_scaffold1437_44	63.04	717	175	889	0	LRR-RLP	same
Bna_LepR3/Rlm2	LRR-RLP	Lal	LA_scaffold611_46	63.038	744	204	943	0	LRR-RLP	same
At_RPP13	CNL	Lal	LA_scaffold1596_4	62.85	856	1	828	0	CNL	same
At_RAC1	TNL	Lal	LA_scaffold1515_10	62.549	510	1	510	0	TN	different
Bna_LepR3/Rlm2	LRR-RLP	Lal	LA_scaffold846_9	62.474	978	9	943	0	LRR-RLP	same
At_RPP1	TNL	Lal	LA_scaffold76_6	62.332	446	66	509	0	TNL	same
At_WRR4b	TNL	Lal	LA_scaffold345_4	62.234	940	160	1061	0	TNL	same
Bna_LepR3/Rlm2	LRR-RLP	Lal	LA_scaffold1263_3	62.234	752	201	949	0	LRR-RLP	same
At_RLM1a	TNL	Lal	LA_scaffold1058_13	62.202	1008	1	995	0	TNL	same
At_RAC1	TNL	Lal	LA_scaffold968_13	62.118	1114	1	1103	0	TNL	same
At_RLP42	LRR-RLP	Lal	LA_scaffold659_19	61.859	721	182	889	0	LRR-RLP	same
At_ADR1	NL	Lal	LA_scaffold2239_11	61.798	801	6	787	0	NL	same
At_RLM1b	TNL	Lal	LA_scaffold2290_11	61.638	1001	16	1010	0	TNL	same
At_RLP32	LRR-RLP	Lal	LA_scaffold846_9	61.461	794	95	860	0	LRR-RLP	same
At_RLM1b	TNL	Lal	LA_scaffold2356_5	61.424	1011	11	996	0	TNL	same
At_WRR8	TNL	Lal	LA_scaffold1515_10	61.417	508	12	519	0	TN	different

At_NGR1a	RNL	Lal	LA_scaffold1143_11	61.389	821	1	809	0	NL	different
Bol_FocBo1	TNL	Lal	LA_C64493_1	61.245	707	8	711	0	TNL	same
At_RLP42	LRR-RLP	Lal	LA_scaffold1437_44	60.779	719	182	889	0	LRR-RLP	same
At_RFO1	Other-RLK	Lal	LA_scaffold2496_4	60.53	755	6	746	0	Other-RLK	same
At_ADR1	NL	Lal	LA_scaffold2735_1	60.358	782	11	764	0	NL	same
At_WRR4a	TNL	Lal	LA_scaffold345_4	60.142	848	159	997	0	TNL	same
At_RPP7	NL	Lme	evm.model.scaffold111.350	62.667	150	996	1136	4.15E-47	NL	same
At_RPP2a	TNL	Lme	evm.model.scaffold28.210	61.438	153	262	414	1.03E-51	NL	different
At_RFO1	Other-RLK	Lme	evm.model.scaffold295.404	62.821	156	310	465	4.78E-52	Other-RLK	same
At_NGR1b	RNL	Lme	evm.model.scaffold284.34	66.892	148	326	472	1.46E-54	NL	different
At_RPS5	TNL	Lme	evm.model.scaffold310.40	67.55	151	120	270	2.81E-56	NBS	different
At_RPP2a	TNL	Lme	evm.model.scaffold81.428	63.03	165	250	414	8.04E-59	NL	different
At_NGR1a	RNL	Lme	evm.model.scaffold284.34	70.47	149	320	467	6.37E-60	NL	different
Bna_LepR3/Rlm2	LRR-RLP	Lme	evm.model.scaffold482.173	64.324	185	760	943	1.30E-60	LRR-RLP	same
At_RLP32	LRR-RLP	Lme	evm.model.scaffold482.173	68.571	175	677	850	5.45E-63	LRR-RLP	same
Bna_LepR3/Rlm2	LRR-RLP	Lme	evm.model.scaffold223.186	65.385	182	743	924	1.07E-64	LRR-RLP	same
At_RLP30	LRR-RLP	Lme	evm.model.scaffold26.340	63.978	186	585	768	1.30E-67	LRR-RLP	same
Bna_Rlm9/4/7	Other-RLK	Lme	evm.model.scaffold356.593	60.28	214	396	608	4.10E-71	Other-RLK	same
At_RLP32	LRR-RLP	Lme	evm.model.scaffold494.52	65.079	189	660	847	1.75E-71	LRR-RLP	same
Bna_LepR3/Rlm2	LRR-RLP	Lme	evm.model.scaffold494.52	63.874	191	743	932	1.11E-71	LRR-RLP	same
At_RLP30	LRR-RLP	Lme	evm.model.scaffold26.341	61.792	212	562	772	6.63E-73	LRR-RLP	same
Bna_Rlm9/4/7	Other-RLK	Lme	evm.model.scaffold830.322	68.478	184	354	530	4.92E-73	Other-RLK	same
At_RLM1b	TNL	Lme	evm.model.scaffold452.106	64.894	188	241	421	1.27E-73	NBS	different
At_RLP32	LRR-RLP	Lme	evm.model.scaffold223.186	72.472	178	662	839	2.34E-74	LRR-RLP	same
At_RPP39	CNL	Lme	evm.model.scaffold50.62	65.174	201	323	522	3.84E-78	NBS	different
At_RLP30	LRR-RLP	Lme	evm.model.scaffold53.617	66.667	198	580	777	5.39E-84	LRR-RLP	same
At_RLP30	LRR-RLP	Lme	evm.model.scaffold625.49	68.78	205	577	781	7.55E-86	LRR-RLP	same
At_RAC1	TNL	Lme	evm.model.scaffold891.92	63.672	256	158	413	2.58E-89	NBS	different
At_WRR8	TNL	Lme	evm.model.scaffold891.92	63.672	256	168	423	2.22E-92	NBS	different
Bna_LepR3/Rlm2	LRR-RLP	Lme	evm.model.scaffold695.418	64.317	227	718	943	2.14E-92	LRR-RLP	same
At_RFO1	Other-RLK	Lme	evm.model.scaffold356.593	76.923	234	343	575	1.13E-93	Other-RLK	same
At_WRR12	TNL	Lme	evm.model.scaffold95.272	60.357	280	11	288	1.08E-94	TNL	same
At_RFO3	Other-RLK	Lme	evm.model.scaffold10.670	60.853	258	495	748	5.73E-97	Other-RLK	same
At_RPP8	CNL	Lme	evm.model.scaffold490.31	65.164	244	168	411	3.37E-98	CN	different
At_RPP39	CNL	Lme	evm.model.scaffold111.249	65.126	238	359	595	1.70E-102	NL	different
At_WRR9	NL	Lme	evm.model.scaffold550.75	62.934	259	309	566	9.50E-105	NBS	different
At_RLP32	LRR-RLP	Lme	evm.model.scaffold695.418	73.303	221	634	854	7.49E-109	LRR-RLP	same
At_RLM1a	TNL	Lme	evm.model.scaffold550.75	63.813	257	305	560	1.16E-109	NBS	different
At_RFO1	Other-RLK	Lme	evm.model.scaffold356.593	69.112	259	11	269	1.35E-113	Other-RLK	same
At_RPP8	CNL	Lme	evm.model.scaffold234.80	61.032	349	560	907	5.80E-115	CNL	same
Bna_LepR3/Rlm2	LRR-RLP	Lme	evm.model.scaffold482.174	62.145	317	624	937	1.56E-118	LRR-RLP	same
At_RLM1b	TNL	Lme	evm.model.scaffold550.75	70.498	261	305	565	1.44E-121	NBS	different
At_RPP8	CNL	Lme	evm.model.scaffold96.54	65.399	263	210	468	6.90E-122	NBS	different
Bna_LepR3/Rlm2	LRR-RLP	Lme	evm.model.scaffold339.145	61.716	303	641	943	5.73E-122	LRR-RLP	same
At_RPS2	NL	Lme	evm.model.scaffold384.356	82.353	221	301	521	3.12E-123	NBS	different
At_RLP32	LRR-RLP	Lme	evm.model.scaffold482.174	63.636	319	537	852	1.49E-124	LRR-RLP	same
At_BAK1	LRR-RLK	Lme	evm.model.scaffold928.81	71.042	259	326	584	2.40E-127	LRR-RLK	same
Bna_Rlm9/4/7	Other-RLK	Lme	evm.model.scaffold275.24	62.426	338	312	644	3.74E-129	Other-RLK	same
At_PBS1	Other-RLK	Lme	evm.model.scaffold306.22	63.607	305	61	364	2.00E-133	Other-RLK	same
At_PBS1	Other-RLK	Lme	evm.model.scaffold785.113	74.902	255	111	364	4.07E-135	Other-RLK	same
At_RPS5	TNL	Lme	evm.model.scaffold384.134	62.5	352	135	486	3.48E-145	NBS	different
At_RPP39	CNL	Lme	evm.model.scaffold195.22	61.765	408	471	869	4.14E-146	CNL	same
At_RPS5	TNL	Lme	evm.model.scaffold50.88	60.934	407	1	407	1.12E-148	CNL	different
At_RPP39	CNL	Lme	evm.model.scaffold971.3	68.038	316	1	315	4.37E-149	CN	different
At_RPP7	NL	Lme	evm.model.scaffold482.72	62.864	412	458	866	2.66E-149	NL	same
At_NGR1b	RNL	Lme	evm.model.scaffold113.778	62.35	417	406	815	1.82E-149	CNL	different
At_RPS5	TNL	Lme	evm.model.scaffold50.115	61.481	405	1	405	3.05E-155	CNL	different
Bna_Rlm9/4/7	Other-RLK	Lme	evm.model.scaffold550.39	61.408	412	159	568	1.22E-157	Other-RLK	same
At_RPP7	NL	Lme	evm.model.scaffold482.72	62.924	383	1	382	9.83E-160	NL	same
At_NGR1a	RNL	Lme	evm.model.scaffold113.778	65.459	414	404	809	1.11E-160	CNL	different
Bna_Rlm9/4/7	Other-RLK	Lme	evm.model.scaffold855.38	63.504	411	127	530	4.91E-163	Other-RLK	same
At_PBS1	Other-RLK	Lme	evm.model.scaffold830.589	70	310	71	378	5.06E-164	Other-RLK	same
At_NGR1b	RNL	Lme	evm.model.scaffold352.97	61.779	416	406	815	1.34E-164	CNL	different
Bna_LepR3/Rlm2	LRR-RLP	Lme	evm.model.scaffold384.176	63.333	420	515	928	3.78E-169	LRR-RLP	same
At_RPP39	CNL	Lme	evm.model.scaffold268.9	72.121	330	1	330	3.22E-171	CNL	same
At_RPP39	CNL	Lme	evm.model.scaffold195.22	64.128	407	1	405	2.80E-172	CNL	same
At_RLP32	LRR-RLP	Lme	evm.model.scaffold191.290	61.785	437	418	854	2.76E-175	LRR-RLP	same
At_NGR1a	RNL	Lme	evm.model.scaffold352.97	65.459	414	404	809	1.37E-177	CNL	different
At_RPS2	NL	Lme	evm.model.scaffold3866.2	80.872	298	192	489	2.24E-178	NBS	different
At_RPS2	NL	Lme	evm.model.scaffold482.7	81.879	298	192	489	1.99E-179	NBS	different
At_BAK1	LRR-RLK	Lme	evm.model.scaffold675.276	96.748	615	1	615	0	LRR-RLK	same
At_BAK1	LRR-RLK	Lme	evm.model.scaffold95.14	96.748	615	1	615	0	LRR-RLK	same
At_BAK1	LRR-RLK	Lme	evm.model.scaffold236.905	96.423	615	1	615	0	LRR-RLK	same
At_BAK1	LRR-RLK	Lme	evm.model.scaffold70.479	95.772	615	1	615	0	LRR-RLK	same
At_PBS1	Other-RLK	Lme	evm.model.scaffold98.135	88.641	493	1	456	0	Other-RLK	same
At_RFO1	Other-RLK	Lme	evm.model.scaffold53.735	82.699	578	1	575	0	Other-RLK	same
At_FLS2	LRR-RLK	Lme	evm.model.scaffold115.6	81.841	1173	4	1173	0	LRR-RLK	same
At_FLS2	LRR-RLK	Lme	evm.model.scaffold369.118	81.159	1173	4	1173	0	LRR-RLK	same
At_SOBIR1	LRR-RLK	Lme	evm.model.scaffold185.309	79.953	429	41	468	0	LRR-RLK	same
At_SOBIR1	LRR-RLK	Lme	evm.model.scaffold146.494	79.751	642	1	641	0	LRR-RLK	same
At_SOBIR1	LRR-RLK	Lme	evm.model.scaffold110.60	79.595	642	1	641	0	LRR-RLK	same
At_RPS4	TNL	Lme	evm.model.scaffold242.956	79.319	1233	1	1209	0	TNL	same
At_RPS2	NL	Lme	evm.model.scaffold297.2	79.098	909	1	909	0	CNL	different
At_BAK1	LRR-RLK	Lme	evm.model.scaffold117.318	77.578	611	9	614	0	LRR-RLK	same
At_BAK1	LRR-RLK	Lme	evm.model.scaffold265.342	75.929	619	9	615	0	LRR-RLK	same
At_RPS2	NL	Lme	evm.model.scaffold26.219	75.527	617	293	909	0	NL	same

At_BAK1	LRR-RLK	Lme	evm.model.scaffold428.149	75.453	607	9	615	0	LRR-RLK	same
At_RPS2	NL	Lme	evm.model.scaffold136.39	74.807	909	1	909	0	CNL	different
At_RFO3	Other-RLK	Lme	evm.model.scaffold345.4	74.769	864	1	849	0	Other-RLK	same
At_BAK1	LRR-RLK	Lme	evm.model.scaffold216.149	74.516	620	9	615	0	LRR-RLK	same
At_BAK1	LRR-RLK	Lme	evm.model.scaffold34.49	74.424	434	29	450	0	LRR-RLK	same
At_BAK1	LRR-RLK	Lme	evm.model.scaffold195.513	72.231	605	24	615	0	LRR-RLK	same
At_BAK1	LRR-RLK	Lme	evm.model.scaffold57.15	72.231	605	24	615	0	LRR-RLK	same
At_BAK1	LRR-RLK	Lme	evm.model.scaffold1220.2	71.901	605	24	615	0	LRR-RLK	same
At_BAK1	LRR-RLK	Lme	evm.model.scaffold339.194	71.122	606	9	612	0	LRR-RLK	same
Bna_MPK9	Other-RLK	Lme	evm.model.scaffold80.44	70.921	478	100	562	0	Other-RLK	same
At_NGR1a	RNL	Lme	evm.model.scaffold113.778	70.777	811	4	806	0	CNL	different
At_RPP2b	TNL	Lme	evm.model.scaffold36.157	70.757	489	719	1207	0	CNL	different
At_NGR1a	RNL	Lme	evm.model.scaffold52.101	69.89	817	1	809	0	NL	different
At_RFO2	LRR-RLP	Lme	evm.model.scaffold57.64	69.608	714	24	737	0	LRR-RLK	different
Bna_Rlm9/4/7	Other-RLK	Lme	evm.model.scaffold1108.2	69.588	388	217	603	0	Other-RLK	same
At_RPS4	TNL	Lme	evm.model.scaffold468.461	69.546	903	1	892	0	Other-NLR	different
At_RPP39	CNL	Lme	evm.model.scaffold297.20	69.531	384	1	383	0	CN	different
At_RFO2	LRR-RLP	Lme	evm.model.scaffold646.66	69.517	725	24	748	0	LRR-RLK	different
At_RPP39	CNL	Lme	evm.model.scaffold136.126	69.021	878	1	868	0	CNL	same
At_BAK1	LRR-RLK	Lme	evm.model.scaffold565.260	68.982	619	9	615	0	LRR-RLK	same
At_RPP39	CNL	Lme	evm.model.scaffold482.70	68.558	617	1	616	0	CNL	same
At_RFO2	LRR-RLP	Lme	evm.model.scaffold34.95	68.516	721	28	748	0	LRR-RLK	different
At_NGR1a	RNL	Lme	evm.model.scaffold352.98	68.254	819	1	809	0	NL	different
At_RLP32	LRR-RLP	Lme	evm.model.scaffold384.176	67.857	420	428	840	0	LRR-RLP	same
At_RFO2	LRR-RLP	Lme	evm.model.scaffold299.729	66.899	716	28	743	0	LRR-RLP	same
At_RFO2	LRR-RLP	Lme	evm.model.scaffold245.123	66.197	710	28	737	0	LRR-RLP	same
At_RFO2	LRR-RLP	Lme	evm.model.scaffold937.13	65.994	694	49	740	0	LRR-RLP	same
At_NGR1b	RNL	Lme	evm.model.scaffold113.778	65.693	822	3	812	0	CNL	different
At_NGR1b	RNL	Lme	evm.model.scaffold52.101	65.69	819	6	812	0	NL	different
At_RPP39	CNL	Lme	evm.model.scaffold111.241	65.65	754	42	786	0	CNL	same
At_RPP8	CNL	Lme	evm.model.scaffold1913.8	65.57	912	1	908	0	CNL	same
At_RPP8	CNL	Lme	evm.model.scaffold433.231	64.803	912	1	904	0	CNL	same
At_RLP23	LRR-RLP	Lme	evm.model.scaffold146.396	64.575	717	175	889	0	LRR-RLP	same
At_RPP8	CNL	Lme	evm.model.scaffold81.519	64.388	907	1	903	0	CNL	same
At_RLM1b	TNL	Lme	evm.model.scaffold111.71	63.614	830	164	986	0	NL	different
At_RPP7	NL	Lme	evm.model.scaffold136.111	63.563	870	1	866	0	NL	same
At_RLM1b	TNL	Lme	evm.model.scaffold26.115	63.561	719	305	1013	0	NL	different
At_NGR1b	RNL	Lme	evm.model.scaffold352.98	63.471	824	3	812	0	NL	different
At_RLP23	LRR-RLP	Lme	evm.model.scaffold237.28	63.395	866	27	890	0	LRR-RLP	same
At_RPP39	CNL	Lme	evm.model.scaffold111.242	63.355	906	1	883	0	CNL	same
At_RPP39	CNL	Lme	evm.model.scaffold550.77	62.812	882	1	868	0	CNL	same
At_RLP42	LRR-RLP	Lme	evm.model.scaffold146.396	62.657	715	182	889	0	LRR-RLP	same
At_RPP39	CNL	Lme	evm.model.scaffold111.242	62.5	880	7	877	0	CNL	same
Bna_Rlm9/4/7	Other-RLK	Lme	evm.model.scaffold384.36	62.264	583	40	611	0	Other-RLK	same
At_RLP42	LRR-RLP	Lme	evm.model.scaffold237.28	62.251	853	33	880	0	LRR-RLP	same
At_RLP32	LRR-RLP	Lme	evm.model.scaffold316.302	62.195	492	362	849	0	LRR-RLP	same
Bna_Rlm9/4/7	Other-RLK	Lme	evm.model.scaffold384.39	61.864	590	22	604	0	Other-RLK	same
At_RLM1b	TNL	Lme	evm.model.scaffold34.933	61.725	742	249	979	0	NL	different
Bna_LepR3/Rlm2	LRR-RLP	Lme	evm.model.scaffold306.586	61.721	674	271	939	0	LRR-RLP	same
At_ADR1	NL	Lme	evm.model.scaffold60.203	61.557	809	6	778	0	NL	same
At_RLM1b	TNL	Lme	evm.model.scaffold971.193	61.406	754	257	999	0	NL	different
Bna_Rlm9/4/7	Other-RLK	Lme	evm.model.scaffold913.30	61.368	585	22	600	0	Other-RLK	same
At_ADR1	NL	Lme	evm.model.scaffold219.287	61.125	818	6	787	0	NL	same
At_RLP32	LRR-RLP	Lme	evm.model.scaffold339.98	61.12	643	211	844	0	LRR-RLP	same
Bna_LepR3/Rlm2	LRR-RLP	Lme	evm.model.scaffold117.254	60.65	892	55	941	0	LRR-RLP	same
Bna_LepR3/Rlm2	LRR-RLP	Lme	evm.model.scaffold339.98	60.62	645	300	931	0	LRR-RLP	same
At_RLP32	LRR-RLP	Lme	evm.model.scaffold306.586	60.597	670	186	854	0	LRR-RLP	same
Bna_Rlm9/4/7	Other-RLK	Lme	evm.model.scaffold215.217	60.441	589	25	602	0	Other-RLK	same
At_RLP30	LRR-RLP	Lme	evm.model.scaffold29.153	60.219	729	43	770	0	LRR-RLP	same
Bna_Rlm9/4/7	Other-RLK	Lme	evm.model.scaffold29.81	60.202	593	25	612	0	Other-RLK	same
At_RLP32	LRR-RLP	Lme	evm.model.scaffold37.460	60.094	639	203	841	0	LRR-RLP	same
At_RLM1b	TNL	Lme	evm.model.scaffold191.111	60.056	711	281	979	0	NL	different
At_RPS5	TNL	Lme	evm.model.scaffold50.106	60.041	493	123	613	0	NL	different
At_RLP32	LRR-RLP	Lme	evm.model.scaffold384.175	60.035	568	281	842	0	LRR-RLP	same
At_RPP39	CNL	Lme	evm.model.scaffold50.60	60.023	878	1	868	0	CNL	same
At_RPP1	TNL	Rra	RrC20884_p1	62.838	148	94	241	1.79E-59	TX	different
At_RPP1	TNL	Rra	RrC26085_p1	68.243	148	94	241	1.78E-67	TX	different
At_RRS1	TNL	Rra	RrC2503_p1	66.667	198	527	717	1.52E-70	TNL	same
At_RFO3	Other-RLK	Rra	RrC28417_p1	62.921	178	515	692	1.40E-73	Other-RLK	same
At_BAK1	LRR-RLK	Rra	RrC14965_p2	78	150	231	376	3.43E-76	Other-RLK	different
At_RLP32	LRR-RLP	Rra	RrC21259_p1	62.632	190	663	852	8.94E-77	LRR-RLP	same
Bra_Crr1a	TNL	Rra	RrC2202_p3	61.187	219	50	266	3.55E-80	TX	different
Bna_Rlm9/4/7	Other-RLK	Rra	RrC912_p3	68.528	197	542	738	2.15E-83	Other-RLK	same
Bra_Crr1a	TNL	Rra	RrC4724_p4	60.909	220	60	279	1.36E-84	TX	different
At_RPP1	TNL	Rra	RrC2202_p3	62.115	227	70	294	5.83E-86	TX	different
At_BAK1	LRR-RLK	Rra	RrC12485_p2	66.502	203	381	583	1.55E-89	LRR-RLK	same
Bna_Rlm9/4/7	Other-RLK	Rra	RrC502_p1	65.66	265	531	793	1.71E-90	Other-RLK	same
Bna_Rlm9/4/7	Other-RLK	Rra	RrC502_p1	66.791	268	527	793	1.35E-90	Other-RLK	same
At_RFO1	Other-RLK	Rra	RrC502_p1	61.479	257	500	750	2.22E-92	Other-RLK	same
At_RFO1	Other-RLK	Rra	RrC912_p3	75.13	193	515	705	1.59E-92	Other-RLK	same
Bra_cRa/cRb	TNL	Rra	RrC9740_p1	61.847	249	73	320	7.60E-93	TNL	same
At_RPP2a	TNL	Rra	RrC12579_p1	60.573	279	136	414	1.48E-93	TNL	same
At_RPP2a	TNL	Rra	RrC1201_p4	66.667	222	193	414	1.47E-93	NL	different
At_RFO1	Other-RLK	Rra	RrC502_p1	60.294	272	486	751	2.72E-94	Other-RLK	same
Bra_cRa/cRb	TNL	Rra	RrC10317_p3	60.87	253	57	308	1.17E-94	TX	different
Bna_LepR3/Rlm2	LRR-RLP	Rra	RrC24229_p1	65.957	235	718	950	3.45E-96	LRR-RLP	same

Bju_WRR1	CNL	Rra	RrC1918_p4	71.296	216	126	336	4.06E-98	NL	different
At_RPP8	CNL	Rra	RrC1918_p4	68.519	216	126	341	1.21E-99	NL	different
Bna_LepR3/Rlm2	LRR-RLP	Rra	RrC9458_p2	67.547	265	685	948	3.35E-102	LRR-RLP	same
At_RLP42	LRR-RLP	Rra	RrC7403_p1	63.855	249	487	731	2.60E-102	LRR-RLP	same
At_RLP32	LRR-RLP	Rra	RrC9458_p2	70.866	254	601	853	6.74E-105	LRR-RLP	same
Bra_Crr1a	TNL	Rra	RrC10317_p3	64.286	252	53	303	4.33E-105	TX	different
Bra_Crr1a	TNL	Rra	RrC9740_p1	64.683	252	65	315	1.21E-105	TNL	same
At_RPP1	TNL	Rra	RrC10317_p3	64.427	253	80	331	2.77E-108	TX	different
At_RLP23	LRR-RLP	Rra	RrC7403_p1	67.49	243	490	731	8.45E-109	LRR-RLP	same
At_RLM1a	TNL	Rra	RrC25189_p1	64.364	275	350	621	2.73E-109	TNL	same
At_RPP1	TNL	Rra	RrC9740_p1	65.079	252	93	343	2.02E-109	TNL	same
At_RLM1b	TNL	Rra	RrC25189_p1	62.366	279	350	626	8.66E-110	TNL	same
At_RPP2a	TNL	Rra	RrC11596_p1	66.787	277	138	414	9.81E-112	NL	different
At_RLP32	LRR-RLP	Rra	RrC24229_p1	75.676	222	634	854	1.88E-112	LRR-RLP	same
At_RPP2a	TNL	Rra	RrC190_p1	65.188	293	1007	1299	1.03E-114	TNL	same
At_RLP23	LRR-RLP	Rra	RrC30304_p1	64.151	318	290	607	3.02E-118	LRR-RLP	same
At_RLP42	LRR-RLP	Rra	RrC30304_p1	65.015	323	291	612	1.24E-123	LRR-RLP	same
At_ADR1	NL	Rra	RrC8290_p1	63.253	332	3	325	4.19E-133	NBS	different
At_RPP2a	TNL	Rra	RrC4901_p1	77.899	276	1025	1299	3.35E-138	TX	different
At_RPP8	CNL	Rra	RrC12445_p1	61.224	343	1	341	1.40E-138	CN	different
Bju_WRR1	CNL	Rra	RrC1918_p4	66.954	348	442	788	5.58E-142	NL	different
At_NGR1b	RNL	Rra	RrC30918_p1	63.818	351	218	558	8.82E-146	NBS	different
At_RLP42	LRR-RLP	Rra	RrC13278_p1	60.95	379	495	873	2.58E-151	LRR-RLP	same
At_PBS1	Other-RLK	Rra	RrC3989_p1	67.203	311	71	381	5.45E-158	Other-RLK	same
At_PBS1	Other-RLK	Rra	RrC2330_p3	71.572	299	71	369	1.47E-158	Other-RLK	same
At_RLP23	LRR-RLP	Rra	RrC13278_p1	63.959	394	495	888	2.62E-162	LRR-RLP	same
At_NGR1a	RNL	Rra	RrC30918_p1	72.206	349	216	552	1.23E-169	NBS	different
At_PBS1	Other-RLK	Rra	RrC2463_p6	77.778	306	65	370	9.29E-174	Other-RLK	same
At_BAK1	LRR-RLK	Rra	RrC2766_p1	96.063	508	1	508	0	LRR-RLK	same
Bol_FocBo1	TNL	Rra	RrC1201_p4	95.563	586	193	778	0	NL	different
At_BAK1	LRR-RLK	Rra	RrC645_p6	93.453	611	5	615	0	LRR-RLK	same
At_RPS2	NL	Rra	RrC1874_p4	84.505	910	1	909	0	CNL	different
Bna_Rlm9/4/7	Other-RLK	Rra	RrC858_p5	82.412	796	1	794	0	Other-RLK	same
At_BAK1	LRR-RLK	Rra	RrC345_p11	82.178	606	10	615	0	LRR-RLK	same
At_RPM1	NL	Rra	RrC828_p2	82.12	934	1	926	0	NL	same
At_SOBIR1	LRR-RLK	Rra	RrC129_p7	80.371	647	1	640	0	LRR-RLK	same
At_SOBIR1	LRR-RLK	Rra	RrC10596_p2	79.888	358	284	641	0	Other-RLK	different
At_WRR12	TNL	Rra	RrC2369_p3	78.414	681	372	1049	0	TX	different
At_RPS5	TNL	Rra	RrC20133_p1	78.219	730	1	730	0	NL	different
At_BAK1	LRR-RLK	Rra	RrC2772_p1	78.161	609	10	615	0	LRR-RLK	same
Bna_LepR3/Rlm2	LRR-RLP	Rra	RrC1351_p1	77.708	951	1	945	0	LRR-RLP	same
At_BAK1	LRR-RLK	Rra	RrC59_p5	77.346	618	24	615	0	LRR-RLK	same
At_NGR1a	RNL	Rra	RrC2329_p4	77.017	818	1	809	0	NL	different
At_RPS5	TNL	Rra	RrC3583_p3	76.313	895	1	889	0	NL	different
At_RFO3	Other-RLK	Rra	RrC4728_p1	75.793	851	19	850	0	Other-RLK	same
At_BAK1	LRR-RLK	Rra	RrC10357_p1	71.933	652	8	615	0	LRR-RLK	same
At_RFO1	Other-RLK	Rra	RrC22170_p1	70.615	667	96	751	0	Other-RLK	same
Bna_LepR3/Rlm2	LRR-RLP	Rra	RrC11009_p1	69.374	911	31	938	0	LRR-RLP	same
At_RFO2	LRR-RLP	Rra	RrC7931_p1	68.511	705	33	737	0	LRR-RLK	different
At_RFO2	LRR-RLP	Rra	RrC23063_p1	68.486	733	8	740	0	LRR-RLP	same
At_NGR1b	RNL	Rra	RrC2329_p4	68.088	821	5	815	0	NL	different
At_RLP42	LRR-RLP	Rra	RrC10614_p1	67.614	880	1	876	0	LRR-RLP	same
At_RPP8	CNL	Rra	RrC9934_p1	67.161	944	1	907	0	CNL	same
At_RPP13	CNL	Rra	RrC1706_p2	66.585	814	1	789	0	NL	different
At_RPS5	TNL	Rra	RrC29576_p1	66.46	483	227	706	0	NL	different
At_RLP23	LRR-RLP	Rra	RrC10614_p1	66.069	893	1	890	0	LRR-RLP	same
At_RFO2	LRR-RLP	Rra	RrC4219_p1	65.694	720	32	748	0	LRR-RLK	different
At_RFO2	LRR-RLP	Rra	RrC4575_p1	65.328	747	6	742	0	LRR-RLP	same
At_RLP32	LRR-RLP	Rra	RrC2862_p1	64.933	750	118	865	0	LRR-RLP	same
Bju_WRR1	CNL	Rra	RrC1918_p1	64.34	530	376	902	0	NL	different
At_RLM1a	TNL	Rra	RrC25117_p1	63.489	493	132	622	0	TNL	same
At_RPP13	CNL	Rra	RrC1706_p3	63.244	857	1	829	0	NL	different
Bna_Rlm9/4/7	Other-RLK	Rra	RrC25334_p1	63.189	508	159	660	0	Other-RLK	same
At_RLP23	LRR-RLP	Rra	RrC15950_p1	62.901	717	175	889	0	LRR-RLP	same
At_RFO1	Other-RLK	Rra	RrC5399_p1	62.881	722	7	712	0	Other-RLK	same
At_RPS5	TNL	Rra	RrC3098_p2	62.851	891	1	880	0	CNL	different
At_RFO1	Other-RLK	Rra	RrC5399_p2	62.418	761	4	751	0	Other-RLK	same
At_WRR8	TNL	Rra	RrC13073_p2	62.397	609	170	769	0	NL	different
At_RLP42	LRR-RLP	Rra	RrC15950_p1	62.34	701	182	880	0	LRR-RLP	same
At_RPP2a	TNL	Rra	RrC4901_p1	62.208	471	408	866	0	TX	different
At_ADR1	NL	Rra	RrC12655_p2	62.088	728	91	787	0	NL	same
At_RLP32	LRR-RLP	Rra	RrC7190_p1	61.13	885	33	853	0	LRR-RLP	same
At_RPS5	TNL	Rra	RrC2474_p3	60.992	887	1	881	0	CNL	different
At_RPP39	CNL	Rra	RrC197_p3	60.987	892	3	885	0	CNL	same
At_RAC1	TNL	Rra	RrC409_p7	60.737	624	9	623	0	TNL	same
At_WRR8	TNL	Rra	RrC409_p7	60.692	636	7	634	0	TNL	same
At_RAC1	TNL	Rra	RrC13073_p2	60.362	608	160	760	0	NL	different
At_WRR9	NL	Rra	RrC25117_p1	60.324	494	136	627	0	TNL	different
At_RLP23	LRR-RLP	Rsa	Rsa1.0_00111.1_g00007.1	64.968	157	723	879	3.40E-60	LRR-RLP	same
At_RLP30	LRR-RLP	Rsa	Rsa1.0_01839.1_g00004.1	62.162	185	602	786	2.53E-61	LRR-RLP	same
At_RLP42	LRR-RLP	Rsa	Rsa1.0_00111.1_g00007.1	67.55	151	723	873	7.75E-63	LRR-RLP	same
At_RAC1	TNL	Rsa	Rsa1.0_00338.1_g00001.1	61.927	218	8	222	5.10E-75	TNL	same
Bol_FocBo1	TNL	Rsa	Rsa1.0_03626.1_g00002.1	62.613	222	13	233	3.38E-78	TX	different
At_WRR8	TNL	Rsa	Rsa1.0_00338.1_g00001.1	64.977	217	19	232	7.08E-81	TNL	same
Bna_Rlm9/4/7	Other-RLK	Rsa	Rsa1.0_11429.1_g00001.1	70.659	167	386	551	1.15E-82	Other-RLK	same
At_RAC1	TNL	Rsa	Rsa1.0_03626.1_g00002.1	69.658	234	20	249	2.04E-91	TX	different

At_RFO1	Other-RLK	Rsa	Rsa1.0_11429.1_g00001.1	79.762	168	352	518	1.10E-93	Other-RLK	same
At_WRR8	TNL	Rsa	Rsa1.0_03626.1_g00002.1	63.396	265	31	283	3.96E-95	TX	different
At_WRR4a	TNL	Rsa	Rsa1.0_09247.1_g00001.1	62.771	231	5	235	1.87E-98	TX	different
Bna_LepR3/Rlm2	LRR-RLP	Rsa	Rsa1.0_00016.1_g00027.1	70.635	252	1	249	8.73E-99	LRR-RLP	same
At_WRR4b	TNL	Rsa	Rsa1.0_13731.1_g00001.1	68.056	216	7	222	1.64E-100	TX	different
At_RLM1a	TNL	Rsa	Rsa1.0_09247.1_g00001.1	63.983	236	1	236	4.49E-103	TX	different
At_WRR4b	TNL	Rsa	Rsa1.0_09247.1_g00001.1	68.398	231	7	237	1.05E-107	TX	different
At_RFO1	Other-RLK	Rsa	Rsa1.0_25697.1_g00001.1	64.041	292	240	519	4.74E-111	Other-RLK	same
At_RPP1	TNL	Rsa	Rsa1.0_01705.1_g00006.1	65.056	269	94	362	2.00E-114	TNL	same
At_RLM1a	TNL	Rsa	Rsa1.0_12238.1_g00001.1	61.922	281	301	581	2.01E-115	NBS	different
Bra_cRa/cRb	TNL	Rsa	Rsa1.0_01705.1_g00006.1	60.294	340	1	339	2.51E-118	TNL	same
Bra_Crr1a	TNL	Rsa	Rsa1.0_01705.1_g00006.1	66.904	281	68	347	1.34E-119	TNL	same
At_RLM1a	TNL	Rsa	Rsa1.0_24800.1_g00001.1	63.701	281	301	581	8.91E-120	NBS	different
At_PBS1	Other-RLK	Rsa	Rsa1.0_04364.1_g00001.1	62.112	322	74	391	5.71E-138	Other-RLK	same
At_RPS5	TNL	Rsa	Rsa1.0_22598.1_g00001.1	64.22	327	308	631	2.16E-139	NL	different
At_RPP4	TNL	Rsa	Rsa1.0_09437.1_g00001.1	60.92	435	690	1113	8.91E-146	TNL	same
At_ADR1	NL	Rsa	Rsa1.0_00127.1_g00035.1	65.607	346	445	787	5.53E-151	NL	same
Bra_cRa/cRb	TNL	Rsa	Rsa1.0_00027.1_g00036.1	61.456	371	54	423	1.04E-151	TN	different
At_PBS1	Other-RLK	Rsa	Rsa1.0_00209.1_g00001.1	66.881	311	71	381	5.97E-157	Other-RLK	same
Bra_cRa/cRb	TNL	Rsa	Rsa1.0_00527.1_g00014.1	60.804	398	75	470	3.02E-159	TNL	same
Bra_Crr1a	TNL	Rsa	Rsa1.0_00027.1_g00036.1	63.686	369	60	426	3.56E-163	TN	different
At_RPP1	TNL	Rsa	Rsa1.0_00027.1_g00036.1	62.228	368	81	447	5.55E-164	TN	different
At_RPP2a	TNL	Rsa	Rsa1.0_04369.1_g00006.1	64.976	414	1	414	9.34E-171	TNL	same
Bra_Crr1a	TNL	Rsa	Rsa1.0_00527.1_g00014.1	62.47	421	50	465	5.90E-171	TNL	same
At_WRR4a	TNL	Rsa	Rsa1.0_08371.1_g00001.1	62.037	432	1	427	5.16E-173	TN	different
At_RPP1	TNL	Rsa	Rsa1.0_00527.1_g00014.1	61.033	426	72	493	5.03E-173	TNL	same
At_RLM3	TN	Rsa	Rsa1.0_00524.1_g00004.1	60.088	456	4	450	1.71E-175	TNL	different
At_RLM3	TN	Rsa	Rsa1.0_09437.1_g00001.1	60.486	453	3	445	3.28E-177	TNL	different
At_RPP2a	TNL	Rsa	Rsa1.0_00524.1_g00004.1	66.912	408	8	414	2.94E-177	TNL	same
At_BAK1	LRR-RLK	Rsa	Rsa1.0_00237.1_g00015.1	94.634	615	1	615	0	LRR-RLK	same
At_BAK1	LRR-RLK	Rsa	Rsa1.0_00127.1_g00028.1	94.435	611	5	615	0	LRR-RLK	same
At_BAK1	LRR-RLK	Rsa	Rsa1.0_00374.1_g00016.1	93.781	611	5	615	0	LRR-RLK	same
Bol_FocBo1	TNL	Rsa	Rsa1.0_00524.1_g00004.1	90.171	1343	1	1340	0	TNL	same
At_RFO1	Other-RLK	Rsa	Rsa1.0_01251.1_g00006.1	85.433	762	1	751	0	Other-RLK	same
At_RPS2	NL	Rsa	Rsa1.0_00102.1_g00007.1	84.396	910	1	909	0	CNL	different
Bna_Rlm9/4/7	Other-RLK	Rsa	Rsa1.0_05858.1_g00001.1	82.412	796	1	794	0	Other-RLK	same
Bna_Rlm9/4/7	Other-RLK	Rsa	Rsa1.0_14156.1_g00002.1	82.016	506	23	528	0	Other-RLK	same
At_WRR12	TNL	Rsa	Rsa1.0_00160.1_g00015.1	81.922	1051	6	1049	0	TNL	same
At_SOBIR1	LRR-RLK	Rsa	Rsa1.0_08551.1_g00001.1	81.123	641	3	640	0	LRR-RLK	same
At_RLP1	LRR-RLP	Rsa	Rsa1.0_00689.1_g00004.1	80.855	1076	1	1076	0	LRR-RLP	same
Bna_LepR3/Rlm2	LRR-RLP	Rsa	Rsa1.0_00016.1_g00027.1	80.534	637	317	946	0	LRR-RLP	same
At_RPM1	NL	Rsa	Rsa1.0_00030.1_g00049.1	80.086	934	1	926	0	NL	same
At_BAK1	LRR-RLK	Rsa	Rsa1.0_03231.1_g00003.1	79.373	606	24	615	0	LRR-RLK	same
At_FLS2	LRR-RLK	Rsa	Rsa1.0_00154.1_g00021.1	78.455	1165	1	1160	0	LRR-RLK	same
At_BAK1	LRR-RLK	Rsa	Rsa1.0_01255.1_g00009.1	78.361	610	8	615	0	LRR-RLK	same
At_BAK1	LRR-RLK	Rsa	Rsa1.0_03481.1_g00005.1	78.161	609	10	615	0	LRR-RLK	same
At_SOBIR1	LRR-RLK	Rsa	Rsa1.0_04268.1_g00001.1	78.14	645	1	641	0	LRR-RLK	same
At_NGR1a	RNL	Rsa	Rsa1.0_01073.1_g00007.1	76.801	819	1	809	0	CNL	different
At_RLM1a	TNL	Rsa	Rsa1.0_03206.1_g00002.1	75.926	378	11	385	0	TNL	same
At_RPS4	TNL	Rsa	Rsa1.0_00091.1_g00018.1	75.598	1213	1	1198	0	TNL	same
At_RFO3	Other-RLK	Rsa	Rsa1.0_00604.1_g00002.1	75.543	875	2	850	0	Other-RLK	same
At_RFO1	Other-RLK	Rsa	Rsa1.0_01004.1_g00008.1	73.969	776	1	751	0	Other-RLK	same
At_RLP32	LRR-RLP	Rsa	Rsa1.0_00013.1_g00043.1	73.818	867	1	864	0	LRR-RLP	same
At_RFO3	Other-RLK	Rsa	Rsa1.0_00628.1_g00003.1	73.557	866	8	839	0	Other-RLK	same
At_BAK1	LRR-RLK	Rsa	Rsa1.0_00140.1_g00002.1	73.52	608	10	615	0	LRR-RLK	same
At_RPP2b	TNL	Rsa	Rsa1.0_02787.1_g00003.1	73.519	1182	16	1193	0	TNL	same
At_RLM1b	TNL	Rsa	Rsa1.0_17859.1_g00001.1	73.499	483	1	474	0	TN	different
At_RLM1b	TNL	Rsa	Rsa1.0_03206.1_g00002.1	72.751	378	11	385	0	TNL	same
Bra_cRa/cRb	TNL	Rsa	Rsa1.0_01124.1_g00005.1	70.964	954	70	1018	0	Other-NLR	different
At_RLM1a	TNL	Rsa	Rsa1.0_08371.1_g00001.1	69.907	432	1	430	0	TN	different
Bju_WRR1	CNL	Rsa	Rsa1.0_01628.1_g00005.1	69.197	909	1	900	0	CNL	same
At_RPS4	TNL	Rsa	Rsa1.0_00259.1_g00003.1	68.918	1229	1	1217	0	TNL	same
At_RFO2	LRR-RLP	Rsa	Rsa1.0_01463.1_g00005.1	68.794	705	33	737	0	LRR-RLK	different
At_RPP8	CNL	Rsa	Rsa1.0_00851.1_g00013.1	68.736	870	1	863	0	CNL	same
At_RPP2a	TNL	Rsa	Rsa1.0_09437.1_g00001.1	68.599	414	1	414	0	TNL	same
At_NGR1b	RNL	Rsa	Rsa1.0_01073.1_g00007.1	68.127	822	5	815	0	CNL	different
Bna_Rlm9/4/7	Other-RLK	Rsa	Rsa1.0_01251.1_g00005.1	67.526	776	24	794	0	Other-RLK	same
At_RPP2a	TNL	Rsa	Rsa1.0_02787.1_g00004.1	67.342	888	415	1299	0	NL	different
At_RPP2b	TNL	Rsa	Rsa1.0_01450.1_g00003.1	67.303	1153	1	1145	0	Other-NLR	different
At_RRS1	TNL	Rsa	Rsa1.0_00091.1_g00017.1	67.006	1179	1	1169	0	TNL	same
At_WRR4b	TNL	Rsa	Rsa1.0_08371.1_g00001.1	66.821	431	3	430	0	TN	different
At_RPP13	CNL	Rsa	Rsa1.0_01236.1_g00007.1	66.628	857	1	829	0	NL	different
At_RPP13	CNL	Rsa	Rsa1.0_04054.1_g00004.1	66.55	855	1	832	0	CNL	same
At_RFO2	LRR-RLP	Rsa	Rsa1.0_02133.1_g00001.1	66.147	706	33	737	0	LRR-RLP	same
At_RFO2	LRR-RLP	Rsa	Rsa1.0_00396.1_g00016.1	65.833	720	32	748	0	LRR-RLK	different
At_RPS5	TNL	Rsa	Rsa1.0_15163.1_g00001.1	65.801	462	1	462	0	CN	different
At_RLM1b	TNL	Rsa	Rsa1.0_08371.1_g00001.1	65.509	432	1	430	0	TN	different
At_RLM1a	TNL	Rsa	Rsa1.0_17859.1_g00001.1	65.424	483	1	474	0	TN	different
At_ADR1	NL	Rsa	Rsa1.0_07850.1_g00001.1	65.327	796	3	787	0	CNL	different
At_RPS5	TNL	Rsa	Rsa1.0_19402.1_g00001.1	65.198	454	138	591	0	NL	different
At_RFO1	Other-RLK	Rsa	Rsa1.0_06912.1_g00003.1	64.986	714	7	705	0	Other-RLK	same
At_RLP42	LRR-RLP	Rsa	Rsa1.0_00111.1_g00003.1	64.892	883	1	876	0	LRR-RLP	same
At_RLP32	LRR-RLP	Rsa	Rsa1.0_00016.1_g00027.1	64.815	648	228	864	0	LRR-RLP	same
At_RLP32	LRR-RLP	Rsa	Rsa1.0_01434.1_g00013.1	64.683	756	114	854	0	LRR-RLP	same
At_RLM1b	TNL	Rsa	Rsa1.0_04941.1_g00001.1	64.599	935	1	925	0	Other-NLR	different
At_RLP23	LRR-RLP	Rsa	Rsa1.0_00111.1_g00003.1	64.541	894	1	890	0	LRR-RLP	same

At_RRS1	TNL	Rsa	Rsa1.0_03896.1_g00002.1	64.092	1178	1	1168	0	TNL	same
At_RPS5	TNL	Rsa	Rsa1.0_01327.1_g00007.1	63.729	885	1	880	0	CNL	different
At_RLM1a	TNL	Rsa	Rsa1.0_07116.1_g00001.1	63.592	618	10	621	0	TNL	same
Bra_cRa/cRb	TNL	Rsa	Rsa1.0_01124.1_g00006.1	63.591	1398	1	1322	0	TNL	same
At_WRR9	NL	Rsa	Rsa1.0_08371.1_g00001.1	63.426	432	5	434	0	TN	different
At_RLP32	LRR-RLP	Rsa	Rsa1.0_02149.1_g00003.1	63.221	745	117	852	0	LRR-RLP	same
At_RLP23	LRR-RLP	Rsa	Rsa1.0_05999.1_g00003.1	63.21	704	175	876	0	LRR-RLP	same
At_RPP5	TNL	Rsa	Rsa1.0_04369.1_g00006.1	63	700	3	671	0	TNL	same
At_RLM1a	TNL	Rsa	Rsa1.0_04941.1_g00001.1	62.514	923	3	917	0	Other-NLR	different
At_RLP42	LRR-RLP	Rsa	Rsa1.0_05999.1_g00003.1	62.428	692	182	870	0	LRR-RLP	same
At_RLP23	LRR-RLP	Rsa	Rsa1.0_01458.1_g00003.1	62.361	720	175	889	0	LRR-RLP	same
At_RLP23	LRR-RLP	Rsa	Rsa1.0_05999.1_g00001.1	62.343	717	175	889	0	LRR-RLP	same
At_WRR9	NL	Rsa	Rsa1.0_17859.1_g00001.1	62.268	485	5	479	0	TN	different
Bna_LepR3/Rlm2	LRR-RLP	Rsa	Rsa1.0_02149.1_g00003.1	62.199	955	1	944	0	LRR-RLP	same
At_WRR4b	TNL	Rsa	Rsa1.0_17859.1_g00001.1	62.112	483	1	474	0	TN	different
At_RLM1b	TNL	Rsa	Rsa1.0_00662.1_g00013.1	61.857	991	1	975	0	TNL	same
Bra_Crr1a	TNL	Rsa	Rsa1.0_13327.1_g00001.1	61.653	472	220	687	0	NL	different
At_RLP42	LRR-RLP	Rsa	Rsa1.0_05999.1_g00001.1	61.626	701	182	880	0	LRR-RLP	same
At_RLP42	LRR-RLP	Rsa	Rsa1.0_01458.1_g00003.1	61.581	721	182	889	0	LRR-RLP	same
At_RLP23	LRR-RLP	Rsa	Rsa1.0_01339.1_g00008.1	61.574	864	17	877	0	LRR-RLP	same
At_RPP39	CNL	Rsa	Rsa1.0_00039.1_g00041.1	61.521	881	1	870	0	CNL	same
Bju_WRR1	CNL	Rsa	Rsa1.0_00851.1_g00013.1	61.495	883	1	860	0	CNL	same
At_RPS5	TNL	Rsa	Rsa1.0_04072.1_g00002.1	61.453	895	1	883	0	NL	different
At_RLM1b	TNL	Rsa	Rsa1.0_04941.1_g00004.1	61.442	1040	1	1014	0	TNL	same
Bol_FocBo1	TNL	Rsa	Rsa1.0_09437.1_g00001.1	61.287	979	5	981	0	TNL	same
At_RPP8	CNL	Rsa	Rsa1.0_01628.1_g00005.1	61.05	914	1	903	0	CNL	same
At_RLP42	LRR-RLP	Rsa	Rsa1.0_04036.1_g00004.1	60.978	879	1	876	0	LRR-RLP	same
At_RLM1b	TNL	Rsa	Rsa1.0_10052.1_g00001.1	60.955	963	1	949	0	TNL	same
Bra_Crr1a	TNL	Rsa	Rsa1.0_03069.1_g00004.1	60.928	1272	52	1214	0	TNL	same
At_RPS5	TNL	Rsa	Rsa1.0_00018.1_g00052.1	60.879	887	1	881	0	CNL	different
At_ADR1	NL	Rsa	Rsa1.0_00123.1_g00044.1	60.811	814	6	787	0	NL	same
At_RLP42	LRR-RLP	Rsa	Rsa1.0_01339.1_g00008.1	60.748	856	19	871	0	LRR-RLP	same
Bol_FocBo1	TNL	Rsa	Rsa1.0_02434.1_g00005.1	60.68	618	7	618	0	Other-NLR	different
Bra_Crr1a	TNL	Rsa	Rsa1.0_01124.1_g00005.1	60.652	1258	1	1224	0	Other-NLR	different
At_RPS5	TNL	Rsa	Rsa1.0_01079.1_g00004.1	60.595	840	43	880	0	NL	different
At_RLP32	LRR-RLP	Rsa	Rsa1.0_00179.1_g00063.1	60.552	905	1	866	0	LRR-RLP	same
At_RLM1b	TNL	Rsa	Rsa1.0_04344.1_g00002.1	60.537	1006	1	993	0	TNL	same
At_RLM1b	TNL	Rsa	Rsa1.0_07116.1_g00001.1	60.514	623	10	626	0	TNL	same
At_RPP2a	TNL	Rsa	Rsa1.0_01450.1_g00006.1	60.394	813	494	1299	0	TNL	same
At_RLP23	LRR-RLP	Rsa	Rsa1.0_04036.1_g00004.1	60.223	895	1	890	0	LRR-RLP	same
At_WRR4b	TNL	Rsa	Rsa1.0_07116.1_g00001.1	60.161	620	11	623	0	TNL	same
At_ADR1	NL	Sir	SI_scaffold249_14	69.554	808	3	787	0	CNL	different
At_ADR1	NL	Sir	SI_C228743_3	61.481	810	6	787	0	NL	same
At_ADR1	NL	Sir	SI_scaffold669_157	60.292	821	3	787	0	NL	same
At_BAK1	LRR-RLK	Sir	SI_scaffold669_147	91.473	645	1	615	0	LRR-RLK	same
At_BAK1	LRR-RLK	Sir	SI_scaffold362_6	79.579	617	1	615	0	LRR-RLK	same
At_BAK1	LRR-RLK	Sir	SI_scaffold1058_17	77.541	610	24	615	0	LRR-RLK	same
At_BAK1	LRR-RLK	Sir	SI_scaffold161_3	77.377	610	20	615	0	LRR-RLK	same
At_BAK1	LRR-RLK	Sir	SI_C231345_8	77.377	610	20	615	0	LRR-RLK	same
At_BAK1	LRR-RLK	Sir	SI_C230793_3	77.377	610	20	615	0	LRR-RLK	same
At_BAK1	LRR-RLK	Sir	SI_C217599_3	74.281	591	30	615	0	LRR-RLK	same
At_FLS2	LRR-RLK	Sir	SI_scaffold1724_31	81.787	1164	1	1160	0	LRR-RLK	same
At_FLS2	LRR-RLK	Sir	SI_scaffold1724_32	70.3	1165	23	1159	0	LRR-RLK	same
At_NGR1a	RNL	Sir	SI_scaffold2426_217	77.178	815	1	809	0	NL	different
At_NGR1a	RNL	Sir	SI_scaffold2426_218	66.141	824	5	809	0	CNL	different
At_NGR1a	RNL	Sir	SI_scaffold2426_219	65.617	413	404	809	0	NL	different
At_NGR1b	RNL	Sir	SI_scaffold2426_219	61.779	416	406	815	1.32E-168	NL	different
At_NGR1b	RNL	Sir	SI_scaffold2426_217	67.476	824	3	815	0	NL	different
At_NGR1b	RNL	Sir	SI_scaffold2426_218	62.606	829	9	815	0	CNL	different
At_PBS1	Other-RLK	Sir	SI_scaffold123_149	74.528	318	69	370	2.76E-168	Other-RLK	same
At_RAC1	TNL	Sir	SI_scaffold1724_46	64.977	217	2	215	5.32E-81	TNL	same
At_RAC1	TNL	Sir	SI_scaffold877_4	61.328	256	1	253	1.44E-91	TN	different
At_RAC1	TNL	Sir	SI_scaffold862_17	60.392	919	12	920	0	TNL	same
At_RFO1	Other-RLK	Sir	SI_scaffold2049_127	87.957	739	18	751	0	Other-RLK	same
At_RFO1	Other-RLK	Sir	SI_scaffold1491_128	63.649	729	32	742	0	Other-RLK	same
At_RFO1	Other-RLK	Sir	SI_scaffold1491_129	61.235	761	10	745	0	Other-RLK	same
At_RFO1	Other-RLK	Sir	SI_scaffold1491_127	60.166	723	19	725	0	Other-RLK	same
At_RFO2	LRR-RLP	Sir	SI_scaffold242_77	72.073	709	29	737	0	LRR-RLK	different
At_RFO2	LRR-RLP	Sir	SI_scaffold227_27	69.429	736	6	740	0	LRR-RLP	same
At_RFO3	Other-RLK	Sir	SI_scaffold744_139	76.157	864	1	850	0	Other-RLK	same
At_RFO3	Other-RLK	Sir	SI_scaffold741_12	68.167	933	1	850	0	Other-RLK	same
At_RLM1a	TNL	Sir	SI_scaffold436_1	62.987	154	5	158	5.68E-58	TX	different
At_RLM1a	TNL	Sir	SI_scaffold325_22	70.064	157	1	157	5.89E-60	TNL	same
At_RLM1a	TNL	Sir	SI_scaffold1338_12	66.184	207	13	219	1.01E-83	TNL	same
At_RLM1a	TNL	Sir	SI_scaffold2804_1	74.879	207	11	217	1.76E-95	TNL	same
At_RLM1a	TNL	Sir	SI_scaffold1847_1	64.977	217	3	219	1.10E-95	TX	different
At_RLM1a	TNL	Sir	SI_scaffold1174_8	65.723	318	38	355	1.41E-142	TN	different
At_RLM1a	TNL	Sir	SI_scaffold146_3	72.022	361	160	519	1.25E-172	NL	different
At_RLM1a	TNL	Sir	SI_scaffold146_21	68.727	534	8	539	0	TNL	same
At_RLM1a	TNL	Sir	SI_scaffold146_4	62.901	903	3	896	0	TNL	same
At_RLM1a	TNL	Sir	SI_scaffold325_13	60.768	989	10	982	0	Other-NLR	different
At_RLM1a	TNL	Sir	SI_scaffold325_17	60.512	937	10	944	0	TNL	same
At_RLM1a	TNL	Sir	SI_scaffold325_14	60.02	993	1	986	0	TNL	same
At_RLM1b	TNL	Sir	SI_scaffold1338_12	61.836	207	13	219	6.28E-75	TNL	same
At_RLM1b	TNL	Sir	SI_scaffold2804_1	61.25	240	14	253	2.76E-84	TNL	same
At_RLM1b	TNL	Sir	SI_scaffold1847_1	62.673	217	4	219	4.49E-88	TX	different

At_RLM1b	TNL	Sir	SI_scaffold1882_1	83.784	185	1	182	4.04E-102	TX	different
At_RLM1b	TNL	Sir	SI_scaffold1174_8	67.619	315	41	355	4.02E-145	TN	different
At_RLM1b	TNL	Sir	SI_scaffold146_3	67.391	368	160	526	3.18E-160	NL	different
At_RLM1b	TNL	Sir	SI_scaffold146_21	68.468	555	8	556	0	TNL	same
At_RLM1b	TNL	Sir	SI_scaffold325_14	66.269	1005	1	994	0	TNL	same
At_RLM1b	TNL	Sir	SI_scaffold325_15	65.823	1027	1	1009	0	TNL	same
At_RLM1b	TNL	Sir	SI_scaffold325_14	63.718	1006	11	998	0	TNL	same
At_RLM1b	TNL	Sir	SI_scaffold325_12	62.762	1050	1	1012	0	TNL	same
At_RLM1b	TNL	Sir	SI_scaffold325_13	62.735	1009	10	997	0	Other-NLR	different
At_RLM1b	TNL	Sir	SI_scaffold146_4	61.531	1032	3	1014	0	TNL	same
At_RLP1	LRR-RLP	Sir	SI_scaffold2746_207	74.299	1070	24	1067	0	LRR-RLP	same
At_RLP1	LRR-RLP	Sir	SI_scaffold2881_1	64.49	490	567	1055	0	LRR-RLP	same
At_RLP23	LRR-RLP	Sir	SI_scaffold651_19	68.806	888	1	884	0	LRR-RLP	same
At_RLP23	LRR-RLP	Sir	SI_scaffold1453_8	65.303	611	175	780	0	LRR-RLP	same
At_RLP23	LRR-RLP	Sir	SI_scaffold718_53	63.18	717	175	889	0	LRR-RLP	same
At_RLP23	LRR-RLP	Sir	SI_scaffold154_84	62.535	718	175	890	0	LRR-RLP	same
At_RLP23	LRR-RLP	Sir	SI_scaffold1453_7	61.389	720	175	889	0	LRR-RLP	same
At_RLP23	LRR-RLP	Sir	SI_scaffold1417_16	60.728	522	1	520	0	LRR-RLP	same
At_RLP30	LRR-RLP	Sir	SI_scaffold857_5	61.161	775	17	786	0	LRR-RLP	same
At_RLP30	LRR-RLP	Sir	SI_scaffold930_20	60.696	776	1	769	0	LRR-RLP	same
At_RLP30	LRR-RLP	Sir	SI_scaffold1097_6	60.181	663	100	760	0	LRR-RLP	same
At_RLP32	LRR-RLP	Sir	SI_scaffold2443_28	66.517	669	187	853	0	LRR-RLP	same
At_RLP32	LRR-RLP	Sir	SI_C228887_1	66.517	669	187	853	0	LRR-RLP	same
At_RLP32	LRR-RLP	Sir	SI_scaffold1201_3	65.984	635	218	852	0	LRR-RLP	same
At_RLP32	LRR-RLP	Sir	SI_C226829_1	65.639	876	1	850	0	LRR-RLP	same
At_RLP32	LRR-RLP	Sir	SI_scaffold712_15	64.486	749	114	854	0	LRR-RLP	same
At_RLP32	LRR-RLP	Sir	SI_scaffold1201_18	64.347	819	69	846	0	LRR-RLP	same
At_RLP32	LRR-RLP	Sir	SI_scaffold651_14	63.594	857	9	853	0	LRR-RLP	same
At_RLP32	LRR-RLP	Sir	SI_scaffold429_15	63.587	736	117	850	0	LRR-RLP	same
At_RLP32	LRR-RLP	Sir	SI_scaffold2390_5	62.251	853	1	844	0	LRR-RLP	same
At_RLP32	LRR-RLP	Sir	SI_scaffold651_24	62.034	885	28	854	0	LRR-RLP	same
At_RLP32	LRR-RLP	Sir	SI_C228331_1	62.034	885	28	854	0	LRR-RLP	same
At_RLP32	LRR-RLP	Sir	SI_C225375_3	62.034	885	28	854	0	LRR-RLP	same
At_RLP32	LRR-RLP	Sir	SI_scaffold651_19	61.893	824	36	853	0	LRR-RLP	same
At_RLP32	LRR-RLP	Sir	SI_scaffold2443_26	61.705	833	69	831	0	LRR-RLK	different
At_RLP32	LRR-RLP	Sir	SI_scaffold651_20	60.343	875	14	841	0	LRR-RLP	same
At_RLP42	LRR-RLP	Sir	SI_scaffold1417_16	61.377	523	1	520	1.25E-174	LRR-RLP	same
At_RLP42	LRR-RLP	Sir	SI_scaffold651_19	68.601	879	1	876	0	LRR-RLP	same
At_RLP42	LRR-RLP	Sir	SI_scaffold1453_8	63.562	612	175	780	0	LRR-RLP	same
At_RLP42	LRR-RLP	Sir	SI_scaffold718_53	62	700	182	876	0	LRR-RLP	same
At_RLP42	LRR-RLP	Sir	SI_scaffold154_84	61.396	702	183	880	0	LRR-RLP	same
At_RPP1	TNL	Sir	SI_scaffold1847_1	60.577	208	97	304	4.32E-85	TX	different
At_RPP1	TNL	Sir	SI_scaffold158_1	60.42	619	95	711	0	TNL	same
At_RPP13	CNL	Sir	SI_scaffold592_1	65.285	386	1	384	2.38E-145	NBS	different
At_RPP2a	TNL	Sir	SI_C227795_1	62.319	414	3	414	4.16E-162	TNL	same
At_RPP2a	TNL	Sir	SI_scaffold842_25	62.155	399	11	409	3.61E-166	TN	different
At_RPP2a	TNL	Sir	SI_scaffold842_29	68.719	406	10	414	1.36E-171	TNL	same
At_RPP2a	TNL	Sir	SI_scaffold930_156	71.742	821	480	1299	0	NL	different
At_RPP2b	TNL	Sir	SI_scaffold930_157	71.263	1211	3	1183	0	TNL	same
At_RPP39	CNL	Sir	SI_scaffold216_7	65.32	297	55	351	6.09E-135	NBS	different
At_RPP39	CNL	Sir	SI_C205065_2	69.307	303	247	549	8.09E-146	NBS	different
At_RPP39	CNL	Sir	SI_scaffold2334_4	64.463	363	1	363	4.91E-147	CN	different
At_RPP39	CNL	Sir	SI_C214951_1	67.227	357	248	599	3.69E-158	CNL	same
At_RPP39	CNL	Sir	SI_C217313_1	64.439	883	1	873	0	CNL	same
At_RPP39	CNL	Sir	SI_C196787_1	63.093	569	299	860	0	NL	different
At_RPP39	CNL	Sir	SI_scaffold2334_3	62.514	875	25	876	0	CNL	same
At_RPP39	CNL	Sir	SI_scaffold1010_13	60.105	569	314	870	0	NL	different
At_RPP8	CNL	Sir	SI_scaffold461_17	68.196	327	3	329	1.52E-150	CNL	same
At_RPP8	CNL	Sir	SI_scaffold1384_210	68.894	913	1	908	0	CNL	same
At_RPP8	CNL	Sir	SI_scaffold162_11	66.156	913	3	908	0	CNL	same
At_RPS2	NL	Sir	SI_scaffold2159_199	84.945	910	1	909	0	CNL	different
At_RPS5	TNL	Sir	SI_scaffold249_155	66.061	881	1	880	0	CNL	different
At_RPS5	TNL	Sir	SI_scaffold249_162	61.628	774	119	880	0	CNL	different
At_RPS5	TNL	Sir	SI_scaffold249_153	60.582	893	1	880	0	CNL	different
At_RPS5	TNL	Sir	SI_scaffold1240_19	60.043	931	1	881	0	CNL	different
At_SOBIR1	LRR-RLK	Sir	SI_scaffold3078_7	85.537	643	1	641	0	LRR-RLK	same
At_WRR12	TNL	Sir	SI_scaffold759_4	61.645	1009	1	989	0	TNL	same
At_WRR4a	TNL	Sir	SI_scaffold325_22	69.079	152	1	152	9.15E-58	TNL	same
At_WRR4a	TNL	Sir	SI_scaffold436_1	66.242	157	1	157	1.43E-60	TX	different
At_WRR4a	TNL	Sir	SI_scaffold1338_12	63.285	207	12	218	1.16E-77	TNL	same
At_WRR4a	TNL	Sir	SI_scaffold2804_1	62.185	238	8	245	6.76E-87	TNL	same
At_WRR4a	TNL	Sir	SI_scaffold1847_1	63.761	218	1	218	7.82E-92	TX	different
At_WRR4a	TNL	Sir	SI_scaffold1174_8	61.587	315	40	353	6.22E-125	TN	different
At_WRR4a	TNL	Sir	SI_scaffold146_21	60.155	517	7	521	0	TNL	same
At_WRR4b	TNL	Sir	SI_scaffold325_22	74.667	150	12	161	1.00E-61	TNL	same
At_WRR4b	TNL	Sir	SI_scaffold436_1	67.532	154	6	159	5.30E-62	TX	different
At_WRR4b	TNL	Sir	SI_scaffold1882_1	72.432	185	1	183	2.98E-88	TX	different
At_WRR4b	TNL	Sir	SI_scaffold1847_1	64.545	220	1	220	1.33E-94	TX	different
At_WRR4b	TNL	Sir	SI_scaffold2804_1	74.396	207	12	218	2.31E-95	TNL	same
At_WRR4b	TNL	Sir	SI_scaffold1174_8	66.349	315	42	355	4.93E-140	TN	different
At_WRR4b	TNL	Sir	SI_scaffold146_3	62.295	366	161	524	6.58E-144	NL	different
At_WRR4b	TNL	Sir	SI_scaffold146_21	67.057	513	11	522	0	TNL	same
At_WRR8	TNL	Sir	SI_scaffold1724_46	61.066	244	13	253	4.25E-85	TNL	same
At_WRR8	TNL	Sir	SI_scaffold862_17	61.838	925	5	918	0	TNL	same
At_WRR9	NL	Sir	SI_scaffold1882_1	67.027	185	5	186	1.05E-77	TX	different
At_WRR9	NL	Sir	SI_scaffold146_3	62.842	366	164	527	3.69E-140	NL	same

At_WRR9	NL	Sir	SI_scaffold325_12	61.039	1001	5	985	0	TNL	different
Bju_WRR1	CNL	Sir	SI_scaffold461_17	65.443	327	3	324	2.74E-139	CNL	same
Bju_WRR1	CNL	Sir	SI_scaffold162_11	61.184	912	3	900	0	CNL	same
Bju_WRR1	CNL	Sir	SI_scaffold1384_210	60.283	919	1	900	0	CNL	same
Bna_LepR3/Rlm2	LRR-RLP	Sir	SI_scaffold429_15	73.053	950	1	945	0	LRR-RLP	same
Bna_LepR3/Rlm2	LRR-RLP	Sir	SI_C226829_1	62.1	781	176	950	0	LRR-RLP	same
Bna_LepR3/Rlm2	LRR-RLP	Sir	SI_scaffold651_14	62.087	757	187	938	0	LRR-RLP	same
Bna_LepR3/Rlm2	LRR-RLP	Sir	SI_scaffold2443_28	61.869	674	276	947	0	LRR-RLP	same
Bna_LepR3/Rlm2	LRR-RLP	Sir	SI_C228887_1	61.869	674	276	947	0	LRR-RLP	same
Bna_LepR3/Rlm2	LRR-RLP	Sir	SI_scaffold1201_3	61.61	646	302	947	0	LRR-RLP	same
Bna_MPK9	Other-RLK	Sir	SI_C212699_1	81.081	407	100	506	0	Other-RLK	same
Bna_Rlm9/4/7	Other-RLK	Sir	SI_scaffold2049_126	75.513	829	1	794	0	Other-RLK	same
Bna_Rlm9/4/7	Other-RLK	Sir	SI_scaffold1597_89	63.317	796	17	793	0	Other-RLK	same
Bna_Rlm9/4/7	Other-RLK	Sir	SI_scaffold397_8	62.893	795	23	794	0	Other-RLK	same
Bna_Rlm9/4/7	Other-RLK	Sir	SI_scaffold1491_127	60.852	728	23	735	0	Other-RLK	same
Bol_FocBo1	TNL	Sir	SI_scaffold842_29	73.875	1378	5	1355	0	TNL	same
Bol_FocBo1	TNL	Sir	SI_scaffold842_25	70	400	10	409	0	TN	different
Bol_FocBo1	TNL	Sir	SI_C227795_1	64.309	905	5	907	0	TNL	same
Bra_cRa/cRb	TNL	Sir	SI_scaffold627_15	62.092	153	930	1082	1.18E-45	TNL	same
Bra_Crr1a	TNL	Sir	SI_scaffold627_18	63.964	222	676	894	1.92E-66	TNL	same
Bra_Crr1a	TNL	Sir	SI_scaffold627_18	60.821	268	676	939	6.61E-76	TNL	same
Bra_Crr1a	TNL	Sir	SI_scaffold101_105	66.379	232	12	213	2.89E-88	TX	different
Bra_Crr1a	TNL	Sir	SI_scaffold553_4	61.011	277	318	594	2.46E-101	NL	different
At_ADR1	NL	Sal	KAF8115254.1	61.501	813	6	787	0	NL	same
At_BAK1	LRR-RLK	Sal	KAF8098353.1	95.13	616	1	615	0	LRR-RLK	same
At_BAK1	LRR-RLK	Sal	KAF8097069.1	94.805	616	1	615	0	LRR-RLK	same
At_BAK1	LRR-RLK	Sal	KAF8050199.1	93.617	611	5	615	0	LRR-RLK	same
At_BAK1	LRR-RLK	Sal	KAF8112805.1	80.395	607	10	615	0	LRR-RLK	same
At_BAK1	LRR-RLK	Sal	KAF8091387.1	79.373	606	24	615	0	LRR-RLK	same
At_BAK1	LRR-RLK	Sal	KAF8118948.1	78.082	584	35	615	0	LRR-RLK	same
At_BAK1	LRR-RLK	Sal	KAF8090271.1	78.029	619	11	615	0	LRR-RLK	same
At_BAK1	LRR-RLK	Sal	KAF8049920.1	72.131	549	68	615	0	LRR-RLK	same
At_BAK1	LRR-RLK	Sal	KAF8049426.1	64.929	633	22	615	0	LRR-RLK	same
At_FLS2	LRR-RLK	Sal	KAF8117201.1	77.959	980	196	1170	0	LRR-RLK	same
At_NGR1a	RNL	Sal	KAF8101461.1	80.346	809	11	809	0	CNL	different
At_NGR1a	RNL	Sal	KAF8112165.1	70.579	656	1	636	0	CNL	different
At_NGR1a	RNL	Sal	KAF8085476.1	62.759	819	5	809	0	NL	different
At_NGR1b	RNL	Sal	KAF8101461.1	68.987	819	11	815	0	CNL	different
At_NGR1b	RNL	Sal	KAF8085476.1	61.642	816	11	815	0	NL	different
At_NGR1b	RNL	Sal	KAF8112165.1	61.002	659	5	642	0	CNL	different
At_PBS1	Other-RLK	Sal	KAF8047997.1	66.149	322	71	392	2.44E-158	Other-RLK	same
At_PBS1	Other-RLK	Sal	KAF8047998.1	60.541	370	28	392	1.54E-158	Other-RLK	same
At_PBS1	Other-RLK	Sal	KAF8116034.1	66.46	322	71	392	7.51E-159	Other-RLK	same
At_RAC1	TNL	Sal	KAF8118565.1	67.066	167	9	172	1.03E-67	TX	different
At_RAC1	TNL	Sal	KAF8044651.1	72.647	340	9	345	8.84E-170	TN	different
At_RFO1	Other-RLK	Sal	KAF8091446.1	62.441	213	542	750	2.48E-81	Other-RLK	same
At_RFO1	Other-RLK	Sal	KAF8103426.1	86.262	757	1	751	0	Other-RLK	same
At_RFO1	Other-RLK	Sal	KAF8083637.1	79.921	762	1	751	0	Other-RLK	same
At_RFO1	Other-RLK	Sal	KAF8094531.1	65.738	718	7	712	0	Other-RLK	same
At_RFO1	Other-RLK	Sal	KAF8064809.1	64.951	719	2	710	0	Other-RLK	same
At_RFO1	Other-RLK	Sal	KAF8094532.1	62.467	762	2	751	0	Other-RLK	same
At_RFO2	LRR-RLP	Sal	KAF8045120.1	60.569	246	504	749	2.49E-102	LRR-RLP	same
At_RFO2	LRR-RLP	Sal	KAF8106071.1	70.968	713	28	740	0	LRR-RLP	same
At_RFO2	LRR-RLP	Sal	KAF8080653.1	66.572	703	47	749	0	LRR-RLP	same
At_RFO2	LRR-RLP	Sal	KAF8099526.1	66.527	717	32	748	0	LRR-RLK	different
At_RFO2	LRR-RLP	Sal	KAF8114266.1	65.84	726	6	730	0	LRR-RLP	same
At_RFO3	Other-RLK	Sal	KAF8109710.1	61.441	236	515	750	1.37E-101	Other-RLK	same
At_RFO3	Other-RLK	Sal	KAF8083319.1	74.419	731	2	705	0	Other-RLK	same
At_RFO3	Other-RLK	Sal	KAF8080741.1	71.512	681	191	850	0	Other-RLK	same
At_RFO3	Other-RLK	Sal	KAF8107361.1	65.136	958	1	850	0	Other-RLK	same
At_RLM1a	TNL	Sal	KAF8087217.1	60.131	153	1	152	3.03E-58	TX	different
At_RLM1a	TNL	Sal	KAF8081485.1	64.286	154	5	158	1.12E-66	TX	different
At_RLM1a	TNL	Sal	KAF8094300.1	60.34	1001	3	993	0	TNL	same
At_RLM1b	TNL	Sal	KAF8082878.1	63.628	1031	1	993	0	TNL	same
At_RLM1b	TNL	Sal	KAF8094299.1	61.049	991	1	981	0	TNL	same
At_RLP1	LRR-RLP	Sal	KAF8104053.1	80.037	1082	1	1076	0	LRR-RLP	same
At_RLP23	LRR-RLP	Sal	KAF8096091.1	62.559	211	678	888	2.73E-81	LRR-RLP	same
At_RLP23	LRR-RLP	Sal	KAF8053593.1	71.579	380	509	888	0	LRR-RLP	same
At_RLP23	LRR-RLP	Sal	KAF8112028.1	69.451	874	19	890	0	LRR-RLP	same
At_RLP23	LRR-RLP	Sal	KAF8096085.1	68.65	874	19	890	0	LRR-RLP	same
At_RLP23	LRR-RLP	Sal	KAF8108771.1	68.276	870	18	887	0	LRR-RLP	same
At_RLP23	LRR-RLP	Sal	KAF8092581.1	67.95	883	1	881	0	LRR-RLP	same
At_RLP23	LRR-RLP	Sal	KAF8108774.1	67.808	876	17	890	0	LRR-RLP	same
At_RLP23	LRR-RLP	Sal	KAF8045493.1	65.694	720	175	889	0	LRR-RLP	same
At_RLP23	LRR-RLP	Sal	KAF8084401.1	64.831	708	182	888	0	LRR-RLP	same
At_RLP23	LRR-RLP	Sal	KAF8092574.1	64.779	724	171	889	0	LRR-RLP	same
At_RLP23	LRR-RLP	Sal	KAF8053603.1	64.505	586	290	874	0	LRR-RLP	same
At_RLP23	LRR-RLP	Sal	KAF8105233.1	63.7	719	175	889	0	LRR-RLP	same
At_RLP23	LRR-RLP	Sal	KAF8105234.1	63.352	704	175	876	0	LRR-RLP	same
At_RLP32	LRR-RLP	Sal	KAF8092002.1	70.136	221	634	854	1.64E-88	LRR-RLP	same
At_RLP32	LRR-RLP	Sal	KAF8085576.1	74.682	865	1	865	0	LRR-RLP	same
At_RLP32	LRR-RLP	Sal	KAF8085575.1	74.51	867	1	867	0	LRR-RLP	same
At_RLP32	LRR-RLP	Sal	KAF8083537.1	64.058	754	118	863	0	LRR-RLP	same
At_RLP32	LRR-RLP	Sal	KAF8106575.1	60.088	912	1	852	0	LRR-RLP	same
At_RLP42	LRR-RLP	Sal	KAF8096091.1	73.653	167	679	845	5.44E-82	LRR-RLP	same
At_RLP42	LRR-RLP	Sal	KAF8053593.1	73.973	365	512	876	0	LRR-RLP	same

At_RLP42	LRR-RLP	Sal	KAF8108774.1	68.354	869	18	880	0	LRR-RLP	same
At_RLP42	LRR-RLP	Sal	KAF8112028.1	67.857	868	19	880	0	LRR-RLP	same
At_RLP42	LRR-RLP	Sal	KAF8096085.1	67.166	868	19	880	0	LRR-RLP	same
At_RLP42	LRR-RLP	Sal	KAF8108771.1	66.047	860	18	876	0	LRR-RLP	same
At_RLP42	LRR-RLP	Sal	KAF8092581.1	64.739	882	1	877	0	LRR-RLP	same
At_RLP42	LRR-RLP	Sal	KAF8084401.1	64.023	706	175	879	0	LRR-RLP	same
At_RLP42	LRR-RLP	Sal	KAF8053603.1	63.621	580	291	868	0	LRR-RLP	same
At_RLP42	LRR-RLP	Sal	KAF8045493.1	63.319	717	183	889	0	LRR-RLP	same
At_RLP42	LRR-RLP	Sal	KAF8092574.1	62.762	717	183	889	0	LRR-RLP	same
At_RLP42	LRR-RLP	Sal	KAF8105234.1	62.572	692	182	870	0	LRR-RLP	same
At_RLP42	LRR-RLP	Sal	KAF8105233.1	62.375	699	182	876	0	LRR-RLP	same
At_RPM1	NL	Sal	KAF8094635.1	83.118	930	1	926	0	NL	same
At_RPP1	TNL	Sal	KAF8081485.1	60.39	154	89	242	5.32E-62	TX	different
At_RPP1	TNL	Sal	KAF8085339.1	66.071	168	94	261	9.92E-74	TX	different
At_RPP1	TNL	Sal	KAF8077018.1	72.193	187	94	280	2.26E-93	TX	different
At_RPP1	TNL	Sal	KAF8086279.1	69.589	365	95	459	1.03E-179	TN	different
At_RPP13	CNL	Sal	KAF8116607.1	68.421	836	1	829	0	CNL	same
At_RPP2a	TNL	Sal	KAF8077026.1	60.391	409	10	414	5.89E-146	TNL	same
At_RPP2a	TNL	Sal	KAF8102895.1	70.239	877	427	1300	0	TNL	same
At_RPP2a	TNL	Sal	KAF8102896.1	68.529	877	427	1300	0	TNL	same
At_RPP2b	TNL	Sal	KAF8102897.1	73.64	1195	1	1193	0	TNL	same
At_RPP39	CNL	Sal	KAF8044555.1	71.084	415	1	415	0	CN	different
At_RPP39	CNL	Sal	KAF8098363.1	65.84	887	1	870	0	CNL	same
At_RPP39	CNL	Sal	KAF8062888.1	64.972	885	1	878	0	CNL	same
At_RPP39	CNL	Sal	KAF8084217.1	62.76	913	1	870	0	CNL	same
At_RPP7	NL	Sal	KAF8116723.1	62.743	875	1	866	0	CNL	different
At_RPP7	NL	Sal	KAF8092678.1	61.859	624	1	620	0	NL	same
At_RPP8	CNL	Sal	KAF8080154.1	63.428	916	1	904	0	CNL	same
At_RPP8	CNL	Sal	KAF8105614.1	63.2	750	1	740	0	CNL	same
At_RPP8	CNL	Sal	KAF8101775.1	62.882	916	1	903	0	CNL	same
At_RPP8	CNL	Sal	KAF8105615.1	61.209	910	1	903	0	CNL	same
At_RPP8	CNL	Sal	KAF8105611.1	61.209	910	1	903	0	CNL	same
At_RPP8	CNL	Sal	KAF8093800.1	60.898	913	1	903	0	CNL	same
At_RPS2	NL	Sal	KAF8114000.1	83.956	910	1	909	0	CNL	different
At_RPS5	TNL	Sal	KAF8044716.1	65.163	399	1	399	0	CN	different
At_RPS5	TNL	Sal	KAF8087363.1	64.779	883	1	880	0	CNL	different
At_RPS5	TNL	Sal	KAF8111125.1	63.229	892	1	881	0	CNL	different
At_RPS5	TNL	Sal	KAF8098474.1	62.812	882	1	880	0	CNL	different
At_RPS5	TNL	Sal	KAF8058996.1	61.4	886	1	881	0	CNL	different
At_RPS5	TNL	Sal	KAF8098470.1	61.338	882	1	880	0	CNL	different
At_SOBIR1	LRR-RLK	Sal	KAF8096053.1	81.719	640	6	640	0	LRR-RLK	same
At_SOBIR1	LRR-RLK	Sal	KAF8086968.1	80.65	646	1	641	0	LRR-RLK	same
At_WRR12	TNL	Sal	KAF8114288.1	83.049	1056	3	1049	0	TNL	same
At_WRR4a	TNL	Sal	KAF8087217.1	62.581	155	1	153	1.53E-58	TX	different
At_WRR4a	TNL	Sal	KAF8081485.1	67.516	157	1	157	3.47E-69	TX	different
At_WRR4b	TNL	Sal	KAF8087217.1	63.576	151	4	153	2.18E-60	TX	different
At_WRR4b	TNL	Sal	KAF8081485.1	68.831	154	6	159	8.74E-71	TX	different
At_WRR8	TNL	Sal	KAF8118565.1	68.675	166	20	182	3.94E-72	TX	different
At_WRR8	TNL	Sal	KAF8044651.1	72.807	342	19	357	2.44E-173	TN	different
Bju_WRR1	CNL	Sal	KAF8101775.1	82.508	909	1	905	0	CNL	same
Bju_WRR1	CNL	Sal	KAF8105611.1	70.374	908	1	904	0	CNL	same
Bju_WRR1	CNL	Sal	KAF8105615.1	69.846	912	1	904	0	CNL	same
Bju_WRR1	CNL	Sal	KAF8105614.1	69.721	753	1	745	0	CNL	same
Bna_LepR3/Rlm2	LRR-RLP	Sal	KAF8092002.1	66.239	234	718	950	1.86E-85	LRR-RLP	same
Bna_LepR3/Rlm2	LRR-RLP	Sal	KAF8100137.1	71.067	712	1	708	0	LRR-RLP	same
Bna_LepR3/Rlm2	LRR-RLP	Sal	KAF8085575.1	60.302	796	163	948	0	LRR-RLP	same
Bna_LepR3/Rlm2	LRR-RLP	Sal	KAF8085576.1	60.302	796	163	948	0	LRR-RLP	same
Bna_Rlm9/4/7	Other-RLK	Sal	KAF8091446.1	69.955	223	575	793	6.20E-95	Other-RLK	same
Bna_Rlm9/4/7	Other-RLK	Sal	KAF8103088.1	84.757	761	1	760	0	Other-RLK	same
Bol_FocBo1	TNL	Sal	KAF8081485.1	63.333	150	5	154	2.80E-60	TX	different
Bol_FocBo1	TNL	Sal	KAF8087217.1	64.43	149	5	152	1.70E-63	TX	different
Bra_cRa/cRb	TNL	Sal	KAF8085339.1	64.848	165	73	237	3.81E-67	TX	different
Bra_cRa/cRb	TNL	Sal	KAF8077018.1	63.784	185	73	257	1.13E-75	TX	different
Bra_cRa/cRb	TNL	Sal	KAF8101393.1	66.821	1296	1	1268	0	TNL	same
Bra_cRa/cRb	TNL	Sal	KAF8114966.1	64.456	754	1	734	0	TNL	same
Bra_Crr1a	TNL	Sal	KAF8087217.1	60.897	156	57	211	1.34E-61	TX	different
Bra_Crr1a	TNL	Sal	KAF8087555.1	65.541	148	67	214	3.34E-64	TX	different
Bra_Crr1a	TNL	Sal	KAF8085339.1	64.458	166	67	232	2.18E-70	TX	different
Bra_Crr1a	TNL	Sal	KAF8077018.1	64.583	192	67	258	1.72E-82	TX	different
Bra_Crr1a	TNL	Sal	KAF8090866.1	60.082	243	68	310	1.45E-90	TN	different
Bra_Crr1a	TNL	Sal	KAF8114966.1	80.15	665	66	730	0	TNL	same
Bra_Crr1a	TNL	Sal	KAF8114969.1	64.056	1213	1	1090	0	Other-NLR	different
Bra_Crr1a	TNL	Sal	KAF8082977.1	61.825	1260	66	1214	0	TNL	same
At_BAK1	LRR-RLK	Tar	Ta02674	95.61	615	1	615	0	LRR-RLK	same
At_BAK1	LRR-RLK	Tar	Ta11929	77.419	620	24	615	0	LRR-RLK	same
At_BAK1	LRR-RLK	Tar	Ta19321	74.92	622	13	615	0	LRR-RLK	same
At_BAK1	LRR-RLK	Tar	Ta25247	64.646	594	10	555	0	LRR-RLK	same
At_FLS2	LRR-RLK	Tar	Ta04995	81.624	1170	1	1166	0	LRR-RLK	same
At_NGR1a	RNL	Tar	Ta02427	67.07	413	404	809	3.01E-178	CNL	different
At_NGR1a	RNL	Tar	Ta02428	77.985	804	12	809	0	CNL	different
At_NGR1a	RNL	Tar	Ta02427	65.239	817	7	803	0	CNL	different
At_NGR1b	RNL	Tar	Ta02427	60.412	437	387	815	2.77E-163	CNL	different
At_NGR1b	RNL	Tar	Ta02428	67.611	812	14	815	0	CNL	different
At_NGR1b	RNL	Tar	Ta02427	64.015	817	9	810	0	CNL	different
At_RAC1	TNL	Tar	Ta08988	68.345	278	160	437	2.54E-102	NL	different
At_RAC1	TNL	Tar	Ta24801	60.567	388	9	393	2.66E-145	TX	different

At_RFO1	Other-RLK	Tar	Ta03721	81.743	241	446	686	7.10E-127	Other-RLK	same
At_RFO1	Other-RLK	Tar	Ta03916	60.187	427	325	747	1.27E-154	Other-RLK	same
At_RFO1	Other-RLK	Tar	Ta06877	87.225	728	22	740	0	Other-RLK	same
At_RFO1	Other-RLK	Tar	Ta06876	73.881	402	309	698	0	Other-RLK	same
At_RFO1	Other-RLK	Tar	Ta03721	68.543	693	12	696	0	Other-RLK	same
At_RFO1	Other-RLK	Tar	Ta03721	64.636	755	10	751	0	Other-RLK	same
At_RFO1	Other-RLK	Tar	Ta03721	63.102	748	1	715	0	Other-RLK	same
At_RFO2	LRR-RLP	Tar	Ta20922	72.184	719	19	737	0	LRR-RLK	different
At_RFO2	LRR-RLP	Tar	Ta03822	70.822	754	1	753	0	LRR-RLP	same
At_RFO3	Other-RLK	Tar	Ta08744	60.173	231	515	745	1.07E-82	Other-RLK	same
At_RFO3	Other-RLK	Tar	Ta16704	71.547	847	30	850	0	Other-RLK	same
At_RFO3	Other-RLK	Tar	Ta16704	68.303	937	1	849	0	Other-RLK	same
At_RLM1a	TNL	Tar	Ta16523	60.234	171	16	183	1.53E-52	TN	different
At_RLM1a	TNL	Tar	Ta25952	63.087	149	10	158	4.55E-59	TX	different
At_RLM1a	TNL	Tar	Ta16522	76.159	151	8	158	2.98E-76	TX	different
At_RLM1a	TNL	Tar	Ta16523	69.466	262	164	423	3.97E-105	TN	different
At_RLM1b	TNL	Tar	Ta16523	60.465	172	15	184	1.59E-53	TN	different
At_RLM1b	TNL	Tar	Ta16523	68.679	265	164	426	7.70E-104	TN	different
At_RLM1b	TNL	Tar	Ta15205	60.211	284	437	720	7.64E-108	LRR-RLP	different
At_RLM1b	TNL	Tar	Ta15202	63.58	648	372	1009	0	NL	different
At_RLM3	TN	Tar	Ta19917	60.432	278	159	427	3.56E-94	NL	different
At_RLM3	TN	Tar	Ta20013	60.65	277	159	427	1.44E-105	NL	different
At_RLP1	LRR-RLP	Tar	Ta12797	73.426	1080	1	1077	0	LRR-RLP	same
At_RLP23	LRR-RLP	Tar	Ta22122	66.168	869	22	890	0	LRR-RLP	same
At_RLP23	LRR-RLP	Tar	Ta13179	65.266	714	175	885	0	LRR-RLP	same
At_RLP23	LRR-RLP	Tar	Ta20228	63.164	866	20	874	0	LRR-RLP	same
At_RLP30	LRR-RLP	Tar	Ta11898	66.514	218	569	783	1.81E-73	LRR-RLP	same
At_RLP30	LRR-RLP	Tar	Ta11897	69.5	200	581	779	6.24E-85	LRR-RLP	same
At_RLP30	LRR-RLP	Tar	Ta13655	61.314	274	485	754	1.59E-105	LRR-RLP	same
At_RLP32	LRR-RLP	Tar	Ta23201	77.181	149	655	803	6.87E-69	LRR-RLP	same
At_RLP32	LRR-RLP	Tar	Ta14238	73.016	189	418	606	2.72E-77	LRR-RLP	same
At_RLP32	LRR-RLP	Tar	Ta16681	63.559	236	612	846	4.76E-83	LRR-RLP	same
At_RLP32	LRR-RLP	Tar	Ta27115	61.635	318	159	475	2.63E-106	LRR-RLP	same
At_RLP32	LRR-RLP	Tar	Ta27222	71.605	243	612	854	5.76E-117	LRR-RLP	same
At_RLP32	LRR-RLP	Tar	Ta23208	63.784	370	159	527	1.37E-136	LRR-RLP	same
At_RLP32	LRR-RLP	Tar	Ta23209	70.06	668	187	853	0	LRR-RLP	same
At_RLP32	LRR-RLP	Tar	Ta16684	69.973	736	114	848	0	LRR-RLP	same
At_RLP32	LRR-RLP	Tar	Ta19632	68.732	710	159	866	0	LRR-RLP	same
At_RLP32	LRR-RLP	Tar	Ta14240	66.98	745	113	856	0	LRR-RLP	same
At_RLP32	LRR-RLP	Tar	Ta16686	66.234	770	90	852	0	LRR-RLP	same
At_RLP32	LRR-RLP	Tar	Ta26856	65.672	737	113	848	0	LRR-RLP	same
At_RLP32	LRR-RLP	Tar	Ta17948	65.195	589	281	868	0	LRR-RLP	same
At_RLP32	LRR-RLP	Tar	Ta14238	64.175	709	42	744	0	LRR-RLP	same
At_RLP32	LRR-RLP	Tar	Ta14238	63.931	865	4	866	0	LRR-RLP	same
At_RLP42	LRR-RLP	Tar	Ta22122	64.245	867	18	880	0	LRR-RLP	same
At_RLP42	LRR-RLP	Tar	Ta13179	63.623	701	182	876	0	LRR-RLP	same
At_RLP42	LRR-RLP	Tar	Ta20228	62.545	841	37	868	0	LRR-RLP	same
At_RPM1	NL	Tar	Ta04171	83.459	931	1	926	0	NL	same
At_RPP1	TNL	Tar	Ta23923	71.975	157	85	241	4.17E-73	TX	different
At_RPP1	TNL	Tar	Ta23545	60.905	243	248	490	2.65E-93	NBS	different
At_RPP13	CNL	Tar	Ta23819	65.333	375	1	375	2.51E-151	CN	different
At_RPP13	CNL	Tar	Ta21221	66.228	835	1	830	0	CNL	same
At_RPP2a	TNL	Tar	Ta19917	64.706	255	161	414	9.00E-91	NL	different
At_RPP2a	TNL	Tar	Ta19921	63.386	254	161	414	3.09E-91	NL	different
At_RPP2a	TNL	Tar	Ta20013	65.354	254	161	414	7.22E-99	NL	different
At_RPP2a	TNL	Tar	Ta10587	68.592	277	138	414	3.12E-102	NL	different
At_RPP2a	TNL	Tar	Ta12833	80	245	1056	1300	1.81E-122	TNL	same
At_RPP2a	TNL	Tar	Ta13049	72.606	825	476	1299	0	TNL	same
At_RPP2a	TNL	Tar	Ta12836	71.413	906	408	1299	0	TNL	same
At_RPP2b	TNL	Tar	Ta12832	78.404	426	752	1165	0	NL	different
At_RPP2b	TNL	Tar	Ta12834	74.979	1187	1	1185	0	Other-NLR	different
At_RPP2b	TNL	Tar	Ta13049	73.391	1041	127	1165	0	TNL	same
At_RPP39	CNL	Tar	Ta19294	77.37	327	171	497	3.08E-178	CNL	same
At_RPP39	CNL	Tar	Ta19295	67.098	851	42	868	0	CNL	same
At_RPP4	TNL	Tar	Ta10587	61.168	291	812	1100	1.40E-93	NL	different
At_RPP4	TNL	Tar	Ta19917	64.539	282	163	443	2.90E-103	NL	different
At_RPP5	TNL	Tar	Ta19917	66.667	279	166	443	5.66E-104	NL	different
At_RPP7	NL	Tar	Ta23683	63.291	158	12	168	9.80E-52	NL	same
At_RPP7	NL	Tar	Ta25896	63.714	700	1	694	0	NL	same
At_RPP8	CNL	Tar	Ta18815	73.684	247	1	247	2.03E-125	CN	different
At_RPP8	CNL	Tar	Ta01723	65.359	918	1	906	0	NL	different
At_RPS2	NL	Tar	Ta01530	85.479	909	1	909	0	CNL	different
At_RPS4	TNL	Tar	Ta12930	65.145	241	953	1193	1.01E-93	NL	different
At_RPS4	TNL	Tar	Ta12930	61.854	1025	129	1139	0	NL	different
At_RPS5	TNL	Tar	Ta25636	72.237	371	65	432	0	NBS	different
At_RRS1	TNL	Tar	Ta12928	68.132	273	497	766	2.62E-112	NL	different
At_SOBIR1	LRR-RLK	Tar	Ta14326	85.358	642	1	641	0	LRR-RLK	same
At_WRR4b	TNL	Tar	Ta16523	61.272	173	16	185	9.26E-55	TN	different
At_WRR4b	TNL	Tar	Ta25952	65.101	149	11	159	8.91E-61	TX	different
At_WRR4b	TNL	Tar	Ta16523	62.205	254	165	416	1.06E-84	TN	different
At_WRR8	TNL	Tar	Ta08988	67.021	282	170	451	7.48E-105	NL	different
At_WRR8	TNL	Tar	Ta24801	63.239	389	20	404	7.20E-155	TX	different
At_WRR9	NL	Tar	Ta16523	61.132	265	168	430	1.32E-88	TN	different
Bju_WRR1	CNL	Tar	Ta18815	70.492	244	1	239	1.32E-117	CN	different
Bju_WRR1	CNL	Tar	Ta09903	61.079	686	141	813	0	NL	different
Bna_LepR3/Rlm2	LRR-RLP	Tar	Ta14238	64.398	191	502	692	4.29E-65	LRR-RLP	same

Bna_LepR3/Rlm2	LRR-RLP	Tar	Ta23201	68.478	184	738	920	8.35E-69	LRR-RLP	same
Bna_LepR3/Rlm2	LRR-RLP	Tar	Ta27222	66.016	256	696	950	5.93E-112	LRR-RLP	same
Bna_LepR3/Rlm2	LRR-RLP	Tar	Ta23208	60.49	367	247	610	5.89E-116	LRR-RLP	same
Bna_LepR3/Rlm2	LRR-RLP	Tar	Ta19632	65.581	706	247	948	0	LRR-RLP	same
Bna_LepR3/Rlm2	LRR-RLP	Tar	Ta14238	64.721	754	198	948	0	LRR-RLP	same
Bna_LepR3/Rlm2	LRR-RLP	Tar	Ta23209	64.157	664	276	938	0	LRR-RLP	same
Bna_LepR3/Rlm2	LRR-RLP	Tar	Ta16684	63.09	848	106	950	0	LRR-RLP	same
Bna_LepR3/Rlm2	LRR-RLP	Tar	Ta17948	62.544	566	370	931	0	LRR-RLP	same
Bna_LepR3/Rlm2	LRR-RLP	Tar	Ta16686	62.423	809	137	933	0	LRR-RLP	same
Bna_LepR3/Rlm2	LRR-RLP	Tar	Ta14238	61.201	683	148	827	0	LRR-RLP	same
Bna_Rlm9/4/7	Other-RLK	Tar	Ta03721	74.699	249	477	725	1.94E-114	Other-RLK	same
Bna_Rlm9/4/7	Other-RLK	Tar	Ta03916	66.412	393	401	790	7.22E-149	Other-RLK	same
Bna_Rlm9/4/7	Other-RLK	Tar	Ta06876	70.277	397	344	730	0	Other-RLK	same
Bna_Rlm9/4/7	Other-RLK	Tar	Ta22586	64.653	778	28	793	0	Other-RLK	same
Bna_Rlm9/4/7	Other-RLK	Tar	Ta22585	63.602	805	1	793	0	Other-RLK	same
Bna_Rlm9/4/7	Other-RLK	Tar	Ta06877	61.675	788	23	794	0	Other-RLK	same
Bol_FocBo1	TNL	Tar	Ta19917	60.3	267	516	780	1.04E-88	NL	different
Bol_FocBo1	TNL	Tar	Ta19917	74.483	290	160	448	6.67E-127	NL	different
Bol_FocBo1	TNL	Tar	Ta19921	72.509	291	160	450	3.11E-131	NL	different
Bra_Crr1a	TNL	Tar	Ta16118	60.377	212	585	793	6.08E-69	NL	different
At_BAK1	LRR-RLK	Tpa	c0014_00534	95.447	615	1	615	0	LRR-RLK	same
At_BAK1	LRR-RLK	Tpa	c0010_00354	79.736	607	10	615	0	LRR-RLK	same
At_BAK1	LRR-RLK	Tpa	c0122_00010	79.008	605	24	615	0	LRR-RLK	same
At_BAK1	LRR-RLK	Tpa	c0012_00490	79.008	605	24	615	0	LRR-RLK	same
At_BAK1	LRR-RLK	Tpa	c0001_02630	73.226	620	11	615	0	LRR-RLK	same
At_FLS2	LRR-RLK	Tpa	c0003_01788	81.392	790	386	1173	0	LRR-RLK	same
At_NGR1a	RNL	Tpa	c0003_00095	78.773	815	1	809	0	NL	different
At_NGR1a	RNL	Tpa	c0362_00004	77.273	814	1	809	0	NL	different
At_NGR1a	RNL	Tpa	c0003_00094	68.069	808	15	809	0	CNL	different
At_NGR1b	RNL	Tpa	c0003_00095	67.072	823	5	815	0	NL	different
At_NGR1b	RNL	Tpa	c0362_00004	65.45	822	5	815	0	NL	different
At_NGR1b	RNL	Tpa	c0003_00094	62.699	815	16	815	0	CNL	different
At_RFO1	Other-RLK	Tpa	c0001_01253	63.66	754	8	751	0	Other-RLK	same
At_RFO1	Other-RLK	Tpa	c0001_01254	62.74	730	7	718	0	Other-RLK	same
At_RFO2	LRR-RLP	Tpa	c0187_00006	65.765	739	13	748	0	LRR-RLK	different
At_RFO2	LRR-RLP	Tpa	c0012_00540	65.765	739	13	748	0	LRR-RLK	different
At_RFO2	LRR-RLP	Tpa	c0001_01341	64.267	750	6	754	0	LRR-RLP	same
At_RFO3	Other-RLK	Tpa	c0005_01418	66.784	855	15	850	0	Other-RLK	same
At_RLM1a	TNL	Tpa	c0009_00368	60.819	171	13	183	7.92E-64	TX	different
At_RLM1a	TNL	Tpa	c0026_00082	69.613	181	1	181	2.42E-87	TX	different
At_RLM1a	TNL	Tpa	c0007_00316	60.274	438	187	621	0	NL	different
At_RLM1b	TNL	Tpa	c0026_00082	76.536	179	1	179	1.77E-93	TX	different
At_RLM1b	TNL	Tpa	c0026_00081	66.176	1020	7	1009	0	Other-NLR	different
At_RLM3	TN	Tpa	c0006_00829	64.103	156	1	153	3.17E-62	TX	different
At_RLP1	LRR-RLP	Tpa	c0001_00531	79.573	1077	1	1077	0	LRR-RLP	same
At_RLP1	LRR-RLP	Tpa	c0136_00002g	67.864	1002	24	1023	0	LRR-RLP	same
At_RLP23	LRR-RLP	Tpa	c0004_01429	64.068	295	175	467	4.92E-106	LRR-RLP	same
At_RLP23	LRR-RLP	Tpa	c0166_00004	69.495	872	1	868	0	LRR-RLP	same
At_RLP23	LRR-RLP	Tpa	c0004_01478	69.495	872	1	868	0	LRR-RLP	same
At_RLP30	LRR-RLP	Tpa	c0012_00051	65.605	157	630	786	2.75E-68	LRR-RLP	same
At_RLP32	LRR-RLP	Tpa	c0246_00007	64.753	871	9	852	0	LRR-RLP	same
At_RLP32	LRR-RLP	Tpa	c0016_00305	61.547	879	1	852	0	LRR-RLP	same
At_RLP42	LRR-RLP	Tpa	c0004_01429	61.458	288	182	467	2.08E-99	LRR-RLP	same
At_RLP42	LRR-RLP	Tpa	c0166_00004	67.664	869	1	862	0	LRR-RLP	same
At_RLP42	LRR-RLP	Tpa	c0004_01478	67.664	869	1	862	0	LRR-RLP	same
At_RPP1	TNL	Tpa	c0005_00285	60.697	201	64	264	1.11E-80	TX	different
At_RPP13	CNL	Tpa	c0007_00166	62.102	847	1	833	0	NL	different
At_RPP2a	TNL	Tpa	c0006_00829	66.234	154	2	155	1.06E-66	TX	different
At_RPP2a	TNL	Tpa	c0006_00830	67.843	255	161	414	3.19E-96	NL	different
At_RPP39	CNL	Tpa	c0015_00366	65.877	211	1	211	2.76E-87	CNL	same
At_RPP39	CNL	Tpa	c0015_00307	75.214	234	1	234	3.31E-120	CN	different
At_RPP39	CNL	Tpa	c1198_00001	74.194	279	1	279	8.71E-145	CN	different
At_RPP4	TNL	Tpa	c0006_00829	68.182	154	3	156	1.93E-68	TX	different
At_RPP5	TNL	Tpa	c0006_00829	71.069	159	1	159	3.02E-70	TX	different
At_RPP8	CNL	Tpa	c0003_02103	60.625	160	216	371	3.06E-57	NL	different
At_RPP8	CNL	Tpa	c0023_00078	77.381	252	1	252	2.10E-130	CN	different
At_RPS2	NL	Tpa	c0006_01486	84.048	909	1	909	0	CNL	different
At_SOBIR1	LRR-RLK	Tpa	c0004_01545	83.488	648	1	641	0	LRR-RLK	same
At_WRR12	TNL	Tpa	c0016_00185	62.064	688	11	691	0	TNL	same
At_WRR4a	TNL	Tpa	c0026_00082	64.641	181	1	180	1.53E-78	TX	different
At_WRR4b	TNL	Tpa	c0009_00368	61.818	165	14	178	7.07E-65	TX	different
At_WRR4b	TNL	Tpa	c0026_00082	70.556	180	1	180	9.11E-88	TX	different
At_WRR4b	TNL	Tpa	c0026_00083	65.289	363	12	370	1.66E-154	TNL	same
At_WRR9	NL	Tpa	c0026_00082	64.804	179	5	183	1.29E-76	TX	different
At_WRR9	NL	Tpa	c0026_00083	62.259	363	15	373	4.56E-146	TNL	different
Bju_WRR1	CNL	Tpa	c0023_00078	72.332	253	1	248	2.93E-122	CN	different
Bna_LepR3/Rlm2	LRR-RLP	Tpa	c0246_00007	62.371	776	180	948	0	LRR-RLP	same
Bna_MPK9	Other-RLK	Tpa	c0002_01170	80.841	428	82	506	0	Other-RLK	same
Bol_FocBo1	TNL	Tpa	c0006_00830	78.012	1187	160	1341	0	NL	different
Bra_cRa/cRb	TNL	Tpa	c0005_00285	60.674	178	61	237	2.77E-68	TX	different
Bra_Crr1a	TNL	Tpa	c0005_00285	65.116	172	62	232	6.13E-75	TX	different
Bra_Crr1a	TNL	Tpa	c0006_01108	64.366	1159	66	1217	0	TNL	same

*note: Aha=Arabidopsis halleri, Aly=Arabidopsis lyrata, Aal=Arabis alpina, Bvu=Barbarea vulgaris, Bst=Boechera stricta, Bcr=Brassica cretica, Csa=Camelina sativa, Cgr=Capsella grandiflora, Cbp=Capsella bursa-pastoris,Cru= Capsella rubella, Chi=Cardamine hirsuta, Esa=Eutrema salsugineum, Lal=Leavenworthia alabamica, Lme=Lepidium meyenii, Rra=Raphanus raphanistrum, Rsa=Raphanus sativus, Sal=Sinapis alba, Sir=Sisymbrium irio, Spa=Schrenkiella parvula, Tpa=Thlaspi arvense, Main RGA classes, NLR= nucleotide-binding site (NBS) -leucine rich repeats (LRR), RLK= receptor-like protein kinases (RLKs), RLP= receptor-like proteins. NLRs subclasses, CN= coiled-coil (CC)-NBS, CNL, NBS, NL= NBS-LRR, TNL= Toll/Interleukin-1 receptor (TIR)-NBS-LRR, TN= TIR-NBS, TX= TIR with unknown domains, Other-NLR= NLR with other domains; RLK subclasses, LRR-RLK, Lysin motif (Lysm)-RLK, Other (other-receptor)-RLK; RLP subclasses, LRR-RLP and Lysm-RLP.
